# Glucosamine Yield Improvement in Engineered *Saccharomyces cerevisiae* with Ethanol Yield Reduction by Carbon Flux Redistribution

**DOI:** 10.3390/foods15071163

**Published:** 2026-03-30

**Authors:** Mingsi Ke, Xinyue Zheng, Jiaqi Feng, Jieshun Cheng, Peizhou Yang

**Affiliations:** Anhui Province Key Laboratory of Agricultural Products Modern Processing, School of Food and Biological Engineering, Hefei University of Technology, Hefei 230601, China; 2024111243@mail.hfut.edu.cn (M.K.); 15256592071@163.com (X.Z.); fengjqi@126.com (J.F.); jieshun@hfut.edu.cn (J.C.)

**Keywords:** *Saccharomyces cerevisiae*, glucosamine, CRISPR-Cas9, pyruvate decarboxylase, transcriptomic

## Abstract

Glucosamine (GlcN) is an essential amino monosaccharide widely used in pharmaceuticals, nutraceuticals, and cosmetics. Microbial fermentation presents a sustainable alternative to its traditional chemical production. However, in *Saccharomyces cerevisiae*, competitive carbon flux towards ethanol significantly limits GlcN yields. In this study, an *S. cerevisiae* strain for GlcN biosynthesis was engineered by integrating heterologous *GlmD* (glucosamine-6-phosphate deaminase) and *GlmP* (glucosamine-6-phosphate phosphatase) genes. To redirect carbon flux, the pyruvate decarboxylase genes *pdc1*, *pdc*5, and *pdc6* were sequentially knocked out using the Clustered Regularly Interspaced Short Palindromic Repeats Cas9 (CRISPR-Cas9) approach, generating strains *S. cerevisiae* *GlmDP/pdc1Δ*, *GlmDP/pdc1Δpdc5Δ*, and *GlmDP/pdc1Δpdc5Δpdc6Δ*. *S. cerevisiae GlmDP/pdc1Δpdc5Δpdc6Δ* achieved a GlcN titer of 2.20 ± 0.11 g/L, a 1.54-fold increase over the parental *S. cerevisia GlmDP* strain, while its ethanol yield decreased by 26%. This enhancement was achieved without significantly affecting cell growth or glucose consumption. Comparative transcriptomics between the triple-knockout and parental yeasts revealed 892 differentially expressed genes. Pathways related to glycolysis and ethanol formation were predominantly downregulated, whereas pathways potentially supporting GlcN synthesis were upregulated. The engineered strain demonstrated high genetic stability over 50 generations. Our findings demonstrate that disrupting ethanol formation is an effective strategy to enhance GlcN production in *S. cerevisiae*, providing valuable insights for carbon flux redistribution.

## 1. Introduction

Glucosamine (GlcN), an amino monosaccharide derived from glucose, is a fundamental building block for glycosaminoglycans in cartilage, tendons, and ligaments [1,2]. It is widely used in the nutraceutical, pharmaceutical, and cosmetic industries, primarily as glucosamine sulfate and N-acetylglucosamine (GlcNAc) [1,3]. Traditional GlcN production relies on the chemical hydrolysis of chitin from crustacean shells [4,5], a process involving harsh acids and alkalis at high temperatures, which poses significant environmental burdens and allergen risks [6]. In contrast, microbial fermentation offers a sustainable, green, and scalable alternative, providing consistent quality and eliminating allergen concerns associated with shellfish sources [7].

Recent advances in metabolic engineering have enabled efficient GlcN production in various microbial hosts, including *Bacillus subtilis* [8], *Saccharomyces cerevisiae* [9], and *Escherichia coli* [10], through enzyme engineering and pathway optimization. *S. cerevisiae* is a particularly attractive chassis due to its Generally Recognized as Safe (GRAS) status, well-characterized genetics, rapid growth, and robust fermentation capabilities [11].

The process conditions of medium composition, inoculum size, temperature, pH, and dissolved oxygen level are critical factors influencing production efficiency [12]. In addition, the metabolic pathway of GlcN-producing strains is directly related to product generation [13]. A feasible approach in engineering *S. cerevisiae* for GlcN production is offered by its innate tendency to divert substantial carbon flux toward ethanol fermentation [14,15]. This competitive pathway limits the availability of precursors, such as glucose-6-phosphate, for GlcN synthesis. Strategies to redirect central carbon metabolism away from ethanol formation are therefore essential. Pyruvate decarboxylase (PDC), encoded by the genes *pdc1*, *pdc5*, and *pdc6* in *S. cerevisiae* [16,17], catalyzes the decarboxylation of pyruvate to acetaldehyde, a key step in ethanol fermentation [18]. Targeted disruption of these genes presents a promising approach to reduce ethanol synthesis and rechannel carbon flux toward desired products.

**Figure 1:**
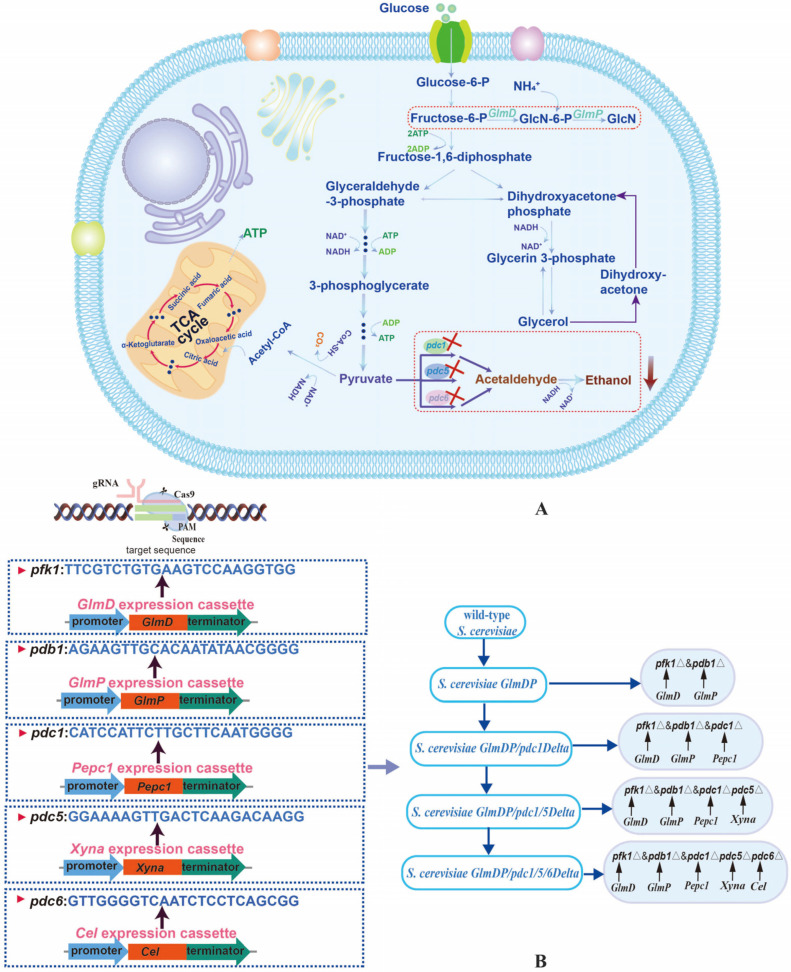
Modification strategy and construction pathway for engineered *S. cerevisiae GlmDP/pdc1/5/6Δ*. Note: (**A**) Strategy for enhancing GlcN and reducing ethanol via CRISPR-Cas9. (**B**) Sequential construction process involving *GlmD/GlmP* integration and *pdc1*, *pdc5*, and *pdc6* deletion.

In this study, an engineered *S. cerevisiae* strain (*GlmDP*) was first constructed by integrating heterologous *GlmD* and *GlmP* expression cassettes to enable GlcN biosynthesis. Subsequently, *pdc1*, *pdc5*, and *pdc6* were sequentially deleted using CRISPR-Cas9 to attenuate ethanol formation (Figure 1A). The effects of these deletions on GlcN titers, ethanol yields, cell growth, and substrate consumption were investigated. Furthermore, comparative transcriptomics was employed to elucidate the global transcriptional changes and metabolic reprogramming underlying the improved phenotype. This work provides an effective metabolic engineering strategy to enhance GlcN production in *S. cerevisiae* by minimizing carbon loss to ethanol.

## 2. Materials and Methods

### 2.1. Strains, Plasmids, Primers, Reagents, and Instruments

A haploid *S. cerevisiae* strain was used as the host for constructing all engineered strains in this study. *Escherichia coli DH5α* was used for plasmid propagation. The Cas9 expression plasmid (Cas9-NAT) carried a nourseothricin resistance marker, while the gRNA vector (gRNA-trp-Hyb) contained a hygromycin B resistance gene. *S. cerevisiae* strains were cultivated in YPD medium prepared from 1% yeast extract (*w*/*v*), 2% peptone (*w*/*v*), and 2% glucose (*w*/*v*) at 30 °C with a shaking speed of 180 rpm. Solid media contained 2% agar. Antibiotics were used at the following concentrations for selection: hygromycin B (100 μg/mL) and nourseothricin (80 μg/mL). Primer synthesis and PCR amplification kits were provided by Sangon Biotechnology (Shanghai, China). The Electrophoresis Instrument (EPS300) and Gel Imager (Universal Hood II) were manufactured by Bio-Rad Company (Hercules, CA, USA). The Polymerase Chain Reaction (PCR) Instrument (TC-96) and UV-Vis Spectrophotometer (UV-1201) were sourced from BIOMETRA Company (Göttingen, Germany). A high-performance liquid chromatography (HPLC) system was obtained from Agilent 1260, Santa Clara, CA, USA. All other reagents were of analytical grade.

### 2.2. gRNA Design and Plasmid Construction

The 23 bp guide RNA (gRNA) sequences targeting *S. cerevisiae pdc1*, *pdc5*, *pdc6, pfk1*, and *pdb1* were designed using the online tool CHOPCHOP (http://chopchop.cbu.uib.no/). Primers containing these target sequences fused to 23 bp vector homology arms were synthesized (Table 1). The gRNA expression cassettes were amplified via PCR using the gRNA-trp-Hyb plasmid as a template. The PCR reaction system consisted of 12.5 μL Phusion master mix, 1 μL each of forward and reverse primers, 0.5 μL template DNA, and 10 μL nuclease-free water. The thermal cycling conditions were: initial denaturation at 95 °C for 30 s; 30 cycles of 95 °C for 15 s, 56 °C for 15 s, and 72 °C for 4.5 min; and a final extension at 72 °C for 5 min.

### 2.3. Yeast Transformation and Strain Construction

Exogenous genes and plasmid vectors were introduced into yeast using the LiAc/SSDNA-PEG-mediated transformation method [19]. The Cas9-NAT and gRNA-trp-HyB plasmids were transformed via antibiotic selection on YPD plates supplemented with nourseothricin (NAT) and hygromycin B (HyB), respectively [20]. The specific transformation mixture consisted of 240 µL of 50% PEG 3350 (*w*/*v*), 36 µL of 1 M LiAc, 50 µL of single-stranded carrier DNA, 2 µL of plasmid DNA, and 32 µL of sterile water. Putative transformants were selected on solid YPD medium containing 100 μg/mL hygromycin and 80 μg/mL nourseothricin. Positive clones were confirmed by sequencing the targeted gene regions after PCR amplification. The engineered strain *S. cerevisiae GlmDP* was constructed by integrating the *GlmD* and *GlmP* expression cassettes. Subsequently, *S. cerevisiae GlmDP/pdc1Δ* was generated by deleting the *pdc1* gene in *S. cerevisiae GlmDP*. Sequential deletion of *pdc5* and *pdc6* yielded *S. cerevisiae GlmDP/pdc1/5Δ* and *S. cerevisiae GlmDP/pdc1/5/6Δ*, respectively (Figure 1B).

### 2.4. Comparison of GlcN Production Among Engineered S. cerevisiae Strains

Glucosamine (GlcN) production was compared among five strains: the wild type, *S. cerevisiae GlmDP*, *GlmDP/pdc1Δ*, *GlmDP/pdc1/5Δ*, and *GlmDP/pdc1/5/6Δ.* Strains were cultivated in YPD medium at 30 °C with shaking at 180 rpm for 48 h. The procedure involved: initial colony growth on solid YPD for 48 h; inoculation into 5 mL YPD in 20 mL test tubes under the same conditions; and after 48 h, the transfer of 1 mL of culture into 50 mL YPD in a 250 mL flask. After incubation, broth was collected for GlcN quantification.

### 2.5. Effect of Gene Modification on Cell Growth and Glucose Consumption

The effect of gene deletion in different combinations on the growth of *S. cerevisiae* was investigated by determining yeast cell concentrations during liquid incubation. The impact of *pdc* gene deletions on cell growth was assessed by measuring the optical density at 600 nm (OD_600_) during incubation using a UV-1780 spectrophotometer (Agilent, USA). *S. cerevisiae GlmDP* and *S. cerevisiae GlmDP/pdc1/5/6Δ* were cultured in 10 mL YPD in 250 mL flasks. After 36 h, 1 mL of each culture (normalized to equal cell density) was transferred to 100 mL fresh YPD. OD_600_ was monitored periodically. Glucose consumption was evaluated by measuring residual glucose in the broth of both strains.

### 2.6. Effect of Gene Modification on Ethanol and GlcN Production

The effect of gene modification on the ethanol and GlcN yields was investigated by determining the ethanol and GlcN contents of *S. cerevisiae GlmDP* and *S. cerevisiae GlmDP/pdc1/5/6Delta*. Cultivation was initiated by inoculating 1 mL of pre-culture into 100 mL YPD in 250 mL flasks. Samples were taken every 6 h over 72 h. After centrifugation at 6000 rpm for 10 min, the supernatant was filtered through a 0.22 μm membrane and analyzed for ethanol and GlcN.

### 2.7. Analytical Methods for GlcN, Ethanol, and Glucose Concentrations

The concentrations of GlcN, ethanol, and glucose were determined by the HPLC method [9]. The ethanol content was determined using the following parameters: Waters 2410 differential refractive index detector (Milford, CT, USA), Shodex SH1011 column (Tokyo, Japan), mobile phase of 10 mmol/L H_2_SO_4_, flow rate of 0.8 mL/min, and column temperature of 30 °C. The GlcN content was determined using the following parameters [21]: Waters 2489 UV detector with an NH_2_ chromatographic column (4.6 mm × 250 mm, 5 μm, Milford, MA, USA), 195 nm detection wavelength, mobile phase of acetonitrile–phosphate buffer, 1.5 mL/min flow rate, and 10 μL injection volume. The glucose content was measured using the following parameters: Waters 2410 differential refractive index detector, Shodex SH1011 column, mobile phase of 10 mmol/L H_2_SO_4_, flow rate of 0.8 mL/min, and column temperature of 30 °C.

### 2.8. Cell Viability Assessment of S. cerevisiae GlmDP/pdc1/5/6Δ

The cell viability of *S. cerevisiae GlmDP/pdc1/5/6Δ* was investigated after culture to the logarithmic growth phase. The detailed process was as follows: The OD_600_ of the initial cell suspension was adjusted to 1.0. Then, 1 mL of this suspension was inoculated into a 250 mL Erlenmeyer flask containing 50 mL of YPD liquid medium. After 48 h of incubation at 30 °C with shaking at 180 rpm, cells were sampled for microscopic examination. The determination of cell viability was performed according to the alkaline methylene blue staining method [22]. After treatment with 0.1% (*w*/*v*) Lü’s alkaline methylene blue stain at 30 °C for 5 min, the cell morphology was observed under a magnification of 100-fold with the oil-immersion objective of a light microscope. The viable (unstained) and dead (blue-stained) cells were counted under a light microscope at 100× magnification with oil immersion. Viability was calculated as the percentage of unstained cells relative to the total.

### 2.9. Genetic Stability Evaluation of S. cerevisiae GlmDP/pdc1/5/6Delta

The contents of GlcN and ethanol were measured during the incubation of 50 sequential subcultures to evaluate the genetic stability of *S. cerevisiae GlmDP/pdc1/5/6Delta*. The detailed steps were as follows: *S. cerevisiae GlmDP/pdc1/5/6Delta* was cultured in a 150 mL Erlenmeyer flask equipped with 10 mL of YPD medium at 30 °C with a shaking speed of 180 rpm. After incubation for 48 h, 1 mL of fermentation broth was sucked out and injected into a 150 mL Erlenmeyer flask equipped with 100 mL of YPD medium. During the next subcultures, 1 mL of fermentation broth was in turn transformed into another 150 mL Erlenmeyer flask equipped with 100 mL of YPD medium with the fermentation conditions of 30 °C and 180 rpm shaking speed.

### 2.10. Transcriptome Analysis: Experimental Processing and Data Analysis

Transcriptome analysis was performed to investigate the effect of *pdc1*, *pdc5*, and *pdc6* deletion on the gene regulation of *S. cerevisiae GlmDP/pdc1/5/6Delta*. The detailed experimental processing and data treatment for transcriptome analysis were as follows. The total RNA of *S. cerevisiae GlmDP* and *S. cerevisiae GlmDP/pdc1/5/6Delta* was extracted using the Total RNA Extractor (Trizol) kit (Waltham, MA, USA) after 24 h of incubation with glucose as the carbon source. The RNA concentration and integrity were assessed with a Qubit 2.0 fluorometer v3.11 and agarose gel electrophoresis 4.6.6, respectively. The sample quality met the requirements for library construction and gene sequencing [23]. The total RNA was used to construct the RNA-Seq library by Sangon Biotech (Shanghai, China) through the following steps: mRNA isolation, fragmentation, double-stranded cDNA synthesis, cDNA fragmentation and modification, magnetic bead purification and size selection, and library amplification. Gene sequencing was performed using the Illumina HiSeq™ platform v2.2.68 [24]. Base calling of the raw image data files obtained from the Illumina HiSeq was conducted using CASAVA v1.8.2 to generate raw sequencing reads. Trimmomatic 0.39 was used to obtain clean data through removing sequences containing N bases, adapter sequences, and low-quality sequences (*q*value < 20) [25]. The sequencing data were aligned to the reference genome of *S. cerevisiae* S288c using HISAT2 2.2.1, and the alignment results were summarized with RSeQC 4.0.0 [26,27]. Based on the sequencing results, gene expression levels were estimated using TPM (Transcripts Per Kilobase of exon model per Million mapped reads). Differential gene expression analysis was performed with DEGseq and functional annotation. An analysis of differentially expressed genes (DEGs) was conducted using the Gene Ontology (GO) and KEGG pathways [28,29]. Genes with a *q*value < 0.05 and |fold change| > 2 were defined as significantly differently expressed.

### 2.11. Data Analysis

Data are presented as the mean ± standard deviation (SD) from three independent replicates. Statistical significance was evaluated using Origin 2024 software (*p* < 0.05).

## 3. Results

### 3.1. Construction of Engineered S. cerevisiae GlmDP for GlcN Production

**Figure 2:**
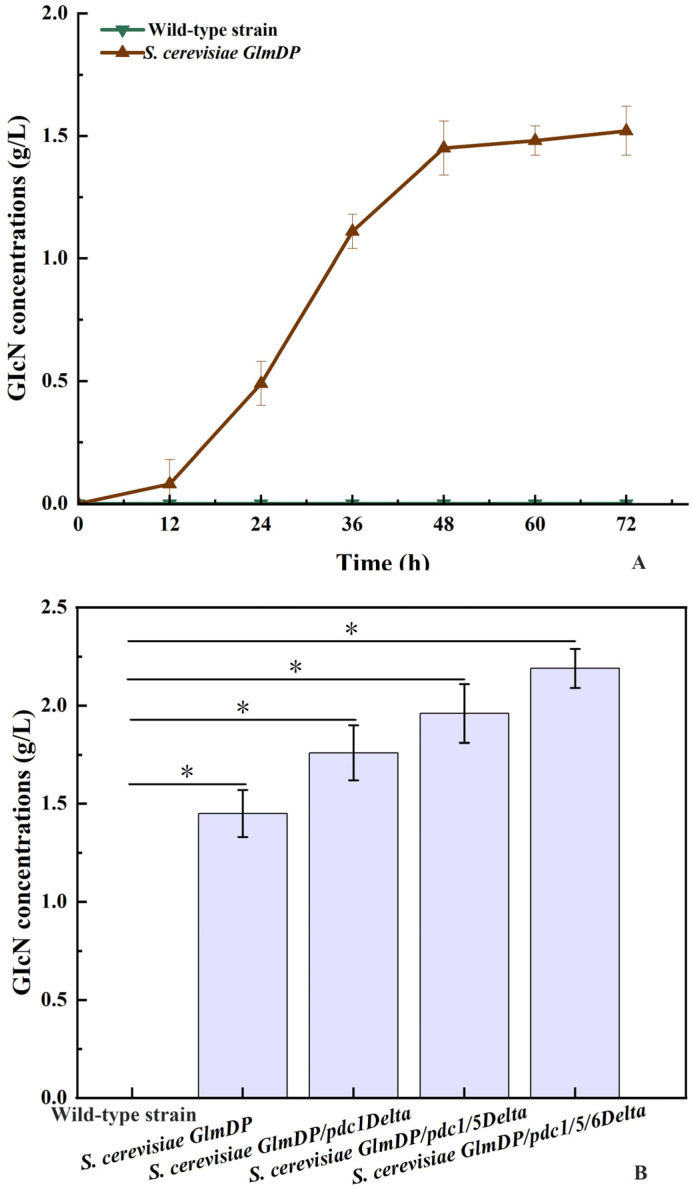
Determination of GlcN concentrations of different *S. cerevisiae* strains. **Note**: (**A**) GlcN concentrations in the wild-type and *S. cerevisiae GlmDP* strains; (**B**) comparison of GlcN concentrations among different engineered *S. cerevisiae* strains. * Data are presented as the mean ± standard deviation (SD) from three independent replicates. Statistical significance was evaluated using Origin 2024 software (*p* < 0.05).

Both the *GlmD* and *GlmP* expression cassettes were integrated into the gene loci *pfk1* and *pdb1*, respectively, of *S. cerevisiae* cells to construct engineered *S. cerevisiae GlmDP* using CRISPR-Cas9 technology. The GlcN concentrations of *S. cerevisiae GlmDP* after culture for 72 h using glucose as a carbon source were investigated in comparison to the wild-type strain (Figure 2A). The GlcN concentrations of *S. cerevisiae GlmDP* gradually increased during the incubation period of 72 h, with no GlcN production from the wild-type strain. This result indicated that *S. cerevisiae GlmDP* enabled de novo GlcN biosynthesis after the integration of the *GlmD* and *GlmP* expression cassettes.

### 3.2. Construction of S. cerevisiae with Sequential pdc Gene Deletions

The engineered yeast was further constructed with *S. cerevisiae GlmDP* as the initial strain by deleting *pdc1*, *pdc5*, and *pdc6* using CRISPR-Cas9 technology. Three engineered strains of *S. cerevisiae GlmDP/pdc1Delta*, *GlmDP/pdc1/5Delta*, and *GlmDP/pdc1/5/6Delta* were constructed by sequentially deleting *pdc1*, *pdc5*, and *pdc6.* The GlcN yields of *S. cerevisiae GlmDP/pdc1Delta* (1.76 ± 0.14 g/L), *S. cerevisiae GlmDP/pdc1/5Delta* (1.96 ± 0.15 g/L), and *S. cerevisiae GlmDP/pdc1/5/6Delta* (2.19 ± 0.10 g/L) were 1.21, 1.35, and 1.51 times that of *S. cerevisiae GlmDP* (1.45 ± 0.12 g/L) after incubation for 48 h, respectively (Figure 2B). The deletion of *pdc1*, *pdc5*, and *pdc6* from *S. cerevisiae GlmDP* resulted in a significant increase in GlcN yield. Thus, progressive *S. cerevisiae pdc1*, *pdc5*, and *pdc6* deletion significantly enhanced the GlcN yield of *S. cerevisiae GlmDP.*

### 3.3. Effect of pdc Deletions on Glucose Consumption and Cell Growth of S. cerevisiae

**Figure 3:**
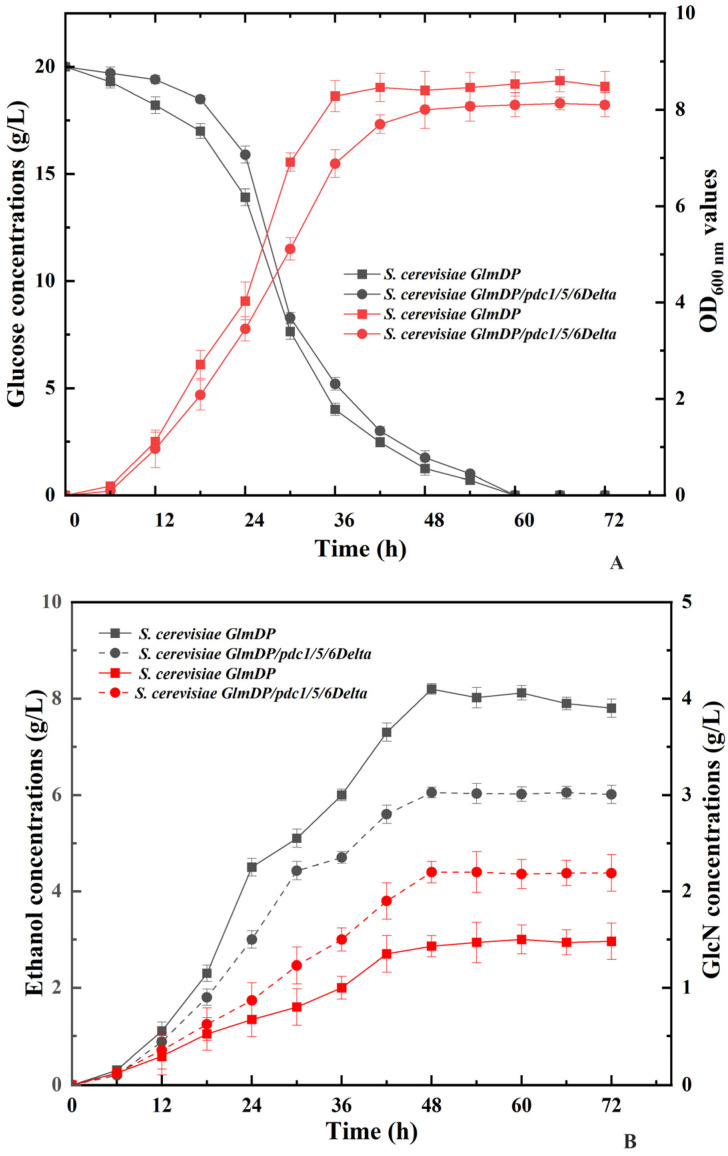
Fermentation characteristics of *S. cerevisiae GlmDP/pdc1/5/6Delta.* **Note**: (**A**) Glucose concentrations (black lines, left Y-axis) and determined OD_600 nm_ values (red lines, right Y-axis) during fermentation; (**B**) determined ethanol yields (black lines, left Y-axis) and GlcN concentrations (red lines, right Y-axis).

The effects of *S. cerevisiae pdc1*, *pdc5*, and *pdc6* deletion on glucose consumption and cell proliferation were investigated by determining glucose concentrations and OD values at the wavelength of 600 nm during incubation (Figure 3A). The glucose contents of *S. cerevisiae GlmDP/pdc1/5/6Delta* during incubation were slightly higher than those of *S. cerevisiae GlmDP.* In addition, the OD_600nm_ values of *S. cerevisiae GlmDP/pdc1/5/6Delta* were slightly lower than those of *S. cerevisiae GlmDP.* Both data sets did not show a significant difference between *S. cerevisiae GlmDP/pdc1/5/6Delta* and *S. cerevisiae GlmDP.* Thus, the deletions of *S. cerevisiae pdc1*, *pdc5*, and *pdc6* did not substantially impair the glucose utilization or cell proliferation of the engineered strains.

### 3.4. Impact of pdc1, pdc5, and pdc6 Deletions on GlcN and Ethanol Yields

The GlcN and ethanol yields of *S. cerevisiae GlmDP/pdc1/5/6Delta* and *S. cerevisiae GlmDP* were determined to investigate the effect of *pdc1*, *pdc5*, and *pdc6* deletion on GlcN biosynthesis and ethanol production (Figure 3B). During incubation for 72 h, the GlcN and ethanol contents dramatically increased within the initial 48 h of incubation. The GlcN and ethanol contents of *S. cerevisiae GlmDP/pdc1/5/6Delta* were 2.20 ± 0.11 g/L and 6.05 ± 0.13 g/L, which were 1.54- and 0.74-fold in comparison to those of *S. cerevisiae GlmDP* (1.43 ± 0.15 g/L, 8.20 ± 0.21 g/L) after incubation for 48 h. Thus, *pdc1*, *pdc5*, and *pdc6* deletions simultaneously increased GlcN output and reduced ethanol formation by the engineered strains.

### 3.5. Cell Viability Determination and Genetic Stability Analysis

**Figure 4:**
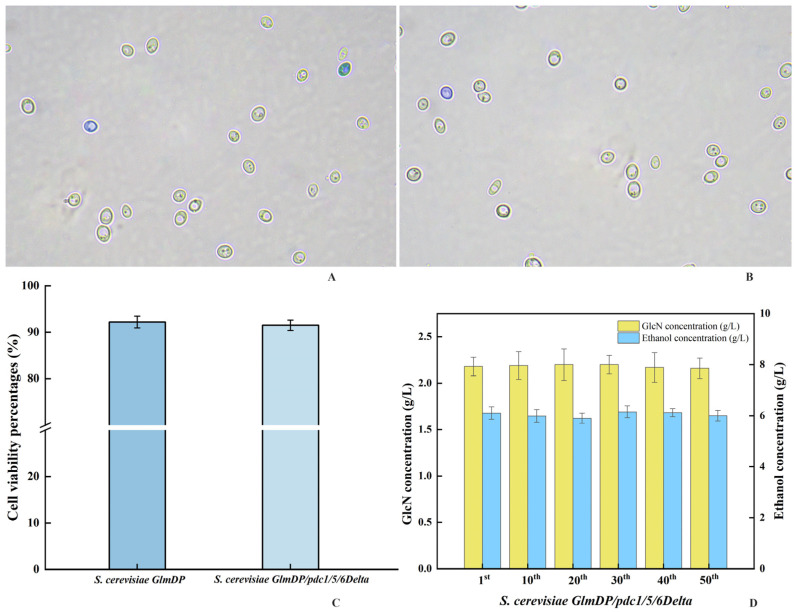
Morphological observation, cell viability, and genetic stability of *S. cerevisiae GlmDP/pdc1/5/6Delta.* **Note**: (**A**) and (**B**) are morphological observations of *S. cerevisiae GlmDP/pdc1/5/6Delta* and *S. cerevisiae GlmDP* under a 100× oil-immersion objective, respectively; (**C**) cell viability of *S. cerevisiae GlmDP* and *S. cerevisiae GlmDP/pdc1/5/6Delta* compared by calculating the proportion of uncolored yeast; (**D**) genetic stability analysis of *S. cerevisiae GlmDP/pdc1/5/6Delta* through calculation of the GlcN and ethanol yields after 50 generations of passage.

The cell viability of *S. cerevisiae GlmDP/pdc1/5/6Delta* was investigated by calculating the numbers of live and dead cells by staining in comparison to those for the control strain *S. cerevisiae GlmDP*. Observation under a microscope showed that a large number of unstained live cells and a very small number of stained dead cells coexisted in the fermentation broth after 72 h of incubation (Figure 4A,B). The percentages of live cells from *S. cerevisiae GlmDP/pdc1/5/6Delta* and *S. cerevisiae GlmDP* were 91.5% and 92.2%, respectively (Figure 4C). No significant difference was shown between *S. cerevisiae GlmDP/pdc1/5/6Delta* and *S. cerevisiae GlmDP*. Further, the genetic stability of *S. cerevisiae GlmDP/pdc1/5/6Delta* was investigated by determining the GlcN content after incubation for 48 h (Figure 4D). The GlcN contents of *S. cerevisiae GlmDP/pdc1/5/6Delta* were 2.18 ± 0.10, 2.19 ± 0.15, 2.20 ± 0.17, 2.20 ± 0.10, 2.17 ± 0.16, and 2.16 ± 0.11 g/L after passage cultivation through the first, 10th, 20th, 30th, 40th, and 50th generations. In addition, the ethanol contents of *S. cerevisiae GlmDP/pdc1/5/6Delta* were 6.10 ± 0.24, 5.99 ± 0.25, 5.90 ± 0.20, 6.15 ± 0.23, 6.12 ± 0.16, and 6.10 ± 0.21 g/L through the first, 10th, 20th, 30th, 40th, and 50th generations. Both GlcN and ethanol contents had no significant differences through passage cultivation for six generations. The lack of a significant observed decline indicated the robust hereditary stability of the yeast. Thus, *S. cerevisiae GlmDP/pdc1/5/6Delta* had high genetic stability for GlcN and ethanol production through multiple generations.

### 3.6. Differentially Expressed Gene (DEG) Analysis

**Figure 5:**
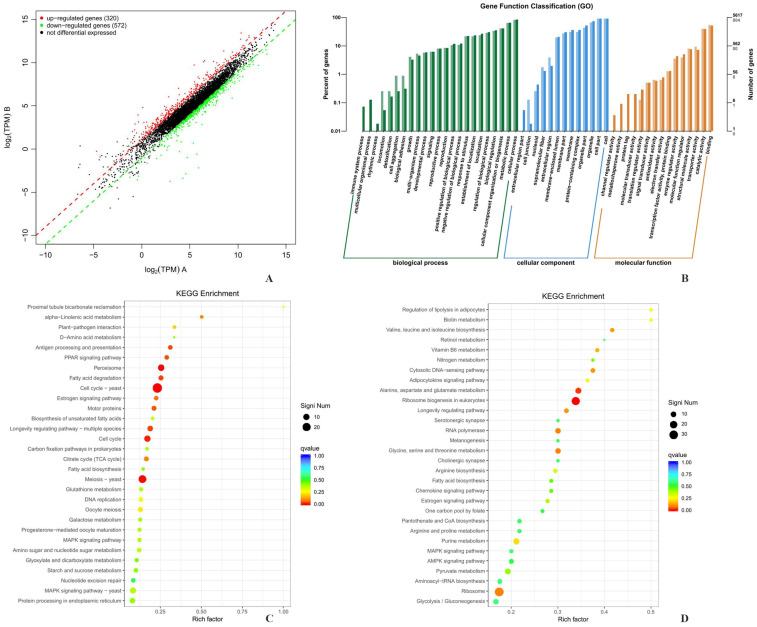
Comparative transcriptomics analysis between *S. cerevisiae GlmDP/pdc1/5/6Delta* and *S. cerevisiae GlmDP*. **Note**: (**A**) Scatterplot from DEG analysis; (**B**) GO functional classification of DEGs of *S. cerevisiae GlmDP/pdc1/5/6Delta*; (**C**) and (**D**) represent upregulated and downregulated metabolic pathways based on KEGG enrichment in *S. cerevisiae GlmDP/pdc1/5/6Delta*, respectively.

To investigate the DEGs between *S. cerevisiae GlmDP/pdc1/5/6Delta* and strain *S. cerevisiae GlmDP*, based on the significance level *q*Value < 0.05, scatter plots of significant DEGs with a fold change factor |Fold Change| ≥ 2 were generated (Figure 5A) (Supplementary Materials). A total of 892 DEGs were screened from the library, with 320 significantly upregulated genes (accounting for 35.87%) and 572 significantly downregulated genes (accounting for 64.13%). The results showed that the gene expression changes for *S. cerevisiae GlmDP/pdc1/5/6Delta* were mainly downregulated when compared to *S. cerevisiae GlmDP*.

### 3.7. Gene Ontology (GO) Annotation Analysis

According to GO, the 892 DEGs obtained were classified into three categories based on Biological Process (BP), Cellular Component (CC), and Molecular Function (MF) (Figure 5B). In terms of biological processes, significantly DEGs were mainly enriched in metabolic processes, cellular processes, and biological regulation. DEGs were also involved in processes such as growth and biological adhesion, indicating that the experimental treatments had a systematic impact on yeast core metabolism and basic cellular activities. In terms of cell composition, the significantly DEGs of *S. cerevisiae GlmDP/pdc1/5/6Delta* were mainly enriched in cells, cell parts, and membranes, mainly causing changes in cell membrane structure and the proportion of cell components. There was also a small number of differentially expressed genes in components such as cell junctions, reflecting potential changes in intercellular connections. In terms of molecular function, significantly DEGs were mainly enriched in binding, catalytic activity, transporter activity, and signal transducer activity. Therefore, the gene deletion of *pdc1/5/6* in *S. cerevisiae GlmDP/pdc1/5/6Delta* had an impact on the membrane structure and substance transport, metabolic enzyme activity regulation, cell signaling, and other biological processes.

### 3.8. Kyoto Encyclopedia of Genes and Genomes (KEGG) Enrichment Analysis

The KEGG database was used to conduct enrichment analysis on the 30 pathways with the highest enrichment degree for the DEGs. The metabolic pathways upregulated by *S. cerevisiae GlmDP/pdc1/5/6Delta* with *pdc1Delta*, *pdc5Delta*, and *pdc6Delta* mainly included proximal tubular bicarbonate reabsorption, alpha linolenic acid metabolism, *D*-amino acid metabolism, and the PPAR signaling pathway (Figure 5C). Among them, the upregulation of the alpha linolenic acid metabolic pathway enhanced the decomposition and transformation of polyunsaturated fatty acids. Upregulation of the *D*-amino acid metabolic pathway facilitated the metabolic recovery of specific amino acids and provided potential amino donors for the GlcN synthesis. The activation of the PPAR signaling pathway was involved in the regulation of lipid metabolism and oxidative stress, maintaining the metabolic homeostasis of cells in a high-yield state. The enrichment of the proximal tubular bicarbonate reabsorption pathway also reflected the optimization of cellular regulation of acid–base balance and ion transport, which together created a favorable metabolic environment for high GlcN yield.

The downregulated metabolic pathways were mainly enriched in core metabolic pathways such as glycolysis/gluconeogenesis, pyruvate metabolism, and fatty acid biosynthesis, as well as biosynthesis pathways such as ribosome biogenesis, ribosome, and valine/leucine/isoleucine biosynthesis in eukaryotes (Figure 5D). The downregulation of the glycolysis/gluconeogenesis and pyruvate metabolism pathways reduced the diversion of carbon sources to competing pathways such as ethanol fermentation. The downregulation of the ribosomal biogenesis and amino acid biosynthesis pathways reduced the consumption of non-essential metabolic resources such as via protein synthesis, allowing more precursor substances (such as glucose-6-phosphate) to be directed towards the biosynthesis of GlcN, thereby optimizing carbon source allocation to support high GlcN production.

## 4. Discussion

A fundamental challenge for native *S. cerevisiae* producing GlcN is the lack of efficient pathways for GlcN metabolism. In addition, ethanol accumulation during fermentation directly inhibits yeast cell growth and reduces GlcN metabolic output [30]. Since *S. cerevisiae* lacks key enzymes like glucosamine-6-phosphate deaminase [31], the introduction of heterologous genes is essential for both catabolism and synthesis of GlcN. In addition, a key hurdle is the conflict between key by-products like ethanol and product synthesis [32]. The modification of metabolic flux to inhibit ethanol formation is also an effective approach to decrease carbon flow loss [33]. In this study, engineered *S. cerevisiae* was constructed through the integration of *GlmD* and *GlmP* expression cassettes and the deletion of *pdc1*, *pdc5*, and *pdc6.* To summarize, this study provided an effective solution to GlcN production by *S. cerevisiae* with an ethanol production decrease by deleting *pdc1*, *pdc5*, and *pdc6.* Admittedly, more approaches including fermentation environment optimization, cell growth and production formation balance, and stress tolerance enhancement are further needed for GlcN accumulation in *S. cerevisiae GlmDP/pdc1/5/6Delta*.

In this study, the part of the carbon being shifted towards glycerol production was likely one way to regenerate NAD^+^. Another way to regenerate NAD^+^ could be through aerobic respiration via the electron transport chain (ETC). The ETC is located in the inner mitochondrial membrane with the final common pathway for oxidative phosphorylation. This study indicated that the upregulated pathways relevant to ETC genes were the citrate cycle (TCA cycle), fatty acid degradation (beta-oxidation), fatty acid biosynthesis, and peroxisome in the knockout mutant.

PDC1, PDC5, and PDC6 isozymes catalyze the decarboxylation of pyruvate to acetaldehyde in *S. cerevisiae* with varying efficiency and intensity [34]. The major isozyme PDC1 is strongly expressed during glucose fermentation and carries the bulk of the metabolic flux toward ethanol [35]. A minor isozyme, PDC5 is active during glucose fermentation in yeast. The expression of *pdc5* and *pdc6* is very weak under normal fermentation conditions. The primary role of PDC6 appears to be in amino acid catabolism. The *pdc6* gene is specifically induced during ethanol production under sulfur limitations. In addition, differences in the gene deletion combination lead to varying effects on ethanol synthesis. Single *pdc1* deletion causes a moderate decrease in ethanol yield. The yeast still remains viable because *pdc5* expression is upregulated to partially compensate. Carbon is partially redirected toward other pathways such as glycerol and acetate formation. The deletion of *pdc5* alone has little effect on the overall ethanol yield and the cell growth. The combination of *pdc1* and *pdc5* deletion synthetically impacts fermentation and metabolism and causes a 30% decrease in ethanol production by *S. cerevisiae* [36]. Triple deletion of *pdc1*, *pdc5*, and *pdc6* severely damages pyruvate decarboxylase activity and dramatically inhibits the direct conversion of pyruvate to acetyl-CoA via the pyruvate dehydrogenase complex [37,38].

In this study, the multiple-deletion combinations of *pdc1*, *pdc1/pdc5*, and *pdc1/pdc5/pdc6* caused dramatic decreases in ethanol yield. The deletion of *pdc1*, *pdc5*, and *pdc6* in *S. cerevisiae GlmDP/pdc1/5/6Delta* resulted in a GlcN yield increase and conversion efficiency enhancement. In addition, comparative transcriptomics indicated that the triple-gene deletion affected the membrane structure and substance transport, metabolic enzyme activity regulation, and so on. The gene deletion also had a significant effect on the downregulated metabolic pathways, which were mainly enriched in core metabolic pathways. Therefore, this study has important reference value for improving the conversion rate of glucose and other products by inhibiting ethanol formation during the production of high-value by-products using yeast as a chassis microorganism.

Table 2 provides a comprehensive overview of the metabolic engineering strategies and the corresponding yields of glucosamine (GlcN)/N-acetylglucosamine (GlcNAc) in various microbial hosts. The initial engineering efforts were predominantly in *E. coli* and *B. subtilis* through the overexpression of key limiting enzymes. The yields of GlcN were 17 g/L and 35.3 g/L in *E. coli* and *B. subtilis* after overexpression of *GlmS* with knockout of degradation genes [39] and *Pyrococcus furiosus Dac* enzyme overexpression by whole-cell catalysis [40], respectively. In addition, the eukaryotic production systems of *Aspergillus* sp. and *S. cerevisiae* are effective platforms for GlcN production. The yields of GlcN were 14.37 g/L and 7.48 g/L in *Aspergillus* sp. with two-stage DO control (0–12 h 30%, 12–60 h 50%) [41] and methanol stimulation control [13], respectively. The yeast *S. cerevisiae* offers a robust and industrial-grade eukaryotic host. The yield of GlcN was 1.95 g/L in *S. cerevisiae* after the deletion of multiple genes in glycolysis (*PFK1*), pyruvate metabolism (*PDB1*), and competing pathways (*GNA1*, *PCM1*, *ISR1*) and the integration of *E. coli* glucosamine synthase *GlmD* and *GlmP* and an ammonium transporter (AMT1) [13]. In this study, we focused on integrating *glmD* and *glmP* genes while deleting the pyruvate decarboxylase genes (*pdc1*, *pdc5*, *pdc6*) to block ethanol production, thereby redirecting carbon flux toward GlcN synthesis. This targeted approach resulted in a GlcN titer of 2.2 g/L, representing a clear improvement over the previous *S. cerevisiae* engineering effort [13]. This result demonstrated that major fermentative pathway elimination in *S. cerevisiae* is a highly effective strategy for enhancing GlcN production, offering a promising platform for further metabolic and process optimization.

## 5. Conclusions

This study constructed an engineered *S. cerevisiae* strain with enhanced glucosamine (GlcN) production by redirecting carbon flux away from ethanol formation. The integration of *GlmD* and *GlmP* expression cassettes enabled de novo GlcN biosynthesis. Sequential CRISPR-Cas9-mediated knockout of *pdc1*, *pdc5*, and *pdc6* in *S. cerevisiae GlmDP* progressively reduced ethanol synthesis while increasing the GlcN yield. The triple-deletion strain of *S. cerevisiae GlmDP/pdc1/5/*6Δ achieved a GlcN titer of 2.20 ± 0.11 g/L, a 1.54-fold increase over that from *S. cerevisiae GlmDP*, alongside a 26% reduction in ethanol, without significantly compromising cell growth or glucose consumption. The engineered strain exhibited high genetic stability over 50 generations. Comparative transcriptomics revealed that the genetic modifications resulted in widespread transcriptional reprogramming, primarily downregulating genes in the glycolysis and ethanol formation pathways while upregulating pathways conducive to GlcN synthesis. These findings demonstrate that targeted disruption of competitive ethanol formation is an effective strategy to enhance the GlcN production of *S. cerevisiae*, providing valuable insights into carbon flux redistribution for developing efficient yeast cell factories.

## Figures and Tables

**Table 1:** Primers and sequences used in this study.

Primer	Sequence (5′→3′)	Description
*pfk1*-gRNA-F	TGTAACCAGAGTGACCACCTTGGGTTTTAGAGCTAGAAATAGCAAG	*pfk1*-gRNA
*pfk1*-gRNA-R	CCAAGGTGGTCACTCTGGTTACA GATCATTTATCTTTCACTGCGGA	
*pdb1*-gRNA-F	TTCCATTAGAGCATTCAAAGCGGGTTTTAGAGCTAGAAATAGCAAG	*pdb1*-gRNA
*pdb1*-gRNA-R	CCGCTTTGAATGCTCTAATGGAAGATCATTTATCTTTCACTGCGGA	
*pdc1*-gRNA-F	CATCCATTCTTGCTTCAATGGGGGTTTTAGAGCTAGAAATAGCAAG	*pdc1*-gRNA
*pdc1*-gRNA-R	CCCCATTGAAGCAAGAATGGATG GATCATTTATCTTTCACTGCGGA	
*pdc5*-gRNA-F	GGAAAAGTTGACTCAAGACAAGGGTTTTAGAGCTAGAAATAGCAAG	*pdc5*-gRNA
*pdc5*-gRNA-R	CCTTGTCTTGAGTCAACTTTTCCGATCATTTATCTTTCACTGCGGA	
*pdc6*-gRNA-F	GTTGGGGTCAATCTCCTCAGCGGGTTTTAGAGCTAGAAATAGCAAG	*pdc6*-gRNA
*pdc6*-gRNA-R	CCGCTGAGGAGATTGACCCCAACGATCATTTATCTTTCACTGCGGA	
pEGFP-*GlmD*-F	CGGGGTCATTAGTTCATAGCCC	2977 bp
pEGFP-*GlmD*-R	GCCCGCTCCTTTCGCTTTCTTC	
pEGFP-*GlmP*-F	ACGGGGTCATTAGTTCATAGCC	2622 bp
pEGFP-*GlmP*-R	CCGCTCCTTTCGCTTTCTTCCC	
pEGFP-*Pepc1*-F	CCCCACACACCATAGCTTCA	3360 bp
pEGFP-*Pepc1*- R	GAAATGTGGATGGTGTGTTACT	
*Xyna*-F	CCCCACACACCATAGCTTCA	1129 bp
*Xyna*-R	GCGGATGTGGGGGGAGGGC	
*Cel*-F	CCCCACACACCATAGCTTCA	880 bp
*Cel*-R	CCGCCTGCGCCGCTCCGGTG	

**Note**: Pairs of *pfk1*-gRNA-F/*pfk1*-gRNA-R, *pdb1*-gRNA-F/*pdb1*-gRNA-R, *pdc1*-gRNA-F/*pdc1*-gRNA-R, *pdc5*-gRNA-F/*pdc5*-gRNA-R, and *pdc6*-gRNA-F/*pdc6*-gRNA-R primers were used to amplify the gRNA vectors of *pfk1*-gRNA, *pdb1*-gRNA, *pdc1*-gRNA, *pdc5*-gRNA, and *pdc6*-gRNA, respectively. In addition, pairs of pEGFP-*GlmD*-F/R, pEGFP-*GlmP*-F/R, pEGFP-*Pepc1*-F/R, *Xyna*-F/R and *Cel*-F/R primers were used to amplify the *GlmD* expression cassette, the *GlmP* expression cassette, *Pepc1*, *Xyna*, and *Cel*, respectively.

**Table 2:** GlcN/GlcNAc yields and engineering strategies in different engineered microorganisms.

Host Strains	Engineering Strategies	Products and Yields
*E. coli*	Overexpression of *GlmS* mutant + knockout of degradation genes	GlcN, 17 g/L [39]
*E. coli*	Overexpression of *GlmS* + *GNA1* + knockout of degradation genes	GlcNAc, 110 g/L [39]
*B. subtilis*	*GlmS* and *GNA1* overexpression + *ldh*, *pta* knockout	GlcNAc, 31.65 g/L [42]
*B. subtilis*	Expression of *Pyrococcus furiosus Dac* enzyme, whole-cell catalysis	GlcN, 35.3 g/L [40]
*Aspergillus* sp.	Two-stage DO control (0–12 h 30%, 12–60 h 50%)	GlcN, 14.37 g/L [41]
*Aspergillus* sp.	Methanol stimulation + optimization of culture conditions	GlcN, 7.48 g/L [13]
*S. cerevisiae*	Deletion of *PFK1*, *PDB1*, *GNA1*, *PCM1*, and *ISR1* + Integration of *GlmD*, *GlmP*, and *AMT1*	GlcN, 1.95 g/L [13]
*S. cerevisiae*	Integration of *GlmD* and *GlmP* + deletion of *pdc1*, *pdc5*, *pdc6*	GlcN, 2.2 g/L, this study

## Data Availability

The original contributions presented in this study are included in the article/Supplementary Materials. Further inquiries can be directed to the corresponding author.

## Supplementary Materials

The following supporting information can be downloaded at https://www.mdpi.com/article/10.3390/foods15071163/s1. DEG data for *S. cerevisiae GlmDP/pdc1/5/6Delta* and strain *S. cerevisiae GlmDP*.
