# Supplementary material for: Glucosamine Yield Improvement in Engineered Saccharomyces cerevisiae with Ethanol Yield Reduction by Carbon Flux Redistribution
Source: Foods. 2026 Mar 30;15(7):1163. doi: 10.3390/foods15071163 (PMC13073038; doi:10.3390/foods15071163)
Supplement: Supplementary file 1 [file foods-15-01163-s001.zip › foods-4162690-supplementary.pdf]

Gene id	MeanTPM (B)	MeanTPM (A)	log2FoldChange	pValue	qValue	result
YMR303C	11019.1543	1271.213379	3.115735385	0	0	up
YGL055W	2017.383301	8081.385254	-2.00211739	0	0	down
YCR010C	8796.649414	2242.966553	1.971546008	0	0	up
YAL005C	4018.965332	1442.975342	1.477777486	0	0	up
YGR142W	2612.081787	364.560059	2.840971656	0	0	up
YOL086C	824.002563	4463.327148	-2.437398826	0	0	down
YLL026W	2933.432373	1255.223145	1.224645873	0	0	up
YBR072W	20290.58203	11324.98633	0.84130094	0	0	-
YIL057C	6721.343262	1230.372681	2.449654209	0	0	up
YGL062W	332.750305	1415.151611	-2.08844473	0	0	down
YAL062W	2362.137695	6035.290039	-1.353330039	0	0	down
YBR067C	5985.668457	1832.27417	1.707876976	0	0	up
YLR110C	14086.67871	6004.692871	1.230169138	0	0	up
YDR134C	7741.845215	2253.669922	1.780401233	0	0	up
YPL240C	2192.482666	913.339355	1.263342532	0	0	up
YGR192C	9106.632812	5370.564453	0.761843989	0	0	-
YEL021W	2851.885498	7575.366699	-1.409399667	0	0	down
YDR171W	3475.760742	1344.809692	1.369926749	0	0	up
YDR256C	1132.487671	175.486954	2.690059657	0	0	up
YBR296C	1937.422607	4160.507812	-1.102620947	0	0	down
YJL052W	890.589111	3056.914795	-1.779244463	0	0	down

YBR101C	2386.696045	678.67041	1.814231827	0	0	up
YOR388C	842.634705	43.035118	4.291321007	0	0	up
YDR258C	1030.160645	370.008881	1.477237527	0	0	up
YFL014W	11657.67773	5564.650391	1.06691747	0	0	up
YLR267W	235.886124	1111.242188	-2.236010817	0	0	down
YPL275W	1199.397705	91.762962	3.708254347	0	0	up
YKL096W-A	6425.441406	1967.861938	1.707166559	0	0	up
YHR096C	751.371033	1992.266724	-1.406813405	0	0	down
YLL024C	1188.867065	450.69873	1.399352117	0	0	up
YER103W	873.438782	264.223999	1.72494508	0	0	up
YAL054C	1752.635254	921.005859	0.928243545	0	0	-
YBL015W	1653.860107	3353.168945	-1.019687965	0	0	down
YER056C	330.171112	1159.01355	-1.811611629	0	0	down
YIL046W	192.747635	797.130859	-2.048103422	0	0	down
YNL007C	1820.326538	723.135986	1.331858393	0	0	up
YCL018W	884.179016	2413.973633	-1.448999517	0	0	down
YLR327C	22064.48438	14550.02832	0.600704072	0	0	-
YPL265W	584.878723	1502.656372	-1.361305719	0	0	down
RDN25-1	1001.708435	1847.027954	-0.882743053	0	0	-
YDR040C	293.887085	776.311035	-1.401372835	0	0	down
YOR298C-A	3892.282471	1578.62207	1.301950589	0	0	up
YER020W	1201.917358	2660.281738	-1.146241341	0	0	down

YNR002C	755.609436	2298.458008	-1.604953686	0	0	down
YJL159W	5169.743164	3709.570801	0.478840332	0	0	-
YLR249W	114.385498	430.687195	-1.912736274	0	0	down
RDN25-2	989.853394	1771.851196	-0.839970678	0	0	-
YOR374W	1886.988281	1070.137207	0.81828968	0	0	-
YOR375C	1394.034546	2850.354004	-1.031874795	0	0	down
YKL217W	1381.304932	2605.206543	-0.915365916	0	0	-
YMR096W	59.344975	670.344666	-3.497705285	0	0	down
YPL276W	1091.411133	85.936752	3.666775604	0	0	up
YDR119W-A	7757.226074	3314.235352	1.226864792	0	0	up
ETS1-2	1193.608276	3058.966797	-1.357715004	0	0	down
YML054C	866.342957	369.83667	1.228049976	0	0	up
YGR211W	665.230591	199.439117	1.737906113	0	0	up
YBR026C	814.165588	242.097672	1.749733038	0	0	up
YCR005C	1535.767822	2946.355713	-0.939971492	0	0	-
ETS1-1	1097.162109	2832.855713	-1.368480418	0	0	down
YNL209W	152.477386	594.512268	-1.963111289	0	0	down
YPL061W	517.490112	1302.15918	-1.331302617	0	0	down
YOR247W	1352.397705	405.420258	1.738029389	0	0	up
YBR286W	1040.470215	518.323608	1.005310656	0	0	up
YPR035W	4316.926758	6845.599121	-0.665172195	0	0	-
YJL217W	1015.818115	220.351242	2.204765173	0	0	up

YOR303W	219.852066	812.876648	-1.886503354	0	0	down
YER024W	537.343628	1077.661987	-1.003987859	0	0	down
YHR139C	608.522095	1612.117065	-1.405574958	0	0	down
YLR259C	1018.523621	532.407593	0.935876387	0	0	-
YIL053W	873.03418	2227.320557	-1.351199164	0	0	down
YGR043C	1188.66272	515.647644	1.204881937	0	0	up
YGR032W	167.482971	371.923035	-1.150989688	0	0	down
YEL071W	638.615906	1415.968994	-1.148769283	0	0	down
YDR038C	146.833221	404.102509	-1.460542894	0	0	down
YLR304C	771.128052	434.78772	0.826659257	0	0	-
YLR058C	211.56073	687.697449	-1.700702135	0	0	down
YMR186W	742.82312	399.473389	0.894919318	0	0	-
YMR251W-A	16479.09766	11124.17188	0.566939307	0	0	-
YOR027W	943.750488	525.308472	0.845240633	0	0	-
YLR286C	564.710144	239.533691	1.237281958	0	0	up
YKL141W	1806.973511	830.90387	1.120821876	0	0	up
YLR174W	601.431213	225.075226	1.417992474	0	0	up
YDR039C	116.54657	325.661743	-1.482467703	0	0	down
YMR016C	40.547871	214.747803	-2.4049453	0	0	down
YBR046C	817.078308	338.769135	1.270171914	0	0	up
YHL032C	326.888153	123.342934	1.406122023	0	0	up
YNL142W	473.434174	1046.27063	-1.144020316	0	0	down

YKL164C	737.52124	299.265717	1.301257581	0	0	up
YDR032C	1162.545288	444.819611	1.385994619	0	0	up
YPR006C	217.850372	44.756161	2.283179379	0	0	up
YER150W	6679.766602	4805.352051	0.475155564	0	0	-
YOR285W	1944.47522	837.379944	1.21542658	0	0	up
YLR216C	671.853394	277.214081	1.277145914	0	0	up
YBR068C	401.641968	865.523804	-1.107663472	0	0	down
YMR058W	33.42556	203.061203	-2.602890988	0	0	down
YIL160C	287.945618	62.210896	2.210557177	0	0	up
YJL079C	745.504578	297.533569	1.325164763	0	0	up
YNL077W	1048.796509	652.590515	0.684484865	0	0	-
YNL192W	324.190857	619.450256	-0.93414503	0	0	-
YGR279C	373.023468	102.701172	1.860813752	0	0	up
YFL016C	495.709045	217.48584	1.18857211	0	0	up
YGL191W	5315.455566	3583.092773	0.568987946	0	0	-
YIL155C	337.812103	138.913574	1.282033438	0	0	up
YDR492W	121.477196	510.046234	-2.069942516	0	0	down
YIL136W	2393.325928	1763.312378	0.440728803	0	0	-
YBL075C	861.78949	556.292847	0.631490949	0	0	-
YNL117W	923.204834	580.87085	0.668433346	0	0	-
YJL034W	499.767609	269.737732	0.889700055	0	0	-
YEL065W	45.684799	216.16066	-2.242317872	0	0	down

YGR121C	259.496429	645.798706	-1.315369865	0	0 down
YER158C	50.474743	235.49971	-2.222091718	0	0 down
YBR230C	2137.037354	1084.244019	0.978922641	0	0 -
YKR042W	767.164734	1499.450684	-0.966825766	0	0 -
YLR284C	396.620636	101.462692	1.966810399	0	0 up
YAL039C	370.386749	977.048157	-1.399397186	0	0 down
YJL144W	1588.608154	597.607849	1.410492312	0	0 up
YGR161C	935.137329	1866.468872	-0.997061297	0	0 -
YOR209C	241.705093	622.679993	-1.365243053	0	0 down
YLR038C	3256.869141	1673.537964	0.960584476	0	0 -
YIL051C	1419.337646	645.383911	1.136988315	0	0 up
YLL019C	209.685608	466.151642	-1.152571505	0	0 down
YMR085W	141.617371	434.463593	-1.617237048	0	0 down
YKR066C	501.734985	213.84259	1.230376322	0	0 up
YGR180C	1211.777954	760.482239	0.672138904	0	0 -
YMR108W	356.538635	697.717834	-0.968585293	0	0 -
YMR083W	595.606689	1192.619385	-1.00170183	0	0 down
YER015W	171.864502	56.574203	1.603055332	0	0 up
YMR088C	82.244858	281.693695	-1.776129884	0	0 down
YNL112W	48.589062	213.891037	-2.138172541	0	0 down
YPL223C	5263.993652	7987.374512	-0.601563611	0	0 -
YML028W	1530.472168	861.828674	0.828503805	0	0 -

YHR008C	2513.529541	1764.328247	0.510595651	0	0	-
YCL064C	830.079407	467.265961	0.829005409	0	0	-
YNL160W	2228.709473	1682.086548	0.405956626	0	0	-
YML123C	136.280502	367.3526	-1.430586319	0	0	down
YML131W	291.273346	85.320343	1.771412018	0	0	up
YMR145C	429.788513	221.414948	0.956874301	0	0	-
YPR030W	252.815643	470.964966	-0.897534012	0	0	-
YBR162C	363.553131	152.461838	1.253718047	0	0	up
YNL135C	1793.360352	858.753906	1.062348747	0	0	up
YPR010C-A	4495.452148	2655.572021	0.759443571	0	0	-
YDL085W	343.253632	159.780945	1.103179619	0	0	up
YMR120C	67.430336	232.62532	-1.786538443	0	0	down
YDL229W	64.33255	223.076248	-1.793916131	0	0	down
YLR414C	729.992737	1464.281738	-1.00423915	0	0	down
YKR009C	232.495651	116.649513	0.995023446	0	0	-
YBR118W	4301.025879	3827.646973	0.168223037	0	0	-
YER065C	563.465942	1003.769409	-0.833027568	0	0	-
YDR186C	118.524605	281.561249	-1.248262206	0	0	down
YNL055C	2671.802246	2051.278809	0.381289635	0	0	-
YGR122W	106.563324	351.311096	-1.721038154	0	0	down
YDL210W	118.443924	325.277893	-1.457468583	0	0	down
YMR297W	858.260193	585.02655	0.552912987	0	0	-

YPR192W	95.014267	369.62793	-1.95985771	0	0	down
YJL048C	1097.210815	742.082642	0.564188981	0	0	-
YMR110C	459.799133	254.994843	0.850535675	0	0	-
YAL053W	193.634659	406.229431	-1.068957559	0	0	down
YJR094C	107.948143	360.939636	-1.741419154	0	0	down
YKL016C	2200.64624	1456.592896	0.595329533	0	0	-
YMR020W	249.664627	543.68396	-1.122776834	0	0	down
YNL009W	272.827362	101.382362	1.428181659	0	0	up
YDL078C	554.601074	281.60675	0.977768495	0	0	-
YDR007W	384.246063	112.147087	1.776638331	0	0	up
YMR256C	3205.05249	1653.964722	0.954419521	0	0	-
YPR002W	393.477631	749.96405	-0.930539819	0	0	-
YJR078W	34.206135	173.713715	-2.344384656	0	0	down
YER004W	1019.816772	581.818115	0.809669849	0	0	-
YIL056W	91.691689	250.822586	-1.451804387	0	0	down
YCR021C	893.494507	560.986389	0.671493092	0	0	-
YKR097W	2065.665039	2919.190186	-0.498961875	0	0	-
YJR009C	405.788269	846.935669	-1.061525232	0	0	down
YLR142W	975.819153	1559.810669	-0.676685219	0	0	-
YOL032W	395.166595	138.934998	1.508050933	0	0	up
YPL250C	472.303741	111.516418	2.082458836	0	0	up
YOL084W	119.528397	256.552185	-1.101898905	0	0	down

YEL060C	1227.079224	1807.652344	-0.558888842	0	0	-
YJL133C-A	2451.810791	4692.091309	-0.93638344	0	0	-
YDL222C	1621.966675	2537.866455	-0.645871977	0	0	-
YNL015W	4084.332275	2661.19873	0.618023989	0	0	-
YOR040W	432.851044	189.143646	1.194388327	0	0	up
YGL056C	138.57872	338.108246	-1.286779466	0	0	down
YLR023C	41.361862	161.451035	-1.964723651	0	0	down
YBR105C	123.678848	357.052582	-1.529537764	0	0	down
YJL158C	492.157501	207.241257	1.247808844	0	0	up
YGR088W	1091.840454	852.966736	0.356200671	0	0	-
YHR007C	133.760666	325.429108	-1.282689363	0	0	down
YEL011W	659.54834	487.055023	0.437393638	0	0	-
YDR037W	75.260292	215.690948	-1.51900484	0	0	down
YGL234W	35.089893	121.210258	-1.788384345	0	0	down
YPR065W	392.791901	776.140991	-0.982553568	0	0	-
YOL147C	347.485077	119.223557	1.5432817	0	0	up
YJR104C	1921.808716	1267.011353	0.601035295	0	0	-
YBR149W	862.532349	569.471558	0.598954777	0	0	-
YJR045C	1024.950195	821.787415	0.318716665	0	0	-
YGR236C	4075.027588	2914.70166	0.483461605	0	0	-
YKL182W	44.092842	97.898636	-1.15074429	0	0	down
YPL231W	81.886559	157.196045	-0.94086635	0	0	-

YOR180C	130.56427	14.087276	3.21229557	0	0	up
YMR195W	376.898254	1025.698608	-1.444359854	0	0	down
YGR230W	323.406189	57.448982	2.492994054	0	0	up
YOR007C	516.277527	291.232178	0.825977034	0	0	-
YHR206W	75.276726	204.449768	-1.441470638	0	0	down
YLL045C	226.314346	569.597656	-1.33161517	0	0	down
YHR137W	270.261597	137.010818	0.980066717	0	0	-
YER124C	161.370392	64.845444	1.315298779	0	0	up
YNR016C	20.954483	56.729321	-1.436835669	0	0	down
YHR097C	1669.533325	2470.296387	-0.565239256	0	0	-
YLR178C	960.02832	583.543762	0.718236112	0	0	-
YMR104C	174.93219	82.424149	1.085656798	0	0	up
YJL094C	131.530075	268.06839	-1.027208394	0	0	down
YHL033C	89.059265	321.064758	-1.850026704	0	0	down
YBL045C	1040.609131	793.140747	0.391779464	0	0	-
YNL202W	381.302094	174.53508	1.127417419	0	0	up
YLR120C	128.076431	296.644775	-1.211731361	0	0	down
YLR377C	466.619324	257.643402	0.856870408	0	0	-
YPL131W	290.777496	638.581116	-1.134954271	0	0	down
YNL278W	49.900146	127.745674	-1.356158495	0	0	down
YER165W	290.010559	535.489624	-0.884753192	0	0	-
YJR148W	576.575439	358.621124	0.685048912	0	0	-

YOR184W	229.465302	494.371979	-1.107320957	0	0	down
YKR076W	397.505798	212.806549	0.901433354	0	0	-
YCR011C	141.223602	75.905792	0.895699339	0	0	-
YBL064C	896.646545	574.585876	0.642016862	0	0	-
YKR013W	181.146591	53.615326	1.756440295	0	0	up
YMR011W	197.415466	403.391296	-1.030944937	0	0	down
YDR077W	9714.149414	9489.433594	0.0337656985938	0	0	-
YGR061C	24.846121	73.939629	-1.57332717	0	0	down
YPR154W	1858.704712	1384.265015	0.42517742	0	0	-
YGR055W	20.173777	98.471611	-2.287226646	0	0	down
YKL096W	398.931183	173.264587	1.203163081	0	0	up
YLR441C	319.295776	700.656067	-1.133812971	0	0	down
YJL045W	97.236557	30.439695	1.675544907	0	0	up
YER025W	67.83815	195.996262	-1.530657408	0	0	down
YGL205W	144.311081	67.762062	1.0906324	0	0	up
YOR065W	1066.545532	761.955444	0.485167015	0	0	-
YIL124W	304.228546	130.830948	1.217451679	0	0	up
YOL052C-A	7116.17334	5453.87793	0.383819243	0	0	-
YJL116C	88.788635	271.039764	-1.610057596	0	0	down
YDR377W	1870.320312	1145.970215	0.706715821	0	0	-
YOL083W	266.235748	128.901031	1.046440495	0	0	up
YPL271W	2740.790283	1620.440796	0.75820563	0	0	-

YBR126C	901.195679	695.378235	0.374042484	0	0	-
YMR034C	116.438065	291.243439	-1.32266278	0	0	down
YKL150W	1109.049438	805.208069	0.461890143	0	0	-
RDN18-2	203.522171	394.153748	-0.953572528	0	0	-
YCL040W	786.612183	593.772766	0.405741607	0	0	-
YDR046C	63.29406	173.746643	-1.456843086	0	0	down
YCR012W	621.723022	425.124939	0.548385108	0	0	-
YDL234C	606.331787	911.264832	-0.587762933	0	0	-
YDL171C	24.426975	59.327366	-1.280222518	0	0	down
YDR298C	1201.131958	824.927185	0.54205597	0	0	-
YJR123W	349.025696	747.87561	-1.09946508	0	0	down
YLR153C	79.588608	190.223633	-1.257062646	0	0	down
YIL047C	181.17215	323.902191	-0.838197027	0	0	-
YJL165C	121.498566	241.096436	-0.988671038	0	0	-
YPL127C	245.962555	88.250687	1.478759284	0	0	up
YLR410W-A	0.219766	59.487267	-8.080468884	0	0	down
YHR001W-A	3134.07666	2140.533447	0.55007009	0	0	-
YMR084W	154.318314	403.674652	-1.387283712	0	0	down
YPL004C	977.688354	724.278503	0.432830112	0	0	-
YKR093W	150.587662	69.573494	1.113993891	0	0	up
YHR099W	29.541397	15.155299	0.962915737	0	0	-
YKL029C	61.457775	158.724533	-1.368857691	0	0	down

YPR149W	2333.791016	1811.405884	0.365565529	0	0	-
YGL255W	63.399689	200.723618	-1.662662712	0	0	down
YER067W	2588.311035	2025.667725	0.35361345	0	0	-
YGR159C	52.370373	171.274963	-1.709491488	0	0	down
YBR169C	306.229675	204.249496	0.584281575	0	0	-
YGR087C	20.445087	89.724854	-2.133753469	0	0	down
RME2	125.35537	251.11908	-1.002347849	0	0	down
YMR246W	125.935989	256.18985	-1.024522695	0	0	down
YLR190W	85.538673	22.262735	1.941945986	0	0	up
YLR048W	129.838791	357.73053	-1.462151777	0	0	down
YBR005W	889.173584	1512.930176	-0.766808413	0	0	-
RDN18-1	162.649826	320.411407	-0.978156231	0	0	-
YDR033W	193.411423	73.236565	1.401036972	0	0	up
YOR128C	103.056931	232.255402	-1.172270617	0	0	down
YDR006C	124.518715	235.998276	-0.922413727	0	0	-
YDL182W	225.923889	443.056305	-0.971653225	0	0	-
YPL089C	107.402657	226.949875	-1.07934401	0	0	down
YMR238W	258.54599	482.304718	-0.899523994	0	0	-
YIL018W	174.567917	427.324158	-1.291542442	0	0	down
YPL055C	241.938232	496.299103	-1.03657108	0	0	down
YDR150W	64.509888	41.482658	0.637011974	0	0	-
YGL187C	1280.657227	853.813782	0.584891029	0	0	-

YEL036C	42.311508	135.90239	-1.683448818	0	0	down
YER061C	104.824005	251.937317	-1.265095694	0	0	down
YFL007W	44.369865	22.348526	0.989400482	0	0	-
YKL035W	409.101624	275.455933	0.570637727	0	0	-
YDR529C	3564.626709	2876.176025	0.309599031	0	0	-
YKL067W	1020.61145	634.105225	0.686639562	0	0	-
YJR109C	54.028625	119.259766	-1.142311539	0	0	down
YMR244W	7.147019	75.521057	-3.401465428	0	0	down
YGL006W	231.89595	365.901428	-0.657977422	0	0	-
YNL036W	731.653198	1265.495361	-0.790470338	0	0	-
YNL006W	339.419617	179.826248	0.916466332	0	0	-
YGL037C	1638.002197	1267.65625	0.369773709	0	0	-
YAL038W	1026.649414	1479.383789	-0.527052766	0	0	-
YMR122W-A	1726.185913	1055.833496	0.709205513	0	0	-
YPL023C	112.908943	233.449875	-1.047953056	0	0	down
YPL274W	29.650429	101.35553	-1.773299921	0	0	down
YLR029C	329.248688	696.052307	-1.080018034	0	0	down
YOR338W	228.76004	456.118317	-0.995573045	0	0	-
YKL085W	625.738464	432.271027	0.533623646	0	0	-
YJR073C	1160.555054	831.051147	0.481805787	0	0	-
YPL026C	234.703415	429.754517	-0.87267397	0	0	-
YML010W	60.626816	129.046005	-1.089857517	0	0	down

YOL036W	42.36087	112.165077	-1.404819433	0	0	down
YOL126C	365.100037	645.758545	-0.822703014	0	0	-
YBL001C	727.96991	345.238281	1.076286376	0	0	up
YNR067C	71.505753	34.876923	1.035786554	0	0	up
YBL007C	106.525726	188.535782	-0.823636473	0	0	-
YLR359W	160.484283	321.63266	-1.002981897	0	0	down
YIL130W	45.214291	106.683167	-1.238481812	0	0	down
YNL173C	302.803009	172.304443	0.813419639	0	0	-
YPL135W	1328.731079	2135.679688	-0.684646138	0	0	-
YOR100C	304.718719	572.963196	-0.910964343	0	0	-
YPR080W	2904.046387	3780.779541	-0.38061923	0	0	-
YAR015W	205.146713	432.169159	-1.074940082	0	0	down
YMR135C	376.466705	630.284607	-0.743481152	0	0	-
YKL068W	52.099686	116.897507	-1.16589758	0	0	down
YDR533C	547.947876	334.736725	0.71101182	0	0	-
YBR011C	687.834839	478.246887	0.524306613	0	0	-
YNL103W	87.978111	187.705429	-1.093253848	0	0	down
YDL055C	168.175385	72.040062	1.223095233	0	0	up
YEL046C	96.601677	236.205597	-1.289923011	0	0	down
YJL012C	52.989559	128.649551	-1.279666396	0	0	down
YGR060W	412.926117	729.024292	-0.820083218	0	0	-
YGR286C	18.655844	94.332466	-2.338126753	0	0	down

YOR197W	298.744049	525.79303	-0.815585043	0	0	-
YKL198C	52.345867	130.730576	-1.320449068	0	0	down
YNL243W	62.857662	132.175232	-1.072291342	0	0	down
YDR025W	199.917526	516.18927	-1.368495199	0	0	down
YIL123W	115.14167	46.860439	1.296967667	0	0	up
YER043C	57.54874	158.66777	-1.463152858	0	0	down
YLR099C	34.103374	122.990791	-1.850563913	0	0	down
YDL004W	1722.547363	1308.94873	0.396135062	0	0	-
YPR191W	1364.352051	1154.717896	0.240675522	0	0	-
YIL135C	134.186325	283.061615	-1.07687847	0	0	down
YOR230W	1136.453857	961.130859	0.241734334	0	0	-
YMR189W	44.756092	100.91465	-1.172979655	0	0	down
YDR155C	2663.206055	2209.200928	0.269639417	0	0	-
YHR203C	184.597778	409.645691	-1.149991452	0	0	down
YMR272C	303.572968	539.803589	-0.830391239	0	0	-
YMR191W	1562.904053	1354.853271	0.206092596	0	0	-
YPR145C-A	692.226807	1481.977661	-1.098206984	0	0	down
YNL065W	8.392848	49.800747	-2.568935025	0	0	down
YJL130C	29.51882	59.889729	-1.020673553	0	0	down
YML115C	47.620243	129.358215	-1.441724789	0	0	down
YER178W	606.480835	458.564056	0.403338781	0	0	-
YPL262W	417.395416	299.790619	0.477459517	0	0	-

YNL101W	102.096336	200.768143	-0.975599275	0	0	-
YPR028W	532.08905	301.686829	0.818616001	0	0	-
YOL016C	903.232422	1302.776367	-0.528420275	0	0	-
YDR050C	866.401855	633.767212	0.451083308	0	0	-
YPL163C	123.707924	34.619785	1.837269244	0	0	up
YDR232W	109.844696	225.792542	-1.039532625	0	0	down
YFR044C	154.684204	78.874207	0.971700379	0	0	-
YGR008C	2579.508789	1894.463257	0.445307203	0	0	-
YMR116C	82.98407	219.757629	-1.405006928	0	0	down
YJL138C	236.597565	435.742828	-0.881041693	0	0	-
YMR105C	493.588959	383.634766	0.363576656	0	0	-
YMR300C	33.932205	105.288513	-1.633620958	0	0	down
YPL159C	238.105469	486.201508	-1.02995361	0	0	down
YLR395C	3126.70459	2370.395508	0.399515123	0	0	-
YLR355C	82.839302	202.282593	-1.287984875	0	0	down
YNR019W	118.594818	228.04689	-0.943289523	0	0	-
YOR138C	55.169102	129.128174	-1.226871409	0	0	down
YPL221W	64.454559	137.053268	-1.088382417	0	0	down
YGR175C	254.174988	436.72522	-0.780903777	0	0	-
YBR117C	151.782425	90.614288	0.744194293	0	0	-
YCR004C	308.523712	163.152237	0.919162605	0	0	-
YMR072W	585.726379	356.153015	0.717729666	0	0	-

YNL111C	111.768005	377.477509	-1.75588343	0	0	down
YBL076C	32.038658	76.1791	-1.249581525	0	0	down
YKL043W	88.933807	214.972702	-1.273349623	0	0	down
YPR001W	720.02002	588.033447	0.292138804	0	0	-
YEL057C	127.702324	35.475941	1.847871923	0	0	up
YJL137C	100.817871	35.402508	1.509827921	0	0	up
YGL117W	211.96257	435.24826	-1.038029005	0	0	down
YGR250C	232.669632	166.418518	0.483466946	0	0	-
YML081W	34.877735	76.68988	-1.136729859	0	0	down
YKL065W-A	545.894104	200.615814	1.444185781	0	0	up
YBR054W	197.513824	100.270409	0.978057719	0	0	-
YOL130W	39.298653	93.15696	-1.245183697	0	0	down
YNR018W	201.732849	437.207336	-1.115871585	0	0	down
YDR216W	146.867859	231.485855	-0.656405333	0	0	-
YDL133C-A	1597.325806	602.788513	1.405934779	0	0	up
YEL066W	179.344513	55.386391	1.695130167	0	0	up
YKL060C	3669.178955	3514.49585	0.0621395187796	0	0	-
YMR008C	206.479507	345.255371	-0.741665256	0	0	-
YGR201C	158.500427	367.19635	-1.212064991	0	0	down
YER144C	70.435143	142.859177	-1.020226382	0	0	down
YNL069C	187.575974	428.572327	-1.192063648	0	0	down
YOR020C	1130.35791	728.191345	0.634390153	0	0	-

YHR170W	40.637001	112.266304	-1.466059137	0	0	down
YGL123W	157.2939	350.789337	-1.157142172	0	0	down
YKR072C	113.583458	223.091599	-0.973883447	0	0	-
YNL268W	124.351906	234.205551	-0.91334665	0	0	-
YDR505C	108.529419	195.87973	-0.851881946	0	0	-
YPL201C	131.716843	64.695503	1.0257025	0	0	up
YNL327W	91.370567	54.598919	0.74285712	0	0	-
YFR034C	132.675583	291.730499	-1.136733333	0	0	down
YFR039C	78.366661	174.087051	-1.151496962	0	0	down
YPL179W	109.33567	217.282578	-0.990808355	0	0	-
YBL017C	31.529139	65.750664	-1.060319691	0	0	down
YBR069C	35.787552	96.272484	-1.427665653	0	0	down
YLR307C-A	925.916748	521.534668	0.828119326	0	0	-
YPL113C	93.896431	34.008999	1.465153779	0	0	up
YPL078C	1723.766846	1466.104736	0.23357648	0	0	-
YBR053C	319.445374	204.260849	0.645156541	0	0	-
YML008C	181.144241	95.548668	0.922831273	0	0	-
YGL026C	51.914783	117.929802	-1.18371103	0	0	down
YHR064C	77.6129	169.644226	-1.12814396	0	0	down
YIR033W	8.297284	32.04327	-1.949310309	0	0	down
YGR046W	208.15863	384.775421	-0.886333272	0	0	-
YML120C	686.075928	568.629883	0.27087833	0	0	-

YBR207W	127.935341	253.793152	-0.988238291	0	0	-
YOR317W	587.279297	829.913086	-0.498913476	0	0	-
YBR089C-A	858.291992	497.54007	0.786655818	0	0	-
YOR335C	40.585442	90.146614	-1.151310978	0	0	down
YNL274C	210.503601	115.111412	0.870814046	0	0	-
YFL039C	455.535889	328.757324	0.470541685	0	0	-
YNL020C	65.510826	141.874985	-1.114814996	0	0	down
YJL110C	84.428535	177.302383	-1.070409341	0	0	down
YML085C	119.474426	56.681126	1.075761513	0	0	up
YFL021W	103.544373	209.414215	-1.016110221	0	0	down
YHL027W	198.965088	333.018707	-0.743087915	0	0	-
YIR037W	804.583801	545.507996	0.560642348	0	0	-
YDL231C	30.694082	69.846397	-1.186225174	0	0	down
YNL052W	3737.370117	3336.012695	0.163898664	0	0	-
YBR182C	31.7959	98.491814	-1.631163076	0	0	down
YBR079C	37.015255	83.253174	-1.169385308	0	0	down
RUF5-2	149.623367	54.791531	1.44931068	0	0	up
YBR150C	58.00214	111.61982	-0.94441519	0	0	-
YDR483W	429.798035	667.763306	-0.635677932	0	0	-
YOL059W	150.201767	285.762146	-0.927913035	0	0	-
YBR208C	14.263115	35.24744	-1.305229385	0	0	down
YMR136W	191.975449	328.86441	-0.776571065	0	0	-

YHR023W	8.387052	1.161407	2.852290166	0	0	up
YFL030W	354.324249	244.36026	0.536060522	0	0	-
YLR164W	309.315735	147.522995	1.068140375	0	0	up
YKL146W	198.30896	323.784546	-0.707284266	0	0	-
YER064C	29.034063	87.872299	-1.597661964	0	0	down
YHR152W	166.85321	53.329132	1.64558369	0	0	up
YGR162W	20.115717	54.913036	-1.448825514	0	0	down
YBL103C	62.887436	146.4552	-1.219615697	0	0	down
YMR250W	428.888031	340.994446	0.330852814	0	0	-
YNL216W	26.95253	69.650566	-1.369714373	0	0	down
YCR053W	49.344517	121.935555	-1.305157169	0	0	down
YLR432W	26.93153	82.507988	-1.615237608	0	0	down
YBL093C	237.409927	473.930786	-0.997296119	0	0	-
YML128C	1052.199463	938.513184	0.164959302	0	0	-
YHR051W	1464.748535	1136.796753	0.365678668	0	0	-
YNL088W	22.329943	7.890641	1.50076516	0	0	up
YBL030C	772.046387	614.565552	0.329120629	0	0	-
YNL178W	333.50296	599.501221	-0.846063126	0	0	-
YAR018C	51.409836	13.112422	1.971110219	0	0	up
YER062C	65.724258	187.092331	-1.509252568	0	0	down
YOL030W	83.639862	35.58654	1.232859012	0	0	up
YDR172W	70.915298	143.150345	-1.01336236	0	0	down

YMR081C	818.406616	1193.638428	-0.544476175	0	0	-
YIL158W	65.931366	6.918752	3.252381238	0	0	up
YIR036C	290.631592	170.04657	0.773261628	0	0	-
YGR209C	730.505615	427.605133	0.772616191	0	0	-
YGL166W	396.19754	693.36554	-0.807396214	0	0	-
YML004C	581.788452	442.459167	0.39495034	0	0	-
YOR142W-B	14.933079	4.602704	1.69795809	0	0	up
YMR147W	78.117371	14.740023	2.405904621	0	0	up
YKL171W	139.611954	227.330032	-0.703365813	0	0	-
YLR357W	20.432854	55.565628	-1.443302002	0	0	down
YOR133W	127.70871	215.007523	-0.751530217	0	0	-
YLR248W	172.584686	291.585571	-0.756614875	0	0	-
YPR198W	54.745953	125.997864	-1.202575051	0	0	down
YNL284C-A	0.50106	26.23041	-5.710113177	0	0	down
YOR142W	297.30658	191.168076	0.637109772	0	0	-
YGR118W	328.641479	661.04425	-1.008232269	0	0	down
YBR082C	543.930664	327.625946	0.731373146	0	0	-
YER145C	32.200214	99.008278	-1.620478876	0	0	down
YBR126W-A	795.895508	409.212799	0.959727765	0	0	-
YIL038C	24.744034	63.725983	-1.364801002	0	0	down
YPR013C	93.387276	213.702393	-1.194305162	0	0	down
YAR035W	689.596924	942.20874	-0.450293375	0	0	-

YBR066C	33.610218	131.553009	-1.968672442	0	0	down
YJR092W	15.022444	3.961498	1.923001565	0	0	up
RUF5-1	135.397705	51.087639	1.406157117	0	0	up
YKL126W	79.525841	153.819504	-0.951742817	0	0	-
YOR215C	696.54541	490.184784	0.506891708	0	0	-
YDR233C	125.94751	268.177094	-1.090363417	0	0	down
YDR516C	252.537689	176.709198	0.515121577	0	0	-
YHL011C	123.770691	258.582153	-1.062952982	0	0	down
YMR001C	31.518097	8.70927	1.855556726	0	0	up
YER114C	35.087154	74.938255	-1.094759452	0	0	down
YPR163C	95.264977	196.253113	-1.042697711	0	0	down
YJR145C	152.28566	316.969604	-1.057564402	0	0	down
YGR130C	206.863113	155.750824	0.409436617	0	0	-
YDL013W	38.991615	94.064018	-1.270479048	0	0	down
YKL148C	625.07373	865.34906	-0.469255825	0	0	-
YEL063C	169.731628	286.036072	-0.752941673	0	0	-
YLL029W	198.182922	311.248322	-0.651233413	2.70118024442e-306	3.35378539147e-305	-
YBL033C	264.625061	452.378693	-0.773581283	9.87138788541e-306	1.22318514955e-304	-
YOR361C	38.06316	86.135826	-1.178218076	1.188706559e-305	1.47001799168e-304	down
YCR007C	187.663132	375.960144	-1.00243448	2.34064940839e-305	2.88881740105e-304	down
YDR277C	161.701401	291.585388	-0.850586246	2.42062099984e-304	2.98159031092e-303	-
YML058W	1007.335632	678.495483	0.570133336	4.98308394609e-304	6.12573963115e-303	-

YBR200W	89.308533	174.453568	-0.965973175	1.7074168438e-302	2.09479125816e-301	-
YLR109W	1198.851196	950.802612	0.334434828	4.34340239482e-302	5.31831204478e-301	-
YPR040W	111.211266	229.529724	-1.04537805	1.21677126975e-301	1.48695197689e-300	down
YPR151C	666.658386	482.987885	0.464960673	3.59857049907e-301	4.3889834299e-300	-
YDR012W	166.464462	309.523956	-0.894836859	5.49535175882e-301	6.68924386642e-300	-
YDR234W	19.102646	56.364746	-1.561020607	2.15467054418e-300	2.61765063372e-299	down
YER026C	219.057373	122.38858	0.839839826	1.61082981618e-299	1.95313115212e-298	-
YOR084W	100.021721	44.834225	1.157640969	7.53758041567e-299	9.1215008227e-298	up
YJR095W	218.058578	129.371414	0.753196871	1.0462101492e-298	1.26359389226e-297	-
YCL043C	240.940155	387.107086	-0.684057864	1.49124880678e-298	1.79760632863e-297	-
YLL018C-A	513.302734	992.441833	-0.951172604	2.67148124406e-298	3.21406115565e-297	-
YNL270C	51.493305	19.333548	1.413278451	1.77100081704e-297	2.12657119384e-296	up
YBR036C	449.285004	679.882385	-0.597654282	3.1943122446e-297	3.82824139276e-296	-
YHR150W	89.487854	171.873596	-0.941584142	3.8392423277e-297	4.59229602512e-296	-
YKL180W	300.717743	568.561157	-0.918905548	3.9013340423e-297	4.65759264127e-296	-
YBR177C	210.399734	138.457718	0.603687405	1.41296577102e-296	1.68362600892e-295	-
YBR038W	30.049417	11.163263	1.428578215	1.23499488041e-295	1.46874486927e-294	up
YML063W	164.023376	331.78656	-1.01635401	1.44135913455e-295	1.71089053677e-294	down
YBR012W-B	11.434978	3.046037	1.908448219	7.90060351422e-295	9.36010431608e-294	up
YIL154C	144.84465	279.229004	-0.946942407	4.09454172665e-292	4.84169810268e-291	-
ITS2-2	238.339676	616.545044	-1.371187165	1.40261155097e-291	1.65540161757e-290	down
YMR251W	51.414551	131.68074	-1.356795724	5.07968172312e-291	5.98380723665e-290	down

YGR067C	53.499321	107.011459	-1.000172805	1.39545627683e-290	1.64071828912e-289	down
YGR268C	366.050934	648.544312	-0.825160745	1.63749396386e-290	1.92165643245e-289	-
YLR375W	174.359329	322.143036	-0.885637851	1.66908057944e-290	1.95502872399e-289	-
YBL067C	63.315872	123.628029	-0.965366764	2.9195271866e-290	3.41326266938e-289	-
YJL101C	30.381962	75.388229	-1.311124242	4.72805908119e-290	5.51725390527e-289	down
YEL007W	48.304523	104.379494	-1.111608126	2.0484291153e-289	2.38586265437e-288	down
YAL035W	35.207996	74.124817	-1.074053525	5.15613461861e-289	5.9942478862e-288	down
YDR275W	304.282776	540.034607	-0.82763919	7.07090061912e-289	8.20488804551e-288	-
YAL040C	21.397066	63.753849	-1.575099459	8.26600776218e-289	9.57376421411e-288	down
YOR322C	38.037178	82.935234	-1.124574927	8.32976936962e-289	9.62964771817e-288	down
YPL111W	57.166706	145.063904	-1.343441512	8.1315953062e-287	9.38307502991e-286	down
YIR038C	664.629211	501.289764	0.406904926	6.68100497004e-286	7.69493114174e-285	-
YMR005W	56.85083	137.233276	-1.271377028	7.52924035888e-286	8.65583780517e-285	down
YNR006W	168.219574	276.885956	-0.718946293	1.0490777275e-285	1.20382154017e-284	-
YNL300W	180.823059	45.908749	1.977737641	2.10653495919e-284	2.4127987134e-283	up
YOR142W-A	0.136372	23.72205	-7.44253742	3.32488007993e-284	3.80126252969e-283	down
YDR070C	2066.905029	1630.656494	0.342019198	4.69567327799e-284	5.35859185842e-283	-
YJR085C	1251.484497	911.124756	0.457919905	2.24871640542e-283	2.5614736596e-282	-
YOR140W	35.982349	80.728897	-1.165795809	2.96189961856e-282	3.36766901685e-281	down
YDL067C	2929.668945	2269.540771	0.368337242	8.83371820413e-282	1.0025543439e-280	-
YER091C	117.413269	198.072433	-0.754432647	3.21271966222e-281	3.63951891662e-280	-
YHL021C	339.562714	257.774719	0.397567271	3.25153643069e-281	3.67678290742e-280	-

YFL010C	234.350693	448.83609	-0.937519624	6.12214021735e-281	6.91022663078e-280	-
YOR213C	170.475433	339.315826	-0.993064871	1.01964974254e-280	1.14881771355e-279	-
YHR087W	1291.085327	961.569031	0.425122013	4.73113392092e-280	5.32081148208e-279	-
YHR028C	102.53347	175.129105	-0.772323942	1.83405501565e-279	2.05891745699e-278	-
YGL200C	277.0914	154.851135	0.839479978	5.78011688061e-279	6.47706960195e-278	-
YLL043W	75.199509	143.211533	-0.929352532	1.30448528153e-278	1.45914317617e-277	-
YGR109W-B	22.309526	46.732658	-1.066771234	1.33978088286e-277	1.49592800733e-276	down
YDL147W	363.759155	278.380005	0.385927966	1.56165124503e-277	1.74052619913e-276	-
YML075C	46.864902	89.514748	-0.933617527	9.44864845125e-277	1.05120447286e-275	-
YDR333C	20.740299	56.567997	-1.447549395	1.27360343398e-276	1.41440610342e-275	down
YDR464W	20.135307	44.279537	-1.136912668	2.71932397014e-276	3.01456485833e-275	down
YKL174C	51.554745	110.078979	-1.094361872	6.88840704753e-275	7.62267931392e-274	down
YGL043W	83.038788	188.705292	-1.18427759	1.58006337125e-274	1.7453796101e-273	down
YDL014W	60.688938	149.347076	-1.299163512	4.78516606486e-273	5.27643178164e-272	down
YGR189C	279.315613	208.49025	0.421916301	9.88220856193e-273	1.08774380767e-271	-
YOL027C	84.685669	161.757812	-0.933645634	4.09173895336e-272	4.49584343761e-271	-
YDR122W	48.373161	91.070442	-0.912776071	3.00549946514e-271	3.29649128615e-270	-
YKL175W	82.942619	164.245453	-0.985667924	3.59565805205e-271	3.93683336634e-270	-
YGR249W	67.682289	28.109087	1.267741766	1.25656760733e-269	1.37337530041e-268	up
YJL111W	107.638794	196.047333	-0.865003884	1.2605252463e-269	1.37527956574e-268	-
YJR016C	43.945705	99.294601	-1.175993104	1.3695567874e-269	1.49161553266e-268	down
YGL167C	46.737995	90.843872	-0.958793353	1.47224961214e-267	1.60065246798e-266	-

YAL061W	350.66568	540.406982	-0.623950075	1.87546941962e-267	2.03547450297e-266	-
YOR151C	39.787071	75.591591	-0.925926058	7.801205071e-267	8.45198622701e-266	-
YDL130W-A	2917.5979	2436.105713	0.260204327	1.45771591261e-266	1.57656801141e-265	-
YER047C	26.767059	61.520435	-1.200607071	3.18574411565e-266	3.43949555999e-265	down
YFL031W	479.708954	767.802185	-0.678575297	3.46798073692e-265	3.73771257201e-264	-
YDR270W	15.72799	41.460609	-1.398406998	3.57841778707e-265	3.85005504716e-264	down
YPL180W	43.875763	90.35392	-1.04216298	4.95860461852e-265	5.32578156951e-264	down
YML116W	34.808716	86.266579	-1.309353147	7.12703040472e-265	7.64155522496e-264	down
YLR295C	1812.382446	1487.855713	0.284652797	7.55640975863e-265	8.08796410028e-264	-
YJL166W	3366.912109	2911.425049	0.209700582	6.2779672287e-264	6.70802419204e-263	-
YJL089W	32.88166	12.765648	1.365016361	1.30981970044e-262	1.3971410138e-261	up
YDL048C	123.240562	222.192413	-0.850332389	1.47492662492e-262	1.57055651586e-261	-
YHR079C	14.924791	38.458496	-1.365591615	7.33102367248e-262	7.79297858883e-261	down
YML076C	38.650112	78.486221	-1.021966803	1.61736036032e-261	1.71633728494e-260	down
YOR219C	50.352001	96.055847	-0.931824322	2.0382068206e-261	2.15924708913e-260	-
YNL079C	543.353577	385.294891	0.495928254	5.35356505056e-259	5.66182825109e-258	-
YLR410W-B	25.39119	13.308847	0.931942421	3.40983885199e-257	3.60004754985e-256	-
YOL139C	222.238998	421.072052	-0.921955122	3.95923846547e-257	4.17299701081e-256	-
YAL056W	89.827919	152.700394	-0.765467967	4.14316089173e-257	4.35944793489e-256	-
YGL219C	193.973648	322.224152	-0.732203963	3.80964445886e-256	4.00173820653e-255	-
YLR194C	601.849365	463.065857	0.378185057	8.53330282064e-256	8.94843647137e-255	-
YJR112W	127.645149	48.501591	1.396034733	2.57795637082e-255	2.69881166105e-254	up

YHR205W	50.923473	99.182404	-0.961753379	3.65818512684e-255	3.8232345568e-254	-
YML118W	142.210159	245.902344	-0.790060957	3.84589826263e-255	4.01266158225e-254	-
YPL137C	24.71073	51.827141	-1.06857018	6.17532767522e-255	6.43228761875e-254	down
YMR305C	335.589142	249.850983	0.425628144	1.45646362588e-254	1.51452699991e-253	-
YNL194C	1109.6875	1532.350464	-0.465592839	4.36904208678e-254	4.53562094895e-253	-
YIL083C	102.67379	205.353271	-1.000039982	1.25150485682e-253	1.29705211205e-252	down
YGR052W	116.416405	225.005035	-0.950662913	2.03090396281e-253	2.10130863352e-252	-
YGL030W	716.456543	461.183899	0.635537054	3.05505852546e-253	3.15570770816e-252	-
YBR114W	32.292965	71.703339	-1.150820393	2.14373106421e-252	2.21067814728e-251	down
YDL224C	38.211433	85.492401	-1.161791829	8.02933129211e-252	8.26634969509e-251	down
YMR217W	43.588421	99.887161	-1.19635431	1.73835155432e-251	1.78670305451e-250	down
YPL204W	77.341568	153.592926	-0.989795853	2.43966992433e-251	2.50338361822e-250	-
YOR196C	84.9403	172.2547	-1.020022238	5.08696930745e-251	5.21120552156e-250	down
YDR101C	16.560211	51.189159	-1.628117249	6.65604303026e-251	6.80736657856e-250	down
YMR019W	20.631708	49.573895	-1.264717355	2.47952396141e-250	2.53172446586e-249	down
YDR322C-A	1020.193237	712.251404	0.518383977	5.51104992068e-250	5.61783216873e-249	-
YOR204W	213.329117	159.097992	0.423165263	3.13285637564e-249	3.18832334098e-248	-
YMR243C	118.119316	217.304703	-0.879474491	4.38516075353e-249	4.45549557413e-248	-
YMR208W	84.70282	168.588943	-0.993028012	4.67579720213e-249	4.74303088739e-248	-
YBR143C	65.720863	140.444824	-1.095580126	7.10835646895e-249	7.19880537671e-248	down
YIL131C	28.395721	5.821245	2.286273901	3.10381057027e-248	3.13818501958e-247	up
YDL010W	191.641037	365.759521	-0.932488894	4.83695189984e-248	4.88256868199e-247	-

YDR418W	253.053391	489.62796	-0.952244138	6.04707191737e-248	6.09419195829e-247	-
YDL185W	158.187225	236.124619	-0.577915374	6.08950024372e-248	6.12700445916e-247	-
YGL121C	1047.773193	787.697632	0.411612613	2.38942160049e-247	2.40024745888e-246	-
YNL054W	13.972281	35.529831	-1.346463264	1.15249495689e-246	1.15584631541e-245	down
YLR337C	43.407932	87.30703	-1.008139132	4.80611208199e-245	4.81231351693e-244	down
YGR110W	217.512558	353.152313	-0.699191849	9.61869366607e-245	9.61559585813e-244	-
YBL042C	36.187962	81.840378	-1.177302947	1.9686483881e-243	1.96485035262e-242	down
YBR065C	98.925491	197.517532	-0.997566489	4.4278215932e-242	4.41218562609e-241	-
YGL244W	43.981415	97.553352	-1.149297425	5.48066212832e-242	5.45255616868e-241	down
YDL047W	161.953064	298.329193	-0.881329397	6.98983207732e-242	6.94286040576e-241	-
YBR059C	61.367668	106.133591	-0.790330671	9.60976348275e-242	9.52993797139e-241	-
YDR463W	240.323273	373.232361	-0.635097714	4.14367953356e-240	4.10270534998e-239	-
YNL239W	191.240875	130.661301	0.549559002	7.43946246278e-239	7.35416926257e-238	-
YOL113W	51.133827	24.078535	1.086530388	2.06222668841e-237	2.03534233412e-236	up
YPL242C	22.328445	10.490801	1.089757947	2.61021539888e-237	2.57209796766e-236	up
YBR191W	157.678101	344.737549	-1.12851614	3.87802753929e-237	3.81533993089e-236	down
YDR389W	51.064087	103.236549	-1.015572905	1.04116549314e-235	1.02271445908e-234	down
YPR084W	29.012276	78.06752	-1.428058961	1.24473375753e-235	1.22074362824e-234	down
YDR111C	185.899353	130.346878	0.512165721	1.45496643761e-235	1.42467376099e-234	-
YLL022C	57.324459	20.632727	1.474216322	1.66483836092e-235	1.62760890466e-234	up
YGL021W	24.69273	7.761091	1.669754972	1.12682455746e-234	1.09989415924e-233	up
YFL041W	32.181633	75.195602	-1.22441075	1.97735097497e-234	1.92706355614e-233	down

YNL311C	32.127968	70.530525	-1.134418041	2.00327362263e-233	1.94926687293e-232	down
YGR256W	133.432449	84.348122	0.661681701	2.22770049162e-233	2.16425111925e-232	-
YPR081C	34.868896	79.450546	-1.188116447	1.25910157532e-232	1.22132852806e-231	down
YNL058C	79.169441	31.568619	1.326452515	1.73067537701e-232	1.67613615296e-231	up
YDR406W	47.099266	79.909203	-0.762657088	5.72505493395e-232	5.5360032757e-231	-
YHL029C	17.167671	48.105228	-1.486499359	1.23276550363e-231	1.1902034598e-230	down
YMR131C	29.870239	75.893753	-1.345272358	5.12878878188e-231	4.94402496241e-230	down
YGL008C	153.1017	118.368675	0.371202965	2.22232832904e-229	2.13894794833e-228	-
YMR205C	73.298828	124.549202	-0.764853742	2.16566170492e-228	2.08118078392e-227	-
YPL048W	299.440643	226.009628	0.405885819	6.65837212607e-228	6.38874407398e-227	-
YGL181W	132.443848	238.670273	-0.849638054	6.73465487387e-228	6.45196565694e-227	-
YMR309C	18.12623	46.424278	-1.356800577	6.79188067055e-228	6.49676351353e-227	down
YLR027C	156.881775	269.985474	-0.783204024	1.15196173879e-227	1.10021207299e-226	-
YOR302W	293.096313	972.515137	-1.730345886	1.42515784265e-227	1.35904452952e-226	down
YOR261C	327.891296	241.44841	0.441502642	4.37817726837e-227	4.16866939909e-226	-
YEL037C	198.494965	329.575684	-0.731507392	7.08572136103e-227	6.73631825563e-226	-
YDR513W	571.681946	390.101379	0.551363632	9.31065190308e-227	8.83800107252e-226	-
YGR214W	185.898361	342.996826	-0.883681176	1.64383497028e-226	1.55800419778e-225	-
YNL054W-A	0.095571	19.216961	-7.651591575	5.98508573823e-226	5.66393479618e-225	down
YOR098C	23.640217	50.878174	-1.105803615	6.91221018554e-226	6.53135476892e-225	down
YBR016W	736.327515	1183.340698	-0.684445985	1.75144969488e-225	1.65243156623e-224	-
YGR243W	1809.352295	1559.486938	0.21440187	2.26244528618e-225	2.13129898886e-224	-

YLL051C	30.527683	68.692772	-1.170040208	3.46482388448e-225	3.25903434468e-224	down
YMR031C	235.605255	194.535629	0.27633731	3.6584935563e-225	3.43599515848e-224	-
YGL023C	65.462395	123.95916	-0.921126593	4.28413730784e-225	4.01751123974e-224	-
YDR527W	64.732826	134.792801	-1.058174054	2.37488734477e-224	2.22372558618e-223	down
YBR290W	192.608246	334.075623	-0.794505246	1.69632498109e-223	1.58596166907e-222	-
YLR257W	558.348328	815.544556	-0.546598264	5.7861600895e-223	5.40157621588e-222	-
YDL039C	13.678948	40.769547	-1.575534645	7.50878535273e-223	6.99918010056e-222	down
YHR110W	183.811111	96.577301	0.928467926	9.34553190403e-223	8.69821020393e-222	-
YPL189C-A	947.338806	606.23175	0.644011071	1.58647196463e-222	1.47437394558e-221	-
ITS2-1	181.4384	469.279419	-1.370967367	2.5038973663e-222	2.32349698804e-221	down
YGR266W	66.468781	37.72757	0.817057716	2.62175094605e-222	2.42922833926e-221	-
YNR059W	67.4095	128.929688	-0.935560675	5.39224057814e-221	4.98882705054e-220	-
YLL010C	92.491531	176.459778	-0.931946199	7.54266566958e-221	6.96798638047e-220	-
YKL216W	57.821182	136.343842	-1.237579536	2.71029587029e-219	2.50007678495e-218	down
YNL093W	419.376678	672.924255	-0.682197489	3.04522970373e-218	2.8048643918e-217	-
YER131W	114.840858	296.676605	-1.36925515	5.52003007487e-218	5.07679210441e-217	down
YLR219W	109.757614	180.542969	-0.718021213	7.5941929905e-218	6.97407545636e-217	-
YDR385W	146.596756	224.673615	-0.61597753	1.06848175141e-217	9.79783561708e-217	-
YNL116W	103.671036	183.786377	-0.826016947	1.26678724763e-217	1.15991375122e-216	-
YBR138C	40.137901	14.828689	1.436574118	3.8250116028e-217	3.49715346542e-216	up
YGR191W	32.87648	75.203651	-1.193746862	7.36750403998e-217	6.72609780591e-216	down
YAL042W	105.519066	195.711197	-0.891222598	8.85872982071e-217	8.07562330792e-216	-

YER121W	153.61235	49.017803	1.647916481	1.37446496393e-216	1.25112587919e-215	up
YPR036W-A	13524.02344	13214.56445	0.0333955465439	7.68147787673e-216	6.9819347963e-215	-
YMR144W	30.615494	5.107696	2.583517396	3.81613765737e-215	3.46353546447e-214	up
YFR015C	273.839722	226.920441	0.27114516	4.49933473959e-215	4.07764526473e-214	-
YIR023W	11.475889	31.809608	-1.470856674	4.50644710394e-215	4.07813755412e-214	down
YKR039W	574.882935	778.246765	-0.436959469	6.76331102691e-215	6.11159168196e-214	-
YKR080W	43.969105	112.004456	-1.348994058	7.42721495108e-215	6.70176604888e-214	down
YDL012C	126.986237	327.224823	-1.365610051	8.13936991484e-215	7.33370223967e-214	down
YDR146C	32.576664	12.990582	1.326372805	1.29426237183e-214	1.16446098613e-213	up
YDR264C	33.501022	70.858673	-1.08073934	6.83472596852e-214	6.14037320008e-213	down
YGR244C	592.105774	510.768951	0.213184094	8.8158793901e-214	7.90881203089e-213	-
YPL117C	486.198517	729.693665	-0.585745436	9.12240864751e-214	8.17199320112e-213	-
YKL117W	630.059814	491.197388	0.359185907	1.4250649002e-213	1.27475546116e-212	-
YPR138C	233.975586	361.578705	-0.627951713	1.57749111984e-213	1.4090740823e-212	-
YBR047W	165.051956	76.075706	1.117412513	1.77601656439e-213	1.5841251195e-212	up
YNL284C-B	11.334166	4.072938	1.476536479	6.46382842595e-213	5.75716597824e-212	up
YMR241W	34.288971	95.641022	-1.479884936	6.05979564545e-212	5.38957182907e-211	down
YMR032W	25.978214	8.367844	1.634374388	1.16785404785e-211	1.03720142047e-210	up
YDL181W	2177.202637	1797.733398	0.276296602	1.96509842404e-211	1.74276157378e-210	-
YLR175W	34.316341	82.291969	-1.26185591	4.60430612699e-211	4.07753672416e-210	down
YKL173W	31.142002	62.154297	-0.996992442	7.31415180553e-211	6.46812740865e-210	-
YGR034W	134.439407	318.970184	-1.246465489	1.89031175677e-210	1.66928241622e-209	down

YIL033C	376.658386	302.644806	0.315631058	5.68645573941e-209	5.01442006112e-208	-
YEL024W	1819.364502	1658.971558	0.133145457	8.79732231034e-209	7.74663502165e-208	-
YIL094C	137.082214	243.797424	-0.830641484	8.79168614208e-208	7.73070645468e-207	-
YOR330C	22.477863	45.956982	-1.031779176	9.63427041912e-208	8.45962528456e-207	down
YOR145C	130.97226	251.864044	-0.943383898	9.95365861657e-208	8.72772778131e-207	-
YJL054W	45.340401	99.611519	-1.135515433	2.89922945584e-207	2.53856367587e-206	down
YOR256C	46.657345	88.572067	-0.924747573	1.14022825232e-206	9.96977040902e-206	-
YPL200W	120.34687	42.46188	1.502958467	2.8003549675e-206	2.44509193224e-205	up
YPR041W	55.245213	120.040138	-1.119595517	5.3807918405e-206	4.69156681823e-205	down
YNL220W	63.179718	129.726761	-1.037942717	3.8033150066e-205	3.31149783464e-204	down
YMR089C	72.476822	124.437569	-0.779830514	4.09864944004e-205	3.5636436588e-204	-
YGR183C	3388.946777	2892.898926	0.228321061	1.03750799864e-204	9.00818133644e-204	-
YOR122C	919.118774	699.673645	0.393569157	2.01982002235e-204	1.75126294675e-203	-
YMR266W	41.89064	78.265297	-0.90174483	3.92526937599e-204	3.39861538161e-203	-
YDR214W	196.656693	131.642746	0.579051262	7.7266946215e-204	6.68068526606e-203	-
YHR033W	51.035728	111.626534	-1.129100524	2.15159719864e-203	1.85773510558e-202	down
YJL117W	87.965431	180.161987	-1.03428606	2.34554092895e-203	2.02237751207e-202	down
YIL022W	163.349258	271.268341	-0.731760783	1.13294403583e-202	9.75494670519e-202	-
YLR209C	67.258064	26.065861	1.367545737	1.60224680174e-202	1.37766594809e-201	up
YKR011C	81.889503	38.879051	1.074685527	4.13934488724e-202	3.55422587275e-201	up
YJR008W	616.83136	522.059265	0.240662521	7.74622598528e-202	6.64206780616e-201	-
YKR059W	115.098747	208.329391	-0.85599426	9.04456678379e-202	7.74464421983e-201	-

YGL116W	33.251286	12.668983	1.392109421	6.5235454488 2e-201	5.5782603507 2e-200	up
YJL221C	22.220194	5.656531	1.97388195	8.7796078416 7e-201	7.4970846604 e-200	up
YOR254C	32.537273	70.914894	-1.123995322	1.0214456486 7e-200	8.7103497073 1e-200	down
YOR310C	24.07029	62.684517	-1.38085562	3.5793481033 8e-200	3.0480923217 8e-199	down
YDR293C	112.601898	167.385529	-0.571943662	4.1357625248 5e-200	3.5170977745 6e-199	-
YBL087C	90.444946	234.79895	-1.376314163	4.4659671619 3e-200	3.7927119208 3e-199	down
YDR316W-B	7.982739	2.21768	1.847832635	1.1233994209 e-198	9.5274092963 8e-198	up
YJL001W	440.57077	323.027649	0.447716126	5.4187978754 1e-198	4.5893447763 4e-197	-
YNL019C	39.031921	104.904221	-1.426346355	1.9531155968 7e-197	1.651899404e-196	down
YIL172C	21.976927	5.636611	1.963089759	4.4732409184 6e-197	3.7782149145 3e-196	up
YPR020W	710.374329	503.496277	0.496598337	4.820131752e-197	4.0656763473 4e-196	-
YAR002C-A	111.178429	48.632164	1.192894207	8.6877878845 6e-197	7.3180172574 4e-196	up
YDL203C	40.355141	83.796371	-1.05413529	1.4153887601 1e-196	1.1906142849 2e-195	down
YBR076W	62.028374	25.42355	1.286762813	2.5330056888 2e-196	2.1278618831 2e-195	up
YPL212C	17.701395	49.963512	-1.49701183	9.8537356739 4e-196	8.2664852788 9e-195	down
YBR248C	24.155649	60.69529	-1.329223952	1.1670957288 7e-195	9.7777736637 3e-195	down
YOR036W	502.638947	741.858154	-0.561620902	1.6503819940 9e-195	1.3808047734 9e-194	-
YIL106W	133.598282	76.636429	0.801799213	2.6517207298 2e-195	2.2155965398 e-194	-
YPL054W	225.138672	373.790741	-0.731416944	5.4818961241 e-195	4.5741412820 4e-194	-
YMR319C	26.167313	63.85379	-1.287006464	3.6728495156 4e-194	3.0605435963 8e-193	down
YDR019C	105.539642	192.296616	-0.86554838	3.3735565910 4e-193	2.8073779245 5e-192	-
YOR183W	0.658283	52.121586	-6.307029233	3.6871137462 7e-193	3.0642037666 5e-192	down

YDR357C	186.584427	79.342117	1.233669782	4.7486882688e-193	3.9411573225 6e-192	up
YFL037W	93.720993	54.458675	0.783210359	4.7490020233 7e-193	3.9361554821 2e-192	-
YLR291C	38.912045	93.952271	-1.271711232	5.8594501866 4e-193	4.8500622344 9e-192	down
YER169W	42.243393	81.050392	-0.940093446	1.1875484959e-192	9.8166458888 8e-192	-
YOR061W	96.559479	186.424774	-0.949103796	1.2171973752 6e-192	1.00483528e-191	-
YLR417W	36.28191	79.242371	-1.127021641	1.6478432482e-192	1.3585406221 6e-191	down
YJR126C	90.424843	147.715622	-0.708031317	1.8507086650 3e-192	1.5237664976 8e-191	-
YER006W	49.770634	101.93856	-1.034333209	2.7747305277 5e-192	2.2815267703 6e-191	down
YPR145W	596.444397	800.201416	-0.423975531	3.8978326834 8e-192	3.2007599601 9e-191	-
YJR117W	109.985924	67.688713	0.700331708	4.1654570321 3e-192	3.4160049214 6e-191	-
YGR253C	464.692902	361.434143	0.362544811	4.6554114619 4e-192	3.8127697039 2e-191	-
YKL059C	74.087578	142.927185	-0.947976769	1.0592086191 6e-191	8.663461275e-191	-
YLR213C	35.350861	85.004265	-1.265789875	1.1989820433 2e-191	9.7937901643 8e-191	down
YLR172C	125.924088	69.829269	0.850650507	2.8187076468 3e-191	2.2994135442 2e-190	-
YGR155W	59.868351	117.52417	-0.973092053	4.5186308199e-191	3.6813202270 3e-190	-
YPL093W	24.47662	57.683949	-1.236765585	5.5652234855 6e-191	4.5280350456 6e-190	down
YLR044C	271.281616	396.425781	-0.547259506	8.3858425386 7e-191	6.8140458743 5e-190	-
YKL142W	1150.781616	1007.69397	0.191556511	9.8299508311 2e-191	7.9770372234 8e-190	-
YNR017W	99.075882	211.147629	-1.091646236	1.8668624423 3e-189	1.5129872117 5e-188	down
YJL056C	13.172003	34.346348	-1.382681962	3.4068444446 2e-189	2.7574563640 4e-188	down
YER036C	184.565765	280.724701	-0.605021039	6.6599249768 1e-189	5.3834393562 6e-188	-
YOL148C	32.646061	71.661087	-1.134280997	3.6575399641 5e-188	2.9526668527 2e-187	down

YGR054W	22.60116	54.506329	-1.270026938	4.62734409034e-188	3.73072105361e-187	down
YBL027W	401.682251	649.166077	-0.692532897	1.94392391891e-187	1.56522434352e-186	-
YCL030C	22.116983	50.052502	-1.178287588	6.68742655497e-187	5.3776611468e-186	down
YLR353W	26.688799	8.983895	1.570821414	8.73155452237e-187	7.0123532309e-186	up
YPR159W	40.081551	79.130867	-0.981302228	6.49848138683e-186	5.21221866272e-185	-
YBL078C	818.041016	610.847534	0.421360849	1.79930458817e-185	1.44130101721e-184	-
YKR086W	15.496041	35.611164	-1.200429917	1.40880597825e-184	1.1270447826e-183	down
YHL009W-B	6.365535	1.531317	2.055508804	2.09659878746e-184	1.6751203697e-183	up
YOR035C	140.530243	213.471039	-0.603159716	2.18605935958e-184	1.74435173576e-183	-
YPL226W	9.051166	23.941864	-1.403359916	3.45340833103e-184	2.75208715264e-183	down
YOR185C	279.560822	187.17128	0.578803112	2.01020358854e-182	1.59991588175e-181	-
YDR034W-B	507.200562	241.547134	1.070251609	3.45661383215e-182	2.74758753777e-181	up
YDL126C	373.645996	337.404907	0.147191107	4.55960159196e-182	3.61969394922e-181	-
YDR028C	60.788246	101.302177	-0.736800882	5.82157755262e-181	4.6156262384e-180	-
YIL069C	132.656921	295.950439	-1.157655651	6.88322862068e-181	5.45039327515e-180	down
YLR056W	170.04895	282.946014	-0.734576716	8.29148512361e-181	6.55713880858e-180	-
YBR025C	129.804199	82.356201	0.656387867	2.57261479289e-180	2.03190745983e-179	-
YKR054C	11.153047	6.814992	0.710654038	1.81439580318e-179	1.43122860815e-178	-
YMR037C	32.513218	67.471992	-1.053262402	8.6995299506e-179	6.85363983926e-178	down
YKL020C	74.519661	118.237335	-0.665992641	3.85005571842e-178	3.02929605829e-177	-
YHR050W-A	241.955322	583.976318	-1.271169192	4.32997780827e-178	3.40259521946e-177	down
YOR019W	48.39222	89.995918	-0.895084441	2.35550362295e-177	1.84866833012e-176	-

YIR006C	67.867714	103.979294	-0.61549894	3.99451727498e-177	3.13105596503e-176	-
YGR056W	24.490206	50.781258	-1.052091226	5.64349016981e-177	4.4180059236e-176	down
YNL061W	31.960413	68.864258	-1.107469337	1.03921582252e-176	8.12525418919e-176	down
YOR385W	253.307632	407.472717	-0.685812919	1.24978121474e-176	9.7592978379e-176	-
YKR025W	55.093498	127.197922	-1.207121131	6.66354030364e-176	5.19689173429e-175	down
YDL176W	27.462933	59.350037	-1.111763218	8.28846863351e-176	6.4560618917e-175	down
YFR029W	26.02	57.840496	-1.152458962	8.84158500261e-176	6.87826562609e-175	down
YPL229W	106.424812	222.209412	-1.062085384	1.50607875435e-175	1.17017983817e-174	down
YNL095C	15.495962	41.508144	-1.421502103	2.1505497296e-175	1.66882659017e-174	down
YNL322C	678.571228	944.745178	-0.47742499	2.22746912538e-175	1.72635809368e-174	-
YDL131W	94.791069	169.324921	-0.836971279	2.7214856546e-174	2.10660635209e-173	-
YGR218W	55.127884	91.718163	-0.734425233	6.45675431775e-174	4.99172239161e-173	-
YDL086W	387.837463	300.02652	0.370362137	4.09499651089e-173	3.16190775368e-172	-
YKL101W	7.95849	2.189823	1.861680464	5.01231924285e-173	3.86540097635e-172	up
YPL079W	164.04126	325.571014	-0.98891353	5.02824277782e-173	3.87286987155e-172	-
YOR063W	497.245056	698.662415	-0.490638505	6.10057761212e-173	4.69298461165e-172	-
YOR326W	21.523922	11.890817	0.85609314	6.26437458612e-173	4.81302443448e-172	-
YHR143W	89.407341	46.14233	0.954302436	9.24786317564e-173	7.09650613033e-172	-
YMR103C	222.094742	110.440384	1.007907429	9.5850406353e-172	7.34616447703e-171	up
YGR161C-D	19.882229	11.158415	0.833347393	1.19347597681e-171	9.13575692236e-171	-
YHR161C	198.492279	293.115204	-0.562384913	1.86841931855e-171	1.42846639527e-170	-
YBL092W	328.513794	581.23822	-0.823175623	3.85657674099e-171	2.94484974269e-170	-

YLR281C	144.965042	65.828224	1.138926859	5.79583955801e-171	4.42021768749e-170	up
YDR035W	200.289062	319.954742	-0.675784212	6.07665308264e-171	4.62869476528e-170	-
YBR014C	87.730293	34.358929	1.35239002	1.33169602338e-170	1.01313344524e-169	up
YLR151C	52.329983	20.482668	1.353234142	2.44320643737e-170	1.85647803711e-169	up
YHR102W	33.32996	61.159603	-0.875759456	2.87491895937e-170	2.18184558677e-169	-
YER040W	47.5769	87.918243	-0.885901283	1.27254228643e-169	9.64583945564e-169	-
YDR041W	558.815063	835.780884	-0.580753852	1.56175308759e-169	1.18236136192e-168	-
YLR043C	358.215454	207.090302	0.790567585	1.64431257892e-169	1.24334865894e-168	-
YDR316W-A	0.813328	14.989087	-4.203931418	1.92586396288e-169	1.45447244302e-168	down
YML100W	190.051559	164.907196	0.204736505	3.49346226966e-169	2.63516570717e-168	-
YPR110C	45.533787	104.318947	-1.195991855	1.98586763401e-168	1.49614881941e-167	down
YJR099W	70.268196	26.411285	1.411717365	3.2118095571e-168	2.41683802794e-167	up
YBR160W	55.054222	20.551388	1.421617372	1.16977265031e-167	8.7917053428e-167	up
YOR221C	132.309875	228.136444	-0.785976189	1.3669173776e-167	1.02609710764e-166	-
YOL121C	106.508362	242.992264	-1.189943683	4.89257934008e-167	3.66825272261e-166	down
YDL194W	74.069893	120.357445	-0.700366228	6.34530858033e-167	4.75170997185e-166	-
YJL068C	229.642609	162.435013	0.499527711	1.33671873881e-166	9.99801196452e-166	-
YMR242C	137.165482	276.88031	-1.013344992	3.26864251184e-166	2.44184509188e-165	down
YDR507C	10.83602	3.194516	1.762165691	4.60072655725e-166	3.4328498158e-165	up
YFR031C-A	177.117584	308.222412	-0.799264321	1.01986161848e-165	7.60060135357e-165	-
YIR022W	201.947433	113.598999	0.830029685	1.18843754976e-165	8.8463073248e-165	-
YBR070C	75.823959	30.515461	1.3131134	1.33038528948e-165	9.89105614026e-165	up

YDL082W	68.519081	161.687637	-1.238631665	1.74927232406e-165	1.29898117079e-164	down
YNL199C	53.321121	102.590645	-0.944120165	2.11447659227e-165	1.56829996235e-164	-
YEL002C	94.761604	57.344791	0.724640176	4.52592203529e-165	3.35285489201e-164	-
YNL121C	131.311859	205.577194	-0.646683012	8.05803470719e-165	5.96236942339e-164	-
YNL161W	32.74073	65.120911	-0.992034393	1.61307635205e-164	1.19214023733e-163	-
YDR335W	44.746189	74.864265	-0.742512426	2.47695234142e-164	1.82840905298e-163	-
YGR079W	30.534164	76.419334	-1.323513339	3.32386891587e-164	2.45066249759e-163	down
YLL023C	217.81456	149.798325	0.540078903	4.80601712012e-164	3.53923538335e-163	-
YLL050C	568.500366	420.532074	0.434945439	6.24303698117e-164	4.59203478426e-163	-
YGL003C	81.13839	140.309113	-0.790152133	6.42630767389e-164	4.7212447384e-163	-
YMR199W	38.319679	17.148472	1.160005444	7.80510106351e-163	5.72743113502e-162	up
YNL074C	115.990295	195.246384	-0.751291731	8.9804183612e-163	6.58210592519e-162	-
YKL151C	441.981293	367.382599	0.266702013	1.11357967873e-162	8.1522436858e-162	-
YJR046W	147.296326	111.333382	0.403835213	1.48122727643e-162	1.08309292486e-161	-
YML098W	110.368431	236.434189	-1.099111095	3.66560982018e-162	2.67718891337e-161	down
YGR086C	1182.340698	1108.140869	0.0935045247491	1.23266601941e-161	8.99223342952e-161	-
YMR080C	34.469234	63.526676	-0.882053294	1.67335676952e-161	1.21927216258e-160	-
YIL055C	96.928963	158.612289	-0.710504831	2.16758059686e-161	1.57753110731e-160	-
YLR403W	35.619831	70.764519	-0.99034551	5.43126435146e-161	3.94816031544e-160	-
YBR214W	441.068542	390.562134	0.175450785	6.35834149913e-161	4.61667649434e-160	-
YIL082W-A	20.087198	37.886738	-0.915416597	9.57073473023e-161	6.94101883239e-160	-
YOL157C	17.365582	4.306995	2.011477209	1.23432939755e-160	8.94132660445e-160	up

YDR017C	21.730675	43.787735	-1.010793837	1.49499409596e-159	1.08169269787e-158	down
YLR224W	56.560047	116.626717	-1.044043097	1.82589705068e-159	1.31957728645e-158	down
YJL156C	24.738335	53.730492	-1.118992645	2.57618563752e-159	1.85964656253e-158	down
YGL035C	74.71994	133.777939	-0.840275023	3.16238141996e-159	2.28014678921e-158	-
YPR088C	110.839462	181.323593	-0.710095041	4.17041156224e-159	3.00347041512e-158	-
YDL072C	543.829956	431.213379	0.334753682	4.28684778877e-159	3.08374867586e-158	-
YPL237W	82.855835	165.290863	-0.996331771	4.48342391239e-159	3.22142310742e-158	-
YJR005W	49.948441	90.82962	-0.862723195	6.34620591478e-159	4.55459495017e-158	-
YAL060W	499.510406	431.998352	0.209488924	7.5193209013e-159	5.39029378236e-158	-
YLR350W	845.835632	1182.43103	-0.483306793	4.51409926479e-157	3.23224085765e-156	-
YGL139W	21.728148	46.875511	-1.109269206	8.02074691357e-157	5.73649733174e-156	down
YHL016C	11.907893	32.046322	-1.428240623	1.78622170334e-156	1.27604883019e-155	down
YGL252C	74.540443	128.886154	-0.790001988	1.89857119723e-156	1.35475057384e-155	-
YBR012W-A	1.785815	16.977598	-3.248977822	3.88603799948e-156	2.76975016083e-155	down
YGL114W	29.688723	60.399899	-1.024631097	4.13653071303e-156	2.94490626909e-155	down
YOL109W	4058.437744	3861.125488	0.0719030397343	4.5623906795e-156	3.24436670542e-155	-
YOR107W	15.384793	52.71096	-1.776597935	7.30629028128e-156	5.18963959567e-155	down
YER122C	54.550114	104.79969	-0.941980333	1.72657235791e-155	1.22497842262e-154	-
YOR276W	138.289856	278.168243	-1.00826239	3.04008668387e-155	2.15443585998e-154	down
YGL120C	15.256704	37.042439	-1.279735772	3.28330408319e-155	2.32414501123e-154	down
YPR161C	32.135883	65.577888	-1.029024325	5.06521551506e-155	3.58141889721e-154	down
YPR069C	84.246735	42.394196	0.99075401	5.17923331497e-155	3.65787035487e-154	-

YOR283W	80.525841	170.108521	-1.078931682	6.01778801111e-155	4.24527590602e-154	down
YDL127W	40.492458	12.877825	1.652764271	8.58103733062e-155	6.04666058439e-154	up
YMR299C	94.728111	51.837959	0.869783699	9.19216919887e-155	6.46995310505e-154	-
YCL025C	325.195221	448.320099	-0.463223123	1.21725218392e-154	8.55798590918e-154	-
YGR210C	88.220207	52.482155	0.749282185	4.9123863739e-154	3.44978445805e-153	-
YOR023C	170.234482	256.894775	-0.593654254	2.35842230677e-153	1.65435996389e-152	-
YLL040C	25.009483	18.638287	0.424205963	3.15774731365e-153	2.21256154889e-152	-
YBL016W	75.901543	145.401398	-0.937840021	4.25081809547e-153	2.97509343142e-152	-
YJL103C	93.004631	152.244659	-0.711017157	4.94863502241e-153	3.45958628594e-152	-
YDL138W	12.411438	32.204231	-1.375579965	5.44211498242e-153	3.80029806646e-152	down
YDR380W	393.425995	353.591644	0.154008103	2.05462655598e-152	1.43315973703e-151	-
YDR372C	90.436333	167.031097	-0.885142321	2.8478816383e-152	1.98424794732e-151	-
YLR131C	25.034611	11.114722	1.171452174	3.99357444344e-152	2.77938454539e-151	up
YLR348C	70.259293	32.760189	1.100745379	5.62183236934e-152	3.90821224512e-151	up
YNL042W	28.311968	69.163406	-1.288596881	9.15304007241e-152	6.3559365514e-151	down
YER032W	11.669754	3.0934	1.915508847	1.10136851397e-151	7.63943657511e-151	up
YMR041C	139.934555	91.34536	0.615348909	1.56571398902e-151	1.08481612097e-150	-
YDR099W	610.258911	521.566406	0.226570508	2.34074213133e-151	1.61999187863e-150	-
YPR037C	226.184097	387.419006	-0.776397234	3.21118958554e-151	2.21994041726e-150	-
YOR116C	8.106234	19.443863	-1.262211147	3.61417906894e-151	2.49575346607e-150	down
YMR009W	276.259338	463.276154	-0.745849194	5.97625959726e-151	4.12229106442e-150	-
YDR064W	248.102509	439.13858	-0.823739961	2.98055451297e-150	2.053638448e-149	-

YPL259C	77.865784	138.178375	-0.827470431	5.05019342271e-150	3.47578722485e-149	-
YJL076W	18.249598	36.647144	-1.005836085	7.48424339214e-150	5.14531372962e-149	down
YOL082W	153.239624	245.404495	-0.679372284	3.72170933566e-149	2.55579331369e-148	-
YPL213W	98.781944	194.021332	-0.973896015	5.40617093163e-149	3.70845404901e-148	-
YNL076W	35.196411	71.525024	-1.023019753	9.74141865615e-149	6.67491468183e-148	down
YDR454C	154.381271	85.155426	0.858327376	1.12089228254e-148	7.6719948071e-148	-
YER010C	158.886871	96.733948	0.715905732	2.22242746317e-148	1.51947463561e-147	-
YLR266C	36.842064	70.670937	-0.93976315	2.67629041671e-148	1.82776797656e-147	-
YDL173W	469.145325	672.401428	-0.519287899	2.71789452256e-148	1.85414166989e-147	-
YDR481C	104.220924	168.833969	-0.69596025	5.04793693154e-148	3.43991135796e-147	-
YGL135W	231.414307	385.411285	-0.735920754	1.19676794055e-147	8.14642036728e-147	-
YJL055W	267.422058	425.239471	-0.669157046	2.09880847149e-147	1.42709780843e-146	-
YOL061W	25.286758	59.051178	-1.223583758	2.38735541662e-147	1.62152105321e-146	down
YDL128W	125.661118	85.147034	0.56151014	3.9364289719e-147	2.67074874946e-146	-
YOR383C	147.452393	272.248138	-0.884672946	7.29144206093e-147	4.9416236151e-146	-
YMR047C	27.533312	50.62867	-0.878776422	1.17411342582e-146	7.94863265814e-146	-
YMR061W	78.64267	129.471329	-0.719248445	1.53408577231e-146	1.03742968132e-145	-
YOL088C	90.59243	47.211266	0.940259332	1.78769274623e-146	1.20761660159e-145	-
YLR214W	23.358177	50.565243	-1.114218378	4.40973622383e-146	2.97561331278e-145	down
YOL013C	43.420238	84.474251	-0.96014402	9.96734088942e-146	6.7184855854e-145	-
YGR019W	138.448898	100.026505	0.468971235	1.46885046119e-145	9.89004735693e-145	-
YNL240C	116.948486	189.707428	-0.697902984	2.96102039896e-145	1.99155088155e-144	-

YDR244W	72.800919	123.642616	-0.764147516	3.20383459817e-145	2.15253302872e-144	-
YBR109C	455.563934	331.814728	0.457275613	6.93691089704e-145	4.65560463231e-144	-
YDR188W	164.960876	249.039154	-0.594248682	5.81616592257e-144	3.89921793167e-143	-
YIL146C	55.605	102.662842	-0.884627583	2.05977010692e-143	1.37940159911e-142	-
YGL005C	221.502808	158.600159	0.481930771	3.65791909866e-143	2.44702174186e-142	-
YFR017C	914.557495	795.221313	0.201717446	4.60800781556e-143	3.07928014198e-142	-
YIL125W	392.766174	508.019226	-0.37121241	8.51696356739e-143	5.68530213187e-142	-
YML113W	42.769073	104.201508	-1.284736313	1.19312483216e-142	7.9558742836e-142	down
YGL186C	28.102045	60.089035	-1.096426633	4.3483270572e-142	2.89639639175e-141	down
YML110C	254.562851	192.782639	0.401046763	6.99115094715e-142	4.65177546408e-141	-
YPR098C	365.322723	258.990173	0.496274134	1.01733702746e-141	6.76191463222e-141	-
YDL208W	78.680199	182.481079	-1.213674371	1.02907369527e-141	6.83260909119e-141	down
YGR009C	65.176155	111.176376	-0.770434111	1.95812585892e-141	1.2987227919e-140	-
YLR053C	44.865952	145.788055	-1.70017959	2.92068915718e-141	1.93507345654e-140	down
YKR084C	30.383888	62.531372	-1.041273679	4.37218834576e-141	2.89366154056e-140	down
YFL022C	22.593178	53.669163	-1.248206171	5.27641890812e-141	3.48839282019e-140	down
YBR273C	111.423363	185.163681	-0.732749383	5.56371883274e-141	3.67442196954e-140	-
YEL009C	1423.592773	1838.730347	-0.369173408	6.6168634005e-141	4.36530159302e-140	-
YER053C-A	3473.378418	2895.186035	0.262683544	2.18344003074e-140	1.43893797355e-139	-
YNL054W-B	10.343207	4.64207	1.155843392	3.34495577894e-140	2.20206632828e-139	up
YDR398W	20.805059	46.876728	-1.171937493	7.00152642849e-140	4.60439365128e-139	down
YMR196W	69.796661	52.687782	0.405689573	7.98835143523e-140	5.24779742962e-139	-

YLR045C	26.454399	13.659554	0.953597264	9.34369618996e-140	6.13167716144e-139	-
YDR090C	113.588539	200.516861	-0.819906279	1.56255754679e-139	1.02432494725e-138	-
YLR181C	86.125862	48.513016	0.828074644	3.59869691096e-139	2.35661502355e-138	-
YCR088W	70.736427	45.784393	0.627597453	3.93679059278e-139	2.57529989463e-138	-
YOR156C	10.929957	29.170336	-1.416214279	8.42428985293e-139	5.50505172705e-138	down
YMR291W	69.24942	118.618683	-0.776457366	2.70799958909e-138	1.76774568339e-137	-
YER030W	174.492813	96.066772	0.861058199	3.5459873356e-138	2.31234132137e-137	-
YPR073C	109.769737	49.162971	1.158836357	4.84777849945e-138	3.15792328695e-137	up
YGR262C	57.146763	123.951126	-1.117027691	3.18859839315e-137	2.07492859797e-136	down
YOR048C	15.878069	33.780231	-1.089143723	4.63038598406e-137	3.00999331823e-136	down
YER179W	24.386101	5.559201	2.133109659	4.91322715161e-137	3.19051403318e-136	up
YMR262W	52.552544	22.630589	1.215486471	8.24980369285e-137	5.3515967947e-136	up
YKL100C	102.793655	74.003777	0.474080406	1.07879173301e-136	6.99075060386e-136	-
YNL197C	37.739594	71.567062	-0.923216852	1.09157388711e-136	7.06620510032e-136	-
YFR047C	175.776016	121.783279	0.52942217	1.71059980276e-136	1.10618787245e-135	-
YBR198C	28.435602	55.089867	-0.954088633	6.39864242172e-136	4.13348305453e-135	-
YOR264W	113.240906	77.230377	0.552154878	1.01755969892e-135	6.56653909657e-135	-
YEL054C	133.996017	260.885132	-0.961224609	1.1350347979e-135	7.31702598686e-135	-
YDR001C	190.90712	269.708649	-0.498530877	1.23870368906e-135	7.97704616357e-135	-
YJR093C	52.784004	109.51844	-1.053001104	2.03437902182e-135	1.30874870129e-134	down
YCR037C	20.743336	41.899029	-1.014268879	2.47092823909e-135	1.58794228864e-134	down
YPR016C	135.121948	239.927658	-0.828337444	3.68682722505e-135	2.36688970146e-134	-

YOL051W	72.910522	111.177849	-0.608670439	4.29824575772e-135	2.7565609157e-134	-
YOL127W	500.81488	775.255005	-0.630393507	5.06853549785e-135	3.24721035817e-134	-
YMR076C	7.893507	2.343369	1.75208227	8.62963898446e-135	5.52296895005e-134	up
YDL101C	21.255386	6.803976	1.643378501	1.7123964692e-134	1.09480507526e-133	up
YPL177C	173.099701	280.774658	-0.697809495	3.42551496852e-134	2.18781861364e-133	-
YGR284C	165.953156	115.280762	0.525624289	3.69196415877e-134	2.35557178804e-133	-
YDL233W	160.624725	247.056717	-0.621148296	8.56937632674e-134	5.46187764234e-133	-
YPR158W	65.250084	28.509306	1.194546839	9.45126251312e-134	6.01778848015e-133	up
YJR059W	85.185959	132.090225	-0.632836148	1.18523695308e-133	7.53888422613e-133	-
YDR274C	15.757957	77.361198	-2.295529633	2.15623244886e-133	1.3701014373 1e-132	down
YMR153W	58.784966	107.998672	-0.877494428	2.53847502242e-133	1.61133465636e-132	-
YKR007W	85.539909	181.041199	-1.081648467	6.41274386257e-133	4.06642634309e-132	down
YOR164C	101.522255	60.758739	0.740632186	1.15439521102e-132	7.31274027552e-132	-
YIL036W	111.37352	174.172302	-0.645108955	1.86689600843e-132	1.18141594499e-131	-
YKR094C	303.249329	519.239868	-0.775896707	2.74646112215e-132	1.73625566663e-131	-
YJL128C	28.745928	57.649609	-1.003953217	3.68012494593e-132	2.32413180715e-131	down
YLR256W	32.989193	53.996101	-0.710861749	4.31929153245e-132	2.72501644649e-131	-
YHL034C	803.09845	729.138672	0.139383634	7.92194030168e-132	4.9928330348e-131	-
YPL277C	36.273289	16.696634	1.119350276	2.63940651686e-131	1.66180889013e-130	up
YPR023C	275.702789	400.057739	-0.537094373	2.66413279206e-131	1.67567744408e-130	-
YNL049C	21.067944	42.609798	-1.016135682	2.90506536408e-131	1.82536900609e-130	down
YOR070C	42.500896	78.355522	-0.882541694	2.99009895419e-131	1.87689932332e-130	-

YML067C	104.351677	180.310181	-0.789027073	6.12558276515e-131	3.84117351576e-130	-
YJR006W	49.983467	27.070333	0.88473834	9.02825662827e-131	5.6556424973e-130	-
YKL064W	73.399109	113.076859	-0.623469259	1.02206876113e-130	6.39617224709e-130	-
YJR002W	41.026936	77.377159	-0.915336345	2.82582781525e-130	1.76664039044e-129	-
YFL009W	27.024796	52.74387	-0.964719703	4.68864571325e-130	2.92828094445e-129	-
YKR062W	97.062859	172.380661	-0.82860667	5.25363790262e-130	3.27784764819e-129	-
YOL038W	214.074341	152.269592	0.491484018	1.732530065e-129	1.079874161e-128	-
YGR085C	174.629471	312.763428	-0.840774772	2.14889478191e-129	1.33804802469e-128	-
YGL228W	158.308945	234.961029	-0.569678714	3.40446380628e-129	2.11772658411e-128	-
YDR169C	122.017097	191.283981	-0.648632749	7.01759700048e-129	4.360885103e-128	-
YAL025C	23.613821	63.344795	-1.423594573	1.12019545482e-128	6.95417338355e-128	down
YHL007C	16.551632	34.851398	-1.074243054	1.61546462166e-128	1.00187855857e-127	down
YBR132C	68.360374	115.319565	-0.754405105	2.6211432726e-128	1.62395782797e-127	-
YGR248W	504.209167	425.209351	0.245849024	2.67268051366e-128	1.65423735083e-127	-
YJL041W	49.409542	83.372978	-0.754790185	3.2138249892e-128	1.98719377819e-127	-
YPL234C	258.59082	172.490356	0.584155358	4.10721168153e-128	2.53707165363e-127	-
YGL009C	26.542692	51.830708	-0.965492402	4.45739992773e-128	2.75064997528e-127	-
YML081C-A	1866.364014	1539.729004	0.27755394	1.05467990668e-127	6.50193928569e-127	-
YKR096W	63.132641	96.067551	-0.605663106	1.60423358739e-127	9.88004177628e-127	-
YHR186C	8.771145	19.108652	-1.123388917	1.91316890193e-127	1.17710134224e-126	down
YLR148W	21.596664	10.750988	1.006339231	2.32773770392e-127	1.43075204613e-126	up
YDL074C	28.358036	55.844097	-0.97764717	3.13791468899e-127	1.9268223926e-126	-

YMR042W	103.025864	207.034195	-1.006862509	5.20334314641e-127	3.19193223843e-126	down
YPL268W	47.859612	80.244431	-0.745592571	9.0618764173e-127	5.55341844014e-126	-
YGL140C	16.1028	31.768997	-0.980307972	1.09509338918e-126	6.70447708089e-126	-
YDR423C	110.809982	182.8172	-0.722313962	1.47451556603e-126	9.01851491024e-126	-
YOR181W	33.998878	65.548317	-0.947071602	1.93731449927e-126	1.18374492239e-125	-
YLR454W	20.412529	14.555512	0.487889347	2.15311045946e-126	1.31430774163e-125	-
YGL025C	61.292919	114.680336	-0.903825719	4.23472860156e-126	2.58243567372e-125	-
YLL027W	257.727356	400.630646	-0.636427106	6.35745595101e-126	3.87311938605e-125	-
YNL137C	41.111065	80.535133	-0.970091542	1.86923449517e-125	1.13766742608e-124	-
YLR136C	6.43013	31.5226	-2.293466724	2.29446851889e-125	1.39510877231e-124	down
YOR101W	31.20393	75.147377	-1.267995006	2.66933706726e-125	1.62145249643e-124	down
YDR373W	115.89669	221.433929	-0.934036931	4.90867674859e-125	2.9787942576e-124	-
YKL026C	345.666626	251.879181	0.456649437	6.68525748357e-125	4.05293734941e-124	-
YBR021W	4.631936	17.854866	-1.946630082	7.68876250958e-125	4.6567646497e-124	down
YLR040C	46.402721	108.941101	-1.231267042	9.20928728858e-125	5.57224712354e-124	down
YGL014W	42.560043	72.404381	-0.766577385	1.89521668003e-124	1.14561880717e-123	-
YDL204W	530.020752	477.71405	0.149901538	2.38223098789e-124	1.43860797401e-123	-
YBL029C-A	763.482483	584.837891	0.384558272	3.03325160187e-124	1.8299733668e-123	-
YDR026C	17.708757	41.910275	-1.242841037	3.28335889973e-124	1.97894097568e-123	down
snR9	156.391312	48.494621	1.689263731	8.05328071912e-124	4.84915292961e-123	up
YDL083C	135.578339	266.61203	-0.975615178	8.91840440789e-124	5.36486962831e-123	-
YFR001W	56.212299	20.210623	1.475772026	9.61873187152e-124	5.78055057681e-123	up

YBL105C	47.094387	75.0513	-0.672321941	1.20647562782e-123	7.24352098407e-123	-
YPL235W	63.356411	112.835587	-0.832659631	1.24534666153e-123	7.46967350217e-123	-
YML064C	48.942188	18.217699	1.42573785	1.28703606649e-123	7.71227789651e-123	up
YOR382W	38.343159	109.315613	-1.511458361	1.78091933019e-123	1.06614727115e-122	down
YNL210W	12.042791	43.943844	-1.86749129	2.46601079253e-123	1.47485500964e-122	down
YKR067W	104.591499	81.440392	0.360949186	2.48801780173e-123	1.48658464996e-122	-
YMR302C	98.315529	146.738815	-0.577759325	3.23358036886e-123	1.93019874326e-122	-
YNL317W	41.579033	81.726791	-0.974952882	4.07859636691e-123	2.43226957212e-122	-
YPL050C	38.015537	79.808861	-1.069959764	4.29224275585e-123	2.55722101999e-122	down
YKR075C	342.612671	496.123199	-0.534119912	4.82701424511e-123	2.87306849795e-122	-
YBR179C	52.1413	85.808289	-0.718690464	9.62579861589e-123	5.72384653328e-122	-
YBR162W-A	270.208771	542.409119	-1.005306928	1.15934980664e-122	6.88731444939e-122	down
YAL021C	43.17907	73.876617	-0.774785633	1.31684554192e-122	7.81546570198e-122	-
YPL090C	185.454636	306.042023	-0.722663432	1.84495180344e-122	1.0939313081e-121	-
YKL195W	282.17627	235.27919	0.262222945	2.86344296545e-122	1.69620743602e-121	-
YOR113W	71.537292	110.433807	-0.626414478	5.94175461197e-122	3.51634057494e-121	-
YHR214C-B	14.384871	8.219868	0.807365152	6.76834960138e-122	4.00170612623e-121	-
YGL130W	64.381432	114.504013	-0.83068159	1.32943766157e-121	7.85266318078e-121	-
YDR294C	139.492493	110.102478	0.341340544	1.48523473838e-121	8.76457914057e-121	-
YMR054W	20.874866	41.313164	-0.984834618	1.64318456559e-121	9.68745468489e-121	-
YMR265C	41.74094	81.854851	-0.971604828	2.47313625289e-121	1.45666317438e-120	-
YFR018C	65.130669	36.009521	0.854958637	2.83822700841e-121	1.67011500172e-120	-

YHR132C	207.472412	306.246429	-0.56177351	2.96600200158e-121	1.74364966153e-120	-
YOR201C	25.847973	59.694435	-1.207545294	3.87749625163e-121	2.27734122328e-120	down
YGL253W	85.705948	142.372314	-0.732201388	7.06953624186e-121	4.14817400657e-120	-
YLR336C	12.581356	28.503975	-1.179875701	7.18025949881e-121	4.20916439742e-120	down
YDR349C	46.682865	83.961761	-0.846839322	1.3118964328e-120	7.68325759887e-120	-
YLR187W	10.80049	24.609249	-1.188103865	1.36327459496e-120	7.97663401088e-120	down
YER003C	52.949486	28.854494	0.875820648	2.35966389349e-120	1.37935908199e-119	-
YNL094W	67.138428	112.569962	-0.745611249	2.73595364113e-120	1.59781751685e-119	-
YJL149W	26.681627	53.30547	-0.998436942	3.6235915376e-120	2.11421581442e-119	-
YLR340W	238.145142	359.953796	-0.595970614	4.04559530665e-120	2.35822118908e-119	-
YOL078W	12.951781	26.910973	-1.055044058	4.6247906056e-120	2.69331145212e-119	down
YDR160W	21.31435	42.296013	-0.988696616	4.63136688266e-120	2.694613459e-119	-
YJL177W	119.01973	225.518311	-0.922043828	5.41578995473e-120	3.1480546853e-119	-
YLR261C	1.605687	40.54314	-4.65819523	5.4694909876e-120	3.17629560814e-119	down
YGR027W-B	7.092714	2.780259	1.351118489	1.71650571687e-119	9.95894157975e-119	up
YOR234C	69.46389	179.494324	-1.369603114	2.32940978535e-119	1.3502311809e-118	down
YOR244W	71.436249	124.608398	-0.802673068	4.58818630402e-119	2.65703923278e-118	-
YOR044W	45.448997	118.982826	-1.388432988	5.43351476017e-119	3.14364022657e-118	down
YKL006W	73.022461	170.557602	-1.223846863	5.96811567543e-119	3.44972645373e-118	down
YKL022C	35.509159	62.738815	-0.821167088	6.88235532589e-119	3.97448017331e-118	-
YHR158C	22.137941	40.343098	-0.865800828	8.34734556331e-119	4.81601498671e-118	-
YNL312W	54.409023	24.011194	1.180138777	9.7773479714e-119	5.63581951778e-118	up

YIL133C	141.672989	253.076096	-0.837006522	1.44879054519e-118	8.34331326953e-118	-
YLR180W	8.957508	31.331587	-1.806448515	4.50032276108e-118	2.58924964789e-117	down
YER086W	29.94211	59.921349	-1.000894213	4.64614850626e-118	2.67067499323e-117	down
YOL040C	370.980682	588.634338	-0.666027641	1.38577286837e-117	7.95825898878e-117	-
YOR191W	32.913624	52.157219	-0.664182063	1.70385032863e-117	9.77588062861e-117	-
YBR129C	80.131889	145.01445	-0.855748273	1.85570451134e-117	1.06373163494e-116	-
YKL184W	24.176853	54.31929	-1.167838155	3.03510265338e-117	1.7381842502e-116	down
YER152C	66.103798	40.244907	0.715926943	3.14396660417e-117	1.7988704773e-116	-
YDR265W	78.791138	142.162537	-0.851436055	4.92240602345e-117	2.81383946534e-116	-
YMR296C	35.85865	68.996513	-0.944202277	6.86567759374e-117	3.92107879503e-116	-
YLL052C	26.190056	86.046051	-1.716089838	7.58768218833e-117	4.32944218981e-116	down
YGR295C	122.486595	86.054924	0.509294219	8.34985071376e-117	4.75995162819e-116	-
YER151C	41.278473	69.413681	-0.749830434	9.60287754771e-117	5.4692352125e-116	-
YJR106W	37.515606	67.293251	-0.842970958	1.64394179455e-116	9.35434524342e-116	-
YOR014W	79.287949	123.11972	-0.634888344	1.90156843889e-116	1.08103817478e-115	-
YDL209C	15.613547	45.296291	-1.536594605	2.46765048542e-116	1.40157129126e-115	down
YOL101C	6.176925	28.308226	-2.196260624	2.97387009165e-116	1.68754895146e-115	down
YLR154C	122.743423	52.002163	1.239002185	6.334438921e-116	3.59125085128e-115	up
YKL109W	583.190369	754.087463	-0.37076497	9.57302415846e-116	5.42238448684e-115	-
YOL064C	60.970875	114.707329	-0.911765417	1.04944048607e-115	5.93885737241e-115	-
YDR043C	203.229401	327.917023	-0.690221667	1.06260018707e-115	6.0078524238e-115	-
YGR202C	70.784874	124.115967	-0.810175715	2.34288852377e-115	1.32344421798e-114	-

YGR094W	24.437065	43.815971	-0.842385809	2.55060280054e-115	1.43946747143e-114	-
YOL067C	182.976761	315.608215	-0.786474328	4.92880817151e-115	2.77911363567e-114	-
YIL162W	414.434387	379.202972	0.128173442	1.72511957648e-114	9.71827797714e-114	-
YDR408C	137.21109	241.153931	-0.813557236	2.17950161595e-114	1.2266859503e-113	-
YNR031C	24.03089	40.191036	-0.741983685	2.87733754974e-114	1.61798111493e-113	-
YKR021W	27.28245	49.637978	-0.863471142	3.17360059144e-114	1.78296040467e-113	-
YMR310C	21.137428	55.782322	-1.400008149	4.18676746723e-114	2.35004090747e-113	down
YEL039C	606.457214	468.351501	0.372814177	4.3692864778e-114	2.45027375377e-113	-
YOR372C	78.17598	54.200974	0.528406622	5.14301171753e-114	2.88157190816e-113	-
YKL081W	51.7999	97.488708	-0.91228581	1.40353142838e-113	7.85673859998e-113	-
YGL144C	9.741606	25.702436	-1.399673561	1.44501737861e-113	8.08168278053e-113	down
YMR169C	272.468262	378.901031	-0.475732876	3.28344819285e-113	1.83471164547e-112	-
YMR049C	13.513234	30.337854	-1.166746055	4.18469922773e-113	2.33620618757e-112	down
YMR215W	23.617453	9.607038	1.297689788	1.17152915325e-112	6.53445910457e-112	up
YOR316C	24.888596	55.718746	-1.162677941	2.11460679017e-112	1.17840924178e-111	down
YMR227C	22.879807	48.234955	-1.076004138	2.18716146875e-112	1.21774873525e-111	down
YDR291W	12.839529	26.88377	-1.066143186	2.32602939659e-112	1.29390595825e-111	down
YOR032C	72.334282	124.938278	-0.788464088	4.94130734856e-112	2.74625210563e-111	-
YLR086W	6.497578	2.112183	1.621167212	6.65871930298e-112	3.69743554856e-111	up
YIR011C	128.831314	211.496307	-0.715149172	7.72288744229e-112	4.28451163912e-111	-
YGR116W	39.994209	62.012894	-0.6327771	1.52330752122e-111	8.44347597478e-111	-
YPL151C	81.457672	54.286018	0.585469921	2.53084008887e-111	1.40155711612e-110	-

YLR413W	2.857776	12.803651	-2.163590514	2.8418360556e-111	1.5723813042 1e-110	down
YGL048C	343.773834	299.184692	0.200423379	3.33857748803e-111	1.84558228368e-110	-
YPR009W	27.548281	69.541092	-1.335903424	9.12998503945e-111	5.04261095417e-110	down
YLR258W	173.652313	148.565781	0.225099767	1.00514533692e-110	5.54661533475e-110	-
YDR422C	35.82753	62.64423	-0.806113047	1.02154654853e-110	5.63211454108e-110	-
YBR056W-A	716.280762	1144.108765	-0.675627109	1.27500867483e-110	7.02329534459e-110	-
YPR119W	18.442858	5.973199	1.626486549	2.02271786793e-110	1.1132121032e-109	up
YBR080C	20.29101	41.10157	-1.018352825	2.3310100907e-110	1.2817458497e-109	down
YMR086W	56.378635	88.194901	-0.6455467	3.74049882701e-110	2.05495723169e-109	-
YLR246W	67.458092	122.493698	-0.860644109	5.44077483134e-110	2.98641292245e-109	-
YHR172W	10.486588	3.277149	1.678032174	8.83716030232e-110	4.8463861446e-109	up
YBR045C	16.584642	6.16538	1.427586148	1.65833743227e-109	9.08645964652e-109	up
YGL156W	136.980484	188.415039	-0.45994376	1.81613114228e-109	9.94227701171e-109	-
YIR003W	66.565041	107.067024	-0.685677608	2.99562644176e-109	1.63848889431e-108	-
YJL020C	87.835655	125.88015	-0.519172208	3.86762311886e-109	2.11357432411e-108	-
YLL008W	9.294355	23.778164	-1.355210668	6.60548105395e-109	3.60658103632e-108	down
YDL184C	980.727966	580.503601	0.756548001	1.02075737757e-108	5.56841985936e-108	-
YNR053C	24.494928	52.980564	-1.112980151	1.0948295213e-108	5.96725344004e-108	down
YPL063W	89.875114	145.736237	-0.697366044	1.60787317462e-108	8.75585672637e-108	-
YDR309C	52.23716	28.213507	0.888690456	2.28360588523e-108	1.24247373668e-107	-
YLR399C	63.975803	103.251114	-0.690559092	3.32386498046e-108	1.80687861635e-107	-
YNL012W	31.773432	17.185631	0.886618107	3.45864290012e-108	1.87850001084e-107	-

YPL105C	30.982903	55.06638	-0.829699444	5.00139974412e-108	2.71404629471e-107	-
YGR124W	104.828461	160.913528	-0.618255156	6.18602038796e-108	3.35395760423e-107	-
YMR307W	144.329056	116.082848	0.314206949	6.50588280581e-108	3.52430370493e-107	-
YGL071W	13.496458	31.319735	-1.214491169	8.94578095081e-108	4.84179669945e-107	down
YER110C	13.869023	27.895136	-1.008147424	9.15460775559e-108	4.95050565738e-107	down
YIR039C	52.146481	32.221504	0.694546063	1.22133391052e-107	6.59881715973e-107	-
YDR204W	231.213593	184.750244	0.323649946	1.50302667524e-107	8.11373008685e-107	-
YBL099W	572.309875	543.93512	0.0733619216685	1.5847039575e-107	8.54721300449e-107	-
YLR330W	176.151031	247.659882	-0.491547269	1.79351333362e-107	9.6650440756e-107	-
YOL068C	93.258553	148.646576	-0.672578282	1.88585516706e-107	1.01538498501e-106	-
YDL031W	17.451756	34.037617	-0.963757828	2.33049993217e-107	1.25370394964e-106	-
YDL046W	855.56073	754.225342	0.181874639	3.7568307978e-107	2.01925589548e-106	-
YKR043C	55.495884	112.115578	-1.014534069	5.26900443352e-107	2.82958300374e-106	down
YML092C	365.751282	301.048584	0.28086659	5.59526987274e-107	3.0021983898e-106	-
YLR247C	24.470272	40.399284	-0.723299586	6.55341884342e-107	3.51326633678e-106	-
YPL220W	124.142166	218.930511	-0.818479805	7.9615926319e-107	4.26450104045e-106	-
YPR015C	35.402279	11.506061	1.621448212	8.77267489001e-107	4.69489359631e-106	up
YOR304W	19.007221	35.275269	-0.89210947	1.06506285791e-106	5.69501311103e-106	-
TLC1	49.118622	27.414257	0.841343714	1.11039571761e-106	5.93230345519e-106	-
YER017C	48.591763	80.605385	-0.730164447	1.13261760161e-106	6.04582121309e-106	-
YMR304W	54.112667	42.088116	0.362553416	2.05301396724e-106	1.09494078253e-105	-
YER161C	125.21489	203.199646	-0.698491757	2.57133169285e-106	1.3701997553e-105	-

YDR497C	202.185059	173.149429	0.223658759	3.85339120674e-106	2.05161686205e-105	-
YPL016W	15.726603	8.691789	0.85548202	4.11730378413e-106	2.19025037634e-105	-
YBL037W	10.565194	23.316771	-1.142048754	4.42757769641e-106	2.35328787152e-105	down
YLR253W	21.436291	45.702324	-1.092212223	7.15071858342e-106	3.79740470195e-105	down
YNL064C	263.082397	222.459915	0.24196932	1.03705008297e-105	5.50257001286e-105	-
YKL196C	300.469086	459.312592	-0.61225977	1.77556811465e-105	9.41308868979e-105	-
YLR448W	66.003685	142.401321	-1.109344052	3.02918610507e-105	1.60453816897e-104	down
YMR026C	66.444305	117.214012	-0.818927588	3.20790811516e-105	1.69775733835e-104	-
YBR172C	17.408461	36.489304	-1.067684966	3.51032334254e-105	1.85622549493e-104	down
YHR091C	21.481819	44.170517	-1.039967558	4.62611733283e-105	2.44416480019e-104	down
YJR061W	14.67155	30.215954	-1.042289198	7.60272810587e-105	4.01341293208e-104	down
YOR220W	1095.937256	1417.529785	-0.371213845	8.15226161824e-105	4.299850478e-104	-
YLR407W	34.723675	83.961792	-1.273813314	1.15729101757e-104	6.09886471739e-104	down
YPR187W	95.572441	192.618027	-1.011076159	1.6408642677e-104	8.63993670387e-104	down
YGR245C	16.317379	34.393166	-1.075712585	2.6708940909e-104	1.40516190816e-103	down
YCL035C	1005.663635	856.121643	0.232260143	2.88029823365e-104	1.51404669217e-103	-
YGR197C	34.716228	65.779518	-0.922028233	4.11661132171e-104	2.16209163157e-103	-
YBR265W	93.780518	160.985153	-0.779567488	5.31793913486e-104	2.79068183848e-103	-
YDR345C	23.135124	48.086159	-1.055536861	8.45001077858e-104	4.43054619201e-103	down
YGL148W	61.893768	111.89016	-0.854217108	1.02480299819e-103	5.36875697281e-103	-
YOL155C	176.574188	238.342926	-0.432764345	1.12070865378e-103	5.86623888929e-103	-
YDR304C	222.9505	164.326187	0.440161029	1.61733291171e-103	8.45863750286e-103	-

YLR314C	59.792439	38.747467	0.62586106	2.00332265378e-103	1.04685412749e-102	-
YPL030W	9.191241	25.615688	-1.478696069	2.74327780958e-103	1.43231864103e-102	down
YMR006C	41.360661	71.309326	-0.785831526	2.82676366245e-103	1.47466796777e-102	-
YNL180C	190.777557	288.676025	-0.597559831	3.29922940796e-103	1.71969909023e-102	-
YPL160W	12.692294	25.962513	-1.032477189	3.73005526028e-103	1.9426328067e-102	down
YNL107W	61.197788	125.103714	-1.031573207	3.98735155196e-103	2.07489341447e-102	down
YDR262W	761.214478	1009.617188	-0.40743347	4.01741638782e-103	2.08878734804e-102	-
YPR174C	32.579994	9.076897	1.843715247	4.7765409404e-103	2.48140302577e-102	up
YOL143C	408.603149	322.330505	0.342159593	5.42502191856e-103	2.81593110956e-102	-
YBR212W	151.169022	215.120224	-0.508980634	6.77238204402e-103	3.51235987713e-102	-
YLR192C	86.483261	156.444611	-0.855159135	1.4225966509e-102	7.3718530958e-102	-
YJR111C	38.919693	84.566231	-1.119581351	1.65018148555e-102	8.54405893436e-102	down
YGR026W	103.738907	65.856125	0.655567544	1.93708725266e-102	1.00211980538e-101	-
YNL229C	83.25988	142.933868	-0.779654417	3.18293278811e-102	1.64526617391e-101	-
YPL145C	72.975571	123.559509	-0.759720544	3.81437834613e-102	1.9700216949e-101	-
YCR091W	79.104195	60.151043	0.395164451	6.05308460371e-102	3.12365330173e-101	-
YLR138W	47.865711	75.572906	-0.65887656	6.34053563321e-102	3.26927285806e-101	-
YOR251C	86.557037	53.383316	0.69726219	9.84490838517e-102	5.07196607927e-101	-
YDL180W	53.83873	92.303513	-0.777741178	2.16892937946e-101	1.11647708024e-100	-
YBR263W	134.603058	105.543213	0.350877378	2.32179903077e-101	1.19417799362e-100	-
YLR197W	24.794643	51.863316	-1.064685998	2.89552913238e-101	1.48803351439e-100	down
YJL173C	96.88903	41.237911	1.232362078	5.12227061421e-101	2.63019486956e-100	up

YDR177W	196.72522	140.031311	0.490432472	5.80494910276e-101	2.97827471322e-100	-
YPL010W	130.713776	79.152168	0.723710424	6.5460763214e-101	3.35574251059e-100	-
YNL252C	61.39138	118.036507	-0.943125128	8.04215859767e-101	4.11928387577e-100	-
YGR204W	149.37764	204.738098	-0.454815376	8.84648059015e-101	4.52753103905e-100	-
YLR064W	83.046127	48.711594	0.769647694	1.05707551495e-100	5.40553937136e-100	-
YDR471W	147.634689	273.826416	-0.891229886	1.34080616325e-100	6.85080219052e-100	-
YJL197W	9.779561	20.521713	-1.069309552	1.53651881629e-100	7.84433290419e-100	down
YNL100W	405.874542	339.492157	0.257655606	1.70032887351e-100	8.67349354704e-100	-
YOL020W	26.410267	51.983318	-0.976949835	1.76027128609e-100	8.97189174386e-100	-
YCR028C-A	203.899719	128.515182	0.665920986	4.46786833876e-100	2.2753508324e-99	-
YPL064C	75.85424	136.550552	-0.848133416	6.00309105487e-100	3.05468764497e-99	-
YLR182W	24.460901	13.592057	0.847713738	8.26868200917e-100	4.20409319516e-99	-
YKL004W	34.135601	69.361031	-1.022848191	1.06694519197e-99	5.42029112255e-99	down
YER057C	518.217407	409.086884	0.341150194	1.4505924473e-99	7.36326893937e-99	-
YPR183W	149.453781	105.226082	0.506207051	1.68132191827e-99	8.52748894497e-99	-
YLR055C	23.835451	47.821564	-1.004552391	1.86969330901e-99	9.47514780597e-99	down
YDR161W	40.035706	78.755493	-0.976093304	2.01923029451e-99	1.02246180003e-98	-
YIL090W	40.770763	75.47802	-0.888521621	2.14169839568e-99	1.08359116873e-98	-
YKL204W	107.163292	159.734146	-0.575861942	2.59557449952e-99	1.31216013787e-98	-
YOR171C	63.70853	102.975662	-0.692744946	4.03900643205e-99	2.04020764281e-98	-
YMR062C	88.470634	143.439804	-0.697174855	4.22523338834e-99	2.13254055893e-98	-
YKL156W	472.246094	766.29187	-0.698355135	4.63213958438e-99	2.33601320388e-98	-

YHR132W-A	431.458435	329.95694	0.38694382	7.39161882877e-99	3.7246079293e-98	-
YER141W	198.386093	282.670715	-0.510811531	7.53509571619e-99	3.79382596968e-98	-
YKL028W	54.345776	94.836967	-0.803281618	1.1257564075e-98	5.66344876644e-98	-
YKL065C	722.484314	643.83905	0.166266184	1.16565972816e-98	5.85944582384e-98	-
YDR465C	31.864954	65.262955	-1.034293734	1.58564911117e-98	7.96416640952e-98	down
YAL041W	17.574886	34.77121	-0.984377941	2.03315577964e-98	1.02035821181e-97	-
YDL215C	155.72525	140.886505	0.144469461	2.16630496324e-98	1.08630219805e-97	-
YNR023W	27.612577	54.029816	-0.968430232	3.84281093624e-98	1.92543747314e-97	-
YNL232W	70.260139	128.934662	-0.875861825	4.31696155938e-98	2.16126591618e-97	-
YDR207C	20.370306	39.024193	-0.937901146	6.17098854365e-98	3.08698604988e-97	-
YOR172W	25.645638	47.200397	-0.880085532	1.62531896198e-97	8.1239775491e-97	-
YML074C	106.951088	169.509506	-0.664415022	1.7268380262e-97	8.62446537945e-97	-
YOR362C	456.53183	401.290405	0.186068789	1.95734935184e-97	9.76786557576e-97	-
YEL058W	53.55909	90.876984	-0.762783507	2.17513319437e-97	1.08459653579e-96	-
YJR019C	147.372208	110.802872	0.411469205	2.98197354495e-97	1.48572165065e-96	-
YOR167C	432.422058	736.493958	-0.768233572	2.9872490772e-97	1.48715655744e-96	-
YKL046C	65.393829	111.230179	-0.766321869	4.39834454243e-97	2.18789446469e-96	-
YNL237W	44.215771	81.144821	-0.875937973	6.45709666496e-97	3.20942002371e-96	-
YKL211C	42.376507	77.577446	-0.872372606	6.60119781444e-97	3.27841888256e-96	-
YOR371C	22.091328	40.760223	-0.883681797	7.12570715715e-97	3.53608233666e-96	-
YDL125C	694.743225	596.859558	0.219088358	8.3954244091e-97	4.16284302969e-96	-
YLR362W	44.411705	74.353363	-0.743458038	9.96672738708e-97	4.93802423137e-96	-

YMR039C	110.639275	184.684555	-0.739199612	1.01083457364e-96	5.00419540124e-96	-
YKL190W	159.526077	273.708771	-0.778849393	1.29585195684e-96	6.41007884308e-96	-
YDL170W	63.331341	105.035034	-0.72987908	1.41500548477e-96	6.99391245977e-96	-
YNL208W	2800.025879	2791.214355	0.00454723888429	1.58882803981e-96	7.84681342176e-96	-
YMR148W	117.808685	63.161667	0.899324747	1.59900905157e-96	7.89081732287e-96	-
YOR114W	22.802565	6.301934	1.855329568	1.99692397132e-96	9.84662749319e-96	up
YBR104W	49.880978	96.014503	-0.94476259	2.17143169866e-96	1.06986095121e-95	-
YER068W	30.910875	58.075798	-0.909822579	3.74074198723e-96	1.84159605525e-95	-
YMR018W	18.560276	7.079264	1.390546882	1.52925973882e-95	7.52269766926e-95	up
YLR438W	56.008568	99.059959	-0.822654481	1.60430707643e-95	7.88562021415e-95	-
YDR346C	43.614216	79.111176	-0.859083062	4.75967296866e-95	2.33766216689e-94	-
YJL190C	72.023544	160.203568	-1.153365783	7.90700102275e-95	3.88036856516e-94	down
YER164W	10.496545	20.422741	-0.960261973	1.72904463896e-94	8.47860119957e-94	-
YJL092W	11.340468	5.376749	1.076674149	2.57037722523e-94	1.25942397902e-93	up
YIL140W	16.680866	8.04764	1.051556515	3.27006791998e-94	1.60099224348e-93	up
YPL126W	12.649364	26.622746	-1.073594536	3.76552124813e-94	1.84210842462e-93	down
YNL016W	72.01432	119.387894	-0.729300835	4.60420449923e-94	2.25062216781e-93	-
YPL186C	386.156036	336.226654	0.199749825	9.08220492508e-94	4.43606043863e-93	-
YGR027W-A	1.951486	12.465053	-2.675243999	1.01178399419e-93	4.93801496536e-93	down
YOR141C	81.596649	119.732956	-0.553238493	1.04808942629e-93	5.11118551328e-93	-
YBL047C	68.613548	97.347359	-0.50464837	1.23906173423e-93	6.03775137058e-93	-
YNL067W	134.653442	233.181824	-0.79220423	1.61462833786e-93	7.86165703641e-93	-

YLR244C	46.964462	87.607246	-0.899480718	1.69842307068e-93	8.26317431252e-93	-
YMR095C	10.533969	39.144444	-1.893758433	1.83276947531e-93	8.9098143326e-93	down
YDR477W	51.302364	85.017548	-0.728735344	1.96073604697e-93	9.52445178371e-93	-
YGL045W	154.292068	222.054749	-0.52525153	2.03595756633e-93	9.88211459873e-93	-
YKL033W-A	90.676872	53.588768	0.758803976	2.11888279563e-93	1.02765815588e-92	-
YNL139C	19.495745	32.535561	-0.738858144	4.48043189156e-93	2.17131312902e-92	-
YKL049C	73.670448	39.624332	0.894699402	9.46661905378e-93	4.5841475106e-92	-
YNL189W	65.135597	106.191849	-0.705154927	1.24109022862e-92	6.00521289108e-92	-
YJL051W	15.535278	7.278373	1.093860165	1.32055399965e-92	6.38473460268e-92	up
YBR287W	347.289917	469.843475	-0.436039688	1.35881456003e-92	6.56460761766e-92	-
YJR004C	41.667633	26.787727	0.637354977	1.52398386162e-92	7.3568365575e-92	-
YGL078C	21.764648	45.510357	-1.064208216	2.02146465306e-92	9.75077899474e-92	down
YDL201W	23.267204	57.148174	-1.296409548	2.43463488883e-92	1.1734637725e-91	down
YJL139C	51.915035	92.424454	-0.832122201	2.57416522759e-92	1.23975312125e-91	-
YNL224C	17.379972	34.839252	-1.003287892	3.27232383964e-92	1.57477413926e-91	down
YBL098W	24.446493	10.662636	1.197063374	3.28316645699e-92	1.57876819249e-91	up
YKR087C	97.999809	160.759567	-0.714053754	5.75755132515e-92	2.76647667388e-91	-
YOL077C	26.497396	61.965061	-1.225604394	5.84508435707e-92	2.80636378103e-91	down
YPL043W	16.697851	34.819	-1.060212329	6.44687779376e-92	3.09290705902e-91	down
YNR071C	7.459671	26.561888	-1.832173787	8.15181930155e-92	3.90783739182e-91	down
YIR025W	50.774845	93.708817	-0.884070866	1.17518407944e-91	5.62927682495e-91	-
YFL036W	18.681738	32.345921	-0.791955114	1.97080206728e-91	9.43310657953e-91	-

YKR095W	11.108929	6.544434	0.763379405	2.08281923792e-91	9.9615884661e-91	-
YMR194C-B	482.378601	336.360382	0.52015812	2.3027302417e-91	1.10048878679e-90	-
YOR337W	15.146129	31.486965	-1.055805583	2.91347683883e-91	1.39129724734e-90	down
YJR155W	30.415977	68.238777	-1.165762444	3.02102481319e-91	1.44154665952e-90	down
YPR086W	123.004135	193.874802	-0.656418493	4.54959659868e-91	2.16927002186e-90	-
YHL009W-A	0.074681	7.639865	-6.676662089	4.97130880625e-91	2.36852533148e-90	down
YLL049W	52.184059	20.33173	1.359876187	6.86284497808e-91	3.26721944969e-90	up
YDR429C	139.780807	223.90564	-0.679724588	9.5029473759e-91	4.52063580916e-90	-
YBR039W	1191.392822	1162.249146	0.0357298063637	1.04410554704e-90	4.96309895561e-90	-
YGR193C	137.864777	208.001801	-0.59334211	1.16618707959e-90	5.53916556242e-90	-
YMR240C	22.110472	48.144348	-1.122636617	1.41490203904e-90	6.71537603849e-90	down
YDR311W	23.322319	45.204109	-0.954742671	1.72786114238e-90	8.19447056675e-90	-
YPR160W	467.347412	459.864502	0.0232865677569	1.98478880906e-90	9.40577780658e-90	-
YGL134W	83.48613	134.942657	-0.692738034	2.28844215295e-90	1.08364980057e-89	-
YKR058W	95.951233	75.40303	0.347678848	6.25636920265e-90	2.96033079345e-89	-
YJR105W	67.813133	119.108688	-0.812642045	6.64610630092e-90	3.14234789917e-89	-
YCR077C	32.900597	56.336266	-0.775950182	1.05391470059e-89	4.97922561739e-89	-
YNL295W	46.78384	81.091187	-0.793534848	1.88709283738e-89	8.90880025435e-89	-
YBR181C	153.701874	247.868896	-0.68944249	2.08743300287e-89	9.84710036612e-89	-
YKL207W	167.054138	262.64212	-0.652782581	2.19465435373e-89	1.03450373789e-88	-
YGR264C	19.468813	37.835136	-0.958561704	2.26549496746e-89	1.06708594522e-88	-
YGL080W	533.414978	432.750763	0.301721972	6.3974124049e-89	3.01100350338e-88	-

YDR384C	473.101715	420.119446	0.171350825	6.90715675564e-89	3.24845675295e-88	-
YMR229C	7.075162	14.350776	-1.020293664	1.37159262258e-88	6.44575851702e-88	down
YPL154C	567.168884	534.211304	0.0863678816994	1.99119762488e-88	9.35049535195e-88	-
YNL181W	48.496662	28.390125	0.77249625	2.56852285852e-88	1.20524489083e-87	-
YIL084C	31.090158	66.176453	-1.089860018	4.25356041313e-88	1.99441865896e-87	down
YOR025W	24.521372	10.846138	1.17685827	6.13930017152e-88	2.87643588413e-87	up
YML069W	61.412613	99.903305	-0.701997417	6.18310163191e-88	2.89477337337e-87	-
YNL323W	36.144413	69.504005	-0.943323451	1.18711659748e-87	5.55359445149e-87	-
YLR150W	324.235291	459.930481	-0.504374685	1.28719373119e-87	6.01724298438e-87	-
YPR133C	123.819748	188.731232	-0.608091757	1.52534884547e-87	7.12518106297e-87	-
YGL146C	50.864368	96.435913	-0.922915151	1.73585172011e-87	8.10238156272e-87	-
YHR154W	5.226173	1.319224	1.986065332	1.89951102244e-87	8.85962766889e-87	up
YNL152W	20.065735	45.312462	-1.175173878	2.47330726447e-87	1.15272458692e-86	down
YER143W	215.627121	183.479691	0.232918265	3.10583901217e-87	1.44644025413e-86	-
YNL134C	823.811951	1047.458618	-0.346506288	3.35061817005e-87	1.55926818588e-86	-
YER095W	54.952427	33.625999	0.70860607	3.36769163465e-87	1.5660396755e-86	-
YGR148C	168.633102	287.018097	-0.767253945	3.60445733398e-87	1.67488556358e-86	-
YGR002C	50.83287	87.810379	-0.788629786	7.54036526491e-87	3.50116586122e-86	-
YJL123C	143.132965	116.31086	0.29937017	1.08869777722e-86	5.05129731015e-86	-
YOL007C	29.746155	12.67314	1.230929175	2.05013829387e-86	9.50504744461e-86	up
YDR156W	80.133202	165.916153	-1.049982318	2.28209118459e-86	1.05725537865e-85	down
YIL093C	52.709263	102.727989	-0.962700883	2.61033853142e-86	1.20842517547e-85	-

YGL112C	63.751301	103.896263	-0.704617074	3.08561865247e-86	1.42738603536e-85	-
YEL061C	13.647967	6.878285	0.988565263	3.31014452052e-86	1.53010999132e-85	-
YNL056W	106.582382	64.506538	0.724451685	3.82593122617e-86	1.76721585209e-85	-
YDR081C	22.916029	40.59441	-0.824924008	4.10008096943e-86	1.89243885935e-85	-
YDR205W	17.036385	34.216618	-1.006077931	4.67399200122e-86	2.15573122909e-85	down
YAL031C	20.05559	38.224133	-0.93047937	5.14364554275e-86	2.37058289008e-85	-
YOL010W	27.08436	57.567974	-1.08780644	6.4933149689e-86	2.99039312514e-85	down
YGL058W	180.801483	123.995277	0.544121342	8.15556644622e-86	3.75313243129e-85	-
YJR154W	13.382612	36.320763	-1.440434783	1.07935651785e-85	4.96344093542e-85	down
YKL135C	50.445686	80.623604	-0.676471377	1.27115177445e-85	5.84108824264e-85	-
YKL104C	44.721703	30.934298	0.531767832	1.39269040099e-85	6.39483876429e-85	-
YNL002C	60.925175	109.524231	-0.84613969	1.39579751467e-85	6.40436878869e-85	-
YIL126W	40.570457	60.926975	-0.586651559	1.40527492491e-85	6.44309212248e-85	-
YNL014W	152.13266	138.817734	0.132138021	3.93564529145e-85	1.80313549589e-84	-
YBR247C	21.176527	44.552311	-1.073034265	4.53891694348e-85	2.07799383371e-84	down
YCR047C	34.567333	74.167763	-1.101382958	6.34866007455e-85	2.90438332666e-84	down
YPR137W	15.672498	34.130245	-1.122815625	1.70518477758e-84	7.79513041178e-84	down
YOL129W	540.620667	753.76416	-0.479496535	2.09184908689e-84	9.55570208344e-84	-
YBR106W	187.545547	300.704987	-0.681107786	2.8903368908e-84	1.31935378074e-83	-
YLR183C	15.53112	5.47871	1.503253725	2.99058131271e-84	1.36410938937e-83	up
YNR064C	35.635578	74.540207	-1.064700501	3.28662769849e-84	1.49804587021e-83	down
YLR293C	229.25267	347.873413	-0.601623886	4.11513651971e-84	1.87430429306e-83	-

YHL024W	24.329414	13.796406	0.818409082	4.35687103749e-84	1.98295127571e-83	-
YHR089C	32.746342	77.733772	-1.247207768	4.75950069183e-84	2.16461394102e-83	down
YER167W	24.970146	43.978168	-0.81658325	5.77221287465e-84	2.62327214684e-83	-
YKL075C	36.787689	68.338356	-0.893472492	1.06027213099e-83	4.81504710255e-83	-
YER133W	332.800507	289.508606	0.201051397	1.30978014098e-83	5.94379759885e-83	-
YBR251W	88.171883	147.943604	-0.746656751	1.37212287998e-83	6.22216131405e-83	-
YIL030C	24.337271	39.611977	-0.702769303	1.55040588688e-83	7.02548886552e-83	-
YPR004C	96.635994	156.288132	-0.693575674	1.80115723628e-83	8.15578710636e-83	-
YER126C	48.968678	96.618187	-0.980435533	1.87793876511e-83	8.4972622841e-83	-
YNL045W	67.415382	50.985367	0.402994558	2.34917944343e-83	1.06217814893e-82	-
YNL186W	28.708174	49.741692	-0.792994013	6.08116043943e-83	2.74758690015e-82	-
YKL212W	24.93347	46.853676	-0.910078565	1.07420337749e-82	4.8499305945e-82	-
YLR049C	34.67675	18.545635	0.902889025	1.09614907423e-82	4.94541675351e-82	-
YER031C	157.809784	112.943993	0.482579111	1.41025765234e-82	6.35793718643e-82	-
YDR192C	27.446674	55.128342	-1.006162879	1.42165574724e-82	6.40467262617e-82	down
YOL039W	161.300262	296.094788	-0.876310314	1.65371987625e-82	7.44473748497e-82	-
YBR050C	272.664978	233.890457	0.221296408	2.32835496233e-82	1.0474222903e-81	-
YOL048C	124.182693	92.898819	0.418731962	2.90128298533e-82	1.3042117866e-81	-
YDL070W	300.997711	280.834991	0.100029814	3.30899377206e-82	1.48641341078e-81	-
YGR267C	193.147034	294.949829	-0.610770049	4.14651159169e-82	1.86128300515e-81	-
YOL062C	33.072678	61.424007	-0.89316276	4.15234519502e-82	1.86255483892e-81	-
YMR203W	160.257278	235.472931	-0.555171346	5.02377891282e-82	2.25181368164e-81	-

YFR006W	74.081757	54.625706	0.439538295	5.66246956049e-82	2.53626342219e-81	-
YBR127C	393.968323	369.681061	0.0917984993167	6.22502067968e-82	2.78622410811e-81	-
YDR279W	32.15588	15.089843	1.091504776	1.73578020044e-81	7.76348954205e-81	up
YGR184C	19.481701	30.686495	-0.655484223	2.93907959041e-81	1.31359295157e-80	-
YJL016W	30.918432	56.448566	-0.868469776	3.21613166125e-81	1.43638455777e-80	-
YBL104C	10.563491	21.531431	-1.027357513	3.8558063186e-81	1.7208372125e-80	down
YOR126C	44.295574	20.12895	1.137890634	4.87985744694e-81	2.1763042407e-80	up
YFR048W	30.516264	53.901241	-0.820740141	4.9461744747e-81	2.20429656417e-80	-
YGL223C	44.734959	26.511538	0.754782327	5.15181188681e-81	2.29429327068e-80	-
YFL017C	108.140358	62.244461	0.796887672	1.20572055638e-80	5.36567255484e-80	-
YDR179C	46.417412	16.862364	1.46085928	1.27817303868e-80	5.68402451585e-80	up
YCL051W	28.858435	52.941685	-0.875411049	1.41695360938e-80	6.29667001219e-80	-
YNL144C	75.476517	112.18335	-0.571758816	1.53677321259e-80	6.82424041757e-80	-
YDL149W	34.218719	54.75087	-0.678096137	2.23820630867e-80	9.93194050337e-80	-
YJL083W	41.800026	70.5252	-0.754635012	2.26380842187e-80	1.0038373345e-79	-
YIL039W	29.036163	55.78413	-0.942003929	2.37482050979e-80	1.0523116149e-79	-
YFR040W	122.328865	166.141342	-0.441646247	6.02133473115e-80	2.66622296797e-79	-
YGR065C	8.673362	22.113304	-1.350251368	6.02523417279e-80	2.66604802171e-79	down
YOR356W	76.450981	59.121784	0.370845211	6.12628001727e-80	2.70882808741e-79	-
YOR208W	78.706108	63.245617	0.315510098	7.96883300517e-80	3.52103311716e-79	-
YGR090W	4.153486	10.568216	-1.347337262	2.77076809372e-79	1.22339461777e-78	down
YNL108C	35.353943	74.312042	-1.071724887	2.79799881663e-79	1.23453991853e-78	down

YOR313C	8.715799	0.783497	3.47563327	2.96433496565e-79	1.30700223485e-78	up
YFR033C	1358.965332	1277.342163	0.08936362	3.56000416884e-79	1.56852419306e-78	-
YPL215W	43.016796	81.376244	-0.91970762	4.13030386701e-79	1.81850541889e-78	-
YLR323C	47.463367	93.276459	-0.974698574	5.05205971767e-79	2.2227630565e-78	-
YLR203C	86.721321	136.005783	-0.649209358	6.97239596861e-79	3.0654840066e-78	-
YLL053C	20.537094	62.617588	-1.608335883	7.69708757301e-79	3.38170698183e-78	down
YGR240C	78.322876	111.469429	-0.509142452	7.96759608658e-79	3.49807896078e-78	-
YLR312C	101.614059	75.945389	0.420065743	8.26890437442e-79	3.62779917713e-78	-
YKL181W	64.412453	106.567635	-0.726357813	1.00929939618e-78	4.42495102504e-78	-
YNL044W	157.176315	259.747772	-0.724727541	1.04880749913e-78	4.59491669344e-78	-
YPL092W	95.619392	146.539673	-0.615916164	1.65958030317e-78	7.26563788579e-78	-
YIL153W	29.210279	58.319668	-0.997506369	1.86038484834e-78	8.1390198298e-78	-
YKL103C	95.556892	74.45282	0.360033437	2.07663666068e-78	9.07870449964e-78	-
YBL043W	48.933643	95.430725	-0.963627141	2.94861261741e-78	1.28817643412e-77	-
YBR278W	65.660248	34.201897	0.940943853	3.11117796586e-78	1.35824140732e-77	-
YDR328C	821.869751	757.388306	0.117876629	6.29094693532e-78	2.74449744023e-77	-
YDL091C	89.640488	138.669052	-0.629423437	7.49059345502e-78	3.2655620905e-77	-
YER060W-A	7.287477	20.37471	-1.483288195	1.36501592534e-77	5.9466799049e-77	down
YIL101C	173.350204	153.742889	0.173169851	1.4253166398e-77	6.205025035e-77	-
YIL112W	32.652191	51.629635	-0.661019597	1.45136459147e-77	6.31399536357e-77	-
YNL244C	427.019562	647.75415	-0.601144191	1.81215844983e-77	7.87806698638e-77	-
YPL141C	30.620743	20.676702	0.5665032	2.69323771018e-77	1.17002237262e-76	-

YER013W	16.307562	28.981985	-0.829615296	3.34647808755e-77	1.452792725e-76	-
YPR029C	30.421228	50.905834	-0.742752613	4.64611344912e-77	2.0155885599e-76	-
YML017W	12.097966	27.387428	-1.178749275	4.819155944e-77	2.08919833103e-76	down
YDR482C	145.9702	256.220734	-0.811713356	4.84657761389e-77	2.09961994606e-76	-
YER042W	73.457993	38.974419	0.914391963	5.83738413124e-77	2.5270907034e-76	-
YJR075W	51.698971	90.054962	-0.800670204	8.50065461535e-77	3.67749573882e-76	-
YKL187C	145.356827	198.8591	-0.452147751	8.73481058111e-77	3.77616323729e-76	-
YHR062C	25.331085	9.462865	1.420559924	8.89452184887e-77	3.84253247305e-76	up
YML073C	174.633865	282.05423	-0.69163922	9.50908506059e-77	4.10517385648e-76	-
YIL109C	54.506516	81.584427	-0.581865086	1.0602650556e-76	4.57409691811e-76	-
YLR212C	17.105913	6.814124	1.327895001	1.53454757549e-76	6.61560510322e-76	up
YGR092W	32.607414	19.729048	0.724878687	1.59449990714e-76	6.86929592196e-76	-
YKL120W	13.944576	36.616623	-1.392794675	1.77807273642e-76	7.65483741171e-76	down
YER082C	25.757202	48.457233	-0.911736144	2.12734731142e-76	9.15216362393e-76	-
YFR011C	215.548309	158.766205	0.441107392	2.2529210856e-76	9.68568843449e-76	-
YIL156W	19.403046	33.659721	-0.794740066	2.47287458133e-76	1.06239483743e-75	-
YMR099C	258.153992	218.14624	0.2429363	2.76437811165e-76	1.18680908141e-75	-
YDL132W	39.80899	28.471241	0.483588891	3.02273097372e-76	1.29682887939e-75	-
YMR043W	119.026619	188.72348	-0.664989674	3.71902694475e-76	1.5944557509e-75	-
YDR152W	68.77462	122.376198	-0.831374815	5.54162504602e-76	2.374217273e-75	-
YOR134W	81.514565	129.600754	-0.668944344	7.42655983314e-76	3.17959196166e-75	-
YBR102C	39.027237	63.393826	-0.699861013	9.04548526949e-76	3.870046351e-75	-

YGL040C	80.56385	132.269211	-0.715272743	1.03296673216e-75	4.41643076669e-75	-
YDR424C	195.597961	117.30719	0.737599889	1.3416482196e-75	5.73224511167e-75	-
YER014W	54.664333	38.262154	0.514681727	1.5911744912e-75	6.79368035859e-75	-
YDR292C	33.595554	58.101841	-0.790313556	1.66692523326e-75	7.11221432857e-75	-
YBR095C	54.253517	91.804283	-0.758844798	1.80093130652e-75	7.67869612011e-75	-
YFL008W	6.568236	2.634452	1.318003071	2.21427614542e-75	9.43460968482e-75	up
YMR198W	9.466242	2.834988	1.739449176	2.42345539118e-75	1.03188004585e-74	up
YPL015C	118.219887	90.159264	0.390925103	4.88711544739e-75	2.07945254951e-74	-
YHL004W	34.148724	64.818909	-0.924583066	7.14814145513e-75	3.03942891462e-74	-
YBR197C	61.377533	116.705978	-0.927095897	8.49522949729e-75	3.60974570289e-74	-
YOR311C	51.027637	94.673698	-0.891684841	1.05066365716e-74	4.46136797788e-74	-
YNL066W	39.188732	22.842627	0.778710317	1.07163770609e-74	4.54731844115e-74	-
YNL219C	37.125916	64.204231	-0.790241751	1.14801831069e-74	4.86809950325e-74	-
YHR175W	132.526489	220.766098	-0.736237891	1.23370225332e-74	5.22786593079e-74	-
YPR061C	107.262459	170.938507	-0.672332194	1.58391284635e-74	6.70731988414e-74	-
YJR121W	1600.079224	1645.614136	-0.040482754	1.7211162372e-74	7.28336032757e-74	-
YBR158W	34.991283	21.61961	0.694655066	1.75582434787e-74	7.42517544386e-74	-
YPL243W	53.334919	38.249908	0.479624114	2.35498230569e-74	9.95216484258e-74	-
YJL153C	37.491665	65.163933	-0.797503784	3.30100184041e-74	1.39405574322e-73	-
YDR364C	31.035551	58.126972	-0.905285997	3.31749065026e-74	1.40006675437e-73	-
YDR190C	68.802284	109.816574	-0.674567445	3.61204615559e-74	1.52334120475e-73	-
YOR046C	29.742064	55.450829	-0.89870426	7.31422141666e-74	3.08259922299e-73	-

YIL048W	15.208188	27.133101	-0.835205669	8.08079475891e-74	3.40336322004e-73	-
YGR129W	37.052967	14.984199	1.306147106	8.73674780562e-74	3.67713426287e-73	up
YAL013W	82.213364	130.149704	-0.662727199	1.06628833268e-73	4.48476827187e-73	-
YLR433C	25.780666	14.283192	0.851971106	1.7723782361e-73	7.44950852382e-73	-
YIR018W	26.915285	61.556255	-1.193479761	1.84209619758e-73	7.73730256739e-73	down
YER140W	21.349796	41.565128	-0.96115137	1.85625851487e-73	7.79151647081e-73	-
YOR178C	164.233994	220.04953	-0.422075516	2.17125528619e-73	9.10753568693e-73	-
YJL078C	53.004974	41.440788	0.355076313	2.39634535452e-73	1.00449101694e-72	-
YMR161W	197.795547	299.476074	-0.5984308	2.39804708577e-73	1.0045260667e-72	-
YNR052C	28.675211	55.157253	-0.943746506	3.73548715253e-73	1.56371572777e-72	-
YHL001W	179.622589	298.629639	-0.733388568	3.74362423586e-73	1.56606598761e-72	-
YPL239W	109.790131	71.339668	0.62197197	4.32255877694e-73	1.80703332574e-72	-
YEL006W	96.51265	153.240128	-0.667004181	5.85121106147e-73	2.44443595354e-72	-
YNL164C	20.712242	45.9105	-1.148340417	6.40687676793e-73	2.67477410728e-72	down
YNL004W	70.051155	111.526398	-0.67090449	7.69542199176e-73	3.21056315355e-72	-
YPL232W	216.138916	312.291687	-0.530935312	8.8010221495e-73	3.66935832801e-72	-
YNL227C	12.884291	5.08775	1.340513464	9.26471285154e-73	3.86008975721e-72	up
YAL032C	32.324497	62.285027	-0.946257468	9.67080114738e-73	4.02658172521e-72	-
YMR029C	91.810722	72.298203	0.344702858	1.0601241915e-72	4.41102612654e-72	-
YDR034C	49.377068	76.070107	-0.623488462	1.49778939767e-72	6.22791465555e-72	-
YHR103W	31.097597	51.022976	-0.714343945	1.71895117605e-72	7.14273688148e-72	-
YNL078W	79.681969	57.736691	0.464764872	1.84759891019e-72	7.67216992273e-72	-

YLR347C	82.106583	69.071632	0.249404585	1.86103956477e-72	7.72281658965e-72	-
YJL019W	17.605158	34.047535	-0.951552178	2.15341766539e-72	8.93013818755e-72	-
YOR078W	33.874901	74.788696	-1.142603499	5.30124196166e-72	2.19693658865e-71	down
YJR064W	71.998978	110.146584	-0.61337642	6.68579347644e-72	2.76887297543e-71	-
YPL158C	34.053738	23.311308	0.546783212	6.89885020059e-72	2.85520413635e-71	-
YHR081W	109.81884	189.152359	-0.784423191	6.95019260427e-72	2.87453668803e-71	-
YJR072C	102.616768	77.904198	0.397493515	7.78294924178e-72	3.21681417397e-71	-
YNR056C	3.236824	12.012324	-1.891864466	8.59314010307e-72	3.54931561942e-71	down
YLR028C	195.237961	175.373062	0.154806429	8.92892031813e-72	3.68555434408e-71	-
YDL207W	29.047544	52.746727	-0.860665388	1.06171010648e-71	4.37946600734e-71	-
YDR143C	12.620435	27.384937	-1.117620924	1.07132861969e-71	4.41620721849e-71	down
YGR288W	22.309118	44.430141	-0.993905243	1.36569799391e-71	5.62591449648e-71	-
YNL315C	92.600914	148.844437	-0.684706964	1.43111085582e-71	5.89146962397e-71	-
YPR112C	12.918255	25.191416	-0.963521014	1.91602581919e-71	7.88249720711e-71	-
YDR196C	209.454346	167.122711	0.325728019	2.16055897125e-71	8.88261595599e-71	-
YDR153C	60.997845	100.772896	-0.724277482	2.33638765842e-71	9.59913605789e-71	-
YPR152C	49.775177	83.441162	-0.745332803	2.36356929991e-71	9.70439035307e-71	-
YJL069C	10.474513	24.163038	-1.205918686	2.95341626983e-71	1.21181812314e-70	down
YOR270C	72.54718	104.910606	-0.532169094	2.97356355834e-71	1.2192789016e-70	-
YGL180W	14.992131	28.122763	-0.907532879	3.35924662043e-71	1.3765150508e-70	-
YLR083C	9.800922	22.267181	-1.183929547	3.58741735024e-71	1.46904267218e-70	down
YMR323W	11.329487	28.093767	-1.310167546	4.16657452453e-71	1.70508204669e-70	down

YNL255C	347.669342	511.023376	-0.555673433	5.3914846017e-71	2.20489699653e-70	-
YOR381W	5.582799	14.923244	-1.418500661	6.79271999442e-71	2.77611624262e-70	down
YLR292C	75.268188	138.507355	-0.879850442	7.36457329176e-71	3.007846776e-70	-
YNL262W	4.723032	2.307397	1.033447061	7.37272322167e-71	3.00919564498e-70	up
YNL168C	207.947754	168.052567	0.307308523	7.79770377818e-71	3.18056143594e-70	-
YOR232W	213.976242	318.073395	-0.571909082	8.34501203093e-71	3.4015649828e-70	-
YCR051W	114.98246	78.837959	0.544451469	9.37125058807e-71	3.81737031829e-70	-
YDR452W	77.244957	62.084908	0.315198137	1.50274501081e-70	6.11740395218e-70	-
YCL050C	72.9291	48.842567	0.578355565	1.60417479752e-70	6.52602696132e-70	-
YPR026W	50.724285	41.925289	0.274855896	1.6724248975e-70	6.79922315891e-70	-
YLR390W-A	411.635406	564.023804	-0.454388973	2.07749429492e-70	8.44050038145e-70	-
YGR082W	82.630219	150.415573	-0.864212545	2.11857593069e-70	8.60177853351e-70	-
YFL047W	6.59429	16.578323	-1.330008837	2.37331305943e-70	9.62975651827e-70	down
YLL018C	300.094116	280.534821	0.0972351775367	2.55294762814e-70	1.03518607939e-69	-
YMR050C	13.540581	9.115794	0.570849416	3.32256147684e-70	1.34637478122e-69	-
YFR053C	2008.577637	2084.568115	-0.053574288	4.21948992342e-70	1.70871451041e-69	-
YOR231W	18.12034	37.168438	-1.036468035	5.21385881352e-70	2.11001535296e-69	down
YGL073W	88.55394	75.347237	0.233001889	5.24470633481e-70	2.12111641215e-69	-
YNL113W	42.909172	98.861244	-1.204118998	6.42918159332e-70	2.59846089397e-69	down
YIL076W	100.196274	71.702652	0.482730475	7.22928879015e-70	2.91993655233e-69	-
YPR074C	19.000248	35.813816	-0.914497998	7.73794652196e-70	3.1233531865e-69	-
YPL049C	98.920464	148.55719	-0.58667752	8.21465113344e-70	3.31361625967e-69	-

YOR011W	6.896991	13.974461	-1.01875365	8.39710196132e-70	3.38501356986e-69	down
YDL090C	39.34502	23.814001	0.724371013	8.6019049968e-70	3.46532292149e-69	-
YLR369W	27.017771	47.483894	-0.813529595	9.52576843938e-70	3.83501754032e-69	-
YPL236C	47.862015	84.391922	-0.818223775	1.30653829116e-69	5.25663623561e-69	-
YGR007W	67.442207	113.758369	-0.754249032	1.63906550048e-69	6.59023227138e-69	-
YER070W	7.930673	3.072013	1.368258978	1.70454885437e-69	6.84908691776e-69	up
YER142C	121.837128	91.368813	0.415180121	1.76847435492e-69	7.1013510966e-69	-
YHL039W	31.783142	55.279362	-0.798479212	2.00341326885e-69	8.03955369944e-69	-
YDR071C	236.453232	185.792664	0.347861323	2.05688651243e-69	8.24880585863e-69	-
YOL054W	61.893085	101.681351	-0.716204966	2.28540893919e-69	9.15934066785e-69	-
YDR128W	23.936827	38.721931	-0.693918974	2.37293618201e-69	9.50399214059e-69	-
YPR190C	35.886929	59.677799	-0.733735857	2.4162325687e-69	9.67116169343e-69	-
YCR057C	4.733908	12.152408	-1.360138638	2.42806799745e-69	9.71227198978e-69	down
YGL221C	90.40374	62.697811	0.527967384	3.00360343602e-69	1.20066774828e-68	-
YPR033C	20.351002	39.670986	-0.962984427	3.32353916202e-69	1.32770470514e-68	-
YLL034C	5.450727	13.774687	-1.337498967	3.34287674055e-69	1.33457098427e-68	down
YKL172W	30.381432	57.00555	-0.907912514	6.38177668777e-69	2.54614843687e-68	-
YKR049C	653.027161	569.810547	0.196660673	6.50991797075e-69	2.5956050586e-68	-
YER160C	11.438141	7.36154	0.635773087	8.65038603273e-69	3.44682904308e-68	-
YBR056W	291.862183	270.032959	0.112151784	1.04510789705e-68	4.16166120905e-68	-
YHR073W	31.61482	49.91737	-0.658940919	1.05409103244e-68	4.19474174961e-68	-
YNL047C	23.634872	42.593815	-0.849726902	1.11198346439e-68	4.42228913961e-68	-

YPR043W	605.862854	881.462646	-0.540908178	1.1870458864e-68	4.7177854435e-68	-
YPR175W	9.964613	3.827514	1.380406121	1.2080613828e-68	4.7982374052 6e-68	up
YLR025W	247.853058	357.750366	-0.529468184	1.2577093807 2e-68	4.9922377464 7e-68	-
YNL289W	40.667019	21.043346	0.950495123	2.2645975651 4e-68	8.9831448462 5e-68	-
YDR063W	246.26973	186.198364	0.403398914	2.6850286144 9e-68	1.0644098109 1e-67	-
YNL098C	449.474792	415.809906	0.112316079	3.5963692451 4e-68	1.4247772989e-67	-
YLR189C	57.058949	80.95768	-0.504714774	4.7027557288 9e-68	1.8619073702 1e-67	-
YKL155C	10.294168	23.138916	-1.168494047	4.8443877295 9e-68	1.9167596574 5e-67	down
YGR147C	151.033127	226.73465	-0.586139864	8.0737437383 3e-68	3.1924714094e-67	-
YLR220W	149.544235	221.386902	-0.565997575	8.3085475740 6e-68	3.2832249102 3e-67	-
YDR427W	166.451508	139.915741	0.250543661	1.4534581063 6e-67	5.7398650917 7e-67	-
YLR060W	23.369888	42.968937	-0.878643267	1.4956226123 1e-67	5.9026224903 1e-67	-
YGR031W	193.722855	163.210846	0.247257237	1.9715650298e-67	7.7760328494 3e-67	-
YMR174C	1757.56897	1597.975342	0.137336158	2.7701671809 7e-67	1.0918855783 8e-66	-
YPL086C	18.163803	35.972095	-0.985811889	3.0225176311 1e-67	1.1905957775 3e-66	-
YML026C	187.10405	301.161316	-0.686695681	3.6180446235 8e-67	1.4242752709 7e-66	-
YER049W	8.224007	19.571568	-1.250845946	4.7580512047 7e-67	1.8718619695 3e-66	down
YCR048W	15.30548	30.887997	-1.012998029	6.5119739177 4e-67	2.5602491501 8e-66	down
YOL058W	85.635986	64.74987	0.403339878	7.6961987820 6e-67	3.0239241796 9e-66	-
YPL060C-A	5.471926	2.670179	1.035112261	8.8730691209 4e-67	3.4841248009 4e-66	up
YJR056C	95.122093	63.984947	0.572047921	1.0964871837 3e-66	4.3027765086e-66	-
YBR199W	167.860107	234.168854	-0.480289795	1.1762448410 2e-66	4.6128414232 9e-66	-

YPL134C	382.745178	347.800812	0.138122901	1.19522713906e-66	4.68432454502e-66	-
YCL045C	12.89251	25.67701	-0.993944051	1.38706871017e-66	5.43275870836e-66	-
YJL084C	30.086109	47.248333	-0.651165893	2.05810854844e-66	8.05595073691e-66	-
YLR229C	124.539032	204.829926	-0.71782854	2.6544515663e-66	1.03836391453e-65	-
YMR117C	24.223932	7.592208	1.673841638	3.44660717846e-66	1.34738900276e-65	up
YGR014W	11.184673	6.53838	0.774517946	4.62053115218e-66	1.8051766767e-65	-
YCL042W	184.131348	123.474976	0.576516575	4.82772040028e-66	1.88493636761e-65	-
YDR367W	51.390095	27.057421	0.925465985	5.45844230234e-66	2.12985605361e-65	-
YPL173W	53.38026	94.849113	-0.82932795	7.43772871212e-66	2.90034044252e-65	-
YJL113W	5.891665	2.778831	1.084197304	1.08354180438e-65	4.22261614665e-65	up
YKL094W	253.706146	352.55603	-0.474694087	1.31660872553e-65	5.12767061987e-65	-
YOR030W	27.7747	48.489491	-0.803900781	1.59828527566e-65	6.22078682839e-65	-
YNR013C	9.917417	20.158791	-1.023372794	2.69357499603e-65	1.0477264145e-64	down
YIL042C	85.018379	131.818802	-0.632709507	2.93880717718e-65	1.14239918322e-64	-
YHR006W	224.994751	299.95871	-0.41487258	4.63377007724e-65	1.8001529812e-64	-
YFR019W	7.458935	4.633168	0.686970659	5.12343249431e-65	1.98913501718e-64	-
YPL019C	18.905809	33.539028	-0.827011293	5.24683549364e-65	2.03577217153e-64	-
YDR379C-A	297.579865	204.648636	0.540127862	5.34754539909e-65	2.07355164507e-64	-
YMR260C	166.304291	269.224487	-0.694984241	7.7226629705e-65	2.99265241703e-64	-
YLR050C	36.963989	13.341272	1.470224231	8.0665620417e-65	3.123968631e-64	up
YOL077W-A	754.267029	614.795166	0.294969542	8.40225980808e-65	3.25194693819e-64	-
YER063W	196.304794	293.375214	-0.579651584	9.28411112914e-65	3.59101320185e-64	-

YIR012W	28.91576	53.94408	-0.899608621	1.01327505837e-64	3.91681915464e-64	-
YBR235W	10.957239	20.520351	-0.905171094	1.02812571462e-64	3.97175136053e-64	-
YDR322W	59.921162	99.529053	-0.732052116	1.05517243374e-64	4.07370054022e-64	-
YHR019C	56.906101	88.952774	-0.644456262	1.19628219178e-64	4.61561208614e-64	-
YAL019W	5.388558	12.131463	-1.170782385	1.3783420203e-64	5.3147498522e-64	down
YJL187C	13.208007	6.858195	0.94551196	1.43192840951e-64	5.51794634776e-64	-
YLR450W	21.521885	35.593811	-0.725821967	1.46847636199e-64	5.65527373154e-64	-
YOR224C	51.017002	108.237152	-1.085145757	1.76124944192e-64	6.77857193767e-64	down
YDR082W	29.860573	53.629864	-0.844794745	1.87214516774e-64	7.20091524247e-64	-
YDR387C	26.531551	47.664288	-0.84519973	1.900414604e-64	7.30512313412e-64	-
YOR034C	16.868177	31.344124	-0.893890945	2.44361232102e-64	9.38734238174e-64	-
YNL177C	92.155693	146.242279	-0.666215261	2.8524434222e-64	1.09511247774e-63	-
YBR189W	274.628845	398.231628	-0.536124643	3.28214636142e-64	1.25930560023e-63	-
YPL020C	65.413887	98.340424	-0.588187631	4.3688260187e-64	1.67521136035e-63	-
YML072C	46.056984	39.490887	0.221900168	4.38704523198e-64	1.68115906174e-63	-
YEL043W	30.863482	48.67342	-0.657233313	4.73224423975e-64	1.81232401236e-63	-
YDR080W	19.22818	12.472809	0.624435803	5.3498569639e-64	2.0475901376e-63	-
IRT1	35.277565	61.064259	-0.791577232	7.8582540685e-64	3.00579428572e-63	-
YAL017W	43.326668	62.077862	-0.518823579	9.05338649481e-64	3.46080193102e-63	-
YLR427W	22.676943	40.382511	-0.832504452	9.83377384261e-64	3.75680418553e-63	-
YNL157W	744.358459	988.080322	-0.408630781	1.04204005775e-63	3.97846536192e-63	-
YGR282C	1049.564575	1039.979492	0.0132358526653	1.28488943606e-63	4.90263897913e-63	-

YDL136W	340.143738	510.285004	-0.585158716	1.47243096352e-63	5.61477360045e-63	-
YGR083C	23.881901	42.279686	-0.824046983	1.77950787087e-63	6.78157450115e-63	-
YFR010W	171.256195	150.682678	0.184642598	2.01566449184e-63	7.67683752476e-63	-
YNR003C	42.279953	24.33543	0.796915506	3.54726831693e-63	1.35018036245e-62	-
YOR294W	46.007233	91.435799	-0.990898429	4.08985220426e-63	1.55574770123e-62	-
YBL091C	182.00705	252.820847	-0.474121096	4.2680503181e-63	1.62253866349e-62	-
YMR115W	84.553368	126.069084	-0.576280399	5.4475654567e-63	2.06967480754e-62	-
YLR163C	72.029533	111.159416	-0.625969704	6.93022174296e-63	2.63136492846e-62	-
YMR280C	77.624962	103.987099	-0.421811992	7.71015526925e-63	2.92571173053e-62	-
YCL048W-A	86.422607	35.438919	1.286074154	9.72832086214e-63	3.68927403251e-62	up
YIR029W	92.768707	144.043915	-0.634798578	1.2014056848e-62	4.55331287621e-62	-
YER102W	241.87883	354.033112	-0.549599793	1.33261827887e-62	5.04752548823e-62	-
YFR052W	170.171448	137.160522	0.311123697	1.84639781936e-62	6.98929125767e-62	-
YKL188C	27.295891	18.895706	0.530625368	2.22062464893e-62	8.40075430867e-62	-
YOR246C	42.335499	76.345215	-0.850669844	2.37115801252e-62	8.96476793041e-62	-
YBL063W	3.946059	1.122154	1.814141848	4.09940213377e-62	1.54894025846e-61	up
YMR171C	60.350731	92.877808	-0.621962678	1.30627858463e-61	4.93271134635e-61	-
YMR044W	104.947487	152.709625	-0.541123374	1.54351815533e-61	5.82502170716e-61	-
YGR136W	234.495468	195.685822	0.261020808	2.00394774513e-61	7.55802405938e-61	-
YNL193W	38.961159	64.213882	-0.720848624	2.83093302204e-61	1.06705720709e-60	-
snR86	71.876534	116.401764	-0.695520175	2.84343742016e-61	1.07112011556e-60	-
YLR007W	39.845688	23.202032	0.780172448	2.90358633539e-61	1.09311485568e-60	-

YMR093W	8.480198	20.843439	-1.297423475	3.57216467927e-61	1.34399989872e-60	down
YJR090C	17.517298	29.250717	-0.739691729	3.9786984471e-61	1.49604845304e-60	-
YDR266C	10.916836	23.246378	-1.090451165	4.09772345557e-61	1.53987089662e-60	down
YPL193W	44.369396	28.135393	0.657178799	5.03972392097e-61	1.89271664255e-60	-
YCL017C	60.379913	94.219292	-0.641953813	5.2156306493e-61	1.95759583258e-60	-
YPR165W	640.925049	842.927124	-0.395252252	5.38472112936e-61	2.0198398049e-60	-
YOR206W	25.892279	43.958542	-0.763621582	6.14669506855e-61	2.30426829623e-60	-
YGL106W	212.616074	159.747269	0.412459403	6.71728395864e-61	2.51665050182e-60	-
YBR255C-A	170.778839	115.080139	0.569490355	8.94289756032e-61	3.34846248821e-60	-
YJR031C	5.882041	2.749728	1.097027924	9.54375129266e-61	3.57128438968e-60	up
YGR260W	54.416149	85.260521	-0.64784301	1.0458722846e-60	3.91131032697e-60	-
YMR255W	419.506805	578.379761	-0.463322856	1.52846445589e-60	5.71264740646e-60	-
YHR013C	51.24411	28.91363	0.825636445	1.8011493654e-60	6.72775888111e-60	-
YLR290C	55.083416	33.866791	0.701746738	2.38060172253e-60	8.88681629193e-60	-
YDL141W	15.046152	28.803736	-0.936861379	2.48833621561e-60	9.28340818899e-60	-
YJR048W	1105.358521	1017.21228	0.119893597	2.59869791898e-60	9.68931932794e-60	-
YGR285C	67.709915	105.819977	-0.644172996	2.98183796236e-60	1.111119452e-59	-
YGL011C	650.334961	615.639709	0.0790966938563	3.92665205787e-60	1.46230689714e-59	-
YEL077C	7.499545	3.80756	0.977936298	4.42927807745e-60	1.64849869933e-59	-
YLR179C	67.178482	39.594486	0.762699664	4.55655405477e-60	1.69485246087e-59	-
YAL048C	12.767627	25.689623	-1.008695309	5.08203802936e-60	1.88917916684e-59	down
YDR208W	138.19632	184.871597	-0.419804392	5.66371082155e-60	2.10414822143e-59	-

YBL055C	32.911064	59.035576	-0.843011944	6.73174581917e-60	2.49944246683e-59	-
YOR077W	39.165058	77.319336	-0.981262153	7.28248891796e-60	2.70231268396e-59	-
YNR075W	5.773554	18.249012	-1.660286788	7.88392820236e-60	2.9237411159e-59	down
YPR024W	74.949806	107.123947	-0.515284374	1.22639026953e-59	4.54533181687e-59	-
YLR226W	21.255413	42.763416	-1.008546811	1.37953921302e-59	5.10989226399e-59	down
YMR070W	50.403919	80.736794	-0.679690389	4.11040012178e-59	1.52160786858e-58	-
YLR130C	28.531393	52.485661	-0.879373149	7.60533103964e-59	2.81370054196e-58	-
YML101C	131.370865	81.859093	0.682430768	8.11450919476e-59	3.00029023711e-58	-
YNL330C	82.922508	125.229774	-0.594741954	1.06875090464e-58	3.94928905714e-58	-
YCR026C	38.087051	59.630585	-0.646751903	1.13605747078e-58	4.19550551971e-58	-
YKL152C	2119.063721	2160.994385	-0.028268353	1.36698291521e-58	5.04532100929e-58	-
YKL159C	84.438461	143.003906	-0.760082364	1.92898669331e-58	7.11535911589e-58	-
YLR054C	22.198927	38.36414	-0.78926847	2.10436014585e-58	7.75764120274e-58	-
YDR236C	48.220963	91.23764	-0.919968667	2.6117156095e-58	9.62227329602e-58	-
YIL144W	22.268578	13.895406	0.680401447	2.95567418656e-58	1.08830518091e-57	-
YFR049W	578.513428	503.063446	0.201610083	3.1565747194e-58	1.16158955886e-57	-
YHR211W	7.311173	3.410619	1.100069289	3.66877147204e-58	1.34927329967e-57	up
YML080W	7.591268	20.341877	-1.422040017	4.44610049222e-58	1.63418542663e-57	down
YMR295C	923.785889	1183.38269	-0.357286283	4.45712427085e-58	1.63726789783e-57	-
YBL097W	6.051311	1.912641	1.661681625	5.68072805289e-58	2.08550915153e-57	up
YJR119C	7.535664	16.9263	-1.167460098	7.20572067016e-58	2.64380105912e-57	down
YPL190C	102.38871	139.950073	-0.450855596	9.28222012261e-58	3.40366346847e-57	-

YMR290C	18.361986	35.736046	-0.96065791	9.3459037697 3e-58	3.4249923614 2e-57	-
YOR284W	20.638847	7.134446	1.532489064	1.1544199664 2e-57	4.2281056941 3e-57	up
YDR392W	37.49007	67.887268	-0.856632508	1.2359646067 3e-57	4.5240968623 8e-57	-
YJL088W	9.671998	25.682224	-1.408884289	1.4808116633 3e-57	5.4171354189 6e-57	down
YGL129C	68.273933	105.162003	-0.623206759	1.7104841786 6e-57	6.2536429806 4e-57	-
YDL159W	61.749565	94.619766	-0.615712619	1.8584803171 1e-57	6.7907273741 e-57	-
YPL143W	313.303101	476.246155	-0.604148396	1.9016248513 1e-57	6.9442865158 5e-57	-
YOR123C	90.514832	133.360291	-0.559103037	2.8412229183 1e-57	1.0369377940 5e-56	-
YKR081C	24.052469	48.050861	-0.998377287	3.1394213529 3e-57	1.1450956380 1e-56	-
YCL014W	4.633102	2.136202	1.116930369	3.1854077026 7e-57	1.1611867890 9e-56	up
YIL115C	23.314077	18.008656	0.372510799	3.3782155171 4e-57	1.2307489395 8e-56	-
YLR264W	37.739307	109.525803	-1.537130956	3.7582820507 6e-57	1.3684114352 5e-56	down
YKL058W	227.55127	355.407745	-0.643283482	3.8950403282 3e-57	1.4173745813 4e-56	-
YDR419W	19.428635	35.350632	-0.863551463	3.9401322395 1e-57	1.4329432304 e-56	-
YOR153W	9.126362	16.196224	-0.827545716	4.3116769271 2e-57	1.5671481477 5e-56	-
YNL302C	228.493195	347.919983	-0.606604344	5.3893017751 3e-57	1.9576820023 4e-56	-
YDR142C	29.684938	55.146748	-0.893544713	7.3991708859 5e-57	2.6862019216 4e-56	-
YPR095C	10.40328	18.748112	-0.849706858	7.5028129492 9e-57	2.7222362822 4e-56	-
YBR115C	24.280966	36.811256	-0.600321156	1.0147501198 9e-56	3.6796546403 3e-56	-
YML045W	14.042803	10.120393	0.472565618	1.0457129454 3e-56	3.7897174344 6e-56	-
YIL074C	24.324465	44.893589	-0.884101362	1.2401452779 6e-56	4.4917280546 e-56	-
YGL197W	36.14534	51.583389	-0.513096899	1.2458995725 e-56	4.5099385108 4e-56	-

YHR061C	16.47543	5.931805	1.473773042	1.33075507679e-56	4.81429342466e-56	up
YHR071W	152.031693	229.308533	-0.592917938	1.48574792838e-56	5.37188301652e-56	-
YMR068W	38.959839	25.196251	0.628778627	1.96677561072e-56	7.10695168297e-56	-
YOR355W	38.513546	63.354816	-0.718088332	2.70825748969e-56	9.78060645493e-56	-
YPR155C	61.862095	49.004253	0.336148728	2.84304440716e-56	1.02614067905e-55	-
YDR075W	29.87714	57.848568	-0.953239195	3.21541900745e-56	1.15986758851e-55	-
YML086C	66.166397	99.743301	-0.592121228	3.41750003291e-56	1.23204646947e-55	-
ITS1-1	110.704933	197.316147	-0.833789511	3.66745904515e-56	1.32139209241e-55	-
YDR395W	10.578604	20.088617	-0.92522899	3.74077968739e-56	1.34702785959e-55	-
YKL193C	70.675209	50.668041	0.480128194	3.92915887816e-56	1.41404164149e-55	-
YDL144C	61.832069	43.469814	0.508341359	5.12712446277e-56	1.84410131314e-55	-
YEL031W	35.141697	51.521496	-0.551990624	5.45111756336e-56	1.95949842695e-55	-
YEL034W	2647.65918	3232.080322	-0.287745629	6.35844502197e-56	2.28433024863e-55	-
YKR028W	13.080705	23.206041	-0.827060118	7.6195043808e-56	2.73579428548e-55	-
YNL130C	30.830187	17.930779	0.781905473	1.00329259312e-55	3.60025457691e-55	-
YHL040C	13.450054	26.546825	-0.98092736	1.05281568054e-55	3.77578263707e-55	-
YMR056C	315.656647	286.497528	0.13983344	1.07593663364e-55	3.85647495476e-55	-
YOR136W	225.202805	200.741364	0.165886873	1.18139790057e-55	4.23203587232e-55	-
YGR203W	54.974358	26.813553	1.035796451	1.18587333718e-55	4.24561803761e-55	up
YJR015W	34.5895	58.132275	-0.74900521	1.31557840854e-55	4.70726844968e-55	-
YFL024C	40.390533	61.088646	-0.59688708	1.51654616451e-55	5.42322499382e-55	-
YHR021C	424.420074	638.4104	-0.58899126	1.56372108435e-55	5.58870494625e-55	-

YDL015C	192.901001	164.902084	0.226250998	2.3243677034 2e-55	8.3024595528 5e-55	-
YGR150C	15.793719	27.897337	-0.820776486	2.6845220671 6e-55	9.5833887250 7e-55	-
YJL093C	24.691265	41.716366	-0.756612736	3.1773366363 7e-55	1.1336152780 8e-54	-
YER111C	4.098807	1.341043	1.611848562	3.3617606949 4e-55	1.1987254677 9e-54	up
YBL036C	85.381073	137.801331	-0.690601624	3.3621948019 1e-55	1.1981920396 2e-54	-
YPL266W	26.413719	52.051674	-0.978657117	3.8012729021 4e-55	1.3538899699 6e-54	-
YML052W	92.573936	69.196304	0.419911082	4.341463541e-55	1.5454017008 3e-54	-
YDR320C-A	184.064148	111.613533	0.721696683	4.3890081507 e-55	1.5614305214 6e-54	-
YER104W	85.588516	57.03175	0.585651931	4.7311261533 4e-55	1.6821781878 6e-54	-
YHR049W	62.143192	107.338676	-0.788501745	6.7270641687 7e-55	2.3904759221 4e-54	-
YGL064C	3.915324	11.850919	-1.597795337	6.9059290507 e-55	2.4526320106 8e-54	down
YHR009C	66.593811	100.028008	-0.586944004	7.1290555383 2e-55	2.5304274889 6e-54	-
YHR030C	54.082413	84.753937	-0.648120864	7.4389373682 1e-55	2.6389098961 1e-54	-
YNL033W	17.075928	39.123329	-1.19606515	7.5327559093 9e-55	2.6706652590 2e-54	down
YHR072W-A	487.631958	753.825378	-0.628437686	7.5890757113 7e-55	2.6890971470 4e-54	-
YKL127W	18.582294	10.304205	0.850695414	8.0519033149 9e-55	2.8514669583 3e-54	-
YDR212W	55.733994	85.013718	-0.609138113	1.0742716242 4e-54	3.8022110851 2e-54	-
YOR370C	55.804173	84.18763	-0.593235258	1.1207412252 5e-54	3.9644225221 4e-54	-
YGL016W	5.086785	1.96532	1.371989882	1.1786140985 8e-54	4.1667632824 7e-54	up
YOL135C	80.501076	134.497864	-0.740503289	1.2160369306 1e-54	4.2966176808 4e-54	-
YDR003W	263.276093	223.060593	0.23914086	1.3194082723 e-54	4.6592073688 6e-54	-
YGL256W	23.884171	46.004353	-0.945715575	1.5385954348 e-54	5.4301310172 e-54	-

YML103C	8.838866	5.549088	0.671610606	1.7477839186e-54	6.16491054924e-54	-
YCR075C	21.885262	8.734777	1.325116879	1.79209449629e-54	6.31761648664e-54	up
YHR053C	27.701759	1.065669	4.70014628	1.90532080239e-54	6.71295774189e-54	up
YGR186W	88.429062	122.982033	-0.47585507	2.36950657445e-54	8.343673746e-54	-
YDR217C	8.994394	16.327341	-0.860191871	2.98986942036e-54	1.05221708399e-53	-
YBL038W	142.418777	215.080017	-0.594734122	3.1409031034e-54	1.10474370912e-53	-
YGR070W	39.727501	32.73439	0.279330951	3.99867696742e-54	1.40565043113e-53	-
YBR195C	5.044737	15.368555	-1.607130561	4.29326462738e-54	1.50835239427e-53	down
YPL123C	106.784843	88.199081	0.275871357	5.81712423776e-54	2.0425739405e-53	-
YKR041W	17.166809	40.629288	-1.242898189	5.94251086964e-54	2.08542156465e-53	down
YDR524C-B	3148.641846	3977.357178	-0.337080464	7.26271745038e-54	2.54728530689e-53	-
YBL050W	241.77124	333.033447	-0.462024438	7.84652471187e-54	2.75049268274e-53	-
YDL099W	77.9841	57.959084	0.428145212	9.58717054075e-54	3.35875590954e-53	-
YNL026W	44.440964	31.395695	0.501323362	9.62561293539e-54	3.37032177681e-53	-
YBR221C	298.692413	395.194733	-0.403903127	1.00727177555e-53	3.52488341748e-53	-
YDR299W	17.545147	33.443558	-0.930656306	1.32267176089e-53	4.62599791075e-53	-
YKR016W	112.519691	96.872437	0.216019355	1.45723484461e-53	5.09375783522e-53	-
YER105C	21.699856	33.146362	-0.611165066	1.66336197707e-53	5.81100233746e-53	-
YDR004W	45.464203	73.606674	-0.695105521	1.68455569419e-53	5.88173326745e-53	-
YHR032W	45.842319	71.332497	-0.637879449	2.09928566824e-53	7.32566915594e-53	-
YNL183C	41.843792	63.060886	-0.591731841	2.36067811237e-53	8.2331964728e-53	-
YML109W	8.934715	4.520638	0.982895316	2.38715306229e-53	8.3208569403e-53	-

YIR019C	5.456113	2.590853	1.074446362	2.45566342995e-53	8.55485890747e-53	up
YBR152W	12.894627	31.743462	-1.299689439	2.66146118062e-53	9.26660179993e-53	down
YGL226C-A	101.956886	52.748016	0.95077048	2.662050873e-53	9.26345954013e-53	-
YHR179W	248.729965	331.470367	-0.414299579	2.69867691353e-53	9.38565057658e-53	-
YLR129W	8.651211	17.037792	-0.977764382	2.76035249543e-53	9.59477507931e-53	-
YAL043C	20.016386	34.109379	-0.768986973	2.92448208807e-53	1.01595885857e-52	-
YDR175C	115.326546	171.199066	-0.569950198	2.93550778906e-53	1.01921881177e-52	-
YJL126W	31.25074	16.854374	0.890767308	3.14330073309e-53	1.09075522365e-52	-
YDL007W	277.209106	258.372528	0.101521969	4.05529151518e-53	1.40643853219e-52	-
YDR245W	55.430321	88.963783	-0.682542775	5.60917347727e-53	1.94426292278e-52	-
YBL086C	241.987488	224.102875	0.110771297	6.3984209925e-53	2.21659584383e-52	-
YOR354C	24.120617	40.566738	-0.750030488	6.53578046647e-53	2.26291830094e-52	-
YAR009C	11.75077	7.352828	0.676384154	6.86039464629e-53	2.37398717749e-52	-
YDR018C	55.300056	39.417206	0.488455424	7.57383627313e-53	2.61940811051e-52	-
YOL049W	46.960472	33.896057	0.470329449	8.02793980603e-53	2.77491371469e-52	-
YLL055W	94.612808	79.435623	0.252249368	1.05067500553e-52	3.62971087051e-52	-
YGR157W	22.354261	15.457316	0.532260021	1.23303359409e-52	4.25732622474e-52	-
YER100W	174.865814	253.404968	-0.535196537	1.49300665215e-52	5.15207631825e-52	-
YLR272C	4.651031	1.849359	1.330525246	1.51528794121e-52	5.22605974392e-52	up
YNL241C	212.849136	196.33107	0.116542732	1.62785855805e-52	5.61118596799e-52	-
YML013W	81.250214	67.669876	0.263857868	1.9749054274e-52	6.80366975209e-52	-
YER088C	119.361214	161.994522	-0.440610914	2.13247071474e-52	7.34241719197e-52	-

YGL047W	129.72171	96.729225	0.423396202	2.34405488808e-52	8.06645939312e-52	-
YKL012W	53.869053	81.528572	-0.597849043	2.61934828127e-52	9.00881669259e-52	-
YCL044C	42.137814	70.059807	-0.733471541	2.98138820525e-52	1.02483156025e-51	-
YLL005C	12.293265	6.990349	0.814431745	3.71650446387e-52	1.2768157007e-51	-
YGR038C-B	14.924308	11.133252	0.422788977	4.35073932734e-52	1.49388217611e-51	-
YMR092C	75.598351	108.618225	-0.522839522	5.08168538474e-52	1.74389733933e-51	-
YDR283C	9.563568	6.258358	0.611764775	5.28602066747e-52	1.81301747534e-51	-
YLR401C	10.867409	21.877647	-1.009449566	7.24877438297e-52	2.48483663001e-51	down
YJL014W	133.189819	182.630402	-0.45544314	7.46146527645e-52	2.55633424041e-51	-
YPR144C	19.622377	35.898293	-0.871415428	8.03827107687e-52	2.75243170685e-51	-
YJL140W	154.442169	121.863007	0.341806476	9.37959382313e-52	3.2099514032e-51	-
YLR157C-B	9.682318	6.477024	0.580021388	1.02341259094e-51	3.5004657656e-51	-
YMR168C	32.638222	53.006489	-0.699606517	1.07731035421e-51	3.68278781881e-51	-
YDL037C	10.526324	26.387289	-1.325841431	1.08854583165e-51	3.71914833401e-51	down
YMR230W	287.292145	221.421646	0.375722282	1.22565644051e-51	4.18529988047e-51	-
YHR005C	48.96769	35.572224	0.461078965	1.38212566626e-51	4.71700722162e-51	-
YDR362C	23.747768	40.071819	-0.754796076	1.89782220214e-51	6.4734506763e-51	-
YGL150C	21.445602	32.301304	-0.590910593	2.26834787608e-51	7.73306074393e-51	-
YNR035C	296.968262	273.046539	0.121161884	2.3418796846e-51	7.97935734468e-51	-
YGR125W	22.997438	36.181522	-0.653779949	2.94199185664e-51	1.00185877378e-50	-
YPR103W	466.050751	439.751343	0.0837990832669	3.04321771851e-51	1.03576181998e-50	-
YDR074W	287.382202	284.3862	0.0151192572793	3.20302644509e-51	1.08955551623e-50	-

YKL125W	23.558008	40.295246	-0.774392087	3.22637966158e-51	1.09689840849e-50	-
YHR209W	61.340942	41.773033	0.554278425	3.5351797179e-51	1.20122581766e-50	-
YAL015C	25.793007	14.574004	0.823582683	3.76881335257e-51	1.27991210573e-50	-
YCR068W	18.349546	34.44186	-0.908418688	3.98960976677e-51	1.35415513571e-50	-
YOL043C	30.385586	54.642227	-0.846629168	4.00265912684e-51	1.35784195953e-50	-
YIL035C	45.137928	75.340652	-0.739088324	4.06441375209e-51	1.37803826177e-50	-
YDR086C	258.501678	183.568176	0.493857676	4.70429486151e-51	1.59411913211e-50	-
YPL168W	24.713028	14.143056	0.805177902	4.86603380765e-51	1.64802716191e-50	-
YLR196W	15.852686	30.035431	-0.921938062	5.43709593085e-51	1.8404302911e-50	-
YJR082C	104.456383	186.686371	-0.837715954	5.77121651549e-51	1.95246387619e-50	-
YOL041C	11.677523	25.416267	-1.122017864	5.94253229836e-51	2.00932682507e-50	down
YAL044C	378.318176	522.928284	-0.467013013	6.2114816015e-51	2.09912236157e-50	-
YDR085C	63.149837	51.231117	0.301758663	7.69444356483e-51	2.59886320187e-50	-
YKL145W	344.703766	441.115448	-0.355799224	9.67788843529e-51	3.26701095195e-50	-
YGR146C	61.03706	107.03051	-0.81026473	1.25840040148e-50	4.24573352848e-50	-
YBR057C	78.885849	120.243126	-0.608115991	1.36532130692e-50	4.60397320662e-50	-
YNL075W	26.373629	52.014248	-0.979810768	1.53837564472e-50	5.18471009902e-50	-
YLL061W	27.866125	18.531414	0.588539431	1.586930695e-50	5.34545076211e-50	-
YCL033C	125.809204	89.983269	0.483508787	2.07365618625e-50	6.98115922137e-50	-
YDR210C-D	11.374137	8.017496	0.504533453	2.21631690273e-50	7.45739584399e-50	-
YNR021W	49.515842	34.916283	0.503990186	2.23641347388e-50	7.52093978647e-50	-
YPR018W	9.087109	3.739041	1.281153093	2.59674236534e-50	8.7279786703e-50	up

YDR476C	106.595863	167.028061	-0.64793905	2.83842878718e-50	9.53515471365e-50	-
YNL027W	47.734413	71.526703	-0.583452224	3.24267246608e-50	1.08872421143e-49	-
YOL069W	18.881676	9.901256	0.931303377	3.57027436812e-50	1.19806828526e-49	-
YKL210W	76.351852	68.425224	0.158134899	3.62700374811e-50	1.21644728624e-49	-
YJR074W	110.664612	81.810432	0.435837231	3.89396440356e-50	1.30527705277e-49	-
YNL136W	36.699078	24.177	0.602108579	4.23648220369e-50	1.41932442097e-49	-
YNL281W	152.306503	111.731606	0.446940197	4.27780401333e-50	1.43239521654e-49	-
YKL052C	68.843994	110.901505	-0.687876241	4.38663742209e-50	1.46804555883e-49	-
YJR032W	13.910635	29.951004	-1.106416087	4.62674434035e-50	1.54756621039e-49	down
YBL039W-B	137.953308	71.382095	0.950545903	4.94845688061e-50	1.65428219251e-49	-
YBL009W	10.076955	4.734759	1.08969686	4.97361160146e-50	1.66179659967e-49	up
YDR496C	16.240429	29.564377	-0.864270133	5.30213454502e-50	1.77061061084e-49	-
YBR073W	13.225111	8.156657	0.69722994	5.65020040428e-50	1.88583032848e-49	-
YJL074C	3.763703	1.377135	1.450482794	5.92585878794e-50	1.97677223834e-49	up
YDR321W	18.174252	36.642139	-1.011607736	8.21406305542e-50	2.73860920774e-49	down
YNR048W	30.340117	53.858711	-0.827953053	9.12871943701e-50	3.04192647692e-49	-
YOR275C	30.686441	49.303726	-0.684095346	9.46558568611e-50	3.15248690662e-49	-
YLR409C	5.803274	12.474242	-1.1040132	9.65688185903e-50	3.21447306064e-49	down
YJR049C	121.356857	167.341675	-0.463541154	1.0370735145e-49	3.45024243195e-49	-
YOR212W	25.047888	45.589867	-0.864024238	1.10023624555e-49	3.65841811055e-49	-
YLR222C	5.883572	13.102036	-1.155026811	1.25856658432e-49	4.18264526523e-49	down
YFR030W	14.5577	24.657049	-0.760217706	1.28141277553e-49	4.25629240797e-49	-

YDR456W	45.885788	69.673019	-0.602552695	1.61778788511e-49	5.37070972768e-49	-
YDR436W	78.44503	109.771935	-0.484755302	1.82418737804e-49	6.05267516989e-49	-
YLR057W	8.773467	4.343188	1.01439266	1.91360508685e-49	6.34597242478e-49	up
YJL225C	8.358209	5.426834	0.623083055	1.9624366688e-49	6.50443504534e-49	-
YML024W	272.207886	399.587524	-0.553802677	1.97732487252e-49	6.55028431624e-49	-
YOL117W	28.388992	19.665062	0.52969689	2.3036294221e-49	7.62716344129e-49	-
YGL107C	15.401603	28.392372	-0.882422867	2.39807605054e-49	7.93563759156e-49	-
YHR131C	13.524893	24.157894	-0.836877511	2.41330031214e-49	7.98176256675e-49	-
YJL044C	45.047821	32.209743	0.483960174	2.84541495021e-49	9.40592971827e-49	-
YBR267W	20.805737	40.096577	-0.946497687	3.02439014023e-49	9.99223735528e-49	-
YAL051W	31.527229	46.976475	-0.575340087	3.33363100388e-49	1.10080751447e-48	-
YNL245C	63.454449	113.466469	-0.838472798	3.36348888262e-49	1.11007650097e-48	-
YIL107C	57.988716	48.883015	0.246438924	4.90654660333e-49	1.61848253525e-48	-
YGL147C	30.490662	63.999481	-1.069692732	4.98482059612e-49	1.64342890391e-48	down
YLR114C	24.621605	39.96006	-0.69863394	5.25389528683e-49	1.73121984823e-48	-
YBL032W	32.18943	56.630585	-0.814994403	5.33415627935e-49	1.75673433327e-48	-
YML012W	243.421692	207.410507	0.230968756	5.70751037062e-49	1.8786969449e-48	-
YPL253C	5.584732	1.676338	1.736174981	5.76577597436e-49	1.89687001849e-48	up
YGR108W	20.498161	11.507184	0.832959659	8.35966547149e-49	2.74877135842e-48	-
YOR092W	4.26423	11.536203	-1.435811297	8.69782162234e-49	2.85844767768e-48	down
YHL047C	18.201691	32.537102	-0.838013271	9.32729590997e-49	3.06369592641e-48	-
YBR183W	121.452591	98.966713	0.295378	1.16927697565e-48	3.83864170534e-48	-

YDL155W	24.587822	44.617199	-0.859656	1.20076828532e-48	3.93994160426e-48	-
YDL080C	10.305589	21.176596	-1.039043742	1.24766774175e-48	4.09166473363e-48	down
YDR031W	191.76062	141.617661	0.437305285	1.37288962341e-48	4.49994655868e-48	-
YDR061W	33.307026	54.399776	-0.70777417	1.78790819894e-48	5.85716838998e-48	-
YNL238W	17.498516	29.815903	-0.768849454	1.80163705589e-48	5.89903103532e-48	-
YNL225C	9.666306	4.119381	1.230537099	1.85622295072e-48	6.07455565527e-48	up
YHR107C	43.824131	71.646851	-0.709177813	1.90472152696e-48	6.22998484689e-48	-
YLR005W	77.59761	62.540737	0.311215998	2.29095701809e-48	7.48934237403e-48	-
YJL010C	11.841139	22.92732	-0.953259866	2.62725123581e-48	8.58419772206e-48	-
YOR195W	5.488712	2.01754	1.443870364	3.26057949412e-48	1.06479103101e-47	up
YGL164C	49.699142	36.237816	0.455724937	3.63764890356e-48	1.18730412162e-47	-
YDR532C	40.021313	26.639555	0.587198516	4.71744567864e-48	1.53893340899e-47	-
YDR409W	22.305687	35.661854	-0.676970125	4.76761962884e-48	1.55448438319e-47	-
YAR014C	19.831511	33.81786	-0.769990765	4.92257688671e-48	1.6041657382e-47	-
YBR002C	48.328426	82.637474	-0.773924147	5.91050394964e-48	1.92510013218e-47	-
YBL101C	65.002449	88.247345	-0.441058801	6.8217667368e-48	2.22074084437e-47	-
YBR295W	10.64972	18.446354	-0.79252019	6.9116786181e-48	2.24883128203e-47	-
YBR154C	58.340164	101.618034	-0.800595111	7.15964279538e-48	2.32829033388e-47	-
YIL138C	134.275894	97.923248	0.45547701	8.30280357274e-48	2.69862851202e-47	-
YOR237W	1.290352	7.076249	-2.455220137	9.31280256927e-48	3.02532068812e-47	down
YPR083W	101.267181	140.360809	-0.470973472	1.0505400622e-47	3.41095852831e-47	-
YKR034W	14.926866	34.631767	-1.214184706	1.16064948418e-47	3.76649869198e-47	down

YOR099W	94.669044	138.205231	-0.545847564	1.5450267764e-47	5.01124672305e-47	-
YOR108W	3.544665	10.22633	-1.528567295	1.87071583784e-47	6.06444068996e-47	down
YCR028C	10.12357	21.780128	-1.105294297	1.9616476048e-47	6.35590205145e-47	down
YER079W	351.544464	315.141327	0.157708217	2.01777058688e-47	6.53433479568e-47	-
YNL248C	9.691649	22.432467	-1.210774228	3.2883189508e-47	1.06433180639e-46	down
YLR116W	71.636894	105.654366	-0.560577694	3.51055028241e-47	1.13566941914e-46	-
YPR101W	76.488083	49.069336	0.640413241	3.83197578495e-47	1.2390055038e-46	-
YHR039C	34.301689	53.857521	-0.65086821	4.44289721573e-47	1.43578895967e-46	-
YOR245C	144.456482	199.259003	-0.464009969	6.13783978912e-47	1.9825030911e-46	-
YLR430W	4.856691	2.898278	0.744777716	6.36672503354e-47	2.05536292295e-46	-
YOR369C	358.448151	502.849609	-0.488362537	6.50815184086e-47	2.09992757942e-46	-
YMR118C	50.594883	92.140434	-0.86484291	7.0381697376e-47	2.26976403798e-46	-
YMR164C	11.426089	21.467129	-0.909797586	7.79913081721e-47	2.51386314191e-46	-
YKL062W	31.698271	50.54372	-0.6731277	8.1060376043e-47	2.61143131539e-46	-
YPL202C	21.034824	39.406239	-0.905644316	8.49727282742e-47	2.73605133364e-46	-
YPR010C	10.097221	17.628082	-0.80391723	9.19328495389e-47	2.95862690481e-46	-
YDR520C	12.994592	23.608545	-0.861397793	1.12247818643e-46	3.61054123388e-46	-
YBR147W	231.8172	206.454727	0.167162162	1.19227878844e-46	3.83307442706e-46	-
YGR127W	52.994793	36.307842	0.54556943	1.21201714482e-46	3.89451471793e-46	-
YNL308C	34.694862	55.029064	-0.665471761	1.23717622708e-46	3.97330057822e-46	-
YJR007W	121.111282	176.584869	-0.544028464	1.27391380678e-46	4.08917110264e-46	-
YCR084C	28.268906	44.934883	-0.668619793	1.81953326733e-46	5.83755169178e-46	-

YMR078C	9.588939	4.793225	1.000374529	1.88447177643e-46	6.04276900209e-46	up
YPL101W	33.645512	56.054874	-0.736425746	2.1801334752e-46	6.98723211877e-46	-
YDL092W	101.662819	170.253525	-0.743892528	2.29442187224e-46	7.34972702934e-46	-
YBL054W	28.083939	47.120419	-0.746607068	2.37088164815e-46	7.59073402357e-46	-
YMR143W	287.092255	238.528061	0.267355413	2.37348803205e-46	7.59516170255e-46	-
YLR071C	22.650963	34.898647	-0.623598718	2.58931133935e-46	8.28152745734e-46	-
YDL100C	206.574234	185.491547	0.155306875	2.65766882566e-46	8.49578170427e-46	-
YCL031C	41.71891	72.369568	-0.794681695	2.90225052529e-46	9.2728622033e-46	-
YHR134W	29.627287	56.568012	-0.933059947	2.93016569198e-46	9.35723694228e-46	-
YGL090W	28.894581	50.308865	-0.800013692	3.02327950372e-46	9.64962424631e-46	-
YDR221W	29.153767	21.144094	0.463427564	3.39901203968e-46	1.08433025397e-45	-
SCR1	13.407468	37.101955	-1.468458398	3.96824265286e-46	1.26527223364e-45	down
YGR004W	8.198911	19.197584	-1.227420555	4.18195751209e-46	1.33273060755e-45	down
YNL200C	495.840851	468.585724	0.0815641345863	4.45624923509e-46	1.41941484102e-45	-
YIL145C	48.967789	81.669739	-0.73796856	4.51289414908e-46	1.43672035269e-45	-
YFR024C-A	95.513092	136.150726	-0.511434273	4.59264384451e-46	1.46135996857e-45	-
YNR039C	20.404144	35.385555	-0.79429836	5.6477385556e-46	1.79616603244e-45	-
YBR294W	6.024049	12.841896	-1.09205281	6.14681425238e-46	1.95388750019e-45	down
YIL177C	8.095977	5.334357	0.601890811	6.18860383771e-46	1.96616441272e-45	-
YNL141W	22.37727	42.911297	-0.939323467	6.19188618015e-46	1.96620099265e-45	-
YLR276C	18.869478	33.344448	-0.821392053	7.54251593878e-46	2.39386190941e-45	-
YNL126W	7.754622	3.748268	1.04883235	8.73741358673e-46	2.77168439174e-45	up

YLR081W	9.075337	3.833969	1.243112545	9.53894624168e-46	3.02440134159e-45	up
YHL002W	96.644547	137.686646	-0.510628403	9.69001139254e-46	3.07072949081e-45	-
YBR121C	37.299831	57.225155	-0.617480372	1.06617113507e-45	3.37693388086e-45	-
YKL215C	11.074934	7.316268	0.598118273	1.31704392524e-45	4.16940779597e-45	-
YPL119C	172.223709	225.289871	-0.387498688	1.43435883508e-45	4.53848096237e-45	-
YER056C-A	216.893127	329.906647	-0.605073509	1.52523456296e-45	4.82356401774e-45	-
YER033C	31.297161	46.018532	-0.556183167	1.61688508786e-45	5.11080581744e-45	-
YBR156C	9.181848	4.397801	1.062002225	1.89354409552e-45	5.9822502519e-45	up
YOR106W	33.467476	61.141296	-0.869387381	1.93787054549e-45	6.11917616805e-45	-
YOL063C	11.303446	20.077713	-0.828832281	2.10494690306e-45	6.64337080538e-45	-
YKR089C	127.822487	166.016281	-0.377183069	2.20270201698e-45	6.94836083404e-45	-
YKL088W	47.230782	71.519234	-0.598603862	2.91708030237e-45	9.19717344697e-45	-
YDL053C	117.107208	183.117661	-0.644941063	3.50785133097e-45	1.10541832805e-44	-
YJL099W	14.014823	8.382044	0.741579524	4.12041800548e-45	1.29779578783e-44	-
YPR022C	21.009731	32.375687	-0.623853116	4.22318848337e-45	1.32949057326e-44	-
YBL022C	56.919506	77.676628	-0.448557433	4.75709103797e-45	1.49680796572e-44	-
YDR324C	12.029924	22.016605	-0.871964492	4.8489781553e-45	1.52494713212e-44	-
YBR275C	3.641492	1.833162	0.99019539	5.11018503267e-45	1.60627993331e-44	-
YIL005W	41.983154	62.739655	-0.579567042	5.93778728561e-45	1.86547487192e-44	-
YJR070C	32.396847	57.725353	-0.83335168	6.89255586941e-45	2.16433924316e-44	-
YGR010W	71.769112	56.237766	0.351823785	7.96184238769e-45	2.49884315181e-44	-
YMR113W	15.946704	31.384243	-0.976782145	8.08788336048e-45	2.53711874188e-44	-

YLR233C	13.756636	7.989137	0.784016147	8.14367635725e-45	2.55333044575e-44	-
YLR320W	6.925408	12.453959	-0.846633462	8.58506009331e-45	2.69036108326e-44	-
YDL169C	354.830475	474.763184	-0.420078142	1.00671425904e-44	3.15322004041e-44	-
YDR261C-D	16.434784	12.693178	0.372699172	1.06422890505e-44	3.33168585101e-44	-
YDR219C	53.660282	81.97467	-0.611323552	1.26434422434e-44	3.95617386324e-44	-
YOR165W	18.942122	31.564602	-0.736709602	1.35200342413e-44	4.22833111185e-44	-
YGL185C	63.801907	97.903534	-0.617761391	1.63033233039e-44	5.09622512944e-44	-
YNR050C	26.45261	16.588045	0.67326621	1.7007908466e-44	5.31379445179e-44	-
YLR442C	12.419083	7.8621	0.659572033	1.88354809291e-44	5.88182422574e-44	-
YLR423C	22.630903	13.096947	0.789063603	1.88487778573e-44	5.88301724172e-44	-
YDR441C	50.400322	92.318619	-0.873188692	2.12973414338e-44	6.64391435281e-44	-
YDR347W	51.115837	83.431587	-0.706823344	2.19003923702e-44	6.82861053912e-44	-
YIL027C	71.119659	42.182224	0.753613246	2.37933876822e-44	7.41512804875e-44	-
YNL250W	5.42333	2.781765	0.96317841	2.66323772535e-44	8.29572493677e-44	-
YMR098C	24.880835	41.147511	-0.725770262	3.10853253107e-44	9.67791873264e-44	-
YKL010C	34.124523	47.641106	-0.481398026	3.26018896164e-44	1.01449890095e-43	-
YOR267C	57.959698	82.172081	-0.503598226	3.55487551071e-44	1.10564464782e-43	-
YPL074W	41.601822	33.305046	0.32090594	3.82035175681e-44	1.18761861323e-43	-
YNR001C	1232.715698	1278.271484	-0.052354165	3.94770425593e-44	1.22659399503e-43	-
YDL122W	22.78013	36.412247	-0.676647792	5.24325635646e-44	1.62832093351e-43	-
YGR138C	10.424184	20.73632	-0.992225432	7.09499423522e-44	2.20228621061e-43	-
YMR175W	2770.860352	3447.460938	-0.315200203	7.33379677207e-44	2.27527288161e-43	-

YPR164W	9.335008	15.837402	-0.762612528	8.80695382231e-44	2.73094751893e-43	-
YBR256C	168.664993	140.977936	0.25869118	9.41976896074e-44	2.9195170099e-43	-
YOR198C	33.504822	54.920147	-0.712966744	1.27149861222e-43	3.93885398438e-43	-
YLR186W	14.245794	33.307419	-1.22530753	1.30888310691e-43	4.05264155995e-43	down
YOR202W	54.810799	94.398148	-0.78429839	1.36481059709e-43	4.2237009904e-43	-
YOR386W	61.443981	50.18861	0.291911702	1.41546987286e-43	4.37829445475e-43	-
YPL218W	265.680969	229.627716	0.210398104	1.44316542015e-43	4.46173851011e-43	-
YGR165W	53.890518	85.847977	-0.671752684	1.44481370622e-43	4.46461099462e-43	-
YPR075C	325.921509	308.530975	0.0791092288689	1.54838625793e-43	4.78227954688e-43	-
YGR128C	6.15015	13.525146	-1.136950665	1.57328761721e-43	4.856772515e-43	down
YER074W	195.890579	295.613007	-0.593661734	1.69432673823e-43	5.22782325593e-43	-
YNL096C	81.092209	133.343536	-0.717512672	1.74435018013e-43	5.3794962336e-43	-
YOR166C	7.852469	18.325912	-1.222666749	1.78287378739e-43	5.49557123741e-43	down
YKR046C	817.316711	811.985474	0.00944131383627	1.96026756682e-43	6.0393752133e-43	-
YAL010C	20.361654	12.168629	0.742688124	2.27727043439e-43	7.01254705193e-43	-
YML117W	81.547043	107.421135	-0.397573401	2.29250146311e-43	7.0559489752e-43	-
YHR090C	70.001541	110.351379	-0.656646072	2.37497824696e-43	7.30617688657e-43	-
YGL091C	75.025551	114.360184	-0.608130932	2.44130685549e-43	7.50650468494e-43	-
YBR155W	22.564737	41.599228	-0.882486792	2.48413787983e-43	7.63441978118e-43	-
YBR062C	165.212936	131.487457	0.32940147	2.58812999316e-43	7.95007966232e-43	-
YLR383W	6.627429	3.494892	0.923201436	2.64122260454e-43	8.10915426756e-43	-
YDL019C	41.811169	36.456493	0.197712595	2.74118963328e-43	8.41191559238e-43	-

YJL050W	8.180356	15.038849	-0.87845862	2.92078379446e-43	8.958609583e-43	-
YNL110C	44.437653	79.731583	-0.843368691	3.80733989468e-43	1.1672082008e-42	-
YER027C	65.670624	98.440613	-0.584005478	6.17115145538e-43	1.89094315079e-42	-
YGL232W	30.237658	17.202398	0.813736713	6.95876052949e-43	2.13122769448e-42	-
YDR411C	84.444878	66.93869	0.335169602	8.06463639658e-43	2.46870131903e-42	-
YNL106C	6.891163	12.855248	-0.899538055	8.43001193039e-43	2.57927619832e-42	-
YJL031C	48.931175	34.035339	0.523720448	8.48187731526e-43	2.59386671789e-42	-
YLR035C-A	5.391615	2.628096	1.036699506	9.18566019873e-43	2.80770942953e-42	up
YKR074W	191.982513	153.248062	0.325106078	9.99050169866e-43	3.05221626699e-42	-
YPL278C	44.190018	17.601231	1.328044187	1.01522464455e-42	3.10010555501e-42	up
YDL218W	10.987039	25.734449	-1.22789826	1.13563599576e-42	3.4660905908e-42	down
YPR102C	105.906456	168.282822	-0.668097379	1.3064760528e-42	3.98555446476e-42	-
YHR037W	104.674431	142.985535	-0.44996013	1.47819096165e-42	4.50717558444e-42	-
YHL023C	11.092945	18.829418	-0.763345979	1.49067128493e-42	4.54299820168e-42	-
YPL148C	34.58931	16.622772	1.05716525	1.82883640162e-42	5.57086181613e-42	up
YCL057W	26.447088	19.204535	0.461661849	2.06689760166e-42	6.29293786714e-42	-
YPR141C	8.079084	3.845353	1.071075688	2.09215003315e-42	6.36669970873e-42	up
YPL196W	171.870239	147.67392	0.218904689	2.13134203675e-42	6.48278851747e-42	-
YFL004W	55.006042	77.276657	-0.490442588	2.8980090071e-42	8.810401526e-42	-
YKR030W	28.741152	53.868748	-0.906330646	3.15359305587e-42	9.58272427353e-42	-
YLR017W	53.86076	85.650887	-0.6692336	3.59368960185e-42	1.09146893583e-41	-
YMR121C	84.223671	135.070786	-0.681418009	4.23012116901e-42	1.28413653874e-41	-

YKL149C	49.814117	36.834438	0.43549942	4.24107205953e-42	1.28683163957e-41	-
YKL205W	6.164186	11.953171	-0.955411095	4.49357863116e-42	1.3627814432e-41	-
YDL206W	19.817289	32.514359	-0.714317367	4.63164181787e-42	1.40396642604e-41	-
YIL159W	4.673454	2.337907	0.999271652	4.98377248367e-42	1.50996874469e-41	-
YJR139C	168.190048	229.359863	-0.447520606	5.03248087448e-42	1.52398250092e-41	-
YML124C	31.004837	20.795574	0.576216798	5.06913396361e-42	1.53433367363e-41	-
YDR356W	7.650961	4.050109	0.917680234	5.57960783383e-42	1.68802170723e-41	-
YNL253W	19.190834	10.715538	0.840713124	6.88770860902e-42	2.08275182878e-41	-
YBR007C	12.348142	7.204927	0.77723826	7.29358509054e-42	2.20440974889e-41	-
YJR050W	113.203804	169.693558	-0.58400936	8.97962692581e-42	2.71267756474e-41	-
YJR086W	116.969162	77.122208	0.600909963	9.12758524636e-42	2.75603352186e-41	-
YLR241W	37.14267	55.022484	-0.566943745	9.17912336319e-42	2.77024782881e-41	-
YGR112W	38.587936	63.27169	-0.713410252	9.52914313e-42	2.87448593543e-41	-
YFL038C	977.602966	965.64978	0.0177486121645	1.04762380654e-41	3.15864428897e-41	-
YNR049C	58.845428	99.976471	-0.764658274	1.07682193374e-41	3.24510221584e-41	-
YOR096W	107.329651	167.235977	-0.639836552	1.13526497011e-41	3.41956571297e-41	-
YIL071C	25.651312	44.26215	-0.787040913	1.19128987793e-41	3.58657980709e-41	-
YIL031W	14.820621	24.211851	-0.708107478	1.19255882816e-41	3.58865981834e-41	-
YBL066C	112.25174	143.81871	-0.357513566	1.35157799896e-41	4.06521134569e-41	-
ITS1-2	99.298126	169.828918	-0.774243742	1.50867743061e-41	4.53553001901e-41	-
YBR142W	9.737204	18.359734	-0.914965684	1.71771989662e-41	5.16147391975e-41	-
YLR299W	48.769714	71.221527	-0.546327856	1.72612029285e-41	5.18420647219e-41	-

YKL191W	41.130856	63.315296	-0.622332978	1.75373663513e-41	5.26460204588e-41	-
YLR251W	146.953003	117.362442	0.324384046	1.83251788103e-41	5.49843934531e-41	-
YHR207C	33.896984	24.445221	0.471604466	2.2661998939e-41	6.79641011659e-41	-
YGL086W	7.072907	3.226258	1.132441478	2.56381840784e-41	7.68526541568e-41	up
YPL068C	6.078412	17.766184	-1.547367468	4.06806224758e-41	1.21884799387e-40	down
YBR222C	324.799469	318.402222	0.0286988700276	4.27629080347e-41	1.28061810458e-40	-
YBR035C	433.253632	406.237457	0.0928885746441	5.17374309787e-41	1.54863052804e-40	-
YHR115C	61.083691	91.881752	-0.588991126	6.17048530531e-41	1.84609025423e-40	-
YOL164W	12.304399	22.839201	-0.892337989	6.70952575394e-41	2.00639382854e-40	-
YNL212W	46.128231	66.305794	-0.523484976	6.84840777893e-41	2.04693863705e-40	-
YIL062C	467.355499	425.053192	0.136876975	7.06543172776e-41	2.11078922839e-40	-
YNR027W	18.040049	36.216747	-1.005453712	8.01782823433e-41	2.39416439051e-40	down
YOR045W	218.875793	147.081467	0.573496934	8.55758639475e-41	2.55411040089e-40	-
YPL270W	56.925152	79.934128	-0.489745357	1.03187574146e-40	3.07827227437e-40	-
YOR207C	24.060575	35.759068	-0.571638015	1.04165141841e-40	3.10594236574e-40	-
YHR057C	171.450821	142.029984	0.271599283	1.12896684841e-40	3.36467892219e-40	-
YBR269C	984.077576	948.101562	0.0537304384401	1.1990182438e-40	3.57173956694e-40	-
YLR117C	56.704018	80.691307	-0.508962291	1.33026643765e-40	3.96081249157e-40	-
YML020W	17.289349	29.631041	-0.777225764	1.71547353047e-40	5.10530185864e-40	-
YBR058C	42.7631	61.958675	-0.534939851	1.95996743235e-40	5.83012832776e-40	-
YDR104C	12.179689	19.822239	-0.702642634	2.05460566171e-40	6.10871261872e-40	-
YPR115W	54.853493	74.641838	-0.444401019	2.15470157939e-40	6.40324911672e-40	-

YKL044W	29.322317	69.727119	-1.249720766	2.80206113032e-40	8.32306004643e-40	down
YKL048C	14.158661	25.435978	-0.845185732	2.84754478746e-40	8.45411670997e-40	-
YOR228C	184.697647	163.420868	0.176573267	2.95768648158e-40	8.77692049601e-40	-
YLL011W	10.646325	21.80983	-1.034623012	3.32093420519e-40	9.85014789577e-40	down
YML042W	225.046204	284.731781	-0.339382302	3.35414642522e-40	9.9439068805e-40	-
YJL122W	25.010544	53.679466	-1.101833882	3.46051639577e-40	1.02543607565e-39	down
YER154W	38.690067	62.60672	-0.694354293	3.76271516506e-40	1.11445304125e-39	-
YBR280C	183.841721	175.234406	0.0691781432604	4.15302439562e-40	1.22946950158e-39	-
YPR042C	25.20723	37.474453	-0.572069829	4.59018671956e-40	1.35824018852e-39	-
YKL008C	34.742435	23.984722	0.534583154	4.84400984127e-40	1.43266379679e-39	-
YBR111C	358.752899	331.724365	0.113005509	5.42721497453e-40	1.60438812199e-39	-
YNL257C	8.045961	4.938582	0.70416788	6.07010036021e-40	1.7935832002e-39	-
YJL163C	58.449684	84.84935	-0.537708383	6.09150391252e-40	1.79905120309e-39	-
YHR016C	150.59169	136.958145	0.136907094	6.11499099746e-40	1.80512905907e-39	-
YER096W	30.705873	21.66963	0.502840101	6.72874497998e-40	1.98536353782e-39	-
YNL305C	627.389099	783.485168	-0.320545501	7.28561611485e-40	2.14865106133e-39	-
YDR030C	51.644661	40.80938	0.339718419	1.12381075645e-39	3.31273370182e-39	-
YBR223C	24.862175	41.110214	-0.72554437	1.57402102989e-39	4.63764715404e-39	-
YER117W	281.530914	399.732849	-0.505742786	1.67232953879e-39	4.92496289222e-39	-
YAL063C	20.697884	30.66679	-0.567193878	1.88534727994e-39	5.54966141008e-39	-
YCR093W	12.706167	10.097822	0.331484735	2.09164303105e-39	6.15399049135e-39	-
YNL032W	64.406174	101.294945	-0.653291283	2.13721431321e-39	6.28509069466e-39	-

YPL107W	96.639824	145.670715	-0.592021141	2.51434770936e-39	7.39065841841e-39	-
YDR536W	16.044353	9.61676	0.738442793	2.56596764057e-39	7.53882021423e-39	-
YGR270W	33.394436	46.481449	-0.477047295	2.59651711291e-39	7.62496605341e-39	-
YKL092C	6.191119	3.304271	0.905868175	2.92090655465e-39	8.57351673345e-39	-
YBR009C	702.386536	935.702148	-0.413784175	2.9562740174e-39	8.67322736295e-39	-
YBR230W-A	348.515564	269.665558	0.370051815	3.04787283187e-39	8.93773950885e-39	-
YOR119C	26.261965	17.572729	0.579636611	3.28936328272e-39	9.64134431497e-39	-
YDR473C	24.50602	15.897019	0.624379939	3.59971376389e-39	1.05460231459e-38	-
YNR038W	7.098918	15.047316	-1.083835121	3.96163695766e-39	1.16008689779e-38	down
YCL008C	23.390905	13.997385	0.740790354	4.32375483713e-39	1.26552899712e-38	-
YOR110W	19.153103	34.783516	-0.860825628	4.45984051111e-39	1.30474504679e-38	-
YDR524C	24.418812	41.336838	-0.759435023	4.66468238007e-39	1.36402959093e-38	-
YPL217C	10.628798	17.771772	-0.741609085	5.4903626915e-39	1.6047161765e-38	-
YBR296C-A	67.628471	167.289917	-1.306647851	5.83119228557e-39	1.70353137453e-38	down
YER055C	121.714798	102.017403	0.2546893	7.03065727717e-39	2.05297838084e-38	-
YNL221C	10.438251	18.568939	-0.831011386	7.08549960754e-39	2.06801981963e-38	-
LSR1	33.297905	55.274673	-0.731187175	7.1603661716e-39	2.08888877788e-38	-
YBR137W	146.180313	116.108658	0.332273473	7.90136343431e-39	2.30397671208e-38	-
YGR072W	25.900345	16.129189	0.683297416	7.9758516504e-39	2.32460502562e-38	-
YPL167C	12.157079	19.052448	-0.648179746	7.98664749508e-39	2.32665920457e-38	-
YLR210W	7.197427	2.489538	1.531603215	8.77268826224e-39	2.55444881482e-38	up
YER075C	3.587073	8.259642	-1.203272146	9.20809384801e-39	2.67997405572e-38	down

YLL032C	17.504477	12.170941	0.524283243	9.43881910251e-39	2.74583828437e-38	-
YDL140C	36.18198	48.784176	-0.431141901	9.54782588429e-39	2.77624838827e-38	-
YLL012W	7.957426	16.722168	-1.071388162	9.91329768591e-39	2.88116816639e-38	down
YDR167W	93.113762	144.662354	-0.635623217	1.14752139326e-38	3.33355770209e-38	-
YPL084W	37.351749	54.172981	-0.536397682	1.48526846856e-38	4.31269721835e-38	-
YMR271C	199.743484	173.143951	0.206176454	1.5741114916e-38	4.56852928463e-38	-
YBR087W	17.776754	9.213619	0.948152069	1.76913700391e-38	5.13215071042e-38	-
YDR494W	131.14476	181.554214	-0.469240252	1.89106924373e-38	5.48330586878e-38	-
YFR016C	13.85745	10.078115	0.459435978	1.93610308491e-38	5.6112642162e-38	-
YOR360C	33.961231	53.307205	-0.650441787	2.3008638776e-38	6.66531169022e-38	-
YER072W	564.089905	749.581909	-0.410161016	2.55105069861e-38	7.38662441091e-38	-
YDR176W	68.040352	94.082855	-0.467541236	2.65236239918e-38	7.67639430028e-38	-
YPL009C	26.944592	21.706907	0.311841569	2.96470193218e-38	8.57636048227e-38	-
YER147C	29.382982	21.74855	0.4340616	3.11337792727e-38	9.00225904633e-38	-
YDL036C	26.822546	44.789261	-0.739706678	3.31351649935e-38	9.57649461264e-38	-
YLR309C	16.417484	11.57007	0.504835456	3.54361855549e-38	1.02367538355e-37	-
YER009W	228.622589	185.095276	0.304699881	3.79961449994e-38	1.09711659608e-37	-
YDL003W	6.943776	2.754207	1.334083415	3.82611193692e-38	1.10425397045e-37	up
YOR075W	30.308819	19.290461	0.651850017	4.02548685723e-38	1.16125568818e-37	-
YGL142C	12.698359	23.245417	-0.872304236	4.14328805065e-38	1.19468333574e-37	-
YNL201C	19.315577	30.643597	-0.665820879	4.70008991134e-38	1.35460344334e-37	-
YDR351W	21.660275	33.705505	-0.637932681	5.20411526349e-38	1.49917158031e-37	-

YBL041W	342.499512	318.225403	0.106052926	5.30646724275e-38	1.52794752519e-37	-
YOR293W	261.045654	384.534912	-0.558812446	5.33564545126e-38	1.53563685496e-37	-
YDR296W	181.041763	254.155121	-0.489386761	5.93322417806e-38	1.70683297949e-37	-
YCR017C	11.328465	7.281818	0.637581802	6.49251126436e-38	1.86686011714e-37	-
YPR057W	21.037079	39.214527	-0.898453799	6.94134730787e-38	1.99499463367e-37	-
YGR049W	97.46302	152.069244	-0.641801566	7.20532614613e-38	2.06990581745e-37	-
YPL222W	204.298798	258.596252	-0.340020649	7.44916830139e-38	2.13896562512e-37	-
YMR283C	14.131753	26.262072	-0.894040306	9.88086388533e-38	2.83589472955e-37	-
YOR020W-A	241.359787	186.10556	0.375064171	1.00679604083e-37	2.88825777332e-37	-
YML102W	34.296795	24.67745	0.47488044	1.05553055451e-37	3.02666682789e-37	-
YLR421C	420.225037	381.891876	0.137997891	1.16848654493e-37	3.34901406782e-37	-
YKR031C	26.990711	37.44812	-0.472430312	1.18816195472e-37	3.40383452464e-37	-
YLL015W	29.067493	25.273031	0.201807951	1.2424320621e-37	3.55766524054e-37	-
YOL154W	25.095194	13.12608	0.934974968	1.29805360533e-37	3.71522212165e-37	-
YGR062C	18.075287	35.486557	-0.973254053	1.47758799064e-37	4.22712730226e-37	-
YER123W	20.912848	35.600468	-0.767506659	1.48663457178e-37	4.25104901962e-37	-
YPL081W	18.707033	41.337616	-1.143874429	1.55609160943e-37	4.44761358717e-37	down
YKR099W	8.075881	15.339304	-0.925541467	2.01515888619e-37	5.75706689622e-37	-
YDR276C	2845.655762	2787.726807	0.0296719638889	2.13976323259e-37	6.11023465865e-37	-
YPL258C	7.15291	15.50175	-1.115828897	2.39252515579e-37	6.82887180098e-37	down
YBR139W	221.079254	211.308319	0.0652140836359	2.83187094407e-37	8.07916122279e-37	-
YGL122C	235.998535	300.817047	-0.350108422	2.93618258071e-37	8.37290834223e-37	-

YML014W	22.41729	11.772536	0.929186749	3.22288264572e-37	9.18625136117e-37	-
YML078W	279.748901	247.332397	0.177681239	3.39347730214e-37	9.66806199711e-37	-
YPR170W-B	268.242798	209.370941	0.357478215	3.83466204382e-37	1.09199917284e-36	-
YBR210W	68.243324	42.695404	0.676607142	4.02226953241e-37	1.14489909478e-36	-
YOR315W	5.964813	1.310459	2.186404692	4.35453286065e-37	1.23890650774e-36	up
YJR113C	144.369446	205.278442	-0.50781668	4.35865019154e-37	1.23950986665e-36	-
YMR178W	137.72847	117.076691	0.234372936	5.97518314987e-37	1.69844033857e-36	-
YER090W	37.085632	57.392136	-0.629992714	6.17432174143e-37	1.75424207647e-36	-
YOR327C	1283.935059	1617.101929	-0.332838384	7.29141522407e-37	2.0706818715e-36	-
YJR115W	44.047447	80.869934	-0.876545031	7.96362792312e-37	2.260548795e-36	-
YAL020C	26.327566	46.634647	-0.824828048	1.1559212561e-36	3.279688829e-36	-
YOR176W	119.95414	105.216171	0.189126497	1.27446241882e-36	3.61437309093e-36	-
YPR158W-B	6.082654	3.942154	0.625716805	1.45590497159e-36	4.12705847653e-36	-
YDR365C	21.151031	34.585529	-0.709440534	1.47989521725e-36	4.19314902269e-36	-
YJR014W	202.513489	173.978043	0.219112765	1.54574351641e-36	4.37772616327e-36	-
YDR097C	8.259264	5.288053	0.643276589	1.55902474364e-36	4.41332677086e-36	-
YGR247W	73.952065	54.924683	0.429135795	1.67861272833e-36	4.74969362693e-36	-
YDR363W-A	439.434265	376.57547	0.222708342	1.88260492084e-36	5.32446986268e-36	-
YPR194C	9.622436	17.162611	-0.834794975	2.00313309219e-36	5.66277333165e-36	-
YOR054C	70.717514	97.282707	-0.460115815	2.03549802829e-36	5.75164850233e-36	-
YLR195C	22.998629	39.25095	-0.771179711	2.06528902558e-36	5.83317300765e-36	-
YDR358W	169.375107	159.90535	0.0830036495711	2.22588269109e-36	6.28389256311e-36	-

YGR089W	7.341745	13.697371	-0.899704105	2.31984860891e-36	6.54619098369e-36	-
YPL171C	27.856651	18.346745	0.602497695	2.33156183785e-36	6.57625437955e-36	-
YOL151W	117.163071	100.722931	0.218125743	2.81087997581e-36	7.9245880517e-36	-
YLR271W	40.63969	26.786018	0.601409271	3.15520814494e-36	8.89129921187e-36	-
YDL219W	28.917713	12.683038	1.1890531	3.30522974366e-36	9.30983042134e-36	up
YDR432W	677.51239	824.973816	-0.284101	3.58628938399e-36	1.00969090684e-35	-
YGL158W	0.608057	3.974823	-2.708612143	3.91004378083e-36	1.10034232962e-35	down
YER166W	16.081699	23.785234	-0.564646386	4.01373049026e-36	1.12900946459e-35	-
YDR259C	20.186377	36.605946	-0.858696006	4.61930614969e-36	1.29876143919e-35	-
YBR055C	12.913774	21.574934	-0.740445462	4.68594192284e-36	1.3169002923e-35	-
YLR234W	11.681768	6.782977	0.784268132	4.93564247184e-36	1.38644653689e-35	-
YGR153W	17.40383	7.033448	1.307100811	5.93151007996e-36	1.66543711336e-35	up
YPL133C	66.176857	95.871254	-0.534771529	5.99086783195e-36	1.6813430154e-35	-
YIL026C	11.049151	7.581685	0.543345097	6.25329859957e-36	1.75420143272e-35	-
YIR014W	33.036732	59.373386	-0.845745409	6.69268865773e-36	1.87661297142e-35	-
YOR058C	17.625277	12.753275	0.466778157	7.38029139617e-36	2.06848076693e-35	-
YIL043C	266.730652	247.28508	0.109208428	9.18882690218e-36	2.57419843902e-35	-
YDL143W	63.375492	90.376945	-0.512029748	1.0488263136e-35	2.93690291153e-35	-
YDR072C	50.643745	74.2332	-0.551680468	1.1674632205e-35	3.26763375693e-35	-
YOR131C	72.157791	52.452065	0.460155602	1.18256577391e-35	3.30841294477e-35	-
YCL061C	3.57709	1.488451	1.264974689	1.30996808434e-35	3.66319003045e-35	up
YOL140W	25.961685	43.706871	-0.751476076	1.42508339409e-35	3.98330378682e-35	-

YKR057W	173.877457	276.702118	-0.670262786	1.44701052859e-35	4.04277288996e-35	-
YJL047C	28.08618	41.821243	-0.574375525	1.47627460611e-35	4.12267780241e-35	-
YPL083C	13.850636	26.06039	-0.91190645	1.50241698095e-35	4.19379704035e-35	-
YML025C	68.662422	104.945793	-0.612051681	1.51888095399e-35	4.2378485224e-35	-
YLL031C	12.145363	20.012117	-0.720468182	1.60112091554e-35	4.465300379e-35	-
YNL071W	342.706543	337.858887	0.0205529321525	1.64759725459e-35	4.59285305635e-35	-
YOL097C	23.892984	40.680649	-0.767755652	1.81887020304e-35	5.06801894995e-35	-
YDR060W	9.688068	16.622694	-0.778873319	1.84101328517e-35	5.12741609437e-35	-
YJR039W	16.12406	12.112027	0.41277473	1.97058203701e-35	5.48581761694e-35	-
YPL118W	112.780342	157.983368	-0.486257061	2.25903307748e-35	6.28600508516e-35	-
YKL032C	399.450958	402.210999	-0.009934145	2.28250969635e-35	6.34848575043e-35	-
YNL102W	3.599459	1.787092	1.010166179	2.32452990179e-35	6.4624637843e-35	up
YLR024C	6.475491	4.409457	0.554388581	2.51142980115e-35	6.97894190041e-35	-
YOR262W	46.157532	72.707825	-0.655544552	2.55239722669e-35	7.08961162563e-35	-
YGR231C	341.34906	326.24118	0.0653088803787	2.6598489694e-35	7.38476851613e-35	-
YKR010C	12.859004	8.243111	0.641518075	2.76338659647e-35	7.66879928069e-35	-
YBR225W	33.131546	47.829224	-0.52968686	3.10401425593e-35	8.61024151063e-35	-
YDR502C	19.679956	35.642487	-0.856871011	3.52737816571e-35	9.78024281052e-35	-
YJL201W	9.992462	5.297959	0.915403502	4.29537101529e-35	1.19043139567e-34	-
YLR107W	77.951408	111.708031	-0.519085922	4.48573147618e-35	1.2426336905e-34	-
YIL021W	53.801895	83.77037	-0.638783058	4.51935916992e-35	1.25139079959e-34	-
YLR370C	330.532959	443.81131	-0.425152297	5.09681616174e-35	1.41065692073e-34	-

YOL111C	298.46936	398.169617	-0.415800302	5.5931902263 9e-35	1.5473495956 1e-34	-
YML035C	33.062027	48.315865	-0.547321813	5.7541923093 4e-35	1.5911815526 2e-34	-
YLR084C	6.735388	4.06525	0.72841698	5.9620068854 1e-35	1.6479135683 3e-34	-
YJR096W	181.842499	247.188217	-0.442920558	7.5778571507 4e-35	2.0936064615 8e-34	-
YML088W	34.104523	50.882801	-0.577215006	8.3339684873 1e-35	2.3014802655 4e-34	-
YOL114C	10.944376	2.863913	1.934130129	8.7156718369 2e-35	2.4058199539 2e-34	up
YDR054C	255.669373	336.017303	-0.394256177	8.9483874438 e-35	2.4689595222 7e-34	-
YDR130C	39.468483	26.60301	0.56911158	9.7074496988 3e-35	2.6772033643 e-34	-
YLR278C	18.234272	26.935167	-0.562838409	1.0005083182 8e-34	2.7580620070 5e-34	-
YOL019W	47.959995	70.131874	-0.548238771	1.0573195253 2e-34	2.9133775469 e-34	-
YLR072W	21.382103	15.423836	0.471242137	1.1577988590 9e-34	3.1888266713 6e-34	-
YOL066C	15.153364	26.407749	-0.801323228	1.2005474995 4e-34	3.3050992803 4e-34	-
YDL108W	47.218365	75.373207	-0.674703691	1.2996505554 2e-34	3.5763433723 6e-34	-
YOR001W	26.237076	40.052971	-0.610302314	1.4804432465 4e-34	4.0720388455 9e-34	-
YMR275C	11.488685	19.16046	-0.737918521	1.5524682176 7e-34	4.2682562866 6e-34	-
YER132C	4.101703	7.482935	-0.867381212	1.5672874618 7e-34	4.3070918828 3e-34	-
YFL054C	24.285942	17.77886	0.449958627	1.5851439894 5e-34	4.3542362329 8e-34	-
YDL088C	46.58453	68.703201	-0.56052638	1.6216187198 8e-34	4.4524586523 8e-34	-
YIL091C	9.074413	16.898563	-0.897024342	1.6310908501 6e-34	4.4764862943 3e-34	-
YAR019C	20.018143	30.477835	-0.606452277	1.9252499939 8e-34	5.2814635274 4e-34	-
YEL018W	34.252617	58.829746	-0.780331589	1.9460205758 3e-34	5.3360846885 1e-34	-
YOR319W	80.943176	125.429237	-0.63189231	2.1322462844 1e-34	5.8441434585 4e-34	-

YKL051W	24.977497	43.583862	-0.803165129	2.2751417755 2e-34	6.2330450760 8e-34	-
YGL250W	72.785919	54.69733	0.412188966	2.3872270404 4e-34	6.5372322307 3e-34	-
YML127W	15.870446	27.443766	-0.790135798	2.4946229431 3e-34	6.8283153575 8e-34	-
YHR031C	15.961333	10.812346	0.561901558	2.6122400668 2e-34	7.1471072432 e-34	-
YDL051W	75.174271	113.385475	-0.592924961	2.9569173682 7e-34	8.0865828291 7e-34	-
YDR512C	132.010101	106.234283	0.313398909	3.3715482103 3e-34	9.2164558739 5e-34	-
YOR069W	31.760872	47.652977	-0.585315823	3.8681597595 8e-34	1.0569337934 6e-33	-
YPR158C-D	12.87228	10.189793	0.337142869	3.9149081357 4e-34	1.0692366786 9e-33	-
YLR165C	33.824303	20.944727	0.691473126	4.4856115208 5e-34	1.2245680000 6e-33	-
YMR180C	39.52597	27.535452	0.521510577	4.6536079812 9e-34	1.2698724548 5e-33	-
YLL038C	63.480381	99.719795	-0.65156913	4.8224397379 4e-34	1.3153649337 9e-33	-
YBL006C	152.189514	220.877289	-0.537376127	5.3529934172 4e-34	1.4594371161 3e-33	-
YML106W	64.740501	102.771332	-0.666697447	5.3568949937 e-34	1.4598597068 e-33	-
YMR140W	207.700729	266.56543	-0.359983414	5.4282630332 9e-34	1.4786598030 1e-33	-
YLR074C	175.846283	253.171585	-0.525800651	5.530589231e-34	1.5058727169 3e-33	-
YLR077W	21.621897	35.205864	-0.703322645	5.8783229124 5e-34	1.5998521981 8e-33	-
YML097C	15.945009	28.96199	-0.861055823	6.2042364175 9e-34	1.6878133076 4e-33	-
YOL137W	12.236769	23.084345	-0.915692119	6.5245651102 6e-34	1.7741787211 8e-33	-
YDL049C	19.135185	37.534088	-0.97197358	9.7190123641 4e-34	2.6416650068 6e-33	-
YGL211W	30.003803	50.185257	-0.742118229	1.0082018118 7e-33	2.7391320998 2e-33	-
YDL217C	37.946571	67.483147	-0.83055773	1.0423625686 5e-33	2.8307028985 8e-33	-
YIL156W-B	369.406586	302.74231	0.287119273	1.0458042819 9e-33	2.8388076006 1e-33	-

YIL040W	17.933224	43.248802	-1.270025294	1.05571990694e-33	2.86447079646e-33	down
YDR331W	32.481106	52.283825	-0.686763932	1.06291077259e-33	2.88272174583e-33	-
YGR258C	24.028862	35.287014	-0.554369029	1.12071349596e-33	3.03816130257e-33	-
YGR013W	41.915726	61.186001	-0.545709993	1.12137202804e-33	3.0386196203e-33	-
YBR213W	17.223665	34.474483	-1.001136752	1.12533585891e-33	3.04803010999e-33	down
YNL283C	15.417871	9.252377	0.736707606	1.2451061438e-33	3.37096334092e-33	-
YBL085W	32.295174	46.003971	-0.510439805	1.38747625616e-33	3.75477445432e-33	-
YOR272W	27.119755	44.146366	-0.702950539	1.42616267078e-33	3.85778556001e-33	-
YBR083W	36.622791	27.762882	0.399584404	1.48178433404e-33	4.00649701468e-33	-
YDR282C	32.851688	52.657272	-0.68066528	1.67033008122e-33	4.51432701096e-33	-
YBL084C	26.516209	40.028702	-0.594160305	1.72638258448e-33	4.66378724301e-33	-
YOR223W	78.828835	116.769135	-0.566863626	1.79718509539e-33	4.85294696483e-33	-
YJL207C	9.419471	14.339672	-0.60629408	1.81942038142e-33	4.91085292516e-33	-
YER018C	50.867577	34.440628	0.562634926	1.83428869671e-33	4.94883278103e-33	-
YGL119W	14.881474	8.813804	0.755680712	1.95813652151e-33	5.28067399023e-33	-
YIL088C	176.898254	229.274109	-0.374153638	2.2909681987e-33	6.17556690296e-33	-
YOL125W	16.631081	29.479311	-0.825820861	2.7354478856e-33	7.37051235842e-33	-
YHR018C	234.133087	298.736267	-0.351543567	2.74973297745e-33	7.40578842691e-33	-
YDR227W	15.725487	23.511381	-0.580254585	3.33786798323e-33	8.98589958365e-33	-
YBR090C	65.356911	39.787674	0.716018237	3.33837817388e-33	8.98337741804e-33	-
YMR311C	613.22406	769.721741	-0.327922695	3.6322608101e-33	9.76996321886e-33	-
YMR106C	15.330324	26.095493	-0.76741247	3.69161406851e-33	9.92530971734e-33	-

YBR205W	34.874306	25.150688	0.471566642	4.2938002976e-33	1.15393559513e-32	-
YMR308C	11.440699	18.575321	-0.699211942	4.30690169623e-33	1.1569556785e-32	-
YNL156C	185.736282	249.303558	-0.424647812	5.41777177598e-33	1.454737335e-32	-
YHR012W	108.219719	154.477051	-0.513429128	5.76997140931e-33	1.54863737609e-32	-
YER125W	43.75251	61.400681	-0.488888873	6.33932376474e-33	1.70071399877e-32	-
YER184C	42.705742	60.203686	-0.495421759	7.46680845656e-33	2.00233031958e-32	-
YBL024W	10.158697	18.437899	-0.859958902	8.41905658998e-33	2.25671430529e-32	-
YGR188C	3.504707	1.458961	1.264352519	9.43563041346e-33	2.52811366451e-32	up
YOR211C	27.228485	39.993553	-0.554650742	1.01942422029e-32	2.73019221724e-32	-
YLR386W	14.611053	10.328071	0.500489332	1.07118505121e-32	2.86757947302e-32	-
YHL026C	12.64232	26.24382	-1.053716493	1.17044691141e-32	3.13195449398e-32	down
YGL032C	30.116812	9.926404	1.601225984	1.18731420033e-32	3.17572018771e-32	up
YNL294C	61.115936	86.484253	-0.500888859	1.20264220007e-32	3.21533280707e-32	-
YAL044W-A	218.195923	176.327179	0.307369277	1.25472207474e-32	3.35312726645e-32	-
YOL044W	95.202919	133.026154	-0.482632205	1.28742791282e-32	3.43905012168e-32	-
YDR182W	58.299953	83.73349	-0.522310037	1.32849235481e-32	3.54721743597e-32	-
YJL049W	17.229401	10.447545	0.721708573	1.39748253191e-32	3.72982440159e-32	-
YDR113C	24.30686	15.555593	0.643930142	1.64473121988e-32	4.38783472842e-32	-
YBL106C	6.512038	3.72202	0.807023311	1.65107043371e-32	4.4028544899e-32	-
YPL115C	15.44708	23.69739	-0.617394024	1.72288759733e-32	4.59239424827e-32	-
YIL149C	4.883559	3.02446	0.691255346	1.74119347854e-32	4.63919704497e-32	-
YPR156C	44.546963	64.188927	-0.526997366	1.89173424643e-32	5.03813221871e-32	-

YLR033W	33.002338	50.990749	-0.627667296	1.90802748852e-32	5.07934590426e-32	-
YBL113C	15.28128	10.660069	0.519548616	2.09879472325e-32	5.58479110241e-32	-
YDR096W	108.302536	138.9702	-0.359708527	2.21302041217e-32	5.88621710316e-32	-
YBL023C	8.918124	5.419554	0.718566129	2.33097545794e-32	6.19730006119e-32	-
YOR241W	21.931721	35.675949	-0.701932781	2.3919641698e-32	6.35672669782e-32	-
YJR033C	16.722569	24.697903	-0.562592055	2.68005961535e-32	7.11930256401e-32	-
YDL030W	23.073086	37.408798	-0.697166635	2.85321572277e-32	7.57603216721e-32	-
YER053C	317.132629	302.838196	0.0665391411913	3.31835466383e-32	8.80733037754e-32	-
YGL194C	17.380047	30.603279	-0.816254256	3.3553092814e-32	8.90160684569e-32	-
YDR147W	12.234477	22.417498	-0.873672839	3.52052380767e-32	9.33592985818e-32	-
YBR268W	106.033562	174.562225	-0.719220413	3.52357721771e-32	9.34003730467e-32	-
YCR002C	32.45179	21.788996	0.574698337	3.8177411249e-32	1.01154660279e-31	-
YBR283C	75.065964	104.626938	-0.479023524	4.11888410119e-32	1.09087169369e-31	-
YLR321C	17.365114	30.952538	-0.833865828	4.22635816661e-32	1.11885848607e-31	-
YOR003W	25.893507	18.205643	0.50820468	4.26049111506e-32	1.12741384665e-31	-
YDR165W	11.667344	22.523844	-0.948976886	4.61918285755e-32	1.22181027608e-31	-
YDR498C	46.386543	35.488392	0.386359125	4.64056436826e-32	1.2269430834e-31	-
YBR071W	443.235809	422.893097	0.0677814303075	4.64751792297e-32	1.22825846172e-31	-
YDR229W	103.82708	141.082108	-0.442352263	6.17879889341e-32	1.63225461831e-31	-
YOL072W	25.724447	41.948826	-0.705490372	6.50180592273e-32	1.71685287828e-31	-
YJL218W	31.231709	17.459047	0.839036704	7.30705533955e-32	1.92866494676e-31	-
YGL172W	60.818901	87.068222	-0.517626516	7.35770237025e-32	1.94120766318e-31	-

YIL029C	43.557365	24.503048	0.82995546	7.54253366346e-32	1.98912697463e-31	-
YBR015C	49.230083	70.252815	-0.513015863	9.6326928828e-32	2.53926783084e-31	-
YAR068W	25.030317	11.445717	1.128868721	1.14422034346e-31	3.01499146528e-31	up
YNL259C	96.858063	57.663822	0.748205689	1.16326019955e-31	3.06386055103e-31	-
YOL089C	46.905445	63.514229	-0.437324426	1.35776624155e-31	3.57464496504e-31	-
YNL037C	220.727631	207.9897	0.0857551540985	1.36015520067e-31	3.579416484e-31	-
YIL147C	6.685595	11.632602	-0.79904597	1.6265071226e-31	4.27854076995e-31	-
YKR061W	10.907196	21.530842	-0.981124475	2.02859583068e-31	5.33397836376e-31	-
YNL233W	8.213138	4.957703	0.728261692	2.16516415186e-31	5.69066005704e-31	-
YOR129C	5.726064	11.040937	-0.947246913	2.24533669218e-31	5.89887862254e-31	-
RDN58-2	45.033604	107.992455	-1.261856676	2.28951741055e-31	6.01240443515e-31	down
YHR162W	558.776489	522.472473	0.0969162900706	2.38687553701e-31	6.26542212845e-31	-
YOL017W	5.77878	2.702347	1.096552009	2.48914678548e-31	6.53111717846e-31	up
YDR044W	46.596104	72.467827	-0.637131302	2.57562792522e-31	6.75517454996e-31	-
YIL067C	6.078011	12.365267	-1.024622201	2.61631586372e-31	6.85899023731e-31	down
YGR208W	10.434973	22.706537	-1.121680828	2.67489097572e-31	7.00959188571e-31	down
YDR475C	28.482603	41.375671	-0.53870171	2.85550334479e-31	7.47973196811e-31	-
YER001W	30.19058	44.310928	-0.553564068	2.99048405952e-31	7.82999790869e-31	-
YPL109C	44.22855	37.118481	0.252840273	3.15948684915e-31	8.26901111278e-31	-
YNL195C	340.837097	438.666992	-0.364043785	3.30057785491e-31	8.63463435452e-31	-
YFL027C	24.819878	39.947433	-0.686606775	3.51108734475e-31	9.18147861676e-31	-
YGL161C	118.234184	102.665482	0.203696007	4.66946557308e-31	1.22054914853e-30	-

YNL097C	78.26368	64.57579	0.27734956	4.96796812438e-31	1.29802803519e-30	-
YJL112W	48.324909	41.642239	0.214719375	5.53406489533e-31	1.44532919101e-30	-
YKL045W	33.814098	26.093266	0.373947338	5.69554534611e-31	1.48687743939e-30	-
YHR142W	27.534508	17.703894	0.637174112	5.93489774409e-31	1.54871144159e-30	-
YGL004C	62.654034	51.822998	0.273814922	6.20467530426e-31	1.61842959197e-30	-
YGL099W	10.804728	19.376547	-0.842648744	6.57938255704e-31	1.71544758144e-30	-
YMR036C	92.510132	83.693993	0.144487303	6.71207670596e-31	1.74931033546e-30	-
YPR188C	66.831421	46.012192	0.538510362	7.12620084477e-31	1.85646054739e-30	-
YGR170W	15.625563	12.061695	0.373475512	8.40413727302e-31	2.18845990734e-30	-
YDR472W	20.463604	38.10487	-0.896915142	8.47439737335e-31	2.20583056158e-30	-
YBR074W	10.71568	17.702967	-0.724267771	8.6552883865e-31	2.25197109402e-30	-
YLL016W	30.039083	42.370575	-0.496221935	9.01014158873e-31	2.34331625399e-30	-
YPR107C	51.846008	35.595097	0.542554377	9.37588077546e-31	2.43741490176e-30	-
YOR312C	201.894989	281.704529	-0.480577655	9.48643601833e-31	2.46512326504e-30	-
YOR377W	39.761299	58.965279	-0.5685008	9.83893298244e-31	2.55565255042e-30	-
YMR211W	28.122145	20.4074	0.462614251	1.01223416129e-30	2.62816799386e-30	-
YPR178W	14.877355	26.637819	-0.840357908	1.06178197435e-30	2.75566157892e-30	-
YNL132W	3.782093	7.706706	-1.026929516	1.08326712103e-30	2.81024750829e-30	down
YER130C	41.399754	62.485233	-0.593893086	1.09357374474e-30	2.83580025369e-30	-
YLR168C	35.141869	22.188393	0.663385739	1.63379124851e-30	4.23489606296e-30	-
YLR087C	6.285674	4.905245	0.357742254	1.70831079679e-30	4.42620760703e-30	-
YOR229W	16.334085	28.599331	-0.808095759	1.78133778603e-30	4.61349394062e-30	-

YLR346C	30.71158	66.943649	-1.124164457	1.81708690682e-30	4.70411823084e-30	down
YPL195W	17.346165	26.637598	-0.618847258	1.90535021085e-30	4.93056027885e-30	-
YDR451C	7.453259	2.643757	1.495281816	2.51966581812e-30	6.51753558287e-30	up
YOR278W	36.671089	24.819712	0.563156735	2.96209502216e-30	7.65876130678e-30	-
YDR225W	882.701233	860.208191	0.0372393440979	3.10839885039e-30	8.03369694556e-30	-
YIL078W	36.43502	30.356379	0.263326071	3.12813404324e-30	8.08133838554e-30	-
YBR188C	69.749336	46.869045	0.573544084	3.2191489179e-30	8.31301018399e-30	-
YDL084W	130.129028	172.624573	-0.407695025	3.22244470848e-30	8.31806101882e-30	-
YER005W	18.346846	29.68189	-0.694050888	3.35549736075e-30	8.65790840214e-30	-
YMR035W	138.52829	200.690842	-0.534794154	3.43420700719e-30	8.85731495665e-30	-
YHL030W	4.23282	2.659009	0.670730479	4.10184767429e-30	1.05748631071e-29	-
YDR088C	33.252041	23.961061	0.472751103	4.11840967131e-30	1.06131536901e-29	-
YBR255W	27.362215	40.978302	-0.582675183	4.6491072199e-30	1.1975791544e-29	-
YOR086C	37.670593	33.671955	0.161891257	4.87119522743e-30	1.25426710792e-29	-
YPL029W	8.252212	15.115843	-0.87320865	6.08894884609e-30	1.56717224032e-29	-
YDR068W	51.35421	39.001099	0.396967775	6.22120758409e-30	1.60054938591e-29	-
YDR338C	29.617666	43.831367	-0.565505719	7.7224738862e-30	1.98596180139e-29	-
YJR053W	5.275499	2.085352	1.339016636	7.85765006605e-30	2.01988785135e-29	up
YOL128C	8.377923	18.061083	-1.108219874	1.01768887289e-29	2.61498862703e-29	down
YIL061C	27.710434	47.438118	-0.775617469	1.03819441143e-29	2.66657464052e-29	-
YOL004W	25.338633	34.810219	-0.458172196	1.16095015995e-29	2.98063630808e-29	-
YLR206W	78.108818	105.186768	-0.429395898	1.24391345009e-29	3.19231694838e-29	-

YNL082W	5.860688	3.129433	0.905168747	1.25708095747e-29	3.22477627437e-29	-
YMR002W	605.795837	773.491821	-0.352554372	1.313131159e-29	3.36716986166e-29	-
YGR154C	23.463902	15.12608	0.633404796	1.33291862555e-29	3.41649827721e-29	-
YOL025W	46.071423	64.935783	-0.495141539	1.47534808775e-29	3.78000863754e-29	-
YHR147C	146.117508	205.204071	-0.489930298	1.57538074857e-29	4.03463848478e-29	-
YMR184W	44.705296	29.363998	0.606397341	1.63783171616e-29	4.19284919336e-29	-
YBL026W	174.065521	133.726608	0.38034391	1.76200001135e-29	4.50886070506e-29	-
YEL017W	55.142658	43.368092	0.346534838	1.98989921503e-29	5.08994409845e-29	-
YKL166C	6.691496	15.096506	-1.173813993	2.09164634265e-29	5.3479985565e-29	down
YMR222C	56.401821	40.798676	0.467219408	2.15660685055e-29	5.51182187247e-29	-
YPL124W	12.198346	4.959451	1.298433211	2.31899498261e-29	5.92441187327e-29	up
YLR154W-C	35.413734	69.505104	-0.972809955	2.38612392844e-29	6.09340080123e-29	-
YJL145W	29.087751	18.942976	0.618748754	2.46403922081e-29	6.28978432681e-29	-
YOR038C	9.015802	5.720408	0.656337783	2.49745756799e-29	6.37246879659e-29	-
YDL110C	394.410522	364.412262	0.114126535	2.57069623329e-29	6.55664840437e-29	-
YNL068C	16.152313	11.969411	0.432388612	2.75923116677e-29	7.03462303217e-29	-
YHR165C	14.482625	20.084679	-0.471772287	3.25758828465e-29	8.3017685021e-29	-
YKL050C	12.895751	9.214582	0.484905165	3.36798660181e-29	8.57958999755e-29	-
YAL036C	12.891421	24.81905	-0.945036596	3.44895570421e-29	8.78224651835e-29	-
YDL226C	25.237862	42.528118	-0.752827314	3.64665737489e-29	9.27995449204e-29	-
YLR275W	80.655258	135.860611	-0.752286755	3.64665737489e-29	9.27995449204e-29	-
YBL029W	35.351601	25.963125	0.445311512	3.89493378995e-29	9.90567348137e-29	-

YLR356W	438.042969	418.624664	0.0654150822361	3.89807334578e-29	9.90959841548e-29	-
YPL164C	30.35701	44.436916	-0.549728994	4.7898963672e-29	1.2171787412e-28	-
YJR134C	23.755133	18.365707	0.371224849	5.5025681834e-29	1.39770635362e-28	-
YCR031C	364.497437	331.258636	0.137950604	5.52468503065e-29	1.40275029326e-28	-
YPR148C	242.716888	235.595383	0.0429632265471	5.87120500682e-29	1.49012431244e-28	-
YOR179C	99.909935	79.030914	0.338211053	7.0973939377e-29	1.80059753025e-28	-
YPL257W	48.334988	32.461002	0.57436035	7.55645119627e-29	1.91627651252e-28	-
YLL003W	24.919979	20.52747	0.279747026	9.95964430919e-29	2.52468239573e-28	-
YGL136C	59.514584	88.487022	-0.572222634	1.0210031794e-28	2.58709703581e-28	-
YPL206C	101.993347	142.083939	-0.478268435	1.06974170252e-28	2.70948857171e-28	-
YJR035W	16.407295	24.628122	-0.585969213	1.09647578967e-28	2.77606920975e-28	-
YNR032W	35.94968	56.341942	-0.648230363	1.24418487183e-28	3.14875649585e-28	-
YLR405W	21.047909	36.284393	-0.785672219	1.24493214311e-28	3.14936379154e-28	-
YLR254C	196.607315	270.252838	-0.458992768	1.24589228493e-28	3.1505088818e-28	-
YNL287W	48.231514	65.092941	-0.432525002	1.24760840634e-28	3.15356391962e-28	-
YGL049C	23.042868	33.793118	-0.552409179	1.35522280096e-28	3.42418524558e-28	-
YJL104W	105.923508	161.529236	-0.608772502	1.41712639776e-28	3.57913778572e-28	-
YIL046W-A	17.785374	56.985096	-1.679893331	1.48517413882e-28	3.74947582504e-28	down
YMR040W	94.545784	145.022385	-0.617190573	1.53967394978e-28	3.88548613018e-28	-
YBR017C	15.75689	24.350889	-0.627991631	1.59311250006e-28	4.0187088177e-28	-
YNL206C	31.436817	48.658375	-0.63023299	1.62040059234e-28	4.08588419061e-28	-
YOL087C	49.544437	65.655487	-0.40619251	1.66618749108e-28	4.1996313214e-28	-

YIR010W	32.122158	25.401705	0.338643482	1.85024013559e-28	4.66164397798e-28	-
YLR021W	77.289795	57.627556	0.423519104	1.93290126308e-28	4.86793145687e-28	-
YGR058W	29.349691	48.277473	-0.718004848	1.96785086082e-28	4.9539408532e-28	-
YLR133W	26.51911	20.250879	0.389047828	2.15742228464e-28	5.42897346698e-28	-
YDL133W	72.459267	101.174065	-0.4815974	2.29272216668e-28	5.7671066494e-28	-
YHR080C	26.919369	37.070816	-0.461639279	2.408521567e-28	6.055934341e-28	-
YML119W	106.910942	94.523407	0.17766598	2.81397348451e-28	7.07252930843e-28	-
YBR159W	44.806858	34.30677	0.385226262	2.84027246915e-28	7.13573916975e-28	-
YOR259C	279.786499	274.789398	0.0260000046623	3.19731547127e-28	8.02950422559e-28	-
YDR329C	272.454895	341.691345	-0.326676294	3.36117477176e-28	8.43759522164e-28	-
YNL023C	9.050788	15.199143	-0.74787467	3.37636864753e-28	8.47231065637e-28	-
YPL087W	224.416077	211.6577	0.0844430591168	3.41992962933e-28	8.57815076318e-28	-
YHL028W	13.181492	8.504816	0.632161748	3.44478559712e-28	8.63700686062e-28	-
YHR083W	39.597694	62.028835	-0.647522613	3.75004788604e-28	9.3985859009e-28	-
YLR456W	20.844618	10.489092	0.990785139	3.80545541288e-28	9.53360258401e-28	-
YJR156C	16.281446	9.224925	0.819619748	3.98958600115e-28	9.99086320901e-28	-
YIL113W	46.543118	31.867693	0.546473281	4.14063394344e-28	1.03649417423e-27	-
YOR298W	1.32211	5.096562	-1.946682158	4.1719986208e-28	1.0439245239e-27	down
YDR416W	7.231267	12.98033	-0.844006711	4.92121344819e-28	1.23089819043e-27	-
YMR003W	17.0184	7.572729	1.168210201	5.62966970065e-28	1.40753078943e-27	up
YOR373W	18.71468	14.423244	0.375774696	5.79437432486e-28	1.44812704544e-27	-
YOR168W	29.69659	42.703392	-0.524053391	7.03160673306e-28	1.75662835408e-27	-

YBL107C	67.023125	104.656662	-0.64293329	7.75927210146e-28	1.93763319412e-27	-
YNR058W	3.042396	8.211367	-1.43241447	8.7182948011e-28	2.17624343085e-27	down
YBL046W	23.471977	16.41448	0.515970315	1.02368326014e-27	2.55427077128e-27	-
YDL248W	99.305649	88.026192	0.173942929	1.32221965137e-27	3.29784636229e-27	-
YKL176C	21.63439	32.255337	-0.576211447	1.34098561803e-27	3.34330872158e-27	-
YNL175C	9.215302	18.593306	-1.012679962	1.47144958789e-27	3.66710519536e-27	down
YJL090C	13.308415	21.63302	-0.700896322	1.60039930363e-27	3.98686953328e-27	-
YDR131C	14.59806	9.552553	0.611818394	1.70888331666e-27	4.25541421171e-27	-
YPR106W	48.486942	70.549606	-0.541041758	1.7273481232e-27	4.2996700677e-27	-
YDL190C	24.806139	35.641163	-0.522847212	1.74137847565e-27	4.33285674423e-27	-
YBR215W	25.095474	38.097176	-0.602256864	1.77703945508e-27	4.41981608057e-27	-
YKL074C	20.703043	33.243732	-0.683239515	2.0023055073e-27	4.97809875423e-27	-
YDL167C	7.793901	14.238882	-0.869418363	2.06963748699e-27	5.14343855853e-27	-
YGR215W	74.753685	126.072395	-0.754035811	2.09492001351e-27	5.20418705237e-27	-
YBR243C	13.773815	8.233594	0.742333989	2.15293166696e-27	5.34615991538e-27	-
YDL134C	155.927307	205.231628	-0.396379476	2.2779287485e-27	5.65429095188e-27	-
YFR031C	6.153017	3.883148	0.664067284	2.49859597479e-27	6.19955388149e-27	-
YHL044W	19.138256	10.040145	0.930679263	2.59579306109e-27	6.43814755224e-27	-
YMR146C	168.935287	221.950317	-0.393766061	2.73690409406e-27	6.78542356866e-27	-
YJR144W	65.984695	98.306839	-0.575160352	2.98198307184e-27	7.3900802036e-27	-
YIL065C	404.870148	378.946564	0.0954648482634	3.10513496567e-27	7.69220984314e-27	-
YNL188W	7.009667	2.991557	1.228449358	3.73720481691e-27	9.25431491957e-27	up

YIL052C	198.898788	284.889709	-0.518369023	3.9762666696e-27	9.84236981057e-27	-
YOR328W	18.8388	26.378	-0.485628112	4.03398975484e-27	9.98127078441e-27	-
YIL169C	3.852193	1.879297	1.035486901	4.13134354085e-27	1.02180799608e-26	up
YPL228W	20.380869	32.507122	-0.673540267	4.8440142937e-27	1.1975962061e-26	-
YML126C	169.411209	216.755402	-0.355538616	5.88381842422e-27	1.45409015834e-26	-
YOL142W	39.657318	64.928802	-0.711271478	6.50427324649e-27	1.60678584617e-26	-
YER148W	189.465744	253.945267	-0.422580557	6.76325967287e-27	1.67010008151e-26	-
YMR165C	36.721657	50.966354	-0.472913991	6.94108461898e-27	1.71333015168e-26	-
YCL057C-A	403.572083	360.477966	0.162915302	7.68737886459e-27	1.89679046071e-26	-
YLR312W-A	43.264206	69.139397	-0.676334095	7.95967124798e-27	1.96319583264e-26	-
YDL223C	113.87291	112.042549	0.0233778647191	8.25851522152e-27	2.03609461855e-26	-
YOR349W	5.063801	9.38221	-0.889707083	8.38259818026e-27	2.06586619703e-26	-
YDL109C	8.410379	15.376467	-0.870481338	9.10112173819e-27	2.24205411709e-26	-
YML046W	8.143393	15.075971	-0.888548991	9.38855959277e-27	2.31194676525e-26	-
YGR095C	36.710373	61.672001	-0.748427883	1.0426356654e-26	2.56648779176e-26	-
YML132W	47.758541	38.094677	0.326169341	1.04339907962e-26	2.56734898385e-26	-
YMR193W	88.377892	127.145157	-0.524719085	1.07935455491e-26	2.6547674631e-26	-
YBR201C-A	814.346863	754.138184	0.110814528	1.08384972467e-26	2.66476795674e-26	-
snR11	284.665558	238.608093	0.254624974	1.1103337671e-26	2.72880127717e-26	-
YPL267W	12.575515	4.918027	1.354465923	1.14892010388e-26	2.8225152374e-26	up
YHR156C	30.940683	21.97434	0.493685209	1.195117714e-26	2.93484603184e-26	-
YGL210W	74.362144	111.527336	-0.584757095	1.19753373257e-26	2.93961621661e-26	-

YLR435W	12.546484	27.713192	-1.143289767	1.20808279555e-26	2.96433912838e-26	down
YGR144W	3.664438	10.476868	-1.515543636	1.22719037458e-26	3.01003470779e-26	down
YBR217W	46.272831	76.968109	-0.734095436	1.25604566444e-26	3.07959379339e-26	-
YDR098C	108.226639	152.564148	-0.495360325	1.37385436441e-26	3.36710931474e-26	-
YDR251W	37.113598	51.591801	-0.475193939	1.50364375834e-26	3.6837491917e-26	-
YKR024C	8.509124	15.032969	-0.821047447	1.52844068232e-26	3.74302159995e-26	-
YNR009W	14.621068	7.023505	1.057785621	1.69734157574e-26	4.15500650716e-26	up
YOR214C	0.92921	6.103692	-2.715605578	1.86466766617e-26	4.56281311453e-26	down
YNL185C	57.023247	93.96505	-0.72057406	1.91119452009e-26	4.67482095378e-26	-
YGR194C	75.829216	68.961838	0.136955581	2.06687129805e-26	5.05361836089e-26	-
YDR500C	588.387451	778.564575	-0.404050226	2.10110524363e-26	5.13529974505e-26	-
YJL124C	80.553001	123.653618	-0.61829421	2.12724585427e-26	5.19714374787e-26	-
YGL060W	11.294106	7.230086	0.643485364	2.15274488045e-26	5.2573722336e-26	-
YHR052W	21.24074	35.758892	-0.751468006	2.40028125222e-26	5.85959339904e-26	-
YHR060W	30.050335	17.410181	0.787449873	2.42661194785e-26	5.92154362116e-26	-
YPL022W	25.678684	35.996452	-0.487283446	2.43475657225e-26	5.93908400807e-26	-
YLR075W	940.228149	1142.988525	-0.281728141	2.64333415246e-26	6.44533323586e-26	-
YPR052C	314.420044	271.441681	0.212050921	2.8294657783e-26	6.89647567792e-26	-
YKR008W	75.871643	101.112465	-0.414328177	2.90461625316e-26	7.07686722905e-26	-
YKR026C	20.688303	36.463024	-0.817618904	2.99487546077e-26	7.29391402922e-26	-
YNL187W	14.346451	26.460119	-0.88312566	3.08609446474e-26	7.51312723024e-26	-
YMR033W	24.289177	38.454681	-0.662845617	3.10623099717e-26	7.5591854294e-26	-

YBR063C	21.357241	35.430885	-0.730282215	3.26881649919e-26	7.95172916416e-26	-
YEL040W	8.441064	4.242621	0.992469057	3.34987324099e-26	8.14571605173e-26	-
YBR165W	70.664322	103.340073	-0.548345912	3.47886487409e-26	8.45606622488e-26	-
YLR066W	97.37674	144.06366	-0.565057354	3.56814950151e-26	8.66969554026e-26	-
YJL006C	28.805317	47.117966	-0.709942127	3.65963914042e-26	8.88851321742e-26	-
YKL161C	19.313951	12.950233	0.576665267	3.67230213108e-26	8.91578084854e-26	-
YMR152W	167.674728	157.164169	0.0933929188053	3.67651397066e-26	8.92251709532e-26	-
YDL097C	412.147217	415.676361	-0.012300953	3.7266992673e-26	9.04077727684e-26	-
YCR082W	237.421753	206.149094	0.203764003	3.8873974187e-26	9.42693874035e-26	-
YGR132C	389.946167	488.676971	-0.325606149	4.70199437506e-26	1.13978840611e-25	-
YGL127C	144.083435	212.310425	-0.559270732	5.07933707395e-26	1.23077769536e-25	-
YJR066W	14.427314	19.718187	-0.450724177	5.08410914298e-26	1.23145335777e-25	-
YOR301W	15.72557	9.915706	0.66532491	5.22972239069e-26	1.26622919662e-25	-
YFL046W	73.295074	56.844963	0.366683723	5.52551338953e-26	1.337325034e-25	-
YDR435C	105.776009	144.922134	-0.454265507	5.53302349595e-26	1.33862080526e-25	-
YHL020C	165.911987	214.912109	-0.373328649	5.57473335147e-26	1.34818639057e-25	-
YJR001W	23.904375	18.361681	0.380576543	6.82461743681e-26	1.64981405949e-25	-
YLL048C	18.209978	15.816366	0.203311018	7.11308158142e-26	1.71887934829e-25	-
YGR057C	113.673264	159.448212	-0.488194948	7.17591942906e-26	1.73338940917e-25	-
YKL025C	28.070429	41.141315	-0.551536795	7.32245532107e-26	1.76809811876e-25	-
YEL050C	47.374245	69.606674	-0.555122689	7.53087655837e-26	1.81771701689e-25	-
YDR194C	33.635239	48.318935	-0.522615147	7.61722150671e-26	1.83784341678e-25	-

YJL132W	11.621767	7.91379	0.554388748	7.78029447549e-26	1.87645952229e-25	-
YBL082C	11.426828	6.566209	0.799292402	8.9743752703e-26	2.16360860885e-25	-
YER035W	789.397949	987.155884	-0.322525151	9.19023523106e-26	2.21478960848e-25	-
YML066C	14.628207	26.517851	-0.858210917	9.38674226736e-26	2.26126876196e-25	-
YOL098C	12.084084	18.766836	-0.635077321	1.03530342334e-25	2.49308132353e-25	-
YKL080W	38.091099	57.633823	-0.597461808	1.16306154773e-25	2.7996456333e-25	-
YNR074C	21.870127	36.400387	-0.734992191	1.16643329708e-25	2.80667360786e-25	-
YPR128C	59.439869	48.430164	0.295525048	1.28106741529e-25	3.0813120938e-25	-
YHR216W	7.499917	3.806543	0.978393254	1.40124122535e-25	3.36905713672e-25	-
YLR122C	32.821098	17.087902	0.941648225	1.4514372626e-25	3.4883943191e-25	-
YOL093W	28.999424	19.692959	0.558344343	1.4874987477e-25	3.57368120191e-25	-
YKL077W	78.879517	109.132118	-0.468353132	1.50716636091e-25	3.61953143851e-25	-
YHR190W	214.14505	208.280518	0.0400604295963	1.5278847025e-25	3.66786861297e-25	-
YMR015C	81.028908	108.50071	-0.42119588	1.54463525008e-25	3.70664693951e-25	-
YDR189W	46.094494	63.826214	-0.469554643	1.54894880307e-25	3.71556188929e-25	-
YPL036W	72.001961	68.268005	0.0768266068918	1.60037920532e-25	3.83744847688e-25	-
YDL102W	6.714444	4.370989	0.619308193	1.638489689e-25	3.92731428159e-25	-
YFR009W	37.538303	52.422253	-0.481815928	1.75740585668e-25	4.21072001476e-25	-
YMR052W	45.686108	74.310539	-0.701811289	2.03536139299e-25	4.87481617581e-25	-
YEL029C	13.977525	26.499311	-0.922845924	2.10735251617e-25	5.04529287326e-25	-
YBL060W	13.322374	9.237439	0.528286351	2.28335033472e-25	5.46454852657e-25	-
YKR070W	38.064396	28.932234	0.395764461	2.37493717906e-25	5.68154528231e-25	-

YNL297C	15.942171	22.439894	-0.493217753	2.60421790847e-25	6.22765207078e-25	-
YLR426W	7.956122	17.259659	-1.117266658	2.65915258551e-25	6.35657268035e-25	down
YGL085W	15.326381	29.292538	-0.934516123	2.73646373281e-25	6.53886329995e-25	-
YLL036C	27.125467	41.474072	-0.612561725	2.92054668903e-25	6.9760499598e-25	-
YBR027C	19.472534	6.820338	1.513525494	3.02795945166e-25	7.22983549073e-25	up
YOR280C	32.828983	22.550045	0.541839745	3.10770664244e-25	7.41739440073e-25	-
YGR296W	10.237993	8.328121	0.29786999	3.32094428455e-25	7.92329827767e-25	-
YCL011C	53.179489	75.996971	-0.515072004	3.37646484121e-25	8.05266758903e-25	-
YML070W	110.07869	103.993073	0.0820477729973	3.54486761564e-25	8.45105151994e-25	-
YER041W	8.284117	14.483596	-0.806000006	3.67136123795e-25	8.74925549528e-25	-
YAR042W	49.85622	65.017265	-0.383049365	3.73783298692e-25	8.90424680844e-25	-
YJL196C	25.608158	17.116007	0.581257309	3.84205639753e-25	9.14901653849e-25	-
YML130C	38.728081	32.227978	0.265066341	4.13157155191e-25	9.83466111743e-25	-
YPL155C	10.849788	7.179119	0.595788137	4.24685024963e-25	1.01051921616e-24	-
YGR205W	58.249084	46.444111	0.326739685	4.29502138474e-25	1.02158976078e-24	-
YJL183W	11.777781	21.632359	-0.877123246	4.40896741765e-25	1.04829068283e-24	-
YJL189W	324.477051	257.963928	0.330947103	4.48546750465e-25	1.06607129666e-24	-
RPR1	15.138864	36.293663	-1.261460719	4.60882401151e-25	1.09497051142e-24	down
YAL029C	7.408524	5.39268	0.458183716	4.61397918296e-25	1.09577592838e-24	-
YKL178C	17.566378	29.113262	-0.728859742	4.7635873574e-25	1.13087381701e-24	-
YPR089W	18.00963	26.885031	-0.578034593	4.96106976864e-25	1.17730585335e-24	-
YLR237W	20.552153	15.378606	0.4183648	5.26368627003e-25	1.24864212321e-24	-

YMR132C	13.188126	5.55209	1.248136716	5.5258999945 4e-25	1.3103432836 6e-24	up
YGL100W	61.057713	87.781532	-0.523743896	6.7445331321 6e-25	1.5987041498 4e-24	-
YDR337W	60.308163	89.008873	-0.56159587	7.6020174133 9e-25	1.8012719123 e-24	-
YPR180W	51.704891	75.899574	-0.55379103	8.6543471295 9e-25	2.0498354437 4e-24	-
YOL034W	6.648211	4.343791	0.614011481	8.814232459e-25	2.0869090429 2e-24	-
YBR218C	37.218842	49.669662	-0.41633176	8.8808007072 2e-25	2.1018685013 5e-24	-
YBR203W	28.717661	24.657728	0.219898375	8.9986739484 7e-25	2.1289545683 e-24	-
YNL149C	265.639404	235.844757	0.171631638	9.0290061413 e-25	2.1353169571 5e-24	-
YML112W	33.818134	54.087708	-0.677503706	9.0417555716 1e-25	2.1375178441 9e-24	-
YKL037W	21.019369	8.330111	1.335311735	9.6581974609 2e-25	2.2823787528 5e-24	up
YMR024W	25.764872	41.166355	-0.676060288	1.0095195002 9e-24	2.3847401285 3e-24	-
YLR263W	5.835152	3.310762	0.817606933	1.0129875004 1e-24	2.3920222147 5e-24	-
YFR043C	51.778164	38.859985	0.414058466	1.0268458709 8e-24	2.4238247783 5e-24	-
YJL109C	5.55317	8.907597	-0.681724727	1.1452413910 7e-24	2.7022647494 3e-24	-
YOR043W	143.370895	184.472656	-0.363654805	1.1526486991 9e-24	2.7187093938 3e-24	-
YOR148C	55.595203	40.076157	0.472216235	1.2125337325 e-24	2.8588717855 6e-24	-
YFL011W	2.285622	6.220471	-1.444436994	1.2578735203 8e-24	2.9646464747 6e-24	down
YNL190W	888.068054	899.237305	-0.018031651	1.2764293909 9e-24	3.0072385803 6e-24	-
YPR172W	151.151123	132.225189	0.19299466	1.3768126537 5e-24	3.2425087080 7e-24	-
YCR015C	12.021041	6.13616	0.97015383	1.4496414976 4e-24	3.4127320505 6e-24	-
YPL003W	19.106697	13.257391	0.527281512	1.5254735026 1e-24	3.5898936710 4e-24	-
YDR341C	31.614239	45.957722	-0.539732797	1.6316227205 9e-24	3.8382394276 e-24	-

YDR002W	193.314865	174.348053	0.148982325	1.71610532237e-24	4.03544766715e-24	-
YGL195W	5.421721	4.216906	0.362566012	1.81536618114e-24	4.26724469994e-24	-
YJR044C	257.166046	230.066055	0.160652037	2.02115827808e-24	4.74918644599e-24	-
YOR344C	135.318558	182.043945	-0.427927047	2.02676336438e-24	4.76055503825e-24	-
YLR354C	97.536537	86.262474	0.177209658	2.21173274933e-24	5.19305480628e-24	-
YLR351C	112.769035	99.782654	0.176510028	2.4066750358e-24	5.64863463979e-24	-
YEL023C	6.393156	3.49474	0.871343171	2.46047084196e-24	5.77271465869e-24	-
YIL092W	9.590795	16.756475	-0.804996373	2.5892858843e-24	6.07264328286e-24	-
YCL039W	22.975065	33.838474	-0.558595566	2.72136798298e-24	6.38000469726e-24	-
YLR177W	100.442734	95.044556	0.079697305	2.81100703692e-24	6.58766768033e-24	-
YMR163C	7.399032	4.36264	0.762135109	2.99617077862e-24	7.01895403536e-24	-
YBR136W	3.532966	2.424324	0.543297344	3.2690423354e-24	7.65530547649e-24	-
YOR249C	9.617073	6.145287	0.64611748	3.37072755428e-24	7.89045122811e-24	-
YFL002C	15.075821	24.38854	-0.693966824	3.44009656288e-24	8.0498000235e-24	-
YOR358W	41.010792	65.407372	-0.673449644	3.59558213023e-24	8.4104649075e-24	-
YHR146W	447.834839	542.294067	-0.276108621	3.78662265672e-24	8.85399376759e-24	-
YPL088W	34.92944	54.077179	-0.630576381	4.18055818899e-24	9.77142516463e-24	-
YDL123W	137.003098	198.585892	-0.535554617	4.42937836742e-24	1.03491083571e-23	-
YHR020W	11.926037	19.623402	-0.718460457	4.68368155588e-24	1.09391629416e-23	-
YMR314W	371.384094	360.599579	0.0425143150221	4.77938307064e-24	1.11584844312e-23	-
YLR306W	23.107981	12.708882	0.862554091	5.04033176591e-24	1.17633006025e-23	-
YMR216C	90.88398	116.974991	-0.364102198	5.27969032406e-24	1.23172933227e-23	-

YDR405W	185.704437	244.850464	-0.398892644	5.41028413277e-24	1.26172215989e-23	-
YDR443C	7.343916	5.344697	0.458441396	5.53173237553e-24	1.28956044263e-23	-
YDL174C	240.583527	240.814362	-0.001383573	5.64987688093e-24	1.31660794583e-23	-
YGR261C	11.668244	18.7402	-0.683548889	5.66067397243e-24	1.31862904393e-23	-
YFR036W	37.755062	69.416702	-0.878612738	6.38353251407e-24	1.48645798377e-23	-
YBL090W	142.126328	199.444168	-0.488811108	6.47758580132e-24	1.50779350036e-23	-
YPL106C	742.447693	782.4646	-0.075736095	6.57766359212e-24	1.53051482683e-23	-
YIR007W	13.232021	9.569784	0.467475162	6.64934527451e-24	1.5466142924e-23	-
YKL192C	651.809814	630.412659	0.0481545691147	7.10337178048e-24	1.65160044993e-23	-
YCR027C	13.858091	28.643726	-1.047490637	7.56063348648e-24	1.75725992827e-23	down
YOL018C	51.195873	42.514469	0.268073597	7.57576738372e-24	1.76011841011e-23	-
YDR180W	2.445777	1.247633	0.971099251	7.82582640847e-24	1.81753574051e-23	-
YHR047C	16.424858	12.780611	0.361924089	7.8464652708e-24	1.82164758419e-23	-
YFR046C	20.19281	13.396437	0.591992344	7.98782900849e-24	1.85377355083e-23	-
YLR143W	5.584095	10.755214	-0.94564084	8.1708083178e-24	1.89552982201e-23	-
YLR016C	105.267426	150.34549	-0.514222514	8.32526116304e-24	1.93063957042e-23	-
YLR451W	11.085115	17.657541	-0.671660709	8.65987785698e-24	2.0074877422e-23	-
YOR314W	7.399197	0.611445	3.597074072	9.60669991196e-24	2.22614382432e-23	up
YLR020C	10.618623	18.690386	-0.815699672	1.00034737073e-23	2.31722256623e-23	-
YDR460W	44.797028	67.315247	-0.587530293	1.03176299924e-23	2.3891028345e-23	-
YGL066W	27.291155	39.813229	-0.544814431	1.11061558216e-23	2.57073136989e-23	-
YPL181W	85.547447	78.353821	0.12672117	1.25374667323e-23	2.90095391257e-23	-

YJL095W	4.556009	2.959971	0.622187556	1.27874678617e-23	2.95769748455e-23	-
YLR034C	31.853609	47.529568	-0.57736845	1.29574321556e-23	2.99589343844e-23	-
YLR009W	57.049633	88.64299	-0.63578894	1.33622469679e-23	3.08834062461e-23	-
YIL041W	301.203583	377.942993	-0.32742971	1.54010478308e-23	3.55823241286e-23	-
YBR264C	48.415161	77.202278	-0.673184526	1.54091424985e-23	3.55877814847e-23	-
YOL002C	19.006157	33.029648	-0.797294743	1.59949272492e-23	3.69269276173e-23	-
YGR063C	40.033363	75.544914	-0.916131816	1.75950209283e-23	4.06059070346e-23	-
YPL012W	6.967674	11.34168	-0.702885327	1.78539490946e-23	4.11881516088e-23	-
YKR038C	38.479298	30.347437	0.342507801	1.82411101449e-23	4.20656804531e-23	-
YHR136C	57.843426	40.394886	0.517980345	1.89751882334e-23	4.37422831611e-23	-
YLR460C	24.579922	17.485119	0.491352723	1.96438501672e-23	4.52668974899e-23	-
YLR208W	127.034698	170.867844	-0.427658313	1.99322336755e-23	4.59143995018e-23	-
YOL119C	46.92585	66.138313	-0.495103369	3.04503023731e-23	7.01170167404e-23	-
YIR015W	57.359924	93.635162	-0.707007282	3.44399885496e-23	7.92745453896e-23	-
YBR078W	294.525177	293.561829	0.00472657484764	3.56412644117e-23	8.20092548066e-23	-
YPR171W	17.491871	27.623104	-0.65919083	3.57080844103e-23	8.21325631786e-23	-
YBR234C	122.61705	113.518829	0.111227988	3.65213078297e-23	8.39719551876e-23	-
YDR100W	186.988602	162.386383	0.203519673	3.82380932144e-23	8.78867392355e-23	-
YNL265C	72.044991	60.623093	0.249030669	3.84201260442e-23	8.82724435538e-23	-
YML071C	10.692576	6.909891	0.629874603	3.84439881448e-23	8.8294590604e-23	-
YGL248W	164.874817	213.150391	-0.370500644	3.86498123387e-23	8.87344803989e-23	-
YBR052C	457.465454	448.518311	0.0284959156854	3.97658962926e-23	9.12630995137e-23	-

YFL042C	63.310894	84.151917	-0.410542369	4.05313765493e-23	9.29855083584e-23	-
YPL152W	42.085289	33.387325	0.334015515	4.08309426421e-23	9.3638157341e-23	-
YKL095W	23.97341	40.672386	-0.762614494	4.16840500891e-23	9.55592994657e-23	-
YOR352W	76.272728	106.17247	-0.477170525	4.46678544658e-23	1.02361772065e-22	-
YOR265W	69.015221	46.393646	0.572987347	4.65618594411e-23	1.06662739266e-22	-
YGL031C	204.628845	279.22464	-0.448416732	4.78398077393e-23	1.09549806878e-22	-
YOR112W	12.214894	8.74428	0.482229841	4.82803222086e-23	1.10517787711e-22	-
YOR074C	16.859875	10.051238	0.746220633	4.95720417677e-23	1.13432818022e-22	-
YML038C	33.398884	49.779827	-0.57576132	5.13636405414e-23	1.17489123243e-22	-
YKL019W	69.884834	99.043358	-0.503080825	5.33258478251e-23	1.21932546334e-22	-
YBR097W	4.990967	3.344126	0.577690159	5.50580682583e-23	1.25847013162e-22	-
YGR174C	440.604858	561.152466	-0.348907405	5.75515565568e-23	1.31497998934e-22	-
YLR420W	69.31765	96.797417	-0.481745805	6.16164515447e-23	1.40733970268e-22	-
YBR173C	446.821991	426.779053	0.0662108238072	6.66221589351e-23	1.5211120363e-22	-
YIL085C	28.355114	42.280827	-0.576394639	7.0345239982e-23	1.60552665371e-22	-
YMR149W	49.862907	74.610725	-0.581416033	7.55321485233e-23	1.72327665576e-22	-
YMR206W	117.943733	158.578537	-0.427098759	7.59001415467e-23	1.73103629214e-22	-
YPL209C	5.652698	2.090994	1.4347507	7.69761359734e-23	1.75493151716e-22	up
YNL325C	8.256014	13.756631	-0.736609875	7.69911450486e-23	1.75462932622e-22	-
YML104C	4.816617	2.94669	0.708924918	7.88243798306e-23	1.79574954124e-22	-
YDR159W	7.322474	11.633553	-0.667888707	8.10400712444e-23	1.84554938476e-22	-
YCR089W	2.269438	1.181633	0.941553052	8.15723101359e-23	1.85698900375e-22	-

YBL088C	6.367668	5.213467	0.288522022	8.94567992559e-23	2.03573244055e-22	-
YIL116W	77.028175	105.703453	-0.456564356	9.30218546466e-23	2.11608528269e-22	-
YDR371W	5.989995	2.857304	1.06790026	9.88022687211e-23	2.24675635246e-22	up
YPR185W	18.348845	14.312642	0.358399246	1.03036147768e-22	2.34217651169e-22	-
YCL054W	5.515862	10.041379	-0.864299143	1.11252432358e-22	2.52802013205e-22	-
YKL160W	325.80069	302.513062	0.106992222	1.23733311287e-22	2.81059786487e-22	-
YBR163W	9.444575	16.55401	-0.809622951	1.28199859297e-22	2.91099022134e-22	-
YJR091C	110.712631	137.040848	-0.307786158	1.3213033202e-22	2.99914113777e-22	-
YIL002C	9.624983	6.883019	0.4837425	1.33706461071e-22	3.0338074208e-22	-
YOR173W	612.090942	627.342041	-0.035506229	1.37720916902e-22	3.12375393543e-22	-
YIL044C	32.249985	51.082424	-0.663528497	1.38353721305e-22	3.13696092718e-22	-
YGR257C	24.797041	39.653675	-0.677286601	1.44496369479e-22	3.27504002089e-22	-
YNL223W	59.205772	81.278633	-0.457138306	1.47368591617e-22	3.33892049912e-22	-
YLR425W	13.610904	19.746969	-0.536868338	1.59092357138e-22	3.6032300369e-22	-
YJR029W	4.624316	3.216287	0.523843838	1.62775590018e-22	3.68530584548e-22	-
YNL040W	36.868618	53.851315	-0.546588236	1.69886715747e-22	3.84490241108e-22	-
YHR169W	15.416606	26.03001	-0.755690678	1.73394632857e-22	3.922863997e-22	-
YGL212W	28.460941	45.54422	-0.678284611	1.76945360542e-22	4.00173696993e-22	-
YPL027W	18.095184	10.312107	0.811266639	1.80051983415e-22	4.07051242913e-22	-
YPL042C	18.109465	28.452497	-0.651811343	1.97839860493e-22	4.4710224024e-22	-
YFR037C	101.831459	132.000885	-0.374364277	2.00961835104e-22	4.53992384398e-22	-
YPL128C	8.235703	14.949056	-0.860090675	2.04687536796e-22	4.62240897937e-22	-

YAL028W	24.273884	18.781652	0.370081003	2.11777631552e-22	4.78078376974e-22	-
YGR005C	60.27515	84.353821	-0.484890084	2.17830764597e-22	4.91564299025e-22	-
YBL051C	58.416832	77.996758	-0.417030037	2.32743567732e-22	5.25026187674e-22	-
YGL028C	33.760368	27.881248	0.276035491	2.38421373383e-22	5.37638897915e-22	-
YGR133W	75.84346	59.700687	0.345277249	2.44608469101e-22	5.51390477915e-22	-
YIL096C	12.547023	7.022737	0.837239788	2.54063897901e-22	5.72496797884e-22	-
YPL013C	326.139984	297.974854	0.130300736	2.61091468156e-22	5.88118952943e-22	-
YCL028W	69.712425	61.271473	0.186200279	2.71824046146e-22	6.12072425998e-22	-
YDR223W	26.973829	20.828943	0.372970701	2.76155241879e-22	6.21599616237e-22	-
YGL087C	233.472916	208.388916	0.163976656	2.89619244271e-22	6.51669542746e-22	-
YEL027W	514.787354	650.326721	-0.33718809	3.01703833509e-22	6.78614999429e-22	-
YPL192C	10.54879	2.785392	1.92112524	3.05367325113e-22	6.86606430388e-22	up
YNL336W	72.97966	64.042976	0.18845408	3.11289603253e-22	6.99669028601e-22	-
YDL044C	14.357931	24.412819	-0.765791026	3.16122581923e-22	7.10274697278e-22	-
YPR135W	7.277795	4.820292	0.594380871	3.20561500873e-22	7.19987625695e-22	-
YML061C	5.834677	3.508316	0.733874093	3.61903606273e-22	8.12548856326e-22	-
YDR368W	164.053085	153.77594	0.093332929	4.08646032146e-22	9.17163618063e-22	-
YOR004W	15.886315	29.631853	-0.899364332	4.24810099204e-22	9.53097613249e-22	-
YDR237W	43.065556	65.231232	-0.599028419	4.37680589032e-22	9.81618893319e-22	-
YGR220C	88.008797	123.176979	-0.485013009	4.68538330488e-22	1.05044635452e-21	-
YLR070C	20.485107	13.9114	0.558307813	4.7350147909e-22	1.06119031848e-21	-
YOR296W	7.273928	11.519399	-0.663258899	4.86322454267e-22	1.0895307817e-21	-

YLR191W	37.871952	56.171822	-0.568716817	5.09921217034e-22	1.1419880647e-21	-
YHR067W	24.880905	16.682867	0.57667172	5.22802101457e-22	1.17041307099e-21	-
YKL054C	29.10743	41.179596	-0.500542212	5.34573751537e-22	1.19633520171e-21	-
YMR263W	39.916142	27.882235	0.517626077	5.73181260716e-22	1.28227360956e-21	-
YIL068C	17.311399	13.676247	0.340049935	6.85326451705e-22	1.53260324646e-21	-
YGR174W-A	225.979126	148.716812	0.603621766	7.31892810328e-22	1.63615072615e-21	-
ICR1	6.900227	4.715817	0.549136089	7.59662623584e-22	1.69761899468e-21	-
YDR312W	18.618214	29.93783	-0.685254968	8.56109724965e-22	1.91246101928e-21	-
YJL154C	38.469776	51.56102	-0.422555375	9.02209711463e-22	2.01471866502e-21	-
YOR037W	25.294331	40.054123	-0.663136673	9.08030979371e-22	2.02698896797e-21	-
YPL007C	11.412058	7.602066	0.586095529	9.8405131601e-22	2.19589883889e-21	-
YPR032W	8.754781	13.912529	-0.668241699	1.08445873605e-21	2.41908725598e-21	-
YDR390C	28.88854	23.989645	0.268085485	1.11501131672e-21	2.48634707406e-21	-
YMR028W	33.531548	50.828415	-0.600116156	1.11625932046e-21	2.48823621595e-21	-
YJL062W	12.83713	19.870073	-0.630274478	1.14360946086e-21	2.54828698242e-21	-
YLR149C	243.31427	295.151398	-0.278634241	1.20221557408e-21	2.67791685824e-21	-
YML091C	82.824821	81.748611	0.0188689625521	1.21263026803e-21	2.70014659395e-21	-
YGR206W	73.492271	50.535229	0.540303067	1.25026844894e-21	2.78295680568e-21	-
YKL011C	11.928403	6.775243	0.816056309	1.28764413986e-21	2.86512359148e-21	-
YIL079C	5.087027	11.628832	-1.192811543	1.40841865931e-21	3.13273487531e-21	down
YHR056C	13.234053	20.185503	-0.609064575	1.48444695374e-21	3.3006614215e-21	-
YMR285C	16.747734	26.78651	-0.677540716	1.50116423138e-21	3.33663714587e-21	-

YHR025W	53.139122	76.040878	-0.517000799	1.51203579891e-21	3.35959851097e-21	-
YPL077C	16.780666	31.094889	-0.889877492	1.59035512694e-21	3.53235228196e-21	-
YGL113W	6.181112	11.39956	-0.883039829	1.68401561038e-21	3.73904467426e-21	-
YMR287C	10.096176	15.82377	-0.648284396	1.69455587348e-21	3.76110220327e-21	-
YFL034W	6.911867	11.339327	-0.714187655	1.7319815163e-21	3.84279530136e-21	-
YNL288W	41.059418	60.30386	-0.554537173	1.76368168122e-21	3.91173128868e-21	-
YMR170C	85.216148	78.839149	0.112214635	1.77277962442e-21	3.93050568158e-21	-
YBR166C	25.389278	38.798309	-0.611774405	1.80039272326e-21	3.9903027583e-21	-
YDR297W	79.26619	108.819656	-0.457161634	1.91169262609e-21	4.23547031505e-21	-
YLR010C	49.234135	34.477596	0.513999728	1.97259539449e-21	4.36884488369e-21	-
YJL157C	10.719891	7.734601	0.470891461	2.00575396635e-21	4.44069922364e-21	-
YHR058C	30.601809	48.529556	-0.665246722	2.10483210451e-21	4.65839490367e-21	-
YLR449W	28.895048	44.228748	-0.614162137	2.16701048522e-21	4.79429832225e-21	-
YDR137W	23.132385	33.942703	-0.553187439	2.33247185108e-21	5.15852698665e-21	-
YJR112W-A	105.687668	80.878151	0.385985127	2.35398459152e-21	5.20425083482e-21	-
YBL010C	18.084028	11.077616	0.707068622	2.36075603102e-21	5.21736327538e-21	-
YOR389W	13.171521	9.330782	0.497352051	2.39252749283e-21	5.28569774929e-21	-
YBR302C	44.739513	36.775723	0.282795848	2.50308440006e-21	5.52797863947e-21	-
YHR127W	20.726732	12.689889	0.707813214	2.50335390568e-21	5.52660776901e-21	-
YDL161W	48.329762	67.938797	-0.491323782	2.62533156742e-21	5.79383518328e-21	-
YNR046W	99.954079	149.057266	-0.576529355	2.66327600228e-21	5.87548593537e-21	-
YGR100W	27.171467	37.537746	-0.466249565	2.72630230708e-21	6.01239244132e-21	-

YMR234W	21.171064	34.678505	-0.711947928	2.93086712812e-21	6.46122980518e-21	-
YNL073W	38.208878	53.66008	-0.489941312	3.18899613605e-21	7.02779127177e-21	-
YDL164C	7.43224	4.729084	0.652236322	3.35612304263e-21	7.39347475111e-21	-
tL(CAA)C	97.385963	44.11964	1.142292821	3.3678914143e-21	7.41676832208e-21	up
YHL031C	153.269119	138.651917	0.144599486	3.39552916248e-21	7.47498051087e-21	-
YPR129W	72.381638	100.152634	-0.468504703	3.41544257213e-21	7.51615295562e-21	-
YDR138W	5.901929	10.677566	-0.855324345	3.42007532411e-21	7.52368093979e-21	-
YBL005W-B	6.161479	4.66121	0.402572185	3.9207378181e-21	8.62201217668e-21	-
YJL191W	200.517792	274.823547	-0.454775367	3.98341207224e-21	8.75673588686e-21	-
YGR038W	13.846331	27.221558	-0.975245899	4.16752005823e-21	9.15821752973e-21	-
YIL014W	15.993621	24.861229	-0.636401011	4.37559405832e-21	9.61206224843e-21	-
YHR135C	107.287415	138.222641	-0.365513095	4.53440514471e-21	9.95740613313e-21	-
YOR016C	53.111328	40.396557	0.394787266	4.58478153296e-21	1.00644709182e-20	-
YBR020W	8.135663	4.719093	0.785750317	4.73593708525e-21	1.03926113203e-20	-
YER136W	222.005142	219.625717	0.0155460959964	4.74896421539e-21	1.04175158478e-20	-
YIL122W	86.395058	117.311691	-0.441326102	4.85705761594e-21	1.06508702507e-20	-
YMR228W	17.033487	29.104925	-0.772889494	4.99870586121e-21	1.09576151082e-20	-
YOL009C	35.180672	25.928602	0.440238618	5.1575299442e-21	1.13017811132e-20	-
YKL163W	352.375031	351.445648	0.00381010977514	5.65669015016e-21	1.23912252831e-20	-
YPR031W	18.50127	27.438597	-0.568582409	5.90960435607e-21	1.29406786041e-20	-
YDR173C	32.200459	24.732428	0.380677376	7.12213433268e-21	1.55903420089e-20	-
YFL002W-B	26.991867	40.886768	-0.599109256	7.18727785454e-21	1.57273954604e-20	-

YKL093W	232.060471	226.145706	0.0372481933752	7.19799318627e-21	1.57452930586e-20	-
YNL339C	9.954226	8.331354	0.256758163	8.20986617316e-21	1.79523949288e-20	-
YER048C	188.693176	183.093414	0.0434623521895	8.58944240626e-21	1.87757952317e-20	-
YPR054W	10.843801	19.730095	-0.86352736	9.42939896917e-21	2.06046141502e-20	-
YJL212C	4.862886	9.015014	-0.890516961	9.82236092494e-21	2.14557412463e-20	-
YDR140W	54.242519	81.93145	-0.594993169	1.00390574624e-20	2.192137486e-20	-
YHR086W	36.343838	51.732014	-0.509346579	1.06134095853e-20	2.3167386324e-20	-
YDL111C	38.104118	59.010406	-0.631022463	1.07095434136e-20	2.33690142397e-20	-
YPR078C	9.808983	5.34836	0.875006988	1.08758108613e-20	2.37234834247e-20	-
YJR101W	39.956524	61.377544	-0.619279836	1.12065920198e-20	2.4436432476e-20	-
YDL199C	39.381855	53.960026	-0.454359975	1.35108706051e-20	2.94506617684e-20	-
YLR380W	37.020531	54.1534	-0.548726331	1.38561217843e-20	3.01926304097e-20	-
YLR393W	19.679224	33.819908	-0.781199402	1.40205889202e-20	3.05402863216e-20	-
YDL022W	548.233582	564.067566	-0.041077281	1.46998369567e-20	3.20086242818e-20	-
YDR466W	40.768688	54.303753	-0.413590379	1.51510612243e-20	3.29795890886e-20	-
YKR064W	15.194487	22.63928	-0.57528011	1.52646086071e-20	3.32151034815e-20	-
YJL185C	60.824005	87.122398	-0.518402849	1.53676229435e-20	3.34275414272e-20	-
YAL049C	220.809647	285.07486	-0.368537614	1.60619623719e-20	3.49256260613e-20	-
YKL007W	235.309296	300.67804	-0.353661189	1.79500182324e-20	3.90174065779e-20	-
YJR025C	113.375061	96.356728	0.234646017	1.81544203387e-20	3.94478969067e-20	-
YLR231C	29.093958	43.30481	-0.573807702	1.84132018747e-20	3.99962061715e-20	-
YAL024C	2.405008	1.289644	0.899068821	1.97138714911e-20	4.28064757667e-20	-

RDN58-1	31.683964	73.372276	-1.211482195	1.97820017721e-20	4.29393940564e-20	down
YHR143W-A	141.87027	219.627808	-0.630488438	2.02867864698e-20	4.40197030426e-20	-
YOR321W	8.606057	5.789464	0.571922609	2.08153755511e-20	4.51508914818e-20	-
YOR186W	45.289223	30.583031	0.566436393	2.19734953454e-20	4.76463356983e-20	-
YAL064C-A	15.072372	5.761376	1.387421158	2.29142718067e-20	4.96689243631e-20	up
YBR122C	136.550903	188.938354	-0.46847674	2.33713641917e-20	5.06420345208e-20	-
YOL011W	17.499224	26.354599	-0.590763793	2.33787744269e-20	5.06404157859e-20	-
YGR161W-A	26.751734	40.429867	-0.59578905	2.35140145165e-20	5.09155919491e-20	-
YGR227W	9.971227	17.269993	-0.792424548	2.41455073458e-20	5.22647523022e-20	-
YKR018C	338.46402	404.483826	-0.257079537	2.51051135436e-20	5.43229504631e-20	-
YOL055C	11.781332	19.574728	-0.732489601	2.79101971717e-20	6.03716041959e-20	-
YDL103C	18.560905	13.567735	0.452087159	2.83515139636e-20	6.1304841061e-20	-
YJL125C	24.846674	38.706329	-0.639516741	2.85155055246e-20	6.16379729445e-20	-
YKL084W	13.016354	30.897463	-1.24716299	3.01869306281e-20	6.52281466547e-20	down
YIL075C	140.142365	141.165955	-0.010499048	3.23556528942e-20	6.98900115403e-20	-
YAR007C	22.095364	17.703146	0.319737936	3.47426661523e-20	7.50199900778e-20	-
YPR201W	11.999917	7.392132	0.698962003	3.48910772812e-20	7.53142586098e-20	-
YGR113W	40.600243	32.611362	0.316113666	3.52272505921e-20	7.60134764254e-20	-
YKL162C	37.333424	30.377411	0.297468913	3.67639470531e-20	7.93018010095e-20	-
YBR196C	400.411652	480.077301	-0.261782767	3.71687087424e-20	8.01470454577e-20	-
YOL081W	82.807945	85.832672	-0.051757717	3.72360467839e-20	8.02643675119e-20	-
YGL013C	47.375744	61.410503	-0.37433682	3.74944881552e-20	8.07933989821e-20	-

YOR332W	634.701721	773.297852	-0.284945451	4.1948463386e-20	9.03594936504e-20	-
YMR128W	12.179158	9.963819	0.289643677	4.54741809838e-20	9.79201233256e-20	-
YPL085W	8.998868	12.706817	-0.497787251	4.56514298406e-20	9.8267710281e-20	-
YNL172W	5.966641	4.535778	0.395568945	4.92259018001e-20	1.05925268068e-19	-
YPL264C	16.406319	10.736749	0.611694362	4.94390644156e-20	1.06347093518e-19	-
YHR151C	17.416325	27.244141	-0.645505769	5.10561039948e-20	1.09787424177e-19	-
YHR066W	11.209839	19.431492	-0.793631121	5.8312195912e-20	1.25346991767e-19	-
YGL154C	59.820808	49.46228	0.274318654	5.94650158789e-20	1.27780830244e-19	-
YIL151C	6.294485	10.277735	-0.707362109	5.9579373719e-20	1.27982267144e-19	-
YBR231C	67.482811	57.738564	0.224984843	5.99750021026e-20	1.28787552077e-19	-
YMR200W	239.392853	305.874969	-0.353561968	6.19643834616e-20	1.33013448316e-19	-
YOR008C	171.291458	218.079712	-0.348402353	6.4025687811e-20	1.37390760432e-19	-
YFR014C	142.370438	181.468445	-0.35006909	6.9513505372e-20	1.49115356375e-19	-
YNL051W	37.76207	30.866949	0.290874962	6.95499968199e-20	1.49142100262e-19	-
YBR060C	10.871012	7.451671	0.544850365	7.27623235257e-20	1.55976693525e-19	-
YBR285W	95.645782	76.915039	0.314435635	7.54752000661e-20	1.61736293411e-19	-
YFL048C	44.920589	63.220695	-0.493020051	8.31776036138e-20	1.78180318576e-19	-
YDR545W	20.670387	19.003809	0.121276788	8.33670889025e-20	1.78524625011e-19	-
YAL047C	8.532379	5.452607	0.646001874	8.52045652973e-20	1.82396531505e-19	-
YLR459W	21.737431	34.267471	-0.656658274	9.12601003752e-20	1.95292210662e-19	-
YDR200C	24.651499	35.9314	-0.543569772	9.25278092531e-20	1.97936815935e-19	-
YDR049W	14.021248	21.996254	-0.649643085	9.36462260011e-20	2.00260341376e-19	-

YPL252C	52.394901	38.746391	0.435364479	9.7109658977e-20	2.07595304039e-19	-
YDR059C	388.96109	372.043396	0.0641549309763	9.71362971447e-20	2.07580768563e-19	-
YPR117W	8.343684	7.165156	0.219686405	1.00470356257e-19	2.14631786526e-19	-
YDL075W	308.814301	411.441986	-0.413949458	1.05940639537e-19	2.26239934726e-19	-
YBR006W	153.244354	149.648041	0.0342605284612	1.07176782177e-19	2.28801053562e-19	-
YMR124W	23.501728	20.365345	0.206650582	1.09753614717e-19	2.34221533229e-19	-
YNL231C	22.188643	35.505764	-0.678231812	1.12085266096e-19	2.39115234337e-19	-
YKR077W	18.093882	12.34425	0.551662783	1.1209587145e-19	2.39055709364e-19	-
YDR363W	41.929626	59.259258	-0.4990706	1.17022336934e-19	2.49476190827e-19	-
YNR051C	19.188265	29.574152	-0.624112533	1.17976738122e-19	2.51424507471e-19	-
YGR050C	80.77005	60.602295	0.414448003	1.20274706951e-19	2.56233830046e-19	-
YDL065C	81.611465	72.835785	0.164124404	1.23845901046e-19	2.63751407785e-19	-
YDL116W	21.384747	30.975401	-0.53454082	1.25951270637e-19	2.68143171508e-19	-
YBL058W	449.204254	460.856659	-0.036946506	1.3032104562e-19	2.7735106315e-19	-
YDL238C	22.529196	34.130699	-0.599274132	1.40951539827e-19	2.99872227296e-19	-
YDR162C	85.652756	73.409447	0.222533934	1.54134727108e-19	3.27806915344e-19	-
YBR260C	12.633677	19.939133	-0.658328086	1.67837835659e-19	3.56827836909e-19	-
YNL125C	99.472771	96.330933	0.0463025272887	1.70163323356e-19	3.61648035396e-19	-
YKL167C	113.819344	164.488068	-0.531237165	1.79663393642e-19	3.81707853432e-19	-
YHR148W	20.372807	37.507999	-0.880553528	1.88903507293e-19	4.01201838273e-19	-
YHR085W	9.011898	17.433523	-0.951961252	1.92337267509e-19	4.08354909952e-19	-
YBR252W	22.336458	12.01146	0.894988907	1.97644226691e-19	4.19478755316e-19	-

YIR002C	19.301592	16.420473	0.233224161	2.0032565855e-19	4.25024500436e-19	-
YDR242W	46.202984	63.291267	-0.454020418	2.11591596287e-19	4.48773703364e-19	-
YER115C	46.963364	34.854408	0.430194629	2.20116763984e-19	4.66695652599e-19	-
YLR262C	26.651239	17.575699	0.600620537	2.26680736166e-19	4.80448620731e-19	-
YDR151C	142.901398	134.491806	0.0875017520104	2.28841621028e-19	4.84863065988e-19	-
YNL138W	97.133659	92.179955	0.0755182452129	2.45712580244e-19	5.20431149149e-19	-
YDR117C	16.524548	25.603266	-0.631717045	2.63593116599e-19	5.58112574299e-19	-
YOR177C	8.326788	4.805632	0.793033918	2.78495231775e-19	5.89464165993e-19	-
YDR079C-A	58.763462	35.237259	0.737817688	2.82463017118e-19	5.97658626541e-19	-
YLR051C	20.549664	36.276756	-0.819930645	3.03945024829e-19	6.42892917934e-19	-
YJL091C	13.423788	9.2385	0.539061303	3.64912740103e-19	7.71586611226e-19	-
YGR074W	42.493973	70.327538	-0.726831477	3.7973557613e-19	8.02655245697e-19	-
YOL122C	24.501234	19.924757	0.298292283	3.95968705539e-19	8.36682683454e-19	-
YHR168W	7.331457	13.383668	-0.86830172	4.58879310025e-19	9.6928300668e-19	-
YGR221C	9.752415	16.216007	-0.733587192	4.66946252933e-19	9.85987189867e-19	-
YDR420W	16.511791	22.241034	-0.429727247	4.68234010983e-19	9.88370193874e-19	-
YDR005C	89.95237	82.683617	0.121559792	4.78390343686e-19	1.00946541591e-18	-
YGR143W	80.190598	102.085075	-0.348266955	4.80320742254e-19	1.01319441655e-18	-
YDR016C	48.612648	30.047529	0.694085368	4.95783190515e-19	1.04545585826e-18	-
YNL099C	105.57589	144.42038	-0.451993935	5.19357247076e-19	1.0947944957e-18	-
YDR488C	6.465656	3.574291	0.855139656	5.37491741663e-19	1.13263704421e-18	-
YDL033C	15.799948	25.841135	-0.709749628	5.46205169394e-19	1.15060797136e-18	-

YPL183C	4.177613	7.443756	-0.833351914	5.50396321653e-19	1.15904354302e-18	-
YAL008W	173.811188	159.382248	0.125029999	5.604295059e-19	1.17977157431e-18	-
YHR010W	225.750473	303.040344	-0.424780863	5.80358677079e-19	1.22131073468e-18	-
YMR129W	8.751564	13.091031	-0.580965953	5.81825529084e-19	1.2239826786e-18	-
YDR501W	6.177932	3.316453	0.897482911	5.83078573218e-19	1.22620317837e-18	-
YGR172C	38.847672	59.708851	-0.620116661	5.8427128309e-19	1.2282953354e-18	-
YJL046W	25.279282	38.475052	-0.605967789	5.95102596845e-19	1.25064215342e-18	-
YCL063W	5.386971	2.429582	1.148766173	6.00317140443e-19	1.26117387745e-18	up
YOR347C	72.245712	66.652946	0.116243319	6.18990090987e-19	1.29996295157e-18	-
YCR008W	189.385452	190.009048	-0.004742608	6.28915630211e-19	1.32036125544e-18	-
YLL054C	6.543419	11.05363	-0.756403667	6.45659117133e-19	1.35505469884e-18	-
YDR240C	8.388353	15.008222	-0.839293594	6.5315199196e-19	1.37031685235e-18	-
YCL024W	2.149876	0.963337	1.158140968	7.368167478e-19	1.54532377376e-18	up
YBR084W	4.454543	7.883327	-0.823527188	7.37106356788e-19	1.54540907225e-18	-
YCL012C	50.794674	35.489067	0.517302587	7.51202903518e-19	1.57443201386e-18	-
YJR057W	35.785728	25.815205	0.471163274	7.70245975684e-19	1.61379919576e-18	-
YGL133W	13.561927	11.407767	0.249545763	7.74267314708e-19	1.62167729072e-18	-
YPL178W	143.902283	130.168152	0.14471297	8.50765269819e-19	1.78129875043e-18	-
YER078C	13.948413	22.466206	-0.687655522	8.57242603077e-19	1.79425558999e-18	-
YML082W	11.033184	17.742657	-0.685372883	9.61944060876e-19	2.01272286145e-18	-
YOL103W-B	6.808967	5.396324	0.33545897	9.90024136198e-19	2.07077824714e-18	-
YML027W	9.0951	5.117534	0.829640718	9.91038042883e-19	2.07220079832e-18	-

YER187W	23.628572	44.181004	-0.902893762	1.02801199976e-18	2.14878737189e-18	-
YIL009W	12.581285	9.361094	0.426530233	1.04240315215e-18	2.17813489349e-18	-
YDR246W	300.353119	291.479034	0.0432675309937	1.05927286321e-18	2.21263995114e-18	-
YDR305C	42.083416	31.613501	0.412711002	1.08653288114e-18	2.26881807135e-18	-
YML093W	8.502447	13.526536	-0.669842415	1.10280057933e-18	2.30201277622e-18	-
YDL145C	17.465307	24.289547	-0.475843582	1.15147756998e-18	2.40281437124e-18	-
YDL243C	20.983797	14.753084	0.508259187	1.1904827607e-18	2.4833726406e-18	-
YKL113C	19.100494	13.63173	0.486641286	1.22323149173e-18	2.55083006405e-18	-
YCL016C	12.952222	8.248925	0.650921594	1.27563084838e-18	2.65920628165e-18	-
YGL081W	9.539154	5.077064	0.909866877	1.34425047492e-18	2.80131149657e-18	-
YNR024W	23.239933	14.192381	0.711489265	1.44319896924e-18	3.00650308759e-18	-
YLR069C	28.315987	39.232063	-0.470416383	1.70879416858e-18	3.55860254899e-18	-
YLR343W	1.355229	3.98916	-1.557548337	1.78645314217e-18	3.71908152468e-18	down
YNL321W	40.981838	53.993355	-0.397797175	1.79489762455e-18	3.73540880093e-18	-
YNL230C	19.335482	30.88797	-0.675794329	1.88920278985e-18	3.93035218477e-18	-
YBR086C	52.164711	49.571373	0.0735669470614	1.93265031183e-18	4.01939468537e-18	-
YDR386W	13.633423	10.141161	0.426925004	1.93634865423e-18	4.02573759057e-18	-
YMR172W	16.741476	24.830738	-0.568700413	1.9384579579e-18	4.02877368686e-18	-
YPL076W	16.538958	28.706299	-0.795498997	1.94977378503e-18	4.05093562833e-18	-
YDR047W	60.858849	84.073296	-0.466180584	2.02993577262e-18	4.21607269201e-18	-
YDR014W	7.077104	4.416311	0.680317349	2.07390600759e-18	4.30595601843e-18	-
YNL251C	15.500773	11.630536	0.414422577	2.09401198344e-18	4.34624754036e-18	-

YPL283C	7.63086	6.253887	0.287092507	2.15069263709e-18	4.46239969621e-18	-
YGR006W	31.102898	49.117119	-0.659176932	2.44197871976e-18	5.06508649926e-18	-
YKR001C	83.438194	106.279259	-0.349080238	2.49242098753e-18	5.1679858018e-18	-
YML003W	16.190323	10.230236	0.662292341	2.5051251748e-18	5.19259335064e-18	-
YPR200C	21.607704	11.116823	0.958801233	2.58658148949e-18	5.35964548957e-18	-
YGR141W	138.042297	174.763397	-0.340292669	2.71388535417e-18	5.6215549812e-18	-
YER172C	10.578188	14.547719	-0.459700444	2.76452247759e-18	5.7245348702e-18	-
YMR045C	12.575277	11.007228	0.192138985	2.8009457239e-18	5.79802302566e-18	-
YOR132W	105.477913	134.415115	-0.349754447	2.80150160349e-18	5.79724065148e-18	-
YHR141C	513.84021	657.509277	-0.355691459	2.90526076759e-18	6.00994963185e-18	-
YPL198W	43.186253	65.14624	-0.593109766	3.05426028029e-18	6.31607189208e-18	-
YLR466W	29.205938	27.305382	0.09707638	3.16326126819e-18	6.53930268163e-18	-
YIL002W-A	130.506683	201.237991	-0.624779006	3.18378106955e-18	6.57953158448e-18	-
YNL084C	139.723007	132.810669	0.0731985501405	3.38772367566e-18	6.99866508436e-18	-
YJL033W	12.335539	18.876617	-0.613779478	3.63391657557e-18	7.50477515007e-18	-
YOL045W	14.198245	20.341024	-0.518679695	3.77760174266e-18	7.79891972678e-18	-
YBR220C	13.784258	21.755754	-0.658375408	3.87181097981e-18	7.99075883067e-18	-
YFR027W	9.545013	4.826977	0.98362721	3.99727179719e-18	8.24694693153e-18	-
YCR019W	10.614192	19.07029	-0.845332232	4.04298688864e-18	8.3384925597e-18	-
YDR120C	30.051178	42.558331	-0.502020029	4.31578082559e-18	8.89816252583e-18	-
YNL277W	24.896248	36.805313	-0.563985704	4.51079894545e-18	9.29715798583e-18	-
YHL014C	10.707973	18.758518	-0.808860447	4.82027544248e-18	9.9317191991e-18	-

YML057W	77.14798	99.706619	-0.370060898	4.86709973306e-18	1.00248689923e-17	-
YER099C	73.756165	101.025848	-0.453888912	4.89039429891e-18	1.00695083939e-17	-
YPR104C	8.789419	13.728221	-0.643304975	4.99963768652e-18	1.02910314184e-17	-
YPL255W	11.152451	6.887822	0.695241044	5.12435167794e-18	1.05442410397e-17	-
YPL169C	64.970879	85.116867	-0.389651825	5.14935625166e-18	1.05921814481e-17	-
YBR141C	19.289297	31.296019	-0.698178586	5.3743972671e-18	1.10514270401e-17	-
YDL165W	102.51947	142.987793	-0.479994063	5.42876798389e-18	1.11595336569e-17	-
YMR218C	6.799539	10.776887	-0.664431661	5.48853604442e-18	1.12786599681e-17	-
YJL026W	619.30072	736.571289	-0.25018504	5.57288186917e-18	1.14481967716e-17	-
YAL034W-A	9.638047	4.999437	0.946975198	5.62404338335e-18	1.15494744703e-17	-
YLR103C	3.036867	1.299489	1.224639305	5.8652741715e-18	1.20408803097e-17	up
YGR001C	47.486031	70.366028	-0.567375901	6.22503542709e-18	1.27752132004e-17	-
YBR058C-A	191.345856	159.709259	0.260734703	6.33934497819e-18	1.30055035111e-17	-
YBR034C	35.881718	52.7411	-0.555678694	6.95408062756e-18	1.42619532659e-17	-
RDN37-2	0.496434	1.241794	-1.322752034	6.9848602017e-18	1.43203474677e-17	down
YNL024C-A	198.797699	286.850006	-0.52899549	7.06455324954e-18	1.44789523186e-17	-
YDR181C	42.23753	36.698372	0.202809406	7.07680453226e-18	1.44992747644e-17	-
YLL013C	29.040356	39.408905	-0.440462525	7.8169352809e-18	1.6010403901e-17	-
YOR071C	15.548864	23.7911	-0.613612797	8.02289169611e-18	1.64268178263e-17	-
YLR204W	147.702393	209.738525	-0.505898681	8.1402071644e-18	1.66615252478e-17	-
YER138C	5.065604	3.826732	0.404621432	8.23049163501e-18	1.68407686454e-17	-
YDR517W	71.932762	64.687508	0.153161864	8.47228358184e-18	1.73297978504e-17	-

YNL092W	22.510918	34.599411	-0.620122587	8.7191810990 6e-18	1.7828944750 6e-17	-
YJR146W	34.079884	20.633368	0.723941092	9.9091793506 3e-18	2.0255576361 1e-17	-
YOL103W	24.830086	35.592258	-0.519474203	9.9509806459 e-18	2.0334327797 8e-17	-
YLR396C	15.228132	22.877094	-0.587164822	1.1099365772 7e-17	2.2673531660 8e-17	-
YBR131W	4.197405	7.953057	-0.922011838	1.1173633425 4e-17	2.2817735626 6e-17	-
YBL005W	8.446642	13.141356	-0.637664338	1.1586646000 2e-17	2.3653370065 5e-17	-
YPL056C	142.317673	204.961594	-0.526238774	1.2085826207 9e-17	2.4664302793 8e-17	-
YLR467W	21.089432	19.665319	0.100866651	1.2610294605 9e-17	2.5726161325 6e-17	-
YPL038W	35.639164	57.913307	-0.700431385	1.2632250291 1e-17	2.5762486796 e-17	-
YEL070W	18.741919	28.569496	-0.608206906	1.2696898585 1e-17	2.5885828051 3e-17	-
YKR056W	27.962498	39.30888	-0.491362011	1.2736790807 2e-17	2.5958633398 3e-17	-
YLR335W	49.68354	65.39447	-0.396400671	1.3040962743 3e-17	2.6569838106 5e-17	-
YDL001W	17.890581	28.116098	-0.652196149	1.3238865684 6e-17	2.6964198874 8e-17	-
YEL042W	44.397648	39.264984	0.177239941	1.3781068247 6e-17	2.8059321640 3e-17	-
YCR076C	46.261944	68.490837	-0.566085099	1.4330817074 5e-17	2.9169086032 4e-17	-
YOL090W	4.659494	2.945576	0.661623516	1.4394411868 3e-17	2.9288924575 e-17	-
YGL245W	100.747551	98.777779	0.028486334	1.5018416876 8e-17	3.0548601563 3e-17	-
YPR137C-B	11.172997	9.688516	0.205668612	1.5872423174 4e-17	3.2275140211 8e-17	-
YDR412W	17.315208	30.504084	-0.816962691	1.7202959253 4e-17	3.4969211213 2e-17	-
YJL134W	21.46648	33.021328	-0.621312499	1.7657415146 4e-17	3.5881254739 5e-17	-
YJR063W	16.088873	33.491146	-1.057716472	1.8004558865 3e-17	3.6574705967 2e-17	down
YNL072W	19.179705	13.166128	0.542749402	1.8188117433 9e-17	3.6935503117 3e-17	-

YPL263C	6.590632	4.105792	0.682756275	1.84669566954e-17	3.74894922057e-17	-
YFL010W-A	67.047272	108.66703	-0.696663749	1.90596219402e-17	3.86800042512e-17	-
YDL160C	127.87748	124.63868	0.0370103596709	2.20357539817e-17	4.4705215921e-17	-
YKL139W	21.392431	31.724476	-0.568495898	2.24927474289e-17	4.56174374513e-17	-
YFL018C	479.273834	498.538666	-0.056855224	2.28312169778e-17	4.62887638792e-17	-
YGL018C	102.870636	143.35173	-0.478728086	2.30883793389e-17	4.67948608998e-17	-
YDR319C	116.891884	155.055023	-0.407605498	2.31055711279e-17	4.68144208754e-17	-
YLR139C	5.412629	9.844817	-0.863034885	2.31141803829e-17	4.6816584606e-17	-
YNL085W	20.524233	28.94224	-0.495848276	2.37451992321e-17	4.80789944007e-17	-
YNR073C	14.725068	23.232267	-0.657855643	2.39954781921e-17	4.85699147756e-17	-
YLL002W	7.477615	4.2283	0.822500453	2.48359989907e-17	5.02548506304e-17	-
YOR089C	369.085876	460.542023	-0.319376273	2.5929578766e-17	5.24505783576e-17	-
YDR098C-B	8.208086	6.81822	0.267650696	2.60591847106e-17	5.26955761184e-17	-
YIL077C	340.67865	342.756653	-0.008773131	2.6286874403e-17	5.31386897733e-17	-
YOR273C	67.793777	64.257591	0.0772859544397	2.66948733641e-17	5.39458899232e-17	-
YER190W	9.24175	7.765938	0.251005874	2.68380691442e-17	5.42176157653e-17	-
YOR017W	12.825148	19.260519	-0.586671105	2.75969707807e-17	5.57325942116e-17	-
YNR032C-A	91.265488	65.044502	0.488642293	2.77049165399e-17	5.59323973592e-17	-
YBR094W	17.793201	14.611755	0.284196606	2.9184460389e-17	5.8900237352e-17	-
YKL038W	47.815666	60.839245	-0.347518879	2.95869730116e-17	5.96931844187e-17	-
YGL027C	20.74682	29.185329	-0.492353109	3.00097890263e-17	6.05265660414e-17	-
YJL170C	12.87694	6.706352	0.941189687	3.09155652954e-17	6.23331696504e-17	-

YGR020C	181.003281	248.880142	-0.459435274	3.1995971742e-17	6.4490582004 7e-17	-
YER052C	58.125687	77.33165	-0.411883133	3.2963657834 1e-17	6.6419470248e-17	-
YIL017C	55.478855	70.997284	-0.355825822	3.3193212123 6e-17	6.6860305276 9e-17	-
YHL006C	30.711828	19.734217	0.638095107	3.5205307861 8e-17	7.0890220955 6e-17	-
YPL001W	6.608778	12.988262	-0.974752953	3.8402522711 1e-17	7.7303132617e-17	-
YDL073W	4.107819	7.2173	-0.81308661	3.8820099133e-17	7.8118371286e-17	-
YPR167C	8.694997	17.449455	-1.004924542	3.9076887212 5e-17	7.8609629233 8e-17	down
YHR106W	41.661087	34.62537	0.266870994	4.1983591435e-17	8.4429587181 3e-17	-
YKL068W-A	5.941107	20.53776	-1.789475162	4.5440679090 5e-17	9.1352246047 2e-17	down
YOL110W	193.285233	249.025955	-0.365564697	4.5597687995 5e-17	9.1638215304 6e-17	-
YJL063C	102.679771	139.24144	-0.439436656	4.8117634555 9e-17	9.6671286512 4e-17	-
YGL098W	53.079769	77.066818	-0.537947733	4.9721922596 3e-17	9.9862082005 1e-17	-
YIL006W	26.840094	40.331139	-0.587504426	5.4507659418 3e-17	1.0943840545 6e-16	-
YHR202W	28.909277	24.780767	0.222311687	5.4579178572 1e-17	1.0954656986e-16	-
YKL015W	27.785767	37.307671	-0.425126235	5.5826498056 3e-17	1.1201386552 5e-16	-
YAL011W	8.892666	14.6668	-0.721866233	5.7017470884 4e-17	1.143665458e-16	-
YER118C	17.21064	12.153685	0.501906941	5.8945218379 7e-17	1.1819506321 1e-16	-
YFL053W	5.816779	3.389302	0.7792323	5.9485419275 8e-17	1.1923974261e-16	-
YMR125W	26.655817	23.685148	0.170467714	5.9528711999 2e-17	1.1928800648 5e-16	-
YOR162C	38.581741	35.471931	0.121240377	6.0291537033 4e-17	1.2077762565 4e-16	-
YGR232W	72.286072	61.526314	0.232514133	6.1635183559 3e-17	1.2342942565 7e-16	-
YNL298W	8.111036	5.83927	0.474098177	6.3416491558e-17	1.2695568513 1e-16	-

YDR334W	17.203907	23.178251	-0.430035469	6.91866379601e-17	1.38462491443e-16	-
YDR105C	37.901131	52.975502	-0.483084454	7.43169792238e-17	1.48681858531e-16	-
YOL006C	18.181942	15.108592	0.267137082	7.88889279936e-17	1.57777855987e-16	-
YDL025C	105.157387	132.297089	-0.331231119	8.07593473089e-17	1.61466675715e-16	-
YDL235C	73.322021	107.111702	-0.546797647	8.12734549044e-17	1.62442243415e-16	-
YOR238W	20.436213	14.416741	0.503382807	8.46943436891e-17	1.6922513216e-16	-
YMR064W	74.111115	96.601418	-0.382354436	9.0686961095e-17	1.81140493719e-16	-
YKL001C	69.050835	99.223305	-0.523020152	9.07849698285e-17	1.81277932678e-16	-
YLR371W	23.788752	31.43491	-0.402088045	9.2765187263e-17	1.85172438112e-16	-
YGL103W	402.721161	508.938873	-0.337711109	1.06259546267e-16	2.12040907498e-16	-
YAR008W	11.808267	6.76653	0.80330916	1.0953510688e-16	2.18507051256e-16	-
YJL121C	126.783455	116.354538	0.12383901	1.09941693965e-16	2.19247682665e-16	-
YDR022C	71.731529	59.774975	0.263065761	1.16012738119e-16	2.31280371947e-16	-
YDR213W	15.956634	22.794418	-0.514524222	1.16313239119e-16	2.31805004318e-16	-
YKL027W	14.474225	10.335615	0.485861867	1.17743749511e-16	2.345806152e-16	-
YDR307W	16.116179	12.809058	0.331345353	1.28841836865e-16	2.56608958376e-16	-
YJR089W	8.775895	13.48796	-0.620053993	1.30436965823e-16	2.59702592634e-16	-
YPL256C	12.650699	9.180373	0.462592425	1.30968124087e-16	2.60676535535e-16	-
YJL005W	27.805565	35.262867	-0.342776123	1.42145957731e-16	2.82834008203e-16	-
YLR170C	86.907265	72.061691	0.270244278	1.4228402582e-16	2.83018017394e-16	-
YOR384W	23.80113	33.484909	-0.492480978	1.55933858642e-16	3.10069633072e-16	-
YDR178W	984.731445	1015.377136	-0.044213446	1.56515319748e-16	3.11126194362e-16	-

YJL023C	60.807522	53.69273	0.179523038	1.62428612635e-16	3.22777473507e-16	-
YHR077C	22.902376	30.90213	-0.432209005	1.70045047478e-16	3.37804689518e-16	-
YMR126C	30.37072	45.191463	-0.573369152	1.77761767803e-16	3.53021450583e-16	-
YFR050C	361.503845	362.332184	-0.003301966	1.78406333375e-16	3.54188205178e-16	-
YKL110C	23.363371	36.586048	-0.647045136	1.81355076054e-16	3.59927209764e-16	-
YOR028C	27.149885	41.921558	-0.626746248	1.8206469106e-16	3.6122007098e-16	-
YPR186C	2.852293	6.74578	-1.241863079	1.85737500712e-16	3.68389266588e-16	down
YHR039C-A	368.201599	475.942841	-0.37029243	1.97898231375e-16	3.92383334517e-16	-
YNR045W	71.349091	93.493851	-0.389976434	2.16962442617e-16	4.30045607843e-16	-
YEL025C	13.236936	11.244772	0.235314806	2.24194046637e-16	4.44237676834e-16	-
YGL176C	5.911563	10.744742	-0.862019311	2.26037339833e-16	4.47747225808e-16	-
YJR140C	4.156395	3.019317	0.461110532	2.403912278e-16	4.76028306916e-16	-
YGR237C	38.124001	50.580559	-0.407883448	2.43451475211e-16	4.81934552969e-16	-
YPL248C	25.391226	34.569973	-0.44518942	2.50596691305e-16	4.95921026338e-16	-
YDR303C	6.575114	10.684072	-0.70037379	2.55857170992e-16	5.06169954595e-16	-
YFL028C	44.747608	36.940289	0.276615411	2.62255048939e-16	5.18661785222e-16	-
YLR085C	35.552631	30.17486	0.23660925	2.65104843633e-16	5.24130850087e-16	-
YMR213W	29.697277	41.441071	-0.480730635	2.80659879626e-16	5.54707587621e-16	-
YFR013W	11.472864	8.871829	0.370922116	2.81368137546e-16	5.55930425807e-16	-
YJL194W	4.028547	1.895525	1.087662103	2.9097956856e-16	5.74737881521e-16	up
YPR063C	202.085983	184.753326	0.129368921	2.9184685514e-16	5.76267581651e-16	-
YGL163C	17.439945	14.826264	0.234240385	2.92551099638e-16	5.77474475851e-16	-

YGR241C	32.09914	27.868954	0.20387579	2.9999820436e-16	5.91986285019e-16	-
YHR046C	5.507526	11.922927	-1.114262146	3.08579555801e-16	6.08726368737e-16	down
YOR127W	8.722888	6.721896	0.375937644	3.25570275591e-16	6.42039476134e-16	-
YDR485C	48.614746	63.159321	-0.377601678	3.27912066907e-16	6.46452242414e-16	-
YKR004C	10.706833	6.721468	0.671683541	3.33370362282e-16	6.57004193347e-16	-
YOR026W	16.124846	11.130084	0.534820901	3.36561664253e-16	6.63083088442e-16	-
YBR043C	83.99511	106.167992	-0.337971636	3.41789955313e-16	6.73170064271e-16	-
YBR241C	176.538422	176.052856	0.00397357590379	3.48735504009e-16	6.86631782077e-16	-
YBR233W	7.470616	4.256325	0.811618892	3.72472237487e-16	7.33134955714e-16	-
YDL114W	0.462267	2.895185	-2.646857257	3.8168444242e-16	7.5102916594e-16	down
YML077W	13.030032	26.303276	-1.013401867	3.87728662367e-16	7.62680461335e-16	down
YGR044C	144.10025	185.608353	-0.3651888	4.0068056933e-16	7.87907815774e-16	-
YDR444W	13.757271	10.759179	0.35462632	4.09854306637e-16	8.05692063206e-16	-
YLR011W	22.486242	14.478297	0.635150655	4.30891501085e-16	8.4677886633e-16	-
YHR114W	39.324978	35.510948	0.147182083	4.50806795343e-16	8.8563562832e-16	-
YLR162W	30.701349	54.291241	-0.822417413	4.64130996014e-16	9.11523322763e-16	-
YJR147W	38.406174	32.088322	0.259289902	4.75402288203e-16	9.33364138256e-16	-
YFR035C	60.65472	44.482197	0.447391871	4.90311828241e-16	9.6233190949e-16	-
YOL092W	32.80748	48.790104	-0.572563776	4.91054443314e-16	9.63484824303e-16	-
YKL073W	8.5109	13.216372	-0.634942594	5.16435141138e-16	1.01296346167e-15	-
YPL052W	98.199112	90.102592	0.12414137	5.36571072039e-16	1.05212672622e-15	-
YDL183C	32.641449	48.326714	-0.566115799	5.40685765102e-16	1.05986019253e-15	-

YDR510W	455.716736	583.858093	-0.35748041	5.60785626238e-16	1.09891324738e-15	-
YOR091W	36.347267	52.538509	-0.531528369	6.24940427768e-16	1.22424429649e-15	-
YDL093W	7.811377	5.49441	0.507612321	6.5155526413e-16	1.27597952042e-15	-
YDR469W	43.724888	67.570549	-0.627939888	6.52105409168e-16	1.27665417222e-15	-
YLR061W	116.209663	165.662521	-0.511517213	6.63301530021e-16	1.29816390239e-15	-
YDR248C	46.321373	35.966515	0.365023641	6.7464188473e-16	1.31994226927e-15	-
YER059W	60.026848	80.571732	-0.424665854	7.38938532242e-16	1.44528368247e-15	-
YMR286W	153.083649	127.410881	0.264831705	7.71975472259e-16	1.50942479741e-15	-
YDR315C	11.926438	7.072455	0.753880229	7.83988453557e-16	1.53243083113e-15	-
YHL043W	28.135046	18.782379	0.582988514	7.84220288551e-16	1.53240149554e-15	-
YJL206C	17.581274	25.216906	-0.520351657	8.08006465579e-16	1.57838393276e-15	-
YOR329C	28.217014	37.951675	-0.427598234	8.32123834818e-16	1.62498419835e-15	-
YPL254W	6.330235	11.564277	-0.869344108	8.55317374711e-16	1.66975165478e-15	-
YLR283W	36.205318	52.915634	-0.547492409	8.67003935938e-16	1.69203408812e-15	-
YKL105C	17.487885	15.410524	0.182439901	9.06326868982e-16	1.76822036538e-15	-
YNL299W	4.784443	8.732421	-0.868030709	9.09186194319e-16	1.77324156278e-15	-
YOR304C-A	97.977272	73.418015	0.416313014	9.10005878061e-16	1.77428281753e-15	-
YER162C	52.11974	67.450668	-0.372002844	1.03808034234e-15	2.02336036585e-15	-
YML039W	7.958176	6.689394	0.250562284	1.07977314796e-15	2.10396475284e-15	-
YDR287W	133.480759	126.25412	0.0803013276066	1.12976824372e-15	2.20069069877e-15	-
YNL123W	8.003254	12.263531	-0.615715827	1.17080033393e-15	2.27990228137e-15	-
YGR077C	26.999817	23.157007	0.221500829	1.27101755026e-15	2.47427938288e-15	-

YHR111W	39.227921	54.5452	-0.475571367	1.28566741624e-15	2.50201357994e-15	-
YJL100W	68.229012	87.923012	-0.365855485	1.33057731326e-15	2.58860042643e-15	-
YNR057C	19.947887	13.361871	0.5781139	1.43010707697e-15	2.78136113215e-15	-
YHR219W	17.942873	14.573259	0.30008737	1.49777751677e-15	2.91205851052e-15	-
YPL091W	73.64727	69.155258	0.0907931013173	1.52717505446e-15	2.96828514029e-15	-
YMR001C-A	8.644444	1.410198	2.615875435	1.80911630695e-15	3.51517810126e-15	up
YJL008C	116.56913	145.739746	-0.322208599	1.92522799013e-15	3.73961682188e-15	-
YDR353W	90.739464	83.650055	0.117363649	1.97676274175e-15	3.83851832993e-15	-
YPR007C	11.890467	9.099273	0.38598219	2.00542434303e-15	3.89295632318e-15	-
YLR166C	18.924639	16.341837	0.211695613	2.04762908357e-15	3.9736421853e-15	-
YDL242W	15.200241	6.778897	1.164971742	2.05131525663e-15	3.97955159787e-15	up
YBR274W	16.904821	25.426947	-0.588923509	2.12083460287e-15	4.11313377526e-15	-
YOR060C	25.111856	39.661648	-0.659375964	2.12488549822e-15	4.11970305215e-15	-
YBR092C	18.328676	14.204435	0.367761127	2.19603684338e-15	4.25632117505e-15	-
YNL005C	115.750404	148.612411	-0.360537375	2.25663616531e-15	4.3724086499e-15	-
YDR342C	801.431274	933.07489	-0.21941407	2.64464734901e-15	5.12261177617e-15	-
YDR226W	126.226143	167.33046	-0.406689349	2.69078206533e-15	5.2103478046e-15	-
YGL088W	0.0001	4.045464	-15.30401756	2.71600441181e-15	5.25754767338e-15	down
YPL199C	72.548172	63.208805	0.198813723	2.94211593398e-15	5.69347123383e-15	-
YPR153W	64.187805	96.243217	-0.58438564	3.04796583545e-15	5.89646989918e-15	-
YBL095W	62.918365	87.386742	-0.473933235	3.05408084977e-15	5.90645916366e-15	-
YDR107C	9.48925	6.915267	0.456509111	3.07665480906e-15	5.94826317491e-15	-

YLR406C	133.402512	187.71344	-0.492746116	3.10376559097e-15	5.99880971007e-15	-
YLR443W	16.571527	25.554226	-0.624855348	3.19131520755e-15	6.16610171443e-15	-
YPL156C	85.746544	114.994049	-0.423408773	3.33157009011e-15	6.43509244537e-15	-
YJR098C	7.695806	5.284189	0.542390363	3.47718048142e-15	6.71425705401e-15	-
YKL072W	3.777501	6.929351	-0.875288099	3.61722259022e-15	6.98249932839e-15	-
YDR350C	44.154736	58.13546	-0.396850222	3.70827264798e-15	7.15603251435e-15	-
YER170W	33.95245	25.804903	0.395870475	4.21402646327e-15	8.12948299688e-15	-
YPR034W	37.705101	51.993473	-0.463570812	4.217433485e-15	8.13352813758e-15	-
YJL164C	89.52697	84.308601	0.08664254	4.40710289388e-15	8.49667539292e-15	-
YFR042W	31.550797	23.003021	0.455853106	4.42651044926e-15	8.53144267898e-15	-
YHR063C	17.1262	26.920179	-0.652482924	4.51959502575e-15	8.70814584726e-15	-
YGR178C	117.80883	145.118744	-0.300786199	4.85439296467e-15	9.35031694839e-15	-
YLR225C	48.527809	43.028332	0.17352481	4.93039845013e-15	9.49376972034e-15	-
YMR220W	44.474545	60.651333	-0.447559507	5.21391971707e-15	1.00365933654e-14	-
YJL053W	86.440529	80.861023	0.0962634455051	5.4066858283e-15	1.04044344768e-14	-
YEL008W	33.594666	22.101881	0.60406303	5.66925806334e-15	1.09063384125e-14	-
YIL007C	88.794037	121.336266	-0.45047612	5.72377548688e-15	1.10078061408e-14	-
YKL040C	110.924385	146.589432	-0.402204545	5.77582593709e-15	1.1104468076e-14	-
YMR114C	359.519501	367.847321	-0.033037057	5.93742209208e-15	1.14116149683e-14	-
YEL062W	10.789421	16.874367	-0.645215937	6.13385263864e-15	1.17855020677e-14	-
YAL064W-B	7.521506	2.109095	1.834397476	6.17016547289e-15	1.18516049677e-14	up
YGL155W	26.862877	39.540173	-0.557705358	6.43000440677e-15	1.23468813354e-14	-

YHR065C	9.219838	15.33098	-0.733636614	6.43381313362e-15	1.23503747475e-14	-
YGR274C	25.937447	34.340076	-0.404856749	6.57542423993e-15	1.2618310257e-14	-
YDL060W	10.260201	15.572202	-0.60191397	7.08518486896e-15	1.35923447672e-14	-
YLR121C	76.444695	98.587349	-0.366986141	7.16578499945e-15	1.37427226681e-14	-
YNR054C	13.881026	23.269192	-0.745306908	7.99818358056e-15	1.53343803793e-14	-
YER045C	18.658834	27.821297	-0.576330844	8.4738086249e-15	1.6241248516e-14	-
YKR044W	15.66768	24.289412	-0.632535998	8.73159774618e-15	1.67301724717e-14	-
YOR343W-B	5.817973	8.529743	-0.551985673	9.44958279351e-15	1.8100280772e-14	-
YJL077C	39.278076	27.374884	0.520871411	9.64338813742e-15	1.84658092403e-14	-
YPL208W	9.879816	7.085527	0.479609013	1.01229711643e-14	1.93781699006e-14	-
YHR149C	4.306745	2.577398	0.740682571	1.1541256808e-14	2.20863508829e-14	-
YJL102W	64.244339	62.547962	0.0386064565318	1.21415057401e-14	2.32278790862e-14	-
YOL159C	47.30761	36.666935	0.367592601	1.2328963912e-14	2.35792384367e-14	-
YJR127C	19.971235	18.429897	0.115875542	1.30513049636e-14	2.49530339433e-14	-
YHR129C	11.533058	7.736115	0.576093949	1.38310696431e-14	2.64357390223e-14	-
YFL052W	27.546593	23.134132	0.251850926	1.44481040699e-14	2.76065958959e-14	-
YOR160W	7.817342	11.926327	-0.609399738	1.50613266779e-14	2.8769451082e-14	-
YBR236C	56.763245	75.67585	-0.414875908	1.58250299169e-14	3.02189436249e-14	-
YDR540C	18.443497	11.304812	0.706175225	1.60317336846e-14	3.06042443771e-14	-
YGL068W	106.603333	144.403549	-0.437853654	1.61832213044e-14	3.08839341708e-14	-
YMR316W	431.8974	444.262817	-0.040724767	1.65670137283e-14	3.16066445069e-14	-
YGL171W	10.836141	17.089964	-0.657298287	1.65761270159e-14	3.16143153655e-14	-

YOR150W	94.57769	81.719025	0.210827914	1.67957393417e-14	3.20233261158e-14	-
YBR151W	189.202225	185.960175	0.024935366	1.71890633104e-14	3.27631885266e-14	-
YDR499W	10.79307	8.382792	0.364602548	1.73441335015e-14	3.30486128844e-14	-
YGL061C	12.99524	7.86882	0.723764066	1.82813609483e-14	3.4823776854e-14	-
YER182W	228.506668	223.554169	0.0316118142934	1.83023732531e-14	3.48531083299e-14	-
YDL230W	25.866505	20.356153	0.345620195	1.87310311777e-14	3.56584610705e-14	-
YDL063C	4.947066	8.858764	-0.840532277	1.97042083508e-14	3.74996092708e-14	-
YDL018C	39.614952	31.391985	0.335648797	2.00151491122e-14	3.8079695277e-14	-
YIL070C	77.942741	105.467064	-0.436305962	2.01541546223e-14	3.83324117326e-14	-
YDR116C	58.321552	80.658279	-0.467793512	2.04484201438e-14	3.88801813943e-14	-
YJL160C	13.416011	8.664737	0.630727912	2.11226519971e-14	4.0149854133e-14	-
YDR103W	12.120586	17.543135	-0.533447133	2.16708379855e-14	4.11792354497e-14	-
YER171W	6.378373	10.38082	-0.702660037	2.41688549464e-14	4.59119496656e-14	-
YPR181C	134.153183	135.998535	-0.019709825	2.48637798716e-14	4.72176033781e-14	-
YHR043C	72.268608	63.686924	0.182371913	2.63754430933e-14	5.00730124535e-14	-
YOL108C	220.242172	286.312531	-0.378500071	2.68236293608e-14	5.09083127703e-14	-
YJR102C	34.601315	26.172474	0.402776563	2.72821052429e-14	5.17626251064e-14	-
YKL218C	48.896835	67.844589	-0.472492672	2.88693321363e-14	5.47573522464e-14	-
YER034W	33.858047	53.011791	-0.646814525	2.93530382054e-14	5.56578073242e-14	-
YHR166C	15.072556	22.244862	-0.561548059	2.94601424758e-14	5.58438364854e-14	-
YJR012C	40.752014	32.027031	0.347581298	3.05542669936e-14	5.79001494188e-14	-
YKR029C	20.908003	29.049809	-0.474473407	3.22737313055e-14	6.11398608314e-14	-

YDR257C	15.262421	11.740504	0.378489485	3.5629979101e-14	6.7477397882 5e-14	-
YML031W	10.243955	15.843557	-0.629123447	3.7630017278 9e-14	7.1243411792 5e-14	-
YJL038C	8.990156	17.724508	-0.979327526	3.8039656236 1e-14	7.1997007900 5e-14	-
YNL041C	14.511063	20.715631	-0.513566558	3.9727360463 1e-14	7.5168379687 6e-14	-
YDL157C	44.478188	31.615856	0.492449734	3.9999447655 8e-14	7.5660137430 7e-14	-
YKR073C	15.848452	32.474815	-1.034979375	4.0561596022 5e-14	7.6700087757 4e-14	down
YBR216C	25.734386	35.351849	-0.458088312	4.1057622976e-14	7.7614410303e-14	-
YGR041W	12.664075	9.593169	0.400662327	4.2367375445 8e-14	8.0065956398e-14	-
YFL044C	144.61911	139.329056	0.0537620506773	4.2832904065 6e-14	8.0921079865 9e-14	-
YDR314C	20.577719	17.648079	0.221571917	4.3268251735e-14	8.1718681707e-14	-
YGL241W	5.305143	3.784642	0.487234801	4.6161957403 9e-14	8.7157369697e-14	-
YLR358C	4.70513	11.6612	-1.309409768	4.6587065164 8e-14	8.7933262555e-14	down
YIR016W	139.498718	132.730713	0.0717496243493	4.9316851139 9e-14	9.3057450418 4e-14	-
YPL211W	17.160757	30.382921	-0.824147382	5.0055967564 9e-14	9.4423411316 6e-14	-
YGL125W	23.021072	32.369934	-0.491699408	5.2124602709 6e-14	9.8295727102 4e-14	-
YNL124W	25.116192	35.766613	-0.509995759	5.4089597081e-14	1.0197030631e-13	-
YIR004W	132.648132	166.128723	-0.324697172	6.0175366039 7e-14	1.1340882585 7e-13	-
YCR023C	28.297806	24.938923	0.182291039	6.0625557867 8e-14	1.1422259886e-13	-
YGR029W	84.890572	117.361404	-0.467281794	6.1420890481 6e-14	1.1568594906 2e-13	-
YLR018C	32.772045	47.961411	-0.549408402	6.4667338949 4e-14	1.2176367612 9e-13	-
YOR137C	11.164633	17.128704	-0.61748017	6.5041301068 4e-14	1.2243068436 4e-13	-
YGR028W	199.411896	246.576736	-0.306285214	6.5518928678 9e-14	1.2329236412 2e-13	-

YJL060W	15.180374	23.392796	-0.623856974	6.69411177335e-14	1.25930442088e-13	-
YOR067C	7.978613	13.072098	-0.712280829	7.81396073936e-14	1.46952645471e-13	-
YML041C	34.41169	50.41164	-0.550858143	7.87733874399e-14	1.48099693891e-13	-
YBL048W	31.602486	19.682819	0.683101192	8.04862554354e-14	1.51274197319e-13	-
YPR014C	48.678928	34.84729	0.482250932	8.11540252558e-14	1.52483107987e-13	-
YHR003C	34.414547	29.747215	0.210263913	8.11895835574e-14	1.52503762398e-13	-
YLR032W	4.594224	3.296043	0.479086136	8.31684646414e-14	1.56173571837e-13	-
YPL095C	24.12221	34.742428	-0.526336493	9.0616473967e-14	1.70107974112e-13	-
YBR192W	13.806893	22.121975	-0.680091489	9.34274965357e-14	1.75331891927e-13	-
YJL013C	23.32235	19.595415	0.251197037	9.36666885948e-14	1.75727652704e-13	-
YGL053W	225.354248	283.066376	-0.328945744	9.58349859604e-14	1.7974126672e-13	-
YLR268W	50.223866	72.480652	-0.529222848	9.8036049024e-14	1.8381389077e-13	-
YER037W	93.415054	121.969398	-0.384792256	9.94572970231e-14	1.86422373164e-13	-
YLL021W	10.706328	9.340418	0.196904737	1.02410358851e-13	1.91899640128e-13	-
YHR024C	22.244831	32.107624	-0.52944577	1.02615305003e-13	1.92225652823e-13	-
YPR049C	20.172457	26.839277	-0.411958994	1.05195658792e-13	1.96999894352e-13	-
YGR104C	21.028723	15.799756	0.412458964	1.12085294948e-13	2.0983881515e-13	-
YBL028C	30.552256	53.249252	-0.801482346	1.19487376157e-13	2.23629071807e-13	-
YDR470C	24.467022	34.729755	-0.505333719	1.22910570755e-13	2.2996649284e-13	-
YHR075C	23.372747	34.313042	-0.553929729	1.23838198718e-13	2.31632280096e-13	-
YCR052W	18.350483	27.112448	-0.563137344	1.26534886955e-13	2.36604993439e-13	-
YPR122W	9.527147	13.624616	-0.516099415	1.30799101103e-13	2.44504914077e-13	-

YCR035C	20.572561	30.762859	-0.580468188	1.36428030065e-13	2.54950394535e-13	-
YOR083W	33.557236	48.832584	-0.54122023	1.49950530358e-13	2.80136290239e-13	-
YBR211C	28.19776	41.441898	-0.555509518	1.54221761359e-13	2.88029089806e-13	-
YER107C	19.270273	29.363663	-0.60765494	1.81133730872e-13	3.38188932707e-13	-
YPL233W	38.879128	57.775749	-0.571468196	1.82107801667e-13	3.39905361619e-13	-
YBR033W	14.576928	12.491501	0.222739868	1.83861165509e-13	3.4307487691e-13	-
YGR168C	19.743671	15.336042	0.364464066	1.90273142942e-13	3.54932593566e-13	-
YNL166C	9.270483	15.38429	-0.730741452	1.93139674605e-13	3.60171553003e-13	-
YPR168W	35.245079	55.625862	-0.658333949	1.94875419596e-13	3.63299280737e-13	-
YOL012C	185.809631	245.677032	-0.402937706	1.95946184128e-13	3.65185803382e-13	-
YDL115C	138.398727	174.515762	-0.334526671	2.02587875706e-13	3.7745063997e-13	-
YLR265C	21.193913	16.37191	0.372427334	2.09100716764e-13	3.89468121714e-13	-
YLR333C	84.494553	124.021889	-0.553664524	2.1414577184e-13	3.98745336407e-13	-
YGL131C	17.044645	22.618143	-0.408161934	2.15703797874e-13	4.01525990165e-13	-
YHR178W	10.043434	15.143679	-0.592463103	2.2884336741e-13	4.25857201703e-13	-
YMR094W	4.449628	2.3718	0.907702367	2.45084685991e-13	4.5594418059e-13	-
YCR086W	10.8348	5.666356	0.935179373	2.51625187024e-13	4.6797158809e-13	-
YHR167W	4.528043	10.044035	-1.149379397	2.61733715078e-13	4.86625607428e-13	down
YPR116W	15.545951	25.560356	-0.717369056	2.70233570214e-13	5.02278444277e-13	-
YJL057C	52.699059	67.839897	-0.364356778	2.78360254704e-13	5.17228512782e-13	-
YCR102C	8.442837	5.224977	0.692303175	2.90852980806e-13	5.40279863807e-13	-
YGL188C-A	13.011704	35.978283	-1.46731643	3.00705445242e-13	5.58414419402e-13	down

YLR285W	38.286003	55.501907	-0.535720289	3.04725324362e-13	5.65710171543e-13	-
YGL203C	16.015062	22.777685	-0.508191744	3.13784980015e-13	5.82354904614e-13	-
YMR022W	216.082962	277.723602	-0.362064467	3.13822939178e-13	5.82251287035e-13	-
YKR050W	20.3284	27.624256	-0.442438941	3.21962465195e-13	5.97174479812e-13	-
YHL015W	658.122864	807.243225	-0.294646484	3.65693692264e-13	6.78084361283e-13	-
YPL227C	11.560571	7.695354	0.587153057	3.67069257146e-13	6.8043175526e-13	-
YML005W	21.433514	31.080761	-0.53615343	3.68160147424e-13	6.82250207526e-13	-
YKL013C	411.583771	411.181	0.00141249550591	3.684046202e-13	6.82499517219e-13	-
YHR041C	15.802857	10.053564	0.652478378	3.79580027925e-13	7.02993082744e-13	-
YOR073W	2.867189	1.378797	1.056226943	3.85407864203e-13	7.13573522509e-13	up
YKL017C	17.22625	24.45833	-0.505717227	4.10371034657e-13	7.59565707558e-13	-
YDR306C	127.080452	125.897728	0.013489879	4.17506172215e-13	7.72541972313e-13	-
YJR118C	35.367146	27.445681	0.365830667	4.26456298532e-13	7.88867908607e-13	-
YMR258C	20.397396	29.087738	-0.512026125	4.3701847726e-13	8.08165238853e-13	-
YNL030W	978.016479	1186.714844	-0.279042632	4.52100896005e-13	8.35807731506e-13	-
YOR057W	44.598751	60.664272	-0.443843789	4.58064193226e-13	8.46580086797e-13	-
YDL081C	680.375977	681.08551	-0.001503737	4.81324389891e-13	8.89304110845e-13	-
YJL146W	37.470951	50.978504	-0.444116444	4.83888696338e-13	8.93775967529e-13	-
YMR298W	174.586121	229.30246	-0.393312958	4.86733322826e-13	8.98762780519e-13	-
YOR390W	3.558239	7.582747	-1.091557171	5.25288758759e-13	9.69667741415e-13	down
YLR439W	82.833038	108.714722	-0.392269114	5.42079717108e-13	1.00036589887e-12	-
YBL004W	6.194413	8.530696	-0.461695878	5.61049509838e-13	1.03506548501e-12	-

YOR396W	14.560893	13.543139	0.104536674	5.79249778959e-13	1.06832520136e-12	-
YGR238C	14.739837	12.65184	0.220373354	5.85233299821e-13	1.07904019165e-12	-
YGL208W	144.198486	179.313736	-0.31442999	6.23263603399e-13	1.14881842337e-12	-
Q0158	1.07519	0.50499	1.090264901	6.37305680474e-13	1.17435252727e-12	up
YEL064C	15.898134	12.521128	0.344492906	6.51024783169e-13	1.19927651451e-12	-
YKL018W	9.819947	16.928419	-0.785660098	6.57449400631e-13	1.21075226316e-12	-
YLL001W	43.198032	55.756767	-0.368181322	6.61667633581e-13	1.21815915459e-12	-
YOR233W	3.96879	6.868944	-0.791389096	7.02679095408e-13	1.29327952099e-12	-
YNR034W	105.699135	100.673874	0.070274234	7.1234044593e-13	1.31067264029e-12	-
YLR185W	216.250961	291.472534	-0.430653398	7.48222072941e-13	1.37628522335e-12	-
YPL279C	3.463624	7.394444	-1.094159349	7.57531655476e-13	1.3929965987e-12	down
YJR011C	27.80953	21.724512	0.356255592	7.60593141128e-13	1.39821208769e-12	-
YML029W	16.323336	22.728111	-0.477541852	7.80931794195e-13	1.43517601491e-12	-
YAL014C	105.321388	137.858826	-0.388393195	8.31948837535e-13	1.52848132093e-12	-
YOR104W	49.049263	42.535122	0.205576867	8.62878368589e-13	1.58483695627e-12	-
YJR136C	9.999662	16.367392	-0.710873223	8.77344797998e-13	1.61093064359e-12	-
YLR418C	46.04863	62.329174	-0.436749359	8.79154577645e-13	1.61377635069e-12	-
YDL095W	20.652996	18.364149	0.169459037	8.83158563139e-13	1.62064686963e-12	-
YDR462W	210.024353	271.889709	-0.372464924	9.66634357842e-13	1.77330558318e-12	-
YNL246W	81.879555	74.876633	0.128987699	9.70410333848e-13	1.77970675112e-12	-
YER149C	21.136377	30.93469	-0.549497472	9.85603304444e-13	1.80703641878e-12	-
YGL236C	3.472468	6.393292	-0.880597588	9.93831528277e-13	1.82158433054e-12	-

YLR201C	186.317276	182.371094	0.0308843732711	1.07329648269e-12	1.96665423982e-12	-
YBR170C	52.989052	50.111294	0.0805585248206	1.07534872112e-12	1.96983324305e-12	-
YDR330W	142.068802	174.912872	-0.300046686	1.08919040193e-12	1.99460000448e-12	-
YOR059C	35.10149	48.099651	-0.454494154	1.15585789627e-12	2.11606187557e-12	-
YLR035C	9.629841	7.432528	0.373658985	1.21508539231e-12	2.22383552932e-12	-
YIL104C	5.702272	10.008714	-0.811647854	1.26857561295e-12	2.32104845423e-12	-
YOR299W	25.088446	22.62015	0.149414615	1.30107521521e-12	2.3798099399e-12	-
YGL162W	4.18757	8.985489	-1.101483712	1.31410858838e-12	2.40294141875e-12	down
YGR200C	14.444956	20.478399	-0.503537121	1.31437444597e-12	2.40271983527e-12	-
YMR059W	39.387394	62.367298	-0.66305579	1.33477000598e-12	2.43928530972e-12	-
YCR065W	4.50586	2.654966	0.763109103	1.38313433721e-12	2.52692700572e-12	-
YDR260C	88.904015	122.222267	-0.459186666	1.43315816124e-12	2.61754806266e-12	-
YPL014W	21.784822	17.512558	0.314933497	1.43816896597e-12	2.62592733551e-12	-
YGR015C	21.821798	17.07645	0.353761891	1.49705097764e-12	2.73263524528e-12	-
YNR014W	68.653419	94.707764	-0.464151131	1.54752187603e-12	2.82393174792e-12	-
YKR102W	2.394062	4.219333	-0.817554438	1.61254077268e-12	2.94171411013e-12	-
YPL146C	14.000663	10.712091	0.386255026	1.67436602414e-12	3.05360290184e-12	-
YBR299W	8.627423	6.26875	0.460751897	1.72724168876e-12	3.14910907601e-12	-
YCR073C	6.316635	5.094144	0.310316468	1.80381659088e-12	3.28775496071e-12	-
YGL143C	21.001284	30.724627	-0.548917961	1.86028253934e-12	3.3896783106e-12	-
YPR132W	243.910675	312.231903	-0.356265055	1.96663593166e-12	3.58241662668e-12	-
YGL095C	37.159691	49.402325	-0.410840435	2.01344694953e-12	3.66661151736e-12	-

RDN37-1	0.330498	0.824799	-1.319401042	2.13153790259e-12	3.88052413468e-12	down
YER060W	5.401421	9.447117	-0.806535126	2.22381971425e-12	4.04733884084e-12	-
YKL023W	121.923508	116.502274	0.0656182029406	2.27861525178e-12	4.14585096221e-12	-
YDL193W	34.353958	47.919743	-0.480143846	2.2866663597e-12	4.15928062145e-12	-
YDL160C-A	144.256927	203.421097	-0.495828714	2.38695405091e-12	4.34042494085e-12	-
YNR037C	327.295074	309.726288	0.0795980522001	2.39894756808e-12	4.36095651615e-12	-
YJL106W	8.883738	6.673443	0.412735569	2.47502214874e-12	4.49793252324e-12	-
YOR182C	309.65152	281.369446	0.138179854	2.48685550384e-12	4.51811500376e-12	-
YML079W	148.963562	140.992203	0.0793440935319	2.51110562945e-12	4.56083784306e-12	-
YOL065C	45.13887	40.500744	0.156421892	2.74870921992e-12	4.99092917146e-12	-
YMR100W	35.998421	47.641575	-0.404287482	2.89316565845e-12	5.2516878385e-12	-
YDL054C	41.998142	55.995289	-0.414979951	2.93800177733e-12	5.33151564854e-12	-
YPL241C	13.973792	9.449845	0.564361	2.95670325793e-12	5.36388481157e-12	-
YPL069C	8.060501	14.24678	-0.821694467	3.11405149588e-12	5.64768673282e-12	-
YNR015W	16.073685	24.581104	-0.612848998	3.13507897338e-12	5.68416187698e-12	-
YAL034C	413.708679	431.195709	-0.059727598	3.33300320192e-12	6.04125076717e-12	-
YDR495C	9.975992	8.331801	0.259831926	3.43145332225e-12	6.21788155999e-12	-
YPR079W	24.708424	35.665359	-0.529520503	3.55893695075e-12	6.44700338205e-12	-
YKL056C	287.572968	360.482513	-0.326001208	3.77727655381e-12	6.84052883491e-12	-
YGR207C	86.374428	79.77961	0.114584181	3.84544076078e-12	6.96194116154e-12	-
YBR140C	19.078135	18.963928	0.00866232557027	3.91020978309e-12	7.07713770653e-12	-
YDR354W	37.373127	51.493126	-0.462378576	3.91806777858e-12	7.08929314177e-12	-

YKL061W	379.996582	370.372467	0.0370095872951	4.14565019762e-12	7.49889173276e-12	-
YMR063W	24.504366	37.627701	-0.618756325	4.25787771671e-12	7.69965186872e-12	-
YLR161W	34.485828	56.551254	-0.71355541	4.28648706933e-12	7.74912979803e-12	-
YLR329W	3.663714	1.306599	1.487490446	4.53045554777e-12	8.18779273379e-12	up
YHL008C	48.522301	62.603096	-0.36758604	4.75947269124e-12	8.59918698114e-12	-
YGR099W	18.237951	15.758092	0.210850792	4.9795756992e-12	8.99424089049e-12	-
YBR133C	10.277815	8.370701	0.296113238	5.42233116282e-12	9.7911087431e-12	-
tH(GUG)G1	15.366854	0.761538	4.334762	5.42422173687e-12	9.79167448168e-12	up
YJL219W	7.280435	5.086032	0.517484114	5.51606107012e-12	9.95456602421e-12	-
YGL257C	25.956318	22.864567	0.182972148	5.60313154118e-12	1.01087592583e-11	-
YGR166W	10.266057	15.802393	-0.62226087	5.68571278163e-12	1.02547661093e-11	-
YPR124W	162.988403	200.723053	-0.300437002	5.74945257821e-12	1.03667155404e-11	-
YOL008W	29.616125	22.74123	0.381072602	5.95154802506e-12	1.07279936526e-11	-
YGR033C	164.247253	159.22113	0.0448374354792	5.98459986945e-12	1.0784440055e-11	-
YDL098C	13.575575	24.002371	-0.82216362	6.15554117037e-12	1.10892627933e-11	-
YBR023C	15.605244	14.118867	0.144406594	6.24360607614e-12	1.12446494113e-11	-
YJL148W	14.26678	24.038998	-0.752717006	6.48616561569e-12	1.16781079299e-11	-
YDR057W	24.372871	33.756271	-0.469879337	6.53518515809e-12	1.17629543234e-11	-
YOR118W	38.169746	35.178391	0.117740088	6.62082704013e-12	1.19136505116e-11	-
YMR157C	36.099442	52.058205	-0.52814903	6.70006243179e-12	1.20527347368e-11	-
YJR042W	23.285107	31.401176	-0.431411079	6.82710614148e-12	1.22777157956e-11	-
YER159C	286.496155	278.603333	0.0403032596718	6.94170632952e-12	1.24801948722e-11	-

YJL082W	22.738504	20.389082	0.15734052	7.35349719361e-12	1.32167083318e-11	-
YBL008W	4.929039	7.977243	-0.694583829	7.79282194842e-12	1.4002268786e-11	-
YDL192W	147.093445	191.340195	-0.379407017	7.90948967215e-12	1.42077870037e-11	-
YIL161W	20.720947	15.150768	0.451699013	7.96442986786e-12	1.43023374659e-11	-
YGL077C	14.09288	20.688799	-0.553883429	8.49265137468e-12	1.52464950069e-11	-
YNL162W	148.393463	201.695038	-0.442748052	9.02056432792e-12	1.61895528614e-11	-
YKL018C-A	62.285072	94.499474	-0.601419868	9.4782523355e-12	1.70060666182e-11	-
YPR120C	8.554482	5.866281	0.544234319	1.00598575105e-11	1.80443789151e-11	-
YKL157W	124.584663	149.502823	-0.26304625	1.01467668947e-11	1.81950112312e-11	-
YPL260W	215.404419	258.470276	-0.262950534	1.02299049412e-11	1.83387958056e-11	-
YKL024C	72.695488	64.42057	0.174344397	1.0359028308e-11	1.85649098545e-11	-
YLR344W	244.131027	313.823303	-0.362296823	1.04789591205e-11	1.87744237288e-11	-
YGL075C	7.3485	4.650393	0.66009715	1.05280253239e-11	1.88568901358e-11	-
YJL186W	36.082455	33.256733	0.11765105	1.11858709534e-11	2.002938762e-11	-
YDL198C	30.058258	43.344429	-0.528085176	1.14274455968e-11	2.04560502494e-11	-
YPL272C	5.909529	3.828217	0.626370539	1.17666358967e-11	2.10571564274e-11	-
YOR286W	92.342445	127.023178	-0.460025933	1.18032807831e-11	2.11166475796e-11	-
YMR158W	42.817638	64.202049	-0.584414129	1.28084659767e-11	2.29083713004e-11	-
YCL005W-A	113.583389	92.73877	0.292507366	1.32048053528e-11	2.36104353773e-11	-
YNR063W	3.697546	6.7163	-0.861098578	1.34416267311e-11	2.40269561609e-11	-
YDL105W	15.361841	11.894257	0.369085969	1.35500392398e-11	2.42137719058e-11	-
YNL267W	35.27615	44.752396	-0.34327181	1.35678960641e-11	2.42387046808e-11	-

YEL047C	34.298573	30.738632	0.158095594	1.3962800319e-11	2.49370150692e-11	-
YGL010W	81.317566	71.772087	0.180144161	1.45329811494e-11	2.59478708586e-11	-
YHR059W	47.519638	71.915924	-0.597787409	1.47453393287e-11	2.6319455593e-11	-
YNR012W	8.398858	13.486261	-0.683225342	1.48047187049e-11	2.64178481518e-11	-
YPL032C	7.327417	11.115994	-0.601260335	1.4902178454e-11	2.65841160467e-11	-
YDR515W	6.43311	4.101132	0.649494178	1.49649634218e-11	2.66884495613e-11	-
YNR041C	17.306053	26.03319	-0.589075378	1.54143238534e-11	2.74819421257e-11	-
YMR102C	6.1973	9.62367	-0.634947364	1.55807414363e-11	2.7770669778e-11	-
YDR437W	21.416119	36.542648	-0.77088412	1.55974224366e-11	2.77924220684e-11	-
YKL168C	32.330547	42.390785	-0.390852766	1.58272885281e-11	2.81939188472e-11	-
YER092W	101.361885	140.400101	-0.470028714	1.62096602379e-11	2.88667730226e-11	-
YKL082C	27.139921	37.883453	-0.481151314	1.62844468215e-11	2.89916391936e-11	-
YBR145W	40.493282	55.445965	-0.453399895	1.68691791709e-11	3.00240436619e-11	-
YCR032W	2.627174	2.001666	0.39231049	1.70872058916e-11	3.04033746562e-11	-
YLR363C	23.050449	17.122383	0.428911351	1.76721128361e-11	3.14350935491e-11	-
YKL087C	56.618938	78.418861	-0.469915999	1.77206264797e-11	3.1512360122e-11	-
YBL034C	3.673777	2.788393	0.397830146	1.88974308548e-11	3.35954326307e-11	-
YKL033W	3.877557	2.732646	0.504849411	1.98673843812e-11	3.53096828624e-11	-
YLR277C	10.512206	15.290956	-0.540613155	2.08286083566e-11	3.70074415221e-11	-
YNL159C	99.709061	128.598236	-0.367074333	2.11004762116e-11	3.74797586041e-11	-
YKR036C	15.164456	21.598795	-0.510257082	2.13246317932e-11	3.78670807129e-11	-
YOR154W	24.13051	33.020084	-0.452485383	2.14897877986e-11	3.8149443138e-11	-

YDR202C	95.201454	121.504196	-0.351950623	2.20897479515e-11	3.9203303397e-11	-
YBR253W	48.721344	74.032761	-0.603609903	2.27592257191e-11	4.03799009042e-11	-
YMR123W	45.345573	34.505978	0.394115386	2.34687996896e-11	4.1626945278e-11	-
YDR055W	315.765503	376.185425	-0.252590387	2.48985799451e-11	4.41503525561e-11	-
YPR036W	285.868652	296.520386	-0.05277887	2.54022664961e-11	4.50306311843e-11	-
YNL053W	32.313507	43.718124	-0.436094159	2.74050446444e-11	4.85670902519e-11	-
YBR276C	15.400823	13.416473	0.199001991	2.80172310288e-11	4.96378339688e-11	-
YDR524W-C	538.98645	728.401184	-0.434484263	2.90875140808e-11	5.15193402036e-11	-
YOL070C	39.224514	36.047123	0.121871453	2.97033824193e-11	5.25951506157e-11	-
YLR262C-A	147.219833	123.577835	0.252552036	3.01195701988e-11	5.33168781848e-11	-
YEL015W	59.94849	76.510437	-0.351933148	3.06579572689e-11	5.42544466149e-11	-
YOR081C	36.697327	47.423752	-0.369934826	3.1419608361e-11	5.55864715603e-11	-
YNL247W	16.607002	14.532556	0.192503185	3.20218805258e-11	5.66358502291e-11	-
YBR037C	81.039856	105.804291	-0.384694623	3.2115123897e-11	5.6784588195e-11	-
YDR378C	201.061554	269.509216	-0.422697365	3.26908014648e-11	5.77860180791e-11	-
YOL145C	15.433328	13.937868	0.147039297	3.28504461502e-11	5.80516850841e-11	-
YLR326W	44.761749	62.909187	-0.491004309	3.3986768792e-11	6.00426467446e-11	-
YDL029W	176.218964	177.558441	-0.010924757	3.47998379211e-11	6.14615629627e-11	-
YKR051W	28.450212	39.546162	-0.475098283	3.65997140944e-11	6.46220207902e-11	-
YMR301C	15.646771	13.404908	0.223103643	3.80967052117e-11	6.72460466176e-11	-
YJL024C	77.653717	69.436958	0.161351237	3.8906690812e-11	6.86562639456e-11	-
YGL204C	13.414186	6.35474	1.077854507	4.05274298038e-11	7.14959602791e-11	up

YBR272C	26.904436	37.031296	-0.460900977	4.25454561346e-11	7.50347135464e-11	-
YDR490C	16.245441	14.201693	0.193971982	4.4317751203e-11	7.81381992241e-11	-
YBL025W	42.491955	63.833969	-0.587134631	4.45869673108e-11	7.85905431758e-11	-
YBR176W	11.354908	7.810604	0.539809995	4.49451965975e-11	7.91994835303e-11	-
YDR209C	113.07476	101.525223	0.155438738	4.49602787743e-11	7.9203578499e-11	-
YML051W	28.53377	24.935402	0.194474913	4.7016515312e-11	8.28024190233e-11	-
YIL009C-A	44.206997	36.265842	0.28566339	4.79082016705e-11	8.43488700995e-11	-
YOL053W	47.655724	63.304981	-0.40966951	5.26104463149e-11	9.26015454275e-11	-
YOR387C	4.465302	1.657486	1.429761067	5.36131734902e-11	9.43397338513e-11	up
YER084W	108.105659	95.900856	0.172826448	5.55735782097e-11	9.77616246885e-11	-
YNR010W	161.994278	152.279816	0.0892181229936	5.66490694404e-11	9.9625332319e-11	-
YNL273W	1.704434	0.988567	0.785882083	5.79731103516e-11	1.01924969998e-10	-
YDL153C	26.322803	35.418034	-0.428171009	6.66732335309e-11	1.17187835153e-10	-
YGL063W	7.570817	12.947006	-0.774097611	6.68733192613e-11	1.17506245676e-10	-
YDL142C	71.369156	65.991112	0.113028983	6.69723545999e-11	1.17646965862e-10	-
YBL002W	244.957047	312.340393	-0.35059036	6.73190258202e-11	1.18222492869e-10	-
YCL052C	114.864059	143.062897	-0.316722112	6.73475078644e-11	1.18239063581e-10	-
YOR266W	20.212713	29.067841	-0.524160943	6.81224137108e-11	1.1956571793e-10	-
YOR066W	40.031551	37.784054	0.0833600107535	7.31790026829e-11	1.28404536081e-10	-
YJL058C	4.929746	3.147081	0.647499004	7.46851366147e-11	1.31010265076e-10	-
YLL066C	27.306387	26.337685	0.0521098960997	7.90222341511e-11	1.3857910441e-10	-
YLR098C	16.185217	22.717348	-0.489117718	8.0829205728e-11	1.41707909958e-10	-

YKL112W	29.264984	27.232668	0.103837159	8.1489157131e-11	1.42824587089e-10	-
YML032C	43.479618	40.307606	0.109287166	8.26917095899e-11	1.44891372603e-10	-
YCR020W-B	34.942898	22.483263	0.636147834	8.38986137137e-11	1.46964614541e-10	-
YLR398C	11.855939	16.022158	-0.434458545	8.42388892921e-11	1.47519047877e-10	-
YGR078C	48.398724	68.497337	-0.501078889	8.4773098687e-11	1.4841268941e-10	-
YDL064W	93.16729	126.325272	-0.439247852	8.50623672158e-11	1.4887712875e-10	-
YHR098C	18.819853	25.121689	-0.416678104	8.59048449093e-11	1.50309266403e-10	-
YER093C	9.282902	8.207522	0.177629176	8.80641880227e-11	1.5404409108e-10	-
YGL115W	58.793625	77.925514	-0.406436034	8.84618079165e-11	1.54696029168e-10	-
YML036W	13.641521	8.497161	0.682951705	8.84696537658e-11	1.5466618152e-10	-
YLR376C	10.123209	6.261243	0.69314569	9.42357610697e-11	1.64700339167e-10	-
YDR519W	67.249413	95.780083	-0.510204011	9.45544611656e-11	1.65210834482e-10	-
YHR007C-A	16.220116	7.092063	1.19350688	9.87592178741e-11	1.72509067125e-10	up
YKR078W	14.878534	12.428967	0.259525987	1.0207904746e-10	1.78257869658e-10	-
YIR035C	46.321758	64.112625	-0.468918472	1.03562647681e-10	1.80797783128e-10	-
YPL153C	2.769786	1.678684	0.722443836	1.06724103905e-10	1.86264615418e-10	-
YLR099W-A	180.947891	243.389587	-0.427693153	1.07476445289e-10	1.87524950072e-10	-
YAR071W	20.606359	17.493439	0.23627568	1.10563690417e-10	1.92857372888e-10	-
YEL032W	13.165855	18.069893	-0.456786749	1.14715104933e-10	2.00042520063e-10	-
YDL225W	26.12529	23.373655	0.160563702	1.1616825013e-10	2.02519656503e-10	-
YGL237C	99.401215	128.27533	-0.367908346	1.19833618358e-10	2.08850955297e-10	-
YAL016W	208.480072	248.27359	-0.252021316	1.20533561497e-10	2.10011885987e-10	-

YJL168C	6.038593	9.468364	-0.648902731	1.2297201623 6e-10	2.1420041436 4e-10	-
YGL206 C	26.71806	26.33574 5	0.0207929856 204	1.2443700655 4e-10	2.1669142684 1e-10	-
YMR231 W	5.152843	7.862864	-0.609686265	1.2676891416 e-10	2.2069024652 3e-10	-
YLR342 W	33.81959 2	34.03326	-0.009086097	1.2698023875 6e-10	2.2099616546 e-10	-
YBR153 W	33.64909	48.42662 4	-0.525232941	1.3208174516 1e-10	2.2981039068 3e-10	-
YGR025 W	22.23128 7	13.46437 4	0.723444314	1.3231992614 1e-10	2.3016029741 7e-10	-
YBR228 W	28.21626 7	40.37642 7	-0.516986116	1.3272816277 4e-10	2.3080572395 1e-10	-
YPL059 W	259.4252 62	326.2182 92	-0.330518709	1.3991866031 e-10	2.4324140106 5e-10	-
YMR179 W	2.529491	1.440073	0.81270516	1.4081325216 5e-10	2.4472807095 1e-10	-
YJL199C	56.83749 8	44.91965 1	0.339496329	1.4311007657 1e-10	2.4865025338 7e-10	-
YGL220 W	137.4093 32	124.9787 67	0.136796974	1.4371481019 7e-10	2.4963109728 7e-10	-
snR37	39.38684 5	29.89924	0.397605044	1.4584699895 8e-10	2.5326382364 5e-10	-
YMR167 W	17.42774 2	23.77816 4	-0.448251663	1.4715965193 9e-10	2.5547178949 7e-10	-
YPR048 W	7.260057	11.33554 4	-0.642800848	1.4735398999 6e-10	2.5573764883 9e-10	-
YDR493 W	35.06670 8	55.12323 4	-0.652558532	1.4865487243 7e-10	2.5792326665 5e-10	-
YGL012 W	25.19561 2	22.05460 7	0.192092447	1.4944044756 9e-10	2.5921383026 1e-10	-
YNR022 C	100.5846 1	136.5774 84	-0.441310081	1.5264131603 1e-10	2.6469198042 5e-10	-
YER022 W	22.36759 2	20.18511	0.148118501	1.5855942292 e-10	2.7487765917 e-10	-
YEL051 W	248.8173 83	250.6632 84	-0.010663413	1.6698446604 1e-10	2.8940244700 8e-10	-
YGL243 W	14.47237	21.76848 2	-0.588939609	1.6773510477 4e-10	2.9062225242 5e-10	-
YOR080 W	6.13446	4.54458	0.432789383	1.6876553972 9e-10	2.9232602417 3e-10	-
YKL114 C	34.65916 1	30.62604 3	0.178477754	1.7030486461 4e-10	2.9491006960 1e-10	-

YHR112C	50.291325	46.442108	0.114876106	1.71513441204e-10	2.96920090071e-10	-
YML018C	8.628094	14.11982	-0.710607898	1.75148660413e-10	3.03128766056e-10	-
YHR040W	25.132549	35.646976	-0.504222695	1.76935264599e-10	3.06135485682e-10	-
YER168C	11.464891	9.095324	0.334025702	1.78998343005e-10	3.09618755469e-10	-
YDR440W	6.952516	5.025606	0.468237589	1.82215513496e-10	3.1509579604e-10	-
YIR032C	47.870564	40.597397	0.237751575	1.84220350416e-10	3.18473944691e-10	-
YPL172C	6.762125	11.21866	-0.730351775	1.92138111784e-10	3.32069431501e-10	-
YPR003C	25.155693	33.240246	-0.402046125	1.93400507017e-10	3.34158181898e-10	-
YDL061C	300.98822	401.271057	-0.414870076	1.95909897143e-10	3.38399733295e-10	-
YPL224C	32.76683	43.942379	-0.423376871	2.08379535733e-10	3.59838708714e-10	-
YBR301W	5.844108	13.912524	-1.251329432	2.11743842812e-10	3.65546656334e-10	down
YMR237W	36.210804	34.455524	0.0716849116602	2.19670721602e-10	3.79125893718e-10	-
YLR096W	6.992369	10.063173	-0.525232045	2.19743477429e-10	3.79146055554e-10	-
YMR012W	29.50264	28.837513	0.0328973083999	2.24153160711e-10	3.86647074658e-10	-
YHL012W	1.594254	3.66779	-1.202029539	2.43182196206e-10	4.19354187234e-10	down
YDR091C	10.768969	15.885233	-0.560806116	2.44747243772e-10	4.21935820421e-10	-
YDR525W-A	429.065826	416.750732	0.042014265	2.499939831e-10	4.30861367875e-10	-
YDR332W	20.586044	27.858892	-0.436471265	2.59101996071e-10	4.46434968529e-10	-
YBR239C	5.1644	8.756149	-0.761695758	2.65545648877e-10	4.5741048508e-10	-
YCL020W	22.977804	32.185574	-0.486173273	2.75463615925e-10	4.74362864816e-10	-
YOR002W	34.015079	31.399025	0.115454678	2.83553503303e-10	4.88158665697e-10	-
YGR123C	5.434659	9.178387	-0.756051123	2.96601500167e-10	5.1048020877e-10	-

YMR158C-A	12.57773	3.174925	1.986077154	2.97352936918e-10	5.11631660861e-10	up
YPL071C	92.334572	124.680199	-0.433289533	3.02220125417e-10	5.19862160873e-10	-
YER155C	14.651495	18.616529	-0.345536231	3.03602298569e-10	5.22095033107e-10	-
YLL017W	27.371679	46.04158	-0.75025341	3.06002796744e-10	5.26077364216e-10	-
YNL148C	26.401178	21.331547	0.307613707	3.06571919418e-10	5.26909877007e-10	-
YDL156W	4.990146	3.217209	0.633272365	3.1142061773e-10	5.35095265671e-10	-
YOL100W	34.408928	43.283237	-0.331025451	3.25202247907e-10	5.58620795519e-10	-
YMR137C	17.642471	15.473739	0.189230794	3.31231598397e-10	5.68820404661e-10	-
YFL060C	45.882942	64.132957	-0.483108028	3.3261892992e-10	5.71044888535e-10	-
YIR024C	53.637444	73.930183	-0.462922995	3.33450821255e-10	5.72314818455e-10	-
YPR045C	42.442196	55.728111	-0.392905947	3.36628187433e-10	5.77608564838e-10	-
YKL006C-A	62.916298	50.009285	0.331237806	3.54964588082e-10	6.08903056869e-10	-
YGR097W	28.80335	36.491741	-0.341333367	3.55641372355e-10	6.09895480548e-10	-
YNL158W	48.819962	68.558105	-0.489856061	3.57708054441e-10	6.13270257379e-10	-
YHL042W	19.157806	12.775399	0.584564	3.74789012889e-10	6.42377192715e-10	-
YGL262W	14.267164	24.523006	-0.781437247	3.75988065264e-10	6.4425446016e-10	-
YGR119C	48.041336	61.819832	-0.363793462	3.76689132081e-10	6.45277630231e-10	-
YLR397C	8.510154	12.491733	-0.553716494	3.87407140136e-10	6.63454765783e-10	-
YML053C	43.357407	36.852421	0.234519079	3.89023566018e-10	6.6603924375e-10	-
YOL124C	4.000953	7.418571	-0.890797634	3.91411876455e-10	6.69943459894e-10	-
YDR534C	3.376842	1.908644	0.823126642	3.95139038584e-10	6.76136480576e-10	-
YLL007C	7.232263	5.497595	0.395646513	4.0521319868e-10	6.93183669717e-10	-

YNL236W	6.578652	9.702118	-0.560507728	4.08038589714e-10	6.97824673538e-10	-
YPR169W	46.838646	60.770359	-0.375668443	4.18606942398e-10	7.15701431674e-10	-
YDL052C	20.337753	30.157633	-0.568362906	4.18676148322e-10	7.15622667616e-10	-
YLR325C	613.540222	610.223694	0.00781972756373	4.23038323608e-10	7.22879689777e-10	-
YLR310C	22.488314	28.33297	-0.333306337	4.26706338109e-10	7.2894687589e-10	-
YDR434W	37.107361	48.703339	-0.392315281	4.29136978015e-10	7.32897485423e-10	-
YLR128W	50.323246	45.034512	0.160193955	4.29614526964e-10	7.33511271559e-10	-
YNL154C	36.846077	48.287838	-0.390148847	4.37200668551e-10	7.46258386132e-10	-
YGR059W	5.758512	9.584893	-0.735066259	4.49976463317e-10	7.67854283747e-10	-
YPR140W	82.244301	104.433044	-0.344590654	4.55373897763e-10	7.76851101213e-10	-
YNL133C	81.905891	73.722664	0.151859016	4.62344613691e-10	7.88526198294e-10	-
YOL073C	101.793022	128.909851	-0.340723849	4.63865946897e-10	7.90903542526e-10	-
YKR048C	319.430634	379.444061	-0.248384537	5.05171804503e-10	8.61094608005e-10	-
YDR198C	11.74953	9.21648	0.350315288	5.09510674688e-10	8.68252063811e-10	-
YNR076W	2.452854	8.005416	-1.70651501	5.12247452168e-10	8.72676230257e-10	down
YDR375C	32.599804	43.787663	-0.425661163	5.26715012813e-10	8.97077311259e-10	-
YMR004W	65.068703	82.285721	-0.338678305	5.33756522458e-10	9.08820760127e-10	-
YDR206W	21.092403	27.790998	-0.397894177	5.54505288583e-10	9.43890548814e-10	-
YOR117W	230.172241	237.239853	-0.043632529	5.55427206936e-10	9.45200685488e-10	-
YKL034W	30.178238	39.128029	-0.374693865	5.58998765205e-10	9.51017904739e-10	-
YDL057W	50.189789	45.950798	0.127303965	5.69480719935e-10	9.68585290235e-10	-
YJR107W	19.422922	28.613337	-0.558927503	5.75025105185e-10	9.77747426182e-10	-

YIL095W	7.965011	11.707067	-0.55563142	5.87908740992e-10	9.99380466616e-10	-
YEL056W	51.974472	48.695187	0.0940240127268	6.03265180149e-10	1.02520400722e-09	-
YCL049C	20.793133	16.80056	0.30759783	6.11974467789e-10	1.03972016859e-09	-
YLR378C	82.649559	103.387367	-0.322980885	6.16734366669e-10	1.04752036889e-09	-
YLR019W	76.959572	97.770065	-0.345292036	6.33052821236e-10	1.07494308376e-09	-
YOR047C	7.092437	11.618741	-0.712100412	6.39963397105e-10	1.08638030332e-09	-
YCR072C	3.976701	7.069576	-0.830051599	6.50451078369e-10	1.10388198319e-09	-
YKL165C	4.310414	3.154386	0.450467229	6.88123205117e-10	1.16749627149e-09	-
YNL254C	10.140979	7.468141	0.441375864	7.14818568331e-10	1.21245728749e-09	-
YGR080W	50.026325	45.912731	0.123793224	7.32429655784e-10	1.24198942997e-09	-
YPR193C	78.412041	69.614868	0.17167975	7.46112241547e-10	1.26484565689e-09	-
YGL089C	10.210866	4.95533	1.043052187	7.54032951613e-10	1.27792425979e-09	up
YNR061C	11.173069	19.132215	-0.775978392	7.69082044945e-10	1.30307350847e-09	-
YNL080C	34.602379	47.082932	-0.444332934	7.70948582445e-10	1.30587961796e-09	-
YGL263W	6.780444	4.423653	0.616141525	8.01178845014e-10	1.35671529456e-09	-
YDR383C	27.140429	39.831299	-0.553459005	8.04951194187e-10	1.36273166444e-09	-
YJR080C	83.426674	81.147324	0.0399652104114	8.18918811582e-10	1.38599999517e-09	-
YDR034C-A	14.242558	5.348199	1.413083229	8.26670410969e-10	1.39873805159e-09	up
YKL140W	27.135565	24.640045	0.139180057	9.3762894038e-10	1.58604917217e-09	-
YOR343C	20.035561	12.347294	0.698368007	9.41287349308e-10	1.59180383125e-09	-
YGL207W	74.208855	89.81707	-0.275398312	9.50187934725e-10	1.60641794629e-09	-
YLR006C	26.287081	34.548538	-0.394270712	9.57340819754e-10	1.61807019032e-09	-

YGL192W	0.112812	0.791543	-2.810747188	9.5916138773 8e-10	1.6207060139 e-09	down
YKL002W	185.330353	230.529999	-0.314855319	9.6531298661 9e-10	1.6306566043 3e-09	-
YLR328W	23.493433	32.911018	-0.486313109	9.7308294049 1e-10	1.6433348461 8e-09	-
YKR085C	38.193153	54.960403	-0.525078558	1.0058128126 4e-09	1.6981468427 8e-09	-
YBR157C	323.443634	390.281128	-0.270999385	1.0170444697 e-09	1.7166427590 8e-09	-
YIL013C	4.823382	3.947119	0.289245057	1.0621016866 9e-09	1.7922063797 2e-09	-
YER098W	24.223896	31.86375	-0.395485149	1.1828357295 e-09	1.9953924480 2e-09	-
YMR321C	9.197476	19.478832	-1.082597261	1.1943209826 3e-09	2.0142202282 4e-09	down
YJR110W	9.148816	13.419497	-0.552673643	1.2229043935 6e-09	2.0618659628 5e-09	-
YDR267C	32.502525	44.855595	-0.464736149	1.2357116815 8e-09	2.0828938689 3e-09	-
YGR234W	14.58375	11.622706	0.327415739	1.2595414121 3e-09	2.1224845511 7e-09	-
YKR103W	1.230072	0.663076	0.891496621	1.3055395142 2e-09	2.1994000825 7e-09	-
YBR282W	111.020363	147.382065	-0.408736657	1.3122803465 5e-09	2.2101563731 3e-09	-
YFR004W	458.260162	478.896484	-0.06354697	1.3274951771 7e-09	2.2351749552 1e-09	-
YBL068W	27.108587	38.169537	-0.49367177	1.341356192e-09	2.2579010954 2e-09	-
YGR135W	259.303894	263.244324	-0.021758553	1.4577832000 2e-09	2.4532171606 8e-09	-
YDR056C	147.483368	187.017654	-0.342622195	1.4590862681 5e-09	2.4547445942 2e-09	-
YKR052C	31.285109	43.637596	-0.480095495	1.5328098979 1e-09	2.5780774441 1e-09	-
YGR098C	2.508098	1.86498	0.427433561	1.5897605859 1e-09	2.6731402268 e-09	-
YOR307C	45.103504	58.75465	-0.381463517	1.6079829091 e-09	2.7030484429 1e-09	-
YKL116C	13.782788	11.401517	0.273641956	1.6405163859 3e-09	2.7569912625 6e-09	-
YFL023W	10.039926	14.275003	-0.507742413	1.6729916542 5e-09	2.8108070878 5e-09	-

YFR023W	0.929109	2.305794	-1.311343864	1.68146069376e-09	2.82427164147e-09	down
YKR088C	36.738495	49.891209	-0.441493103	1.73482337025e-09	2.91311427712e-09	-
YNL260C	41.192776	58.400505	-0.503589491	1.76026883689e-09	2.95504297983e-09	-
YGR023W	68.809135	86.029518	-0.322231647	1.76408992084e-09	2.9606569961e-09	-
YNL284C	110.903122	108.771919	0.0279938262891	1.76646329537e-09	2.96383895613e-09	-
YCR090C	21.700947	16.044649	0.435665772	1.78291031276e-09	2.99062610689e-09	-
YLL067C	22.204277	21.343369	0.057049676	1.87564831355e-09	3.14533353066e-09	-
YDR393W	22.764051	31.444891	-0.466068318	1.90411443198e-09	3.19220696563e-09	-
YPL138C	76.017838	97.035667	-0.352177136	1.90617697605e-09	3.19480201602e-09	-
YDR374W-A	98.387115	84.188736	0.224842168	1.95940829088e-09	3.28313270439e-09	-
YOR243C	10.251414	14.822132	-0.531930061	1.96392747658e-09	3.28981699261e-09	-
YLR243W	40.012287	35.232349	0.183542427	2.03584548864e-09	3.40936843632e-09	-
YBR227C	21.151674	29.043468	-0.457441885	2.05841383166e-09	3.44623329744e-09	-
YJR149W	23.729769	33.030342	-0.477093851	2.10431084731e-09	3.52212503103e-09	-
YJR027W	4.983407	4.258039	0.226943242	2.11345293038e-09	3.53647325924e-09	-
YPR127W	74.081383	70.994362	0.0614065741394	2.13604533898e-09	3.57331432616e-09	-
YER188W	10.17635	6.620926	0.620115285	2.16406242581e-09	3.61920785007e-09	-
YLR410W	38.031662	47.14547	-0.309918169	2.45955583743e-09	4.11228727141e-09	-
YKR027W	6.116681	4.714406	0.375673031	2.49757563683e-09	4.17473062827e-09	-
YOR359W	4.976783	8.329569	-0.743028364	2.54654112916e-09	4.25543131355e-09	-
YPR134W	43.482292	38.676796	0.168959702	2.64109970283e-09	4.41225698471e-09	-
YGL128C	53.612476	71.349434	-0.41233322	2.66617666816e-09	4.45295258432e-09	-

YCR038C	4.082385	6.812332	-0.738736501	2.7367279603e-09	4.5695554538 8e-09	-
YMR268C	19.589108	16.783916	0.222972343	2.94084719411e-09	4.90905603147e-09	-
YCL026C-B	6.998186	3.690167	0.923294902	2.98491323385e-09	4.98127455799e-09	-
YML058W-A	16.392687	7.857643	1.060883825	3.02266999566e-09	5.04292806585e-09	up
YGR163W	59.083256	76.810524	-0.378554658	3.02447501029e-09	5.04458378932e-09	-
YNL263C	40.357513	54.533112	-0.434295215	3.06719746003e-09	5.11446731986e-09	-
YFR003C	176.59584	170.401688	0.0515117313977	3.10925304566e-09	5.18320164002e-09	-
YMR261C	40.656178	40.402744	0.0090213182546	3.13845478142e-09	5.23047712297e-09	-
YFL017W-A	128.401016	112.08683	0.196039844	3.17311504941e-09	5.28682185366e-09	-
YHR048W	12.160123	9.914682	0.294519416	3.2467630819e-09	5.40807759926e-09	-
YBR096W	25.838467	20.872068	0.307946928	3.25533604272e-09	5.4209029381e-09	-
YJR010C-A	55.474594	43.771042	0.341850475	3.2813366881e-09	5.46273482428e-09	-
YLR079W	244.441147	249.111908	-0.027306831	3.36153641197e-09	5.59475014626e-09	-
YDL056W	22.81838	29.786266	-0.384450908	3.53143547235e-09	5.87594516546e-09	-
YCR046C	54.470047	75.882072	-0.478295961	3.55699194144e-09	5.91688262928e-09	-
YBR077C	243.660446	241.116791	0.015139952	3.6366131498e-09	6.04770812589e-09	-
YLR001C	37.470428	47.16637	-0.332006116	3.89252628201e-09	6.47155949617e-09	-
YGL168W	27.072437	44.038303	-0.701934115	4.10132527286e-09	6.81687477749e-09	-
YLR236C	12.167357	6.286526	0.952680925	4.10843544298e-09	6.82686489026e-09	-
YGR021W	37.51437	51.367233	-0.453405032	4.15420445581e-09	6.90107071492e-09	-
YER039C-A	36.344852	60.306473	-0.730561832	4.28451770839e-09	7.1156463172e-09	-
YAR003W	9.268688	6.934622	0.418547892	4.2902749762e-09	7.12330223382e-09	-

YOR120W	214.204224	218.079559	-0.02586762	4.46155026998e-09	7.40569627701e-09	-
YMR219W	18.182596	17.691557	0.0394971709844	4.51474184216e-09	7.49198539324e-09	-
YMR317W	9.462926	12.961189	-0.453839822	4.52762847985e-09	7.51136226695e-09	-
YNL310C	105.168304	137.453903	-0.386247907	4.61671500962e-09	7.65711108194e-09	-
YKL119C	21.75131	16.881145	0.36568953	4.84303500493e-09	8.03033154664e-09	-
YDR365W-B	2.189844	1.63253	0.423718595	5.32254958696e-09	8.82306751291e-09	-
YDR261W-B	10.20494	13.348137	-0.387370699	5.37467426689e-09	8.90709499435e-09	-
YPR204W	12.668749	11.464931	0.144046398	5.56086478072e-09	9.2131968398e-09	-
YML094W	30.112963	23.602242	0.351460762	5.66274691333e-09	9.37949115207e-09	-
YIL050W	103.961784	101.124283	0.0399238224288	5.70569341629e-09	9.44810475549e-09	-
YHR050W	57.26141	72.111115	-0.332658454	5.72118583568e-09	9.47123244477e-09	-
YNR069C	7.728909	5.747384	0.427359337	5.7827485441e-09	9.57059529772e-09	-
YDL112W	3.896242	3.13348	0.314317503	6.17967433865e-09	1.02247916563e-08	-
YHR104W	184.952332	187.579361	-0.020347609	6.21728382248e-09	1.02842787024e-08	-
YBR088C	9.028553	5.910926	0.611110627	6.37400803839e-09	1.05407144119e-08	-
YIL099W	10.987141	8.960746	0.294125277	6.48634220375e-09	1.072362514e-08	-
YDR479C	66.875031	83.378456	-0.318207001	6.54843296118e-09	1.0823395054e-08	-
YCR100C	3.909532	2.002213	0.965400457	6.57732284427e-09	1.08682513221e-08	-
YML133C	7.910551	7.0172	0.172882706	6.69009865558e-09	1.10516584497e-08	-
YER023W	129.054199	127.463753	0.0178900380393	6.77992213156e-09	1.11970621423e-08	-
YHR180W	36.441998	29.641163	0.298000005	6.8529772749e-09	1.13147029049e-08	-
YBR093C	30.190641	40.258457	-0.415190491	6.96134194436e-09	1.14905638901e-08	-

YAL027W	34.046761	47.452988	-0.478981378	6.97751573354e-09	1.15141992753e-08	-
YGR120C	25.279997	20.9536	0.27079816	7.09067880335e-09	1.16978299259e-08	-
YDL069C	9.794863	16.665117	-0.766734227	7.35937820798e-09	1.21378905194e-08	-
YGL065C	20.185476	17.780375	0.183031856	7.70951053264e-09	1.27119897441e-08	-
YOR159C	47.054314	71.414604	-0.601892133	7.75886292199e-09	1.27899684067e-08	-
YPR158W-A	26.011127	23.239851	0.16252809	8.03359588823e-09	1.32393319018e-08	-
YEL052W	61.962917	60.622318	0.0315560456864	8.25448684538e-09	1.35997490276e-08	-
YDR164C	24.064104	31.50058	-0.388495685	8.33723680972e-09	1.37324399349e-08	-
YBL003C	235.542007	296.248688	-0.330824389	8.81860580794e-09	1.45214601739e-08	-
YPL257W-B	4.14243	3.491607	0.246586134	9.15835163e-09	1.50769151204e-08	-
YDR148C	631.875854	675.043762	-0.095339896	9.39108656339e-09	1.54559558286e-08	-
YMR038C	85.939507	110.724297	-0.365578431	9.53665636981e-09	1.56913762904e-08	-
YOR363C	16.257868	21.393047	-0.3960039	9.67822082004e-09	1.59200834263e-08	-
YOR109W	9.202832	12.61807	-0.455341462	9.91596858159e-09	1.63068431668e-08	-
YMR182W-A	38.685181	18.920717	1.031814263	9.92443668716e-09	1.63164467569e-08	up
YER039C	22.108347	32.4776	-0.554853864	1.01663305979e-08	1.67097115043e-08	-
YNR043W	47.546989	61.806042	-0.378393897	1.05279286054e-08	1.72994655326e-08	-
YOR018W	24.000778	31.056511	-0.371814589	1.06775937356e-08	1.75407520272e-08	-
YJR141W	13.265337	10.34851	0.358238267	1.08027933321e-08	1.77417304249e-08	-
YJL059W	14.287432	11.645109	0.295022489	1.18878857641e-08	1.95186444919e-08	-
YHR118C	18.016632	15.386652	0.227649993	1.21109899754e-08	1.98797000971e-08	-
YDR394W	316.727356	374.039795	-0.239950291	1.2281620631e-08	2.01544543688e-08	-

YLR227W-B	3.873805	3.240113	0.257707205	1.25641643358e-08	2.06126670711e-08	-
YER021W	332.321503	350.804413	-0.07808725	1.29563049279e-08	2.12503939214e-08	-
YKR002W	38.407429	36.644245	0.0677987527898	1.31154137358e-08	2.15056757717e-08	-
YDR402C	2.78453	1.525505	0.868146928	1.32071716761e-08	2.16504150423e-08	-
YKL042W	18.125696	15.079761	0.26542283	1.33983414376e-08	2.19579999061e-08	-
YKR037C	30.704754	26.782505	0.197171141	1.41689823087e-08	2.32148435399e-08	-
YNR060W	6.685345	9.984502	-0.578688459	1.42852663463e-08	2.33991908912e-08	-
YDR263C	6.78317	4.794336	0.50062863	1.43008922473e-08	2.34186069826e-08	-
YPL207W	34.226379	33.263721	0.0411591069147	1.43490885224e-08	2.34913347962e-08	-
YER046W	61.456509	53.419724	0.202193296	1.45882598773e-08	2.38765930182e-08	-
YMR109W	15.27373	19.840744	-0.3774137	1.48318124892e-08	2.4268817062e-08	-
YLR301W	68.062256	63.501255	0.100069867	1.53721495891e-08	2.51463253358e-08	-
YNL184C	25.860188	18.095806	0.515077396	1.68011234199e-08	2.74766528427e-08	-
YDR410C	16.190197	24.907204	-0.621442538	1.73597033517e-08	2.83826806445e-08	-
YLR436C	19.108335	24.349228	-0.349673957	1.74806573295e-08	2.85729122437e-08	-
YHR070W	21.547878	29.276342	-0.442189502	1.79990356471e-08	2.94124804679e-08	-
YDR382W	656.884399	673.657532	-0.036375851	1.82901448755e-08	2.98803208914e-08	-
YOR271C	30.524666	41.769932	-0.452489285	1.83368706531e-08	2.99487747999e-08	-
YLR381W	9.309319	7.807621	0.253792612	1.86124638874e-08	3.03908931649e-08	-
YJL161W	120.573997	115.212708	0.0656189537291	1.861500418e-08	3.03870486325e-08	-
tD(GUC)N	7.409319	28.067625	-1.921494139	1.91983681736e-08	3.13310908574e-08	down
YIR034C	30.417952	41.116497	-0.434794335	1.96276581898e-08	3.20232594067e-08	-

YBR168W	19.862854	17.143208	0.212435826	1.96391565142e-08	3.2033600536e-08	-
YOR042W	394.666901	416.113953	-0.076343133	1.97692351794e-08	3.22373028615e-08	-
YGL179C	3.479544	2.203353	0.659197603	1.99095910771e-08	3.24576526804e-08	-
YDR042C	1.378822	4.239851	-1.620577342	2.09579064707e-08	3.41577010685e-08	down
YDL137W	656.121948	685.520264	-0.063235331	2.14039019666e-08	3.48754392148e-08	-
YJL184W	65.956985	92.151886	-0.48248824	2.14382467929e-08	3.49222346078e-08	-
YLR324W	74.445175	92.212402	-0.308782451	2.20741155061e-08	3.5948612031e-08	-
YMR159C	45.351036	38.144825	0.249648164	2.23240645145e-08	3.63461296894e-08	-
YPL122C	15.311802	21.451321	-0.486422414	2.25512936645e-08	3.67064580674e-08	-
YOR289W	241.408539	245.765915	-0.025808136	2.27188232704e-08	3.69694508159e-08	-
YMR162C	7.180991	9.656377	-0.427299048	2.36563329199e-08	3.84849357356e-08	-
YMR257C	8.246513	11.780065	-0.514491383	2.47917204049e-08	4.03214567131e-08	-
YIL143C	101.8405	121.873245	-0.259070036	2.50289853808e-08	4.06966844536e-08	-
YNR070W	13.40569	17.431036	-0.37881284	2.5543700206e-08	4.15227260745e-08	-
YBL080C	8.460372	12.661024	-0.581601087	2.56989930329e-08	4.17642274211e-08	-
YKR090W	15.252904	13.652854	0.159881379	2.68464083206e-08	4.36175092526e-08	-
YBR008C	13.176479	11.199489	0.234531999	2.71283551732e-08	4.4064057801e-08	-
YJR013W	22.198008	30.642143	-0.46508698	2.73203191101e-08	4.43642534752e-08	-
YOR341W	5.788197	7.934702	-0.455062017	2.82511620525e-08	4.58638111981e-08	-
YIL023C	56.947483	54.111958	0.0736846331303	2.83423705617e-08	4.59998526659e-08	-
YIL105C	118.062271	121.741669	-0.044275051	2.84421715597e-08	4.61497650399e-08	-
YER019C-A	423.593933	525.238098	-0.310289639	2.92436536791e-08	4.74378369585e-08	-

YBR081C	9.541006	8.697862	0.133480572	2.93378986451e-08	4.757828495e-08	-
YJR010W	7.574491	5.781499	0.389705347	3.03755898278e-08	4.92482793552e-08	-
YHR182W	10.536779	14.646279	-0.475100268	3.11974208778e-08	5.05675166604e-08	-
YGR169C-A	97.402473	85.390182	0.189888201	3.17650211458e-08	5.14740932584e-08	-
YPR166C	85.630074	75.937401	0.173306949	3.20649500588e-08	5.1946557924e-08	-
YKR063C	23.535	31.61269	-0.42569595	3.25202899321e-08	5.26704826241e-08	-
YCR036W	35.571537	47.749619	-0.424765909	3.35487837667e-08	5.43220786707e-08	-
YDR326C	5.927116	5.160288	0.199878707	3.41813548406e-08	5.5331903742e-08	-
YDL120W	59.629238	81.01738	-0.44221153	3.446171507e-08	5.57712010309e-08	-
YEL049W	23.073498	37.139412	-0.686714238	3.46213137239e-08	5.60148854829e-08	-
YLR373C	10.611307	14.519837	-0.452422893	3.55125226858e-08	5.74418292948e-08	-
YIL024C	53.519573	72.852486	-0.444911598	3.56959606659e-08	5.77235019052e-08	-
YER186C	97.122284	95.212822	0.0286464798353	3.61662224684e-08	5.8468726324e-08	-
YJL176C	23.296907	30.069099	-0.36814321	3.68002043589e-08	5.94781746056e-08	-
YBR041W	20.017252	18.382664	0.122898076	3.71661876885e-08	6.00540586076e-08	-
YJR142W	36.084354	32.845802	0.135664435	3.73521674665e-08	6.03388643331e-08	-
YDL177C	50.58868	69.953102	-0.467573438	3.92436352501e-08	6.33778583852e-08	-
YLR431C	22.167738	19.729645	0.168096567	3.99751643395e-08	6.45424759998e-08	-
YOR380W	18.613483	25.31296	-0.443528179	4.03178909329e-08	6.50789045532e-08	-
YLR429W	39.43708	49.90699	-0.339689159	4.10049798138e-08	6.61707602506e-08	-
YER048W-A	212.026688	202.151749	0.0688071838688	4.19190931726e-08	6.76283083201e-08	-
YPR082C	210.838654	264.186035	-0.32541482	4.26566574363e-08	6.88003453791e-08	-

YEL048C	9.725333	5.661522	0.780557701	4.31351241989e-08	6.95539872797e-08	-
YKL124W	50.317303	63.176426	-0.328331728	4.32666047781e-08	6.97478791126e-08	-
YNL272C	16.470482	15.025244	0.132494356	4.36361004749e-08	7.03252626553e-08	-
YJL167W	418.732117	440.973083	-0.074663018	4.45361078235e-08	7.17571132541e-08	-
YMR190C	5.356106	4.619521	0.213441247	4.53080118317e-08	7.2981872717e-08	-
YGL169W	15.005078	21.408113	-0.512706818	4.67731004376e-08	7.53222846994e-08	-
YJL155C	303.782654	319.302917	-0.071886238	4.91044000319e-08	7.90560465243e-08	-
YHL025W	152.96463	186.600235	-0.286752706	4.98171258563e-08	8.01827112564e-08	-
YPL273W	20.316553	17.132765	0.245897647	5.15275658961e-08	8.29142377094e-08	-
YBR167C	48.402466	41.028812	0.23844317	5.25797175999e-08	8.45853554963e-08	-
YDR145W	81.175995	99.688286	-0.296370826	5.27143059533e-08	8.47798993155e-08	-
YCL019W	4.617634	6.407019	-0.472499441	5.32902804068e-08	8.56840354223e-08	-
YMR111C	12.611963	18.167953	-0.526603036	5.38066166587e-08	8.64918374462e-08	-
YEL055C	9.533105	8.473206	0.170038243	5.43813119484e-08	8.73930066207e-08	-
YOR339C	10.058572	17.971725	-0.837303389	5.4587297335e-08	8.7701330708e-08	-
YLL039C	776.335266	898.063293	-0.2101373	5.4624158987e-08	8.7737847087e-08	-
YPR125W	22.183077	30.133442	-0.44190598	5.49518238023e-08	8.82413145795e-08	-
YGR075C	14.807405	22.786713	-0.621874	5.55762259395e-08	8.92208975e-08	-
YPR058W	38.178905	34.67408	0.138918121	5.68049222091e-08	9.11698441246e-08	-
YDR457W	21.956802	22.572254	-0.039882547	5.77418808464e-08	9.26496759613e-08	-
YOR378W	18.521933	25.287748	-0.449203893	5.82167227076e-08	9.33874456251e-08	-
YDR108W	5.178316	7.947789	-0.618070566	5.93402994877e-08	9.51652232548e-08	-

YOR324C	21.751837	28.857412	-0.407804676	6.14775296947e-08	9.85672790147e-08	-
YOR391C	65.431793	61.128864	0.0981380480579	6.17898418095e-08	9.90424316946e-08	-
YGL070C	22.918932	36.545124	-0.673139111	6.34648667146e-08	1.01701056418e-07	-
YDR288W	33.492668	29.95507	0.1610451	6.47873186555e-08	1.03793464313e-07	-
YLR052W	62.217274	81.318619	-0.386270529	6.66648856803e-08	1.06773893267e-07	-
YCR101C	3.857433	1.56049	1.305641987	6.81202702721e-08	1.09076770144e-07	up
YCL058C	20.05909	14.713414	0.44712412	7.10522237508e-08	1.13742187995e-07	-
YPL100W	75.512054	93.356316	-0.306040671	7.2692362942e-08	1.16337764667e-07	-
YER135C	6.571159	3.01746	1.122813204	7.43621932085e-08	1.18979509134e-07	up
YJR034W	68.210312	95.445946	-0.48469406	7.43730529933e-08	1.18966223392e-07	-
YBR111W-A	146.780289	136.023071	0.109806874	7.44452001705e-08	1.19050953802e-07	-
YJR122W	32.305008	42.115932	-0.38260826	7.47113104679e-08	1.19445741794e-07	-
YPL031C	141.017334	141.69664	-0.006933037	7.61799344089e-08	1.21762366841e-07	-
YLR384C	18.256271	23.103632	-0.339727555	7.77540669186e-08	1.24246395735e-07	-
YML121W	53.905807	70.047882	-0.377900734	7.81455107821e-08	1.24839766067e-07	-
YGR093W	12.203562	17.40402	-0.512118274	7.8151682619e-08	1.24817505968e-07	-
YPR121W	3.427569	5.80572	-0.760289286	8.27966866023e-08	1.32202116879e-07	-
YOL022C	39.262604	36.852867	0.0913789999687	8.43318415138e-08	1.34618686582e-07	-
YLL035W	1.062315	2.332354	-1.134575154	8.60166631483e-08	1.37272864994e-07	down
YDR285W	1.513865	0.859791	0.816178644	8.62802675532e-08	1.37658160105e-07	-
YKL057C	19.417574	18.583607	0.0633324141102	8.87897543207e-08	1.41625589626e-07	-
YMR017W	6.409455	10.363225	-0.693199441	9.29941615215e-08	1.48293797772e-07	-

YBR018C	3.123184	1.671469	0.901900969	9.35448705723e-08	1.49133681693e-07	-
YGR036C	17.191208	25.789185	-0.585095257	9.42067900089e-08	1.50150385719e-07	-
YOR323C	78.80146	97.548653	-0.307899592	9.53797784885e-08	1.51980920138e-07	-
YBR291C	11.751431	18.042212	-0.618539778	9.70283486668e-08	1.54568126385e-07	-
YDR210W-B	7.951279	10.456629	-0.395158983	9.9687950979e-08	1.5876418668e-07	-
YDR300C	12.460159	18.048552	-0.534560619	1.00121704934e-07	1.59414091878e-07	-
YKR105C	12.259359	17.178267	-0.486700953	1.0095568021e-07	1.60700734037e-07	-
YDL150W	17.338268	24.225491	-0.482566118	1.0351326893e-07	1.64729652273e-07	-
YER081W	37.772919	35.828636	0.0762391550365	1.04487230406e-07	1.66236987791e-07	-
YDL089W	19.620516	17.49029	0.165808773	1.09759903453e-07	1.745809584e-07	-
YJR150C	1.912552	4.375736	-1.194026726	1.10622427331e-07	1.7590779428e-07	down
YOL158C	17.183779	23.171623	-0.431311754	1.11428616385e-07	1.77144391938e-07	-
YDL130W	344.933502	426.286804	-0.305506138	1.13521378929e-07	1.80425171631e-07	-
YPR108W	159.014954	163.446808	-0.039658758	1.13857700269e-07	1.80913387066e-07	-
YLR097C	108.884674	108.75676	0.00169582520494	1.1430851255e-07	1.81583225668e-07	-
YOL014W	24.869081	18.47216	0.429000621	1.15560777087e-07	1.83525531889e-07	-
YPL005W	24.759438	23.153927	0.0967216668604	1.21875945333e-07	1.93505337244e-07	-
YDR504C	17.631195	28.975048	-0.716680791	1.29819999134e-07	2.06065598217e-07	-
YMR194W	143.309906	187.903305	-0.390852106	1.29937459254e-07	2.06199321843e-07	-
YDR480W	26.398197	23.258881	0.182657707	1.34107583922e-07	2.12762555836e-07	-
YHR208W	14.947192	21.347652	-0.514202915	1.40644127291e-07	2.23075815591e-07	-
YFL067W	5.130391	2.460927	1.059866919	1.40772690621e-07	2.23222698181e-07	up

YGR117C	13.861808	11.81653	0.230309	1.46278920822e-07	2.31894673253e-07	-
YKL220C	2.205704	1.356867	0.700959883	1.54433446497e-07	2.44759467922e-07	-
YPR113W	67.318123	63.026508	0.095036219	1.5605164779e-07	2.47261008035e-07	-
YDR129C	615.567566	707.631531	-0.201081115	1.6042201661e-07	2.54120918375e-07	-
YGR111W	87.754669	87.453148	0.00496556932572	1.64428812158e-07	2.60401547417e-07	-
YGR251W	37.494392	52.14592	-0.475879549	1.71208999626e-07	2.71069999918e-07	-
YFR032C-A	98.745323	82.985588	0.25085161	1.75444749945e-07	2.77705509347e-07	-
YMR101C	3.436712	1.902564	0.853083972	1.8772354037e-07	2.97065444461e-07	-
YDR170W-A	18.327137	25.23307	-0.461334313	1.89861021851e-07	3.00371361786e-07	-
YLR188W	15.395055	13.975773	0.139538941	1.92230803462e-07	3.04043013475e-07	-
YOR076C	6.167966	9.052594	-0.55353644	1.92628978648e-07	3.04595185799e-07	-
YIL137C	50.773617	51.475639	-0.019810796	1.97309542951e-07	3.11916893975e-07	-
YDR067C	110.781586	139.553574	-0.333100975	2.04990702601e-07	3.23977159305e-07	-
YLL064C	2.138973	6.375886	-1.575707563	2.09807920021e-07	3.31506125602e-07	down
YGR263C	14.688217	12.461244	0.237211179	2.12287483005e-07	3.35338599109e-07	-
YNL129W	6.403232	3.939364	0.700837553	2.12545016827e-07	3.35660001136e-07	-
YDR511W	133.08313	171.24054	-0.363696587	2.18380921317e-07	3.44788596016e-07	-
YPL230W	153.732208	157.506195	-0.034989122	2.28121912582e-07	3.60076489527e-07	-
YDR254W	16.290136	14.197973	0.198313673	2.28431004904e-07	3.60472719482e-07	-
YKL208W	21.119623	17.726116	0.252707622	2.30541964427e-07	3.63711439685e-07	-
YBL039C	8.241265	12.0263	-0.545255145	2.38809019987e-07	3.76658129085e-07	-
YLL009C	300.672516	290.229248	0.0510000844057	2.41308708507e-07	3.8050405446e-07	-

YGL196W	83.199951	83.107391	0.00160589262226	2.42180234945e-07	3.81781335333e-07	-
YOL136C	8.31354	12.762465	-0.618372176	2.43404142035e-07	3.83613331748e-07	-
YKL185W	246.318909	260.110443	-0.07859694	2.471613683e-07	3.89435983352e-07	-
YDL087C	19.514206	28.203533	-0.531351134	2.64912494414e-07	4.17299356844e-07	-
YFR022W	4.928452	7.45166	-0.596427274	2.70459827133e-07	4.25929631365e-07	-
YDR239C	23.011236	29.545784	-0.360613804	2.71942909206e-07	4.28156627022e-07	-
YJL205C	255.830261	238.434998	0.101590911	2.73870445623e-07	4.31082080737e-07	-
YJL036W	109.368187	133.270996	-0.28516969	2.75225683779e-07	4.33105461317e-07	-
YBR022W	46.869682	64.179581	-0.453459364	2.78482876541e-07	4.38120044999e-07	-
YBR061C	21.504955	30.10866	-0.485509389	2.9237716756e-07	4.59862542745e-07	-
YDR238C	25.062197	31.55891	-0.332534488	2.95091771265e-07	4.64014619052e-07	-
YLR207W	25.589025	32.485081	-0.344252124	3.01735267691e-07	4.74340982989e-07	-
YMR173W	172.346588	178.241791	-0.048522897	3.08058449243e-07	4.84158696938e-07	-
YPR062W	229.624054	282.802368	-0.300520423	3.11603089492e-07	4.89605664279e-07	-
YLR364W	15.627666	10.074536	0.633388933	3.14210833448e-07	4.93578151328e-07	-
YPL112C	34.903767	32.602013	0.0984217022403	3.32125884823e-07	5.21588032629e-07	-
YBL052C	6.194639	5.126727	0.272982134	3.34177249932e-07	5.24676875969e-07	-
YDR289C	57.423229	72.435661	-0.335065671	3.34989763571e-07	5.25819583376e-07	-
YER016W	15.878099	13.287331	0.256986854	3.35660407286e-07	5.26739082011e-07	-
YOR260W	15.390965	20.911674	-0.442224865	3.38180049056e-07	5.30558944792e-07	-
YDR325W	3.894233	3.089269	0.334073706	3.45869073013e-07	5.42484892689e-07	-
YBL031W	4.516398	2.789768	0.695027476	3.54686707617e-07	5.56174559455e-07	-

YFR041C	38.457195	35.290768	0.123962712	3.59715889112e-07	5.63918242324e-07	-
YMR133W	20.830873	18.624552	0.161517579	3.61592968518e-07	5.66717785549e-07	-
YCR071C	51.596153	71.160759	-0.463818396	3.75715897357e-07	5.88703758404e-07	-
YNR026C	12.71559	17.968437	-0.498866516	3.8908081465e-07	6.09491218104e-07	-
YOR064C	188.377304	230.482269	-0.291030611	3.89777177365e-07	6.10428031555e-07	-
snR7-S	11.501924	4.972788	1.209748378	4.02466905839e-07	6.3014238372e-07	up
YDR323C	11.364002	16.111469	-0.503617051	4.02685363765e-07	6.30325450896e-07	-
YNL182C	6.589665	9.913021	-0.589119662	4.07302410271e-07	6.37391823282e-07	-
YKR019C	13.661088	18.622086	-0.446942301	4.12059783942e-07	6.44674178102e-07	-
YDR369C	6.51984	9.297264	-0.511969662	4.13131059666e-07	6.46187356615e-07	-
YML062C	39.556313	51.214256	-0.372637494	4.59696617424e-07	7.18840453644e-07	-
YIL019W	25.58799	22.917803	0.158998077	4.66510620525e-07	7.29311995019e-07	-
YKR083C	29.723516	23.618128	0.331710163	4.67599666995e-07	7.30830496653e-07	-
YOL149W	62.322819	58.614571	0.0885011423018	4.74100034611e-07	7.40803678547e-07	-
YEL026W	41.050102	34.18169	0.264162079	4.74738929178e-07	7.4161531765e-07	-
YPL150W	25.045282	31.617895	-0.336202466	4.79436214061e-07	7.48764784123e-07	-
YML059C	4.345491	3.797747	0.194375399	4.92150473452e-07	7.68428103418e-07	-
YGR255C	58.802551	58.135319	0.0164638323631	5.03401489491e-07	7.8579744701e-07	-
YDR083W	23.569973	31.810902	-0.432571174	5.17051006449e-07	8.06901118158e-07	-
YMR187C	17.779539	24.426485	-0.458228356	5.2233755363e-07	8.14946351579e-07	-
YDR272W	347.555176	363.092712	-0.063095942	5.22571067181e-07	8.15105825392e-07	-
YBR298C	58.86755	59.048096	-0.004417955	5.29454865698e-07	8.25635721239e-07	-

YNR075C-A	2.896669	13.89589	-2.262191495	5.43012708208e-07	8.4656526684e-07	down
YJL174W	226.854065	233.518448	-0.041772016	5.73224679254e-07	8.9344182998e-07	-
YOR210W	89.001251	124.691658	-0.486467431	5.96047266886e-07	9.28780480128e-07	-
YOR095C	26.684956	36.999577	-0.471482145	6.09688584715e-07	9.4979842758e-07	-
YHR184W	3.007379	1.967692	0.612002277	6.47701467508e-07	1.0087633493e-06	-
YDL166C	38.503006	52.867264	-0.457403582	6.69202761412e-07	1.04198915045e-06	-
YNL086W	62.893448	87.770645	-0.480828779	6.70129172474e-07	1.04316998564e-06	-
YPL006W	30.856598	37.909782	-0.29699115	6.75387121592e-07	1.05109131382e-06	-
YPL094C	128.07486	128.389236	-0.003536939	6.75898308682e-07	1.05162323316e-06	-
YOL112W	23.365026	21.561331	0.115904403	6.90810571182e-07	1.07455575693e-06	-
YBL111C	5.001398	3.871421	0.369468213	7.28725791478e-07	1.13324892623e-06	-
YEL076C	3.428736	1.594898	1.104212665	7.32490560261e-07	1.13881828152e-06	up
YGL145W	9.565079	13.273848	-0.47273787	7.42985669137e-07	1.15484602754e-06	-
YKR065C	136.819443	170.567139	-0.318066466	7.69670992809e-07	1.19602441135e-06	-
YML007W	236.076782	275.817169	-0.224456104	7.84454141361e-07	1.21869151891e-06	-
YDR195W	17.74947	23.810106	-0.423798097	8.29313531934e-07	1.28806064705e-06	-
YPL116W	12.985214	11.712126	0.148866811	8.6353700268e-07	1.34087986809e-06	-
YML095C	23.372545	33.517834	-0.520114086	8.70399072234e-07	1.3511971594e-06	-
YOL005C	103.587616	135.797058	-0.390600687	8.78312197783e-07	1.36314053096e-06	-
YKL089W	3.067899	2.014089	0.607123552	8.84009374982e-07	1.37163964006e-06	-
YJR054W	5.783866	4.356984	0.408704313	8.96159071991e-07	1.39014380783e-06	-
YKR003W	15.486867	13.544012	0.193390159	9.00105304509e-07	1.39591649523e-06	-

YNL087W	10.182806	9.458916	0.106388405	9.02266140596e-07	1.39891813207e-06	-
YJL198W	10.747668	9.756801	0.139543553	9.76873836925e-07	1.51421542563e-06	-
YKL009W	51.844463	68.0373	-0.392135973	9.79517272254e-07	1.51793390568e-06	-
YLL028W	45.455097	44.93	0.0167630148315	9.80802609644e-07	1.51954644389e-06	-
YMR010W	14.87173	20.819605	-0.485370214	1.0382672666e-06	1.60817444886e-06	-
YIL166C	34.167381	32.960983	0.0518603976058	1.05193709631e-06	1.62894125565e-06	-
YBR123C	21.654488	20.432932	0.0837698248559	1.05234979521e-06	1.6291739473e-06	-
YER054C	55.890678	69.114616	-0.30638316	1.0577606071e-06	1.63714232083e-06	-
YDR023W	77.076561	77.453995	-0.007047451	1.06112345857e-06	1.64193779432e-06	-
YKR020W	133.156693	129.976822	0.0348705672319	1.10061040607e-06	1.70261385519e-06	-
YBR103W	46.751282	58.429657	-0.321694894	1.10755575659e-06	1.71293127477e-06	-
YGR103W	18.480484	24.393244	-0.40047909	1.13601903546e-06	1.75651461324e-06	-
YBR202W	25.490316	24.861444	0.0360391650677	1.13924024571e-06	1.7610566348e-06	-
YGL001C	44.245747	56.963692	-0.364503868	1.1831369998e-06	1.82845767856e-06	-
YOL107W	5.177879	3.475194	0.575267734	1.192002182e-06	1.84169973764e-06	-
YMR235C	33.440575	43.467472	-0.378336539	1.20806578765e-06	1.8660543443e-06	-
YJR143C	11.031644	9.9003	0.156103658	1.23091210106e-06	1.90087122473e-06	-
YOR005C	13.660516	12.812966	0.0924075041792	1.2781674693e-06	1.97335579444e-06	-
YJL209W	6.673493	9.687554	-0.537690361	1.28814394613e-06	1.98826395266e-06	-
YAL002W	14.167851	18.045431	-0.349012656	1.33685255589e-06	2.06293330026e-06	-
YPR147C	69.022743	87.060577	-0.334947773	1.40029374651e-06	2.16029412981e-06	-
YKL039W	61.863968	76.20182	-0.300726083	1.4046200216e-06	2.16643008548e-06	-

YER139C	57.676258	54.219791	0.0891580143946	1.46925873944e-06	2.26556340149e-06	-
YLR078C	62.018929	79.888847	-0.365285495	1.48527825067e-06	2.28969639438e-06	-
YLR239C	27.528297	36.94627	-0.424513359	1.48566317665e-06	2.28972120175e-06	-
YHR088W	75.736237	95.145157	-0.329146482	1.49881993539e-06	2.30942520697e-06	-
YPR173C	79.374115	79.789124	-0.007523506	1.511722496e-06	2.32872785488e-06	-
YNR007C	90.068275	111.731506	-0.310945118	1.55248184644e-06	2.3909221788e-06	-
YOL057W	25.475281	32.410152	-0.347347727	1.57355153772e-06	2.42276982792e-06	-
tN(GUU)N1	19.210613	6.914826	1.474138701	1.58851264703e-06	2.44519873859e-06	up
YHR119W	8.771413	11.710796	-0.416957969	1.59735795452e-06	2.45820480457e-06	-
YLR313C	2.2424	1.332628	0.750769538	1.60391645365e-06	2.46768608285e-06	-
YDR029W	56.736671	79.227249	-0.481715205	1.6070966252e-06	2.47196626592e-06	-
YOR252W	59.148464	78.245575	-0.403668462	1.61111926523e-06	2.47753985598e-06	-
YCL002C	16.805475	24.301682	-0.532124851	1.63965923372e-06	2.5208034975e-06	-
YGL254W	49.600838	47.177475	0.0722662892485	1.6725269485e-06	2.57069752322e-06	-
YJR120W	541.864502	559.748596	-0.046846866	1.6745846105e-06	2.57322308465e-06	-
YDL179W	32.277431	29.561659	0.126798522	1.6859595857e-06	2.59006115021e-06	-
YIL114C	21.844866	18.900692	0.2088552	1.70931455592e-06	2.62529063908e-06	-
YBR010W	278.836548	281.792511	-0.015213598	1.74126809482e-06	2.67370574639e-06	-
YBR164C	41.159237	36.607296	0.169085025	1.77319135397e-06	2.72205042667e-06	-
YDR295C	9.925583	8.696099	0.19078348	1.80136212573e-06	2.76461213264e-06	-
YNL326C	13.737497	19.726042	-0.521982344	1.82213425009e-06	2.79580064868e-06	-
YNL138W-A	109.038155	145.728073	-0.418445768	1.83759585602e-06	2.81882754489e-06	-

YLR288C	31.523224	30.01355	0.0708011213797	1.87497429249e-06	2.8754546462e-06	-
YLR047C	7.111827	10.141421	-0.511967679	1.94805851882e-06	2.9867985391e-06	-
YJR153W	22.299942	19.992123	0.157608275	1.9591518646e-06	3.00306537664e-06	-
YLR026C	36.695454	47.842201	-0.382682416	1.98085027766e-06	3.03557603647e-06	-
YDR168W	95.950424	115.881966	-0.272294979	2.13725521513e-06	3.27445221508e-06	-
YPR111W	20.521978	19.13011	0.101324622	2.14376637672e-06	3.28361748499e-06	-
YLR255C	4.486926	9.801379	-1.127257352	2.1470788874e-06	3.28788005254e-06	down
YHR044C	18.880339	15.742046	0.262261608	2.1484285094e-06	3.28913543437e-06	-
YPR056W	26.057945	34.927261	-0.422630197	2.15123916682e-06	3.29262641707e-06	-
YJR022W	17.100634	11.84139	0.530211372	2.19135451245e-06	3.35319911592e-06	-
YDR489W	9.414975	7.131377	0.40077659	2.20002854068e-06	3.36564247919e-06	-
YDL197C	56.032516	55.91621	0.00299769645964	2.21641148449e-06	3.38987004083e-06	-
YMR247C	3.999505	3.486311	0.198120183	2.25346791398e-06	3.44569675124e-06	-
YLL014W	55.441051	77.122604	-0.476199157	2.26113042866e-06	3.45656185696e-06	-
YHR218W	4.42781	3.328544	0.411702077	2.3129481227e-06	3.53490446719e-06	-
YDR421W	20.46327	19.904394	0.0399497555492	2.36277336454e-06	3.610164176e-06	-
YKL209C	10.263227	9.692476	0.0825472567647	2.4261032582e-06	3.70601600071e-06	-
YOR163W	155.144257	191.570541	-0.304265433	2.56695139679e-06	3.92020523278e-06	-
YJL213W	26.690571	24.258993	0.137810505	2.58078593825e-06	3.94036377389e-06	-
YLR215C	12.430017	17.865801	-0.523372328	2.67246882009e-06	4.0793426199e-06	-
HRA1	4.558827	8.728571	-0.93708282	2.7003506422e-06	4.1208890823e-06	-
YIL152W	81.395081	102.901588	-0.338251731	2.72297910841e-06	4.15440017326e-06	-

YIL034C	229.592133	273.973328	-0.254962241	2.73562952391e-06	4.17267520502e-06	-
YDR273W	12.938329	18.448843	-0.511879038	2.78612866692e-06	4.24865801136e-06	-
YJL136C	389.261108	391.895508	-0.009730828	2.81024521268e-06	4.28438169949e-06	-
YMR212C	10.110459	9.089722	0.153540418	2.96278741331e-06	4.5158321291e-06	-
YDL244W	5.891644	4.160274	0.50199171	2.96693932571e-06	4.52105040108e-06	-
YNL331C	24.575495	32.740459	-0.413854072	3.0519180017e-06	4.64940047965e-06	-
YJL216C	1.482954	0.788086	0.91204887	3.10412249017e-06	4.72777046589e-06	-
YLL033W	17.283846	14.138522	0.289792969	3.11983041716e-06	4.75052912184e-06	-
YDL214C	57.644459	70.061287	-0.281435553	3.15672667095e-06	4.80553192086e-06	-
YGL159W	19.520041	26.602657	-0.446614262	3.15904152264e-06	4.80787687486e-06	-
YML108W	38.735085	56.145908	-0.535539975	3.20338034447e-06	4.87416303394e-06	-
YLR298C	76.218079	96.734764	-0.343901204	3.20695174673e-06	4.87840148094e-06	-
YML060W	3.872566	2.519908	0.619918764	3.27778015579e-06	4.98492386261e-06	-
YNL083W	18.425753	24.567114	-0.41500481	3.28426666149e-06	4.99356537706e-06	-
YNL271C	21.192595	25.72613	-0.279674193	3.32500726017e-06	5.05427156492e-06	-
YDR381W	305.037842	316.808716	-0.054623797	3.33702678435e-06	5.07130043506e-06	-
YGR047C	14.808152	18.974657	-0.357682198	3.36755856111e-06	5.11644727053e-06	-
YKL197C	22.004364	21.68865	0.020849418	3.43814281849e-06	5.22241023175e-06	-
YHR196W	8.31119	11.812525	-0.50719042	3.46443659885e-06	5.26106223231e-06	-
YKR098C	16.056444	15.016747	0.0965800932609	3.48883580454e-06	5.29681894707e-06	-
YNL293W	27.643175	35.084827	-0.34392392	3.4972462874e-06	5.30828971936e-06	-
YDR484W	13.480363	12.254767	0.137516292	3.50113557458e-06	5.31289407161e-06	-

YMR320W	16.42473	27.194273	-0.727433203	3.50876907525e-06	5.32317654427e-06	-
YIL003W	32.637566	29.989239	0.122088557	3.57124134178e-06	5.41662991687e-06	-
YNL143C	45.213005	39.263039	0.203565964	3.59518270759e-06	5.45161071048e-06	-
YMR292W	34.832817	49.43119	-0.504974487	3.63076208296e-06	5.50421758511e-06	-
YDL121C	81.259338	76.38546	0.0892355644619	3.63274313921e-06	5.50587632036e-06	-
YOL146W	43.021404	38.969391	0.142713222	3.63863511867e-06	5.5134602921e-06	-
YNL286W	55.191586	53.0103	0.0581756385458	3.64239392431e-06	5.51780904884e-06	-
YDR506C	22.78723	29.381006	-0.366658232	3.66906716271e-06	5.55685995269e-06	-
YDR404C	60.693813	79.904083	-0.396719766	3.7641363055e-06	5.69945321574e-06	-
YDL168W	94.602982	95.728416	-0.017061578	3.92534397662e-06	5.94209592949e-06	-
YJR108W	33.464439	48.31089	-0.529719602	3.96110005647e-06	5.99476088507e-06	-
YJL002C	85.91317	104.25705	-0.279193734	3.99567081607e-06	6.04560673317e-06	-
YJR125C	154.638214	184.591324	-0.255437866	4.03072233985e-06	6.0971550404e-06	-
YEL044W	250.509705	255.164795	-0.026562799	4.07422641639e-06	6.16146104579e-06	-
YPL047W	38.965897	56.666279	-0.540278443	4.07669767699e-06	6.16369682872e-06	-
YOL076W	10.519721	14.099024	-0.422498853	4.16157861624e-06	6.29049915988e-06	-
tL(UAG)J	5.326401	0.0001	15.70087343	4.20177129885e-06	6.34970696769e-06	up
YMR185W	11.497545	15.03385	-0.38688867	4.24460229733e-06	6.41287200336e-06	-
YLR341W	1.563646	0.79096	0.98323729	4.28699916002e-06	6.47535055606e-06	-
YDR183W	24.593891	21.290314	0.208102774	4.40726884722e-06	6.65539406556e-06	-
YKL005C	54.884579	67.262512	-0.293401809	4.44070983892e-06	6.70426232491e-06	-
YOR376W-A	189.175171	250.044281	-0.402460859	4.4471490129e-06	6.71235134259e-06	-

YLR073C	9.413973	15.178014	-0.68910741	4.44987290677e-06	6.71483009364e-06	-
YPL139C	22.948231	30.148203	-0.393689067	4.52329495494e-06	6.82396478257e-06	-
YDL236W	37.84697	49.281078	-0.380856011	4.53595633326e-06	6.84140352694e-06	-
YCR106W	2.56297	1.859995	0.462517849	4.62008637122e-06	6.96660096977e-06	-
YPR189W	7.820982	10.221606	-0.386200219	4.76663517758e-06	7.1858356441e-06	-
YMR183C	68.820786	67.441589	0.0292058409381	4.82024685414e-06	7.26489256385e-06	-
YCL032W	25.714268	34.245029	-0.413325496	4.85609380656e-06	7.31714328911e-06	-
YLR452C	4.400578	3.453927	0.349455438	4.86649892814e-06	7.3310423067e-06	-
YLR132C	10.861166	8.587005	0.338952055	4.86733702401e-06	7.33052601773e-06	-
YBR110W	30.104206	38.79578	-0.365934665	4.93338731798e-06	7.42819996847e-06	-
YOL021C	14.015246	18.015759	-0.362262366	4.94293107771e-06	7.44076530806e-06	-
YHR133C	28.685179	38.389977	-0.420424175	4.95546629126e-06	7.4578266027e-06	-
YPL011C	44.313969	56.550697	-0.351783258	5.0444790471e-06	7.58994811546e-06	-
YEL067C	23.425274	19.716612	0.248654255	5.14480209191e-06	7.7390189936e-06	-
YGR280C	58.237625	56.078262	0.0545098844493	5.30598697539e-06	7.97954630407e-06	-
YER066W	25.528936	21.619209	0.239819671	5.32854677678e-06	8.01153266899e-06	-
YOR097C	81.185638	104.170364	-0.359648458	5.43591371696e-06	8.17098119973e-06	-
YGR161W-B	5.764232	7.581973	-0.395444916	5.45572213634e-06	8.19877100517e-06	-
YCR095C	28.888948	37.926262	-0.392679518	5.4784243992e-06	8.23089512832e-06	-
YPR100W	259.004578	262.427673	-0.018942262	5.49323554679e-06	8.25115080437e-06	-
YPR131C	22.329264	18.673717	0.257926575	5.55292312888e-06	8.33878732078e-06	-
YBR240C	12.242616	17.034338	-0.476534016	5.55852452967e-06	8.34518023705e-06	-

YLR462W	4.773257	2.723224	0.809658359	5.61928554001e-06	8.43436282215e-06	-
YMR270C	13.538288	11.538958	0.230532362	5.64315043827e-06	8.46813582808e-06	-
YLR015W	24.866959	23.537031	0.0792977421131	5.70246509883e-06	8.555075721e-06	-
YER120W	325.894928	385.752563	-0.243268843	5.71697708098e-06	8.57477499848e-06	-
YML065W	10.391635	9.588255	0.11608248	5.87451877588e-06	8.80894023205e-06	-
YOR194C	105.801689	129.942673	-0.296512629	5.93851241842e-06	8.9027493585e-06	-
YDR459C	16.068022	22.146585	-0.462891911	5.98797524853e-06	8.9747345106e-06	-
YCL009C	188.180115	194.64859	-0.048757708	6.0731622669e-06	9.10021514674e-06	-
YLR345W	305.30957	355.179749	-0.218276515	6.10218910781e-06	9.14150337386e-06	-
YHL035C	7.715085	7.288627	0.0820349800984	6.1958257479e-06	9.27953829746e-06	-
YHR123W	5.563853	4.092076	0.443251364	6.31793269616e-06	9.46013656001e-06	-
YBR030W	4.650399	7.114494	-0.613406649	6.39415885634e-06	9.57196483728e-06	-
YMR069W	5.667671	3.849653	0.558027604	6.40257878453e-06	9.58225870164e-06	-
YGR145W	13.669183	18.036232	-0.399970956	6.42584044923e-06	9.61475476231e-06	-
YMR210W	36.447113	46.29562	-0.345071165	6.52843534344e-06	9.765910027e-06	-
YPL125W	15.593384	19.817177	-0.345817413	6.7645657423e-06	1.0116700585e-05	-
YFL002W-A	6.276365	8.191849	-0.384259868	6.7992285152e-06	1.01660911904e-05	-
YIR008C	13.809045	19.125666	-0.469896437	7.41885388601e-06	1.10898735671e-05	-
YOL026C	202.312958	200.349396	0.0140705660047	7.70045609793e-06	1.15080480154e-05	-
YLR036C	29.442753	25.853136	0.187573284	7.77864771834e-06	1.16221047017e-05	-
YKL047W	10.820292	9.431656	0.198156428	8.19448260306e-06	1.22404590952e-05	-
YER119C	59.124733	59.068001	0.00138497483856	8.30217189918e-06	1.23983360958e-05	-

YDR316W	16.676683	22.337976	-0.421666107	8.32478231318e-06	1.24291122175e-05	-
YLR218C	81.254135	76.945869	0.0785973587099	8.39529136552e-06	1.25313702325e-05	-
YPR093C	64.685669	81.367401	-0.331004789	8.40259544284e-06	1.25392578147e-05	-
YGL079W	37.078876	49.550377	-0.418298523	8.43898355974e-06	1.25905335109e-05	-
YNL214W	36.13018	32.521084	0.151829096	8.62497022946e-06	1.28649243596e-05	-
YLR147C	43.841934	62.169598	-0.503897809	8.73625808424e-06	1.30277901002e-05	-
YDR336W	20.901321	28.444311	-0.444546009	8.98613120653e-06	1.33971908093e-05	-
YGR024C	114.172195	114.217552	-0.000573023	9.0725775451e-06	1.35228238655e-05	-
YLR152C	43.946236	54.343884	-0.306378076	9.08203781916e-06	1.35336751756e-05	-
YBL013W	11.740002	10.003265	0.230961691	9.16861417084e-06	1.36594088727e-05	-
YKL201C	11.637113	11.131228	0.0641204308343	9.41342235052e-06	1.40207595854e-05	-
YER176W	14.403662	18.271893	-0.343190457	9.67233445608e-06	1.4402938907e-05	-
YLR094C	151.792847	179.611649	-0.242777114	1.02302695768e-05	1.52300991685e-05	-
YGL231C	93.600494	91.347725	0.0351473447939	1.02326938174e-05	1.52300559142e-05	-
YBR004C	121.474213	145.497253	-0.26034183	1.03404640536e-05	1.53867691382e-05	-
YER076C	15.246125	12.967463	0.233546356	1.05125766841e-05	1.56391267805e-05	-
YCR107W	4.874032	3.451859	0.497742188	1.07396828263e-05	1.59731554829e-05	-
YBL059C-A	244.746719	244.905655	-0.000936567	1.0789609171e-05	1.60435673613e-05	-
YOR205C	9.14635	12.755518	-0.479853455	1.08788022829e-05	1.61723191026e-05	-
YKR106W	12.397156	16.611534	-0.422176112	1.09257546264e-05	1.62382295237e-05	-
YKL138C-A	137.525696	127.634659	0.10768106	1.10370583067e-05	1.63997266557e-05	-
YJL151C	1764.339111	2033.698364	-0.204977838	1.12171673096e-05	1.66633583771e-05	-

YDR126W	10.098801	14.750684	-0.546597839	1.13081172327e-05	1.67944477943e-05	-
YOR174W	31.747118	41.916615	-0.400896586	1.17525510331e-05	1.74503316943e-05	-
YGR068C	8.668623	12.075547	-0.478213796	1.20587488338e-05	1.79006964993e-05	-
YMR288W	10.455537	13.681818	-0.387992783	1.22326923278e-05	1.81545670502e-05	-
YEL005C	85.609375	85.236252	0.00630163694622	1.22677765143e-05	1.82022840824e-05	-
YDR518W	10.702971	14.769824	-0.46464131	1.23466292956e-05	1.8314904341e-05	-
YGR167W	433.766754	508.914307	-0.230503268	1.23760801899e-05	1.83542058813e-05	-
YKL041W	82.59729	80.940514	0.029232437	1.23898721185e-05	1.83702713427e-05	-
YJR133W	80.158592	78.019226	0.0390274815719	1.29060036811e-05	1.91309624767e-05	-
YLR002C	7.756563	10.784645	-0.475489259	1.31514169055e-05	1.94900921818e-05	-
YDR076W	19.160879	17.459055	0.134188273	1.31605827366e-05	1.94990209139e-05	-
YNL275W	9.854502	13.569403	-0.461502378	1.31904781402e-05	1.95386514661e-05	-
snR17a	27.233446	23.174473	0.232843015	1.32952270142e-05	1.96891148149e-05	-
YOL116W	39.512276	50.233116	-0.346337818	1.33084364017e-05	1.97039764325e-05	-
YKL133C	72.01664	72.987267	-0.01931451	1.36700924901e-05	2.02346051927e-05	-
YNL122C	85.962868	112.526596	-0.388480505	1.37357387406e-05	2.03269287488e-05	-
YLR030W	6.219156	4.330433	0.522207519	1.37827534176e-05	2.03916428066e-05	-
YJR052W	36.971775	46.09198	-0.318091435	1.38302070262e-05	2.04569752725e-05	-
YER083C	115.394753	140.624405	-0.285269366	1.40022720254e-05	2.0706551866e-05	-
YDR003W-A	162.024277	145.105591	0.159106888	1.42416199087e-05	2.10554837802e-05	-
YKR069W	6.285573	9.04037	-0.524337551	1.44872088941e-05	2.14134744796e-05	-
YDR317W	10.469369	8.883761	0.236932006	1.52286719692e-05	2.25040694085e-05	-

YFL066C	5.612331	4.220098	0.411323596	1.55472513751e-05	2.29693804228e-05	-
YMR221C	21.285658	27.67956	-0.378939321	1.56497420025e-05	2.31152982041e-05	-
YOL159C-A	17.154827	11.830087	0.536153893	1.56562740234e-05	2.31194455607e-05	-
YOR297C	28.861874	39.719379	-0.46067809	1.57164658251e-05	2.32028109018e-05	-
YPL214C	27.324318	34.744884	-0.346615077	1.65680280698e-05	2.44541888392e-05	-
YPR066W	23.057152	31.057991	-0.429750188	1.66354339396e-05	2.45478426187e-05	-
YIL163C	3.730927	1.481692	1.332288547	1.68320654635e-05	2.48320965773e-05	up
YER101C	62.983635	63.239983	-0.005859957	1.699132686e-05	2.506109697e-05	-
YLR112W	9.222566	6.063514	0.605014084	1.70162284738e-05	2.50918637448e-05	-
YGL153W	90.003052	90.939072	-0.014926358	1.73041759563e-05	2.55104071092e-05	-
YDR458C	24.960434	31.486246	-0.335078741	1.74168689475e-05	2.56704469197e-05	-
YHR185C	1.236628	0.34758	1.830994601	1.74974357624e-05	2.57830717335e-05	up
YBR098W	8.52713	7.559357	0.173796728	1.7616624884e-05	2.59525408827e-05	-
YLR464W	3.337243	1.768389	0.916221074	1.81350570521e-05	2.6709948797e-05	-
YDR425W	50.247383	61.296852	-0.286764525	1.82046370682e-05	2.68060690037e-05	-
YDL024C	21.922293	20.573322	0.0916239409211	1.8617027045e-05	2.74068067099e-05	-
YMR122C	11.898312	8.155662	0.544883023	1.88687338081e-05	2.77707680135e-05	-
YDL085C-A	24.989758	40.09753	-0.682176439	1.89387806613e-05	2.78672553556e-05	-
YIL045W	132.294983	156.551193	-0.242876151	1.93284613872e-05	2.84339071782e-05	-
YDR144C	2.085402	1.359758	0.616975603	1.98363818625e-05	2.9174190619e-05	-
YPR068C	2.794447	4.683401	-0.744993756	1.98495773861e-05	2.91866831864e-05	-
YLL042C	21.314457	17.654955	0.271759158	2.01009069206e-05	2.95492375474e-05	-

YGL189C	781.095398	917.193909	-0.231728013	2.0208650455e-05	2.97005923354e-05	-
YBR209W	21.607599	16.510756	0.388132591	2.02617022672e-05	2.97715142425e-05	-
YPR051W	25.740011	36.064205	-0.486554951	2.028228647e-05	2.97947076209e-05	-
YGL227W	67.641479	70.528831	-0.060304923	2.03268353035e-05	2.98530857734e-05	-
YNL176C	39.064442	48.154621	-0.301818241	2.0859172639e-05	3.06276593526e-05	-
YCR040W	7.2939	4.82436	0.596352853	2.15470913132e-05	3.16302536941e-05	-
YHR001W	96.373131	98.912277	-0.037518623	2.15753033669e-05	3.16641804495e-05	-
YGR042W	70.134575	87.643967	-0.321528945	2.18899688794e-05	3.21183944228e-05	-
YJR088C	77.4338	95.863121	-0.308012467	2.20102475953e-05	3.22872441096e-05	-
YIL060W	76.753616	99.25843	-0.370954915	2.2016451855e-05	3.22887155955e-05	-
YEL022W	2.985931	2.552873	0.226059059	2.21285018924e-05	3.24453801956e-05	-
YMR253C	46.093529	57.570862	-0.320774587	2.21971344033e-05	3.25383259447e-05	-
YEL004W	14.366419	12.477811	0.203335635	2.22466453293e-05	3.26032044864e-05	-
YPR196W	16.393715	15.017091	0.126537451	2.23968237837e-05	3.2815549221e-05	-
YDL205C	15.503939	13.533482	0.196101725	2.25536871714e-05	3.30375861161e-05	-
YGR275W	114.357758	143.288101	-0.325364568	2.30269829586e-05	3.37229323441e-05	-
YDR127W	21.957794	26.638361	-0.278772199	2.31602484086e-05	3.39100995567e-05	-
YNL048W	20.736721	26.760439	-0.367913998	2.32630728609e-05	3.40526187975e-05	-
YCR030C	26.891417	33.226231	-0.305176873	2.38343642068e-05	3.48806537002e-05	-
YHR015W	4.879342	3.988796	0.290733266	2.42769388145e-05	3.55199708132e-05	-
YPL149W	278.709198	328.239105	-0.23598651	2.43222981652e-05	3.55779516987e-05	-
YIL089W	14.403017	11.567548	0.316287959	2.43879735348e-05	3.56656159492e-05	-

YPL132W	49.20554	47.712395	0.0444566497011	2.49240980767e-05	3.64410741546e-05	-
YHR177W	7.310778	6.002332	0.284501824	2.50052678064e-05	3.65511425812e-05	-
YAL046C	48.647011	43.180283	0.171978466	2.50415329845e-05	3.65955359624e-05	-
YDL154W	2.315068	3.582821	-0.630041397	2.51920708239e-05	3.68068664803e-05	-
YGL126W	28.802235	37.247246	-0.370952993	2.5888085021e-05	3.78148780731e-05	-
YFL056C	14.243233	19.597242	-0.460373979	2.59250131603e-05	3.7859911009e-05	-
YBR028C	14.419644	19.182146	-0.411728584	2.64487905976e-05	3.86157318979e-05	-
YJL072C	22.21553	30.885475	-0.475359958	2.65902693927e-05	3.88131653868e-05	-
YER175C	79.334831	97.86898	-0.302897261	2.66843289221e-05	3.89413055826e-05	-
YHR005C-A	386.549805	467.839142	-0.27535826	2.81470504337e-05	4.10662489054e-05	-
YBR161W	11.259507	9.623922	0.226446805	2.82447281202e-05	4.11990771077e-05	-
YHL022C	6.736262	10.028443	-0.574077476	2.83037066528e-05	4.12754077756e-05	-
YHR215W	16.92889	15.566732	0.121021277	2.83487072642e-05	4.13313233199e-05	-
YMR013C	3.701331	5.788696	-0.645194236	2.85325957025e-05	4.15896581641e-05	-
YHR126C	3.137672	6.54735	-1.061216556	2.85537285706e-05	4.16106917762e-05	down
YJL062W-A	365.098083	444.488495	-0.283861984	2.86644657163e-05	4.17622631229e-05	-
YGL141W	24.986322	30.87928	-0.305500563	2.94228146963e-05	4.28570703038e-05	-
YOR147W	120.942001	126.869034	-0.069024627	2.94618953229e-05	4.29039282582e-05	-
YML002W	7.178473	6.317477	0.184328481	2.95746363807e-05	4.30580071884e-05	-
YGR222W	14.382465	12.289999	0.226826161	3.07047954227e-05	4.46929355179e-05	-
YLR390W	47.163368	64.730354	-0.456775647	3.07491711979e-05	4.47470358172e-05	-
YLR173W	51.691277	52.44606	-0.020913551	3.08094575757e-05	4.48242588774e-05	-

YCR060W	37.892418	32.350124	0.228137962	3.13993731158e-05	4.56718154412e-05	-
YIL118W	36.494835	48.061958	-0.397203125	3.16733843109e-05	4.60595853367e-05	-
YDR449C	13.902287	18.817467	-0.436750208	3.17388705903e-05	4.61440067037e-05	-
YJR079W	1.812765	4.991574	-1.461302903	3.25312542729e-05	4.72849511886e-05	down
YDR528W	3.975535	2.852417	0.478964114	3.36226201394e-05	4.88598375059e-05	-
YJL214W	2.281634	1.537233	0.569731533	3.4123472621e-05	4.95760631947e-05	-
YCL066W	6.911493	4.515109	0.614236574	3.44881478145e-05	5.00941557399e-05	-
YJL073W	5.20962	4.29923	0.27709985	3.52227600224e-05	5.11492150221e-05	-
YIL072W	5.213244	4.268509	0.288449164	3.77962577692e-05	5.48735192308e-05	-
YJL222W	1.731158	1.381456	0.325547791	3.7986986734e-05	5.51375294937e-05	-
YGL051W	18.307322	15.742069	0.2177956	3.82520404659e-05	5.55092723731e-05	-
YIL128W	4.883649	6.730376	-0.462727591	3.86423830752e-05	5.60626113884e-05	-
YPL057C	628.93689	723.173157	-0.20142587	3.86434893361e-05	5.60511172427e-05	-
YDR453C	112.33297	112.040215	0.00376476740188	3.86547218337e-05	5.6054312811e-05	-
YGL036W	15.548698	19.702473	-0.341582946	3.93457276468e-05	5.7043035318e-05	-
YOL102C	40.578999	52.909908	-0.382804632	3.93864957753e-05	5.7088808259e-05	-
YML006C	9.85108	13.051907	-0.405906808	3.93945433606e-05	5.70871440669e-05	-
YGL249W	4.746493	3.932576	0.271387308	3.95141182018e-05	5.72470585291e-05	-
YML083C	6.546471	5.255239	0.316961027	4.10949727504e-05	5.952346963e-05	-
YIR018C-A	2.431061	8.602982	-1.823250725	4.1507614437e-05	6.01071309599e-05	down
YGL178W	10.506013	9.831276	0.0957646937502	4.27329175294e-05	6.18670597068e-05	-
YHR022C	3.418809	6.138546	-0.844403148	4.29428535252e-05	6.21565014419e-05	-

YMR065W	13.224502	12.008401	0.139169336	4.32282236925e-05	6.25549679914e-05	-
YHR076W	55.561375	54.987167	0.0149873434629	4.47734166416e-05	6.47758961806e-05	-
YNR030W	11.035604	14.907903	-0.433911744	4.54935619537e-05	6.58024307103e-05	-
YMR284W	32.152973	31.867798	0.012852806	4.60273444429e-05	6.65589923833e-05	-
YOL028C	7.421226	11.58086	-0.642012945	4.61180324799e-05	6.66746030823e-05	-
YNL131W	489.265045	576.608154	-0.236975024	4.63361035087e-05	6.69742795302e-05	-
YFR002W	17.935863	22.604382	-0.333755312	4.90840227729e-05	7.09296120517e-05	-
Q0020	1.377897	0.778582	0.823547152	4.91590486848e-05	7.10214973784e-05	-
YJR024C	38.453671	36.195503	0.0873108672131	4.98721914214e-05	7.20350312574e-05	-
YBL061C	14.114909	13.323967	0.0831961416928	5.00690572932e-05	7.23025605201e-05	-
YOR290C	34.560852	41.006474	-0.246712913	5.0389081011e-05	7.27477709108e-05	-
YER051W	32.902275	41.280655	-0.327278521	5.24299991958e-05	7.56766879812e-05	-
YBR238C	2.153684	3.446568	-0.678353894	5.3375802751e-05	7.70239385119e-05	-
YDR073W	76.63607	74.065628	0.0492194003846	5.42520474029e-05	7.82702092208e-05	-
YKL078W	1.858231	3.036319	-0.708393513	5.4458810696e-05	7.85502548329e-05	-
YGL094C	12.801051	16.168789	-0.336949365	5.72798550012e-05	8.26000789424e-05	-
YMR318C	40.840435	51.499821	-0.334569185	5.76017224764e-05	8.30449357021e-05	-
YDL211C	2.749983	1.75404	0.648741052	5.7846869183e-05	8.3379002528e-05	-
YHR034C	23.038513	21.368616	0.108554132	6.09741199598e-05	8.78661412977e-05	-
YOR287C	6.1079	4.569553	0.418623396	6.0981514597e-05	8.78564034854e-05	-
YDR218C	5.125307	7.774728	-0.60115378	6.1060356704e-05	8.79495810715e-05	-
YHR176W	16.484777	21.882284	-0.408628959	6.14568260591e-05	8.85001104558e-05	-

YPL067C	22.461388	19.532764	0.201550968	6.14779665703e-05	8.85100223721e-05	-
YOR216C	11.352501	10.144393	0.162327621	6.17623223942e-05	8.88987937452e-05	-
YDR185C	18.039219	15.019242	0.264324875	6.28297473538e-05	9.04142493214e-05	-
YPL033C	0.53837	1.613513	-1.583535277	6.44714300966e-05	9.27551884217e-05	down
YCR105W	8.690816	12.539526	-0.528919268	6.5501048163e-05	9.42146679787e-05	-
YBR049C	43.289246	52.243484	-0.271242438	6.68956645747e-05	9.61983520222e-05	-
YFL001W	9.036618	12.664413	-0.486925366	6.73815551045e-05	9.68746396686e-05	-
YJL097W	22.173223	19.501987	0.185197366	6.83855110902e-05	9.82952657671e-05	-
YDL006W	47.874714	46.491539	0.0422956846208	6.94113544961e-05	9.97466872018e-05	-
YNL307C	66.06131	80.894753	-0.292240551	7.09061778606e-05	0.000101871222439	-
YDL237W	126.622894	150.875839	-0.252823519	7.11791112354e-05	0.000102239685921	-
YOR217W	13.095912	12.558188	0.0604882173607	7.19346319881e-05	0.000103300993611	-
YJR137C	4.56157	4.166889	0.130559787	7.22856144842e-05	0.000103781011729	-
YFL019C	24.044767	19.615623	0.293719789	7.23698300335e-05	0.000103877897075	-
YOR368W	8.877141	7.517103	0.239918341	7.25541876669e-05	0.000104118445917	-
YNL018C	2.136274	3.490983	-0.708536631	7.32132858126e-05	0.000105039999613	-
YNL264C	22.598839	29.673237	-0.392913659	7.43014774187e-05	0.000106576610863	-
YPR085C	29.523336	37.401794	-0.341251723	7.60687054905e-05	0.000109086284057	-
YML023C	10.896899	9.874426	0.142148845	7.62565773651e-05	0.000109330446255	-
YCR042C	6.81599	6.450757	0.0794547473787	7.65442620688e-05	0.000109717566133	-
YPL008W	7.963791	7.309653	0.123652439	7.65889165839e-05	0.000109756231337	-
YMR087W	25.698687	23.716862	0.115781513	7.74759368146e-05	0.000111001757615	-

YJL030W	37.977859	35.166164	0.110970605	8.0826852156e-05	0.000115775980199	-
YDR313C	93.382378	113.871338	-0.286182426	8.0936411991 9e-05	0.000115906169699	-
YDR062W	43.246685	52.975811	-0.292744219	8.0963332996 8e-05	0.000115917982298	-
YCL036W	1.441704	2.563974	-0.830606641	8.2854785173e-05	0.000118598687193	-
YGL017W	26.227125	33.185974	-0.339513949	8.4324064592 9e-05	0.000120673995618	-
YER180C	30.890192	40.400772	-0.387234023	8.4460419827e-05	0.000120841273631	-
YNL280C	41.252377	40.889801	0.0127362095113	8.4713188246 6e-05	0.000121174993695	-
YMR313C	74.854576	89.203278	-0.253006213	8.5331447797 8e-05	0.000122031243476	-
YKL108W	9.998022	8.7635	0.190135528	8.5562460332e-05	0.000122333430157	-
YKL143W	34.04454	42.619614	-0.324094087	8.6951719211 9e-05	0.000124291105887	-
YPL216W	6.82116	6.323383	0.109320499	8.7742498294 4e-05	0.000125392594248	-
YHL048W	74.370064	75.532936	-0.022383853	8.8273216352 4e-05	0.000126122008542	-
YBR187W	22.496593	30.025356	-0.416474823	8.8909270785 2e-05	0.000127001553851	-
YOR340C	7.282657	10.823401	-0.5716171	8.9161260035 8e-05	0.000127332206649	-
YBR048W	160.924332	196.692093	-0.289556482	9.0149016020 2e-05	0.000128713222505	-
YGR292W	12.782289	11.849635	0.10930359	9.0678479342 4e-05	0.000129439411303	-
YCR033W	29.322817	35.303635	-0.267793029	9.0725791279 1e-05	0.000129477175232	-
YGR195W	28.620684	37.869339	-0.403972088	9.1047242586 5e-05	0.000129906063428	-
YOL056W	6.390786	9.769635	-0.612311285	9.2323442345 2e-05	0.00013169667511	-
YGR134W	10.453105	13.31987	-0.349648457	9.3535590405 3e-05	0.000133395117215	-
YMR048W	8.988159	13.023201	-0.534986544	9.3684274502 3e-05	0.00013357647591	-
YBL059W	56.979267	72.898323	-0.355448564	9.3903978110 7e-05	0.000133858988774	-

YDR163W	81.932549	80.111	0.032436347	9.471891467e-05	0.000134989674534	-
YGR017W	57.491108	56.865986	0.0157728630724	9.55436204327e-05	0.000136133760763	-
YPL249C-A	570.591858	675.983765	-0.244529437	9.60211754416e-05	0.000136782803383	-
YDL135C	106.912231	107.158478	-0.003319085	9.62082200694e-05	0.000137017809174	-
YER007C-A	55.951355	53.336422	0.0690520231057	9.72078535433e-05	0.000138409714403	-
YMR188C	102.027138	124.863518	-0.291399076	9.82517728017e-05	0.000139864023287	-
YPL176C	23.499228	23.341217	0.0097335775024	9.96917049478e-05	0.000141881271049	-
YCR044C	4.752864	3.534425	0.427321592	0.000100654839598	0.000143219171264	-
YNL034W	2.370253	1.694621	0.484078408	0.000100791414409	0.00014338063718	-
YOL152W	8.226962	7.333945	0.165770333	0.00010167249617	0.000144600883442	-
YJR028W	21.973316	20.875364	0.07395125	0.000102370571787	0.000145560354937	-
YDR249C	4.594141	7.151675	-0.638486041	0.000103876595053	0.000147667941858	-
YER094C	315.969543	331.441772	-0.068969938	0.000104621672369	0.000148693072817	-
YDL146W	11.237159	10.109563	0.152556701	0.000105675186601	0.000150155998722	-
YIL121W	5.393822	4.450254	0.277420232	0.000105702683045	0.000150160699392	-
YGL151W	9.287688	8.88916	0.0632724137926	0.000106885599857	0.000151806406752	-
YER058W	27.860432	40.079014	-0.524629388	0.000108540629552	0.000154121735649	-
YMR025W	15.315576	13.490902	0.183012816	0.000110047420848	0.000156225563372	-
YGL174W	22.95908	30.667496	-0.417645543	0.000110584745975	0.000156952469824	-
YHR195W	171.73645	203.577835	-0.245384218	0.000110955395262	0.000157442535722	-
YER071C	54.396935	50.1768	0.116504898	0.000111205018283	0.000157760684073	-
YNL070W	222.060089	216.605072	0.0358830943667	0.000115631791646	0.000164003235672	-

snR3	9.927418	5.470027	0.859870585	0.000119682407901	0.000169709545054	-
YDR447C	172.050415	210.564728	-0.291432417	0.000119822490109	0.000169869380816	-
YDR078C	16.935026	14.566293	0.217376432	0.000123152752876	0.000174550751108	-
YIL110W	8.936965	12.646251	-0.50085288	0.000124199110195	0.000175993626133	-
YCR098C	3.321014	5.140831	-0.630377781	0.000125231068916	0.000177415444051	-
YBL081W	3.423212	5.58038	-0.705012725	0.000126613846642	0.00017933350672	-
YHR027C	92.769569	108.398422	-0.224620211	0.000127175945169	0.000180088564691	-
YDR539W	25.617363	32.353584	-0.336803562	0.000129825506614	0.00018379857356	-
YKR079C	3.182542	2.599419	0.291990353	0.000131152668946	0.00018563515021	-
YGR246C	41.341625	50.46244	-0.287614869	0.000131908095103	0.000186661831411	-
YDR280W	31.410093	40.434689	-0.364365301	0.000133282774097	0.000188564143481	-
YJL080C	10.032075	9.730363	0.0440545067434	0.000133668809347	0.000189067206295	-
YGL083W	13.795591	13.282952	0.054631456	0.000133992743537	0.000189482221385	-
YKL186C	323.656799	382.467773	-0.24087338	0.000134645134644	0.000190361420149	-
YGR048W	92.83062	111.858551	-0.269002888	0.000135088332608	0.000190944528423	-
YDL079C	135.111099	142.052505	-0.07227808	0.000139980730497	0.000197814790559	-
YLR300W	163.889221	172.738647	-0.075869923	0.000142534306765	0.000201377554938	-
YBR200W-A	5.856193	13.062236	-1.157366873	0.000143215519126	0.000202293957391	down
YEL041W	14.750058	13.730242	0.103363574	0.000143848670994	0.000203142072232	-
YBL091C-A	34.162235	31.145826	0.13336353	0.000145888268777	0.00020597552253	-
YNL196C	9.48809	7.875949	0.268663926	0.000150396888094	0.000212292833399	-
YPL098C	258.925537	264.225372	-0.029231746	0.00015239176474	0.00021505980348	-

YBR024W	51.356827	50.642391	0.02021055	0.000154507740979	0.000217996376364	-
YDR376W	22.459316	21.651787	0.0528278903455	0.000155042370105	0.000218700984688	-
YLR289W	11.027594	14.495731	-0.39451003	0.000159854120629	0.000225437160579	-
YGR045C	14.68671	11.120446	0.401296601	0.000160210855017	0.000225888936621	-
YIL016W	41.472103	38.261646	0.116242267	0.000165081098363	0.00023270287435	-
YMR027W	284.563843	304.376892	-0.097106472	0.00016703454889	0.000235403060048	-
YMR090W	309.658844	326.240753	-0.07525736	0.000172864449529	0.000243563890757	-
YHL050C	15.497426	14.932428	0.05357985	0.000172893573541	0.000243549649318	-
YMR239C	6.629547	9.416656	-0.506304534	0.000173449731565	0.000244277661878	-
YML056C	4.3881	6.474497	-0.561171711	0.000173782192748	0.000244690372551	-
YLR331C	38.069225	33.949619	0.165229816	0.000174127752018	0.00024512133436	-
YGR294W	2.575933	5.748825	-1.158172045	0.000174154925469	0.000245104007552	down
YMR278W	54.446785	55.960861	-0.039571297	0.000178685803612	0.000251423723668	-
YLR211C	52.78619	51.251141	0.0425764153896	0.000181695482976	0.000255600625043	-
YDL058W	4.146804	3.873294	0.0984388470161	0.000187652119169	0.000263920334345	-
YLR115W	12.851694	12.421363	0.0491350460139	0.000189679826189	0.000266711746542	-
YGL093W	24.252184	24.461405	-0.012392598	0.000201499815736	0.000283267856905	-
YIL173W	1.514937	1.224393	0.307191097	0.00020884897213	0.000293532809369	-
YPR055W	9.894037	12.606661	-0.349555014	0.000211836272513	0.000297664006283	-
snR60	8.516985	3.189335	1.417087166	0.000216077235393	0.000303554532093	up
YKL214C	47.648411	61.020878	-0.356874835	0.000217641697761	0.000305683180927	-
YGL213C	15.551501	20.601963	-0.405727974	0.000221221160885	0.000310640345346	-

YDL139C	20.916645	28.394728	-0.440971628	0.000233340900452	0.000327584873361	-
YOR010C	10.481932	8.679856	0.272161641	0.000236158472195	0.000331465474879	-
YPL058C	5.588799	5.301084	0.0762508882335	0.00024421322652	0.000342693424556	-
YDL066W	124.991501	130.79985	-0.065530887	0.00024772403982	0.000347541432588	-
YLR067C	8.465715	10.960788	-0.372647695	0.000247823191735	0.000347601982442	-
YKL137W	115.909897	113.494064	0.0303869133081	0.000248854801596	0.00034897009449	-
YLR162W-A	2.285887	6.485113	-1.504377626	0.000251065936798	0.000351991268212	down
YPL162C	57.710453	57.233498	0.0119728712389	0.000252480717676	0.000353894851058	-
YCR024C-A	548.177734	669.560181	-0.288570003	0.000253037339344	0.000354594989311	-
YGL124C	16.749886	16.210711	0.0472039079088	0.000255840548914	0.000358442366883	-
YML111W	6.157365	8.203159	-0.4138665	0.000258998635577	0.000362785092432	-
YPL129W	107.887062	130.79126	-0.277744273	0.000260274630828	0.000364490166519	-
YOR308C	14.622805	13.901925	0.0729354140725	0.00026842523123	0.000375819538899	-
YIL119C	27.984509	27.176352	0.0422766253953	0.000269300498553	0.000376959976329	-
YDR121W	76.264076	94.928581	-0.315838879	0.00027055575424	0.0003786316 7771	-
YLR059C	36.049908	46.056343	-0.353404284	0.000272200422239	0.000380847469295	-
YJL077W-B	17.178699	9.36766	0.874860161	0.0002739382 5095	0.000383192578165	-
YJR030C	1.945602	1.445915	0.428233873	0.000276610808772	0.000386843861423	-
YBR284W	5.109167	7.00435	-0.455163083	0.000282520474039	0.000395019617756	-
YOR334W	3.682382	5.607783	-0.606791228	0.000283499530689	0.000396299276406	-
YJR017C	168.32074	203.200378	-0.271690133	0.000285210724078	0.000398601570256	-
YDR014W-A	6.319397	4.277434	0.563041308	0.000285607902913	0.000399066815504	-

YOR250C	12.752945	11.688393	0.125753851	0.000288453813429	0.000402952581856	-
YAL003W	702.274475	810.881775	-0.207456588	0.000298995524478	0.000417584750497	-
YOR155C	42.987831	43.19809	-0.007039206	0.000301812146005	0.000421423707243	-
YBR259W	6.424796	8.737329	-0.443541669	0.000301970392995	0.00042154985377	-
YPR202W	7.922743	6.209623	0.351494323	0.000308353475204	0.000430363843091	-
snR65	1.151165	6.29794	-2.451785379	0.000312479987449	0.00043602512072	down
YLR125W	20.285301	16.98921	0.255815941	0.000323702567432	0.000451583267105	-
YDR361C	50.130463	62.534538	-0.318965656	0.000324919515379	0.00045317913985	-
YLR296W	1.739483	0.454021	1.937827646	0.000325667845424	0.000454120841058	up
YBR130C	15.28968	20.085321	-0.393583306	0.000328997055879	0.00045866016683	-
YNL162W-A	11.407593	19.604244	-0.781171593	0.000333877016331	0.000465358894787	-
YDL009C	21.246029	30.962221	-0.543315745	0.00033746463988	0.000470253756314	-
YOR189W	93.63253	118.538651	-0.340275798	0.000341820294938	0.000476216425263	-
YPR071W	10.514939	15.387085	-0.549279466	0.000342006503406	0.000476368941697	-
YAR061W	0.95207	0.385076	1.305924441	0.000342057366346	0.000476332913925	up
YGL170C	1.457262	0.818148	0.832826532	0.000345608813526	0.000481170557159	-
YLR415C	14.170158	21.710825	-0.615558704	0.000353364593066	0.000491858160035	-
YPL037C	289.448334	343.474854	-0.24689863	0.000353413505394	0.000491815969847	-
YHL010C	19.285892	18.769695	0.0391406670676	0.000354395062035	0.000493071390658	-
YLR106C	7.399745	7.653678	-0.048677651	0.000359392045511	0.000499911677914	-
YBR146W	89.280891	108.29422	-0.278532914	0.000360780371123	0.000501730408587	-
YDR253C	7.999311	6.037529	0.405917529	0.000369894164992	0.000514289580352	-

YGL007W	29.94042	41.254036	-0.46244081	0.000377275362536	0.000524434718008	-
YIL129C	10.737434	13.005661	-0.276490461	0.000380576493392	0.000528905052827	-
YJR132W	4.979056	6.64836	-0.417126263	0.000381584711722	0.000530187531416	-
YER145C-A	30.63575	41.500145	-0.437900207	0.000384102177313	0.000533565969291	-
YML022W	43.365215	55.881565	-0.365834163	0.00038771917124	0.000538469936255	-
YGR273C	0.084119	0.72947	-3.116345043	0.000388992266242	0.000540117197234	down
YDR439W	22.987114	29.653347	-0.367369603	0.000390330645881	0.000541854349201	-
YGL247W	15.682336	22.052143	-0.491778386	0.000391592170148	0.000543484058188	-
YNL313C	6.763369	8.910244	-0.397722872	0.000392714505299	0.000544919903642	-
YDR399W	154.086517	184.498703	-0.259870047	0.000395766778264	0.000549032437868	-
YHL036W	22.083857	21.628382	0.0300664217582	0.000397530955453	0.00055135660667	-
YNL279W	8.364751	11.09875	-0.408002702	0.000403885215257	0.000560044542398	-
snR61	8.598568	3.02928	1.50512148	0.000405193628921	0.000561733373904	up
YIL148W	386.516418	404.442108	-0.065403513	0.000408228728089	0.000565814678271	-
YDR158W	67.676956	82.043106	-0.277717431	0.000408684111466	0.000566319411603	-
YGR278W	11.796572	11.05225	0.0940275824149	0.000409075359031	0.00056673506558	-
YMR156C	48.734802	47.629135	0.033108035	0.000416408515294	0.000576765743629	-
YLR245C	40.014088	52.944626	-0.403976227	0.000420220500873	0.000581915875401	-
YLR104W	32.459679	28.992966	0.162945803	0.000423226344286	0.000585947623846	-
YGL240W	15.461898	21.19964	-0.45532234	0.000428716565312	0.000593416374015	-
YFL033C	6.743434	8.47287	-0.329367283	0.000434575785067	0.000601392437292	-
YJL127C-B	278.839264	276.74472	0.0108779325715	0.000438429083226	0.000606589647574	-

YGR185C	35.027126	34.636639	0.0161736753286	0.000444556306959	0.000614929936186	-
YMR254C	17.557741	13.733203	0.354439093	0.000446425423934	0.000617377819511	-
YDL119C	18.692198	17.247484	0.116050302	0.000448113088323	0.000619573731026	-
YPL104W	9.454879	12.412338	-0.392643988	0.000460324960295	0.000636316489315	-
YOR242C	2.178305	1.385961	0.652319309	0.000464460867909	0.000641890709701	-
YLR242C	4.666608	3.520902	0.406429212	0.000466261545615	0.000644235850251	-
YAL059W	26.935102	35.612228	-0.402885169	0.000466841731573	0.000644893962974	-
YDL022C-A	7.824183	4.663872	0.74641192	0.00046934313479	0.000648205157013	-
YMR209C	11.214896	14.965452	-0.416219611	0.000470845521421	0.000650135453065	-
YMR142C	334.347595	391.96228	-0.229366085	0.000479197502407	0.000661520590381	-
YOR034C-A	8.442334	14.952001	-0.824624758	0.000486814614563	0.000671886422233	-
YNR036C	592.111023	630.152649	-0.089833639	0.000487796862418	0.000673092447631	-
YJR083C	33.685566	32.730083	0.0415132794304	0.000489203762696	0.000674883768626	-
YPL140C	31.807425	31.759722	0.00216529756965	0.000490435898956	0.000676433250548	-
YDL113C	159.975021	185.684952	-0.215010248	0.000498412479717	0.000687282246575	-
YPL245W	1.436489	2.558705	-0.832866879	0.000498471745226	0.000687211324531	-
YMR192W	13.404346	17.035799	-0.345868781	0.000503695791434	0.000694259208087	-
YKL128C	16.143784	21.643694	-0.422967973	0.000510077860853	0.000702899746986	-
YLR004C	8.512111	11.476137	-0.431048227	0.000515091459709	0.000709651083416	-
YMR202W	132.527695	159.329208	-0.265716885	0.00051612263911	0.000710913987929	-
YGR084C	21.606636	27.980865	-0.37296609	0.000528978144642	0.000728459698743	-
YNL204C	3.974609	2.872926	0.46829211	0.000532189539659	0.000732719596852	-

YGR161W-C	710.919067	750.872192	-0.078882035	0.000533032849468	0.000733717944456	-
YHR160C	50.957211	63.206215	-0.310780106	0.000544633164859	0.000749519549422	-
YGR101W	27.426741	34.85796	-0.345904938	0.000547741413543	0.000753630029981	-
YOL105C	12.011131	11.286802	0.0897352343224	0.000556351480821	0.000765306889639	-
YBL074C	8.67616	7.486581	0.212749645	0.000556385625493	0.00076518430728	-
YER087C-B	144.845932	142.186539	0.0267342776174	0.000558587888252	0.000768042881565	-
YPL121C	0.526978	0.049472	3.413058605	0.000559943384281	0.000769736166877	up
YBL020W	16.699589	21.199549	-0.344220977	0.00056530583375	0.000776935713066	-
YOR130C	13.139674	17.927969	-0.448282577	0.000570713847863	0.000784194680729	-
YLR315W	15.719049	13.109344	0.261918443	0.000572804166057	0.000786892733543	-
YCR063W	6.583354	4.611048	0.513728092	0.000584417312518	0.000802668733653	-
YGL251C	8.605996	8.367994	0.0404603525856	0.000584573140114	0.000802705165634	-
YLR118C	107.527092	109.750465	-0.029526852	0.00058839678314	0.000807776919446	-
ETS2-1	9.233368	16.319328	-0.821652759	0.000593221882965	0.000814220970473	-
YNL042W-B	6.23488	3.488075	0.837930732	0.000593363774567	0.000814235701263	-
YGR254W	1700.049683	1922.130127	-0.1771291	0.000594798980974	0.00081602476771	-
YOR052C	743.993835	861.521362	-0.211595903	0.000602374869733	0.000826235791273	-
YHL013C	22.75746	29.511917	-0.374958092	0.000610145080596	0.000836708782933	-
YML054C-A	21.230322	15.092639	0.492281165	0.000623727690435	0.00085514609148	-
YHL005C	6.981694	11.509622	-0.721191421	0.000628072927739	0.000860913388254	-
YLR455W	31.031555	30.027044	0.0474735361693	0.00064711374197	0.000886817242858	-
YLR091W	30.156393	38.437931	-0.350066804	0.000657644342461	0.000901049675126	-

YOR012W	7.630269	12.274663	-0.685877592	0.000668924227348	0.000916302207276	-
YNL242W	13.793142	14.028269	-0.024385868	0.000680722343035	0.000932257733413	-
YJL096W	77.357071	96.538513	-0.319571434	0.000684612705366	0.000937378843165	-
YNR008W	14.146325	17.95928	-0.344302201	0.000686471569895	0.000939716759848	-
YCR020C	5.535285	4.040728	0.45404236	0.000687812674743	0.000941345036333	-
YIL063C	51.686527	51.876236	-0.00528554	0.0006893895122	0.000943295149159	-
YGR071C	10.221621	13.029525	-0.350160486	0.000690545028318	0.000944668033451	-
YLR167W	769.05658	889.358948	-0.209676071	0.000698241261213	0.000954986065127	-
snR30	17.118368	23.555632	-0.460526873	0.000698830584848	0.000955581557431	-
YPR162C	27.794493	27.707045	0.00454621276744	0.000701322436019	0.000958777732395	-
YPL144W	32.39756	29.596699	0.130448884	0.000701528008101	0.000958847616532	-
YMR138W	44.900665	43.177723	0.0564496480354	0.000702278088191	0.000959661538959	-
YJR131W	17.639414	17.114649	0.0435709290514	0.000704867842158	0.000962988460413	-
YPL040C	8.878796	11.305636	-0.348606194	0.000717910357794	0.000980591309391	-
YMR226C	424.430725	491.037109	-0.210302959	0.00072846954195	0.000994795186191	-
YOR336W	5.886514	7.545589	-0.358219999	0.000731973539556	0.000999360398848	-
YKR014C	463.867523	496.313873	-0.09753994	0.000735606725346	0.00100409994524	-
YJL181W	6.376277	5.685505	0.165425806	0.000737780068168	0.00100684516667	-
YNR068C	12.668676	11.15463	0.183623097	0.000740749272978	0.00101067505201	-
snR44	20.253275	30.999445	-0.614087172	0.000744694814717	0.00101583507136	-
YPL028W	218.069046	232.202606	-0.090599164	0.000752483328377	0.00102623385381	-
YDL212W	119.875946	144.663544	-0.2711592	0.000753158770055	0.00102692941895	-

YLR119W	50.669964	49.561554	0.0319093800375	0.000755784309395	0.00103028304627	-
YCR020C-A	42.889286	38.204895	0.166859804	0.000767277996267	0.00104572158086	-
YGR126W	14.180164	19.594206	-0.466552895	0.000778933438798	0.00106137374628	-
YOL163W	7.772281	5.865149	0.406170302	0.000779676374548	0.00106215293684	-
YAL065C	8.88572	6.465906	0.458636147	0.000783242775821	0.00106677734802	-
YLR238W	17.928616	17.268166	0.054149257	0.000802282883789	0.00109247030984	-
YNL269W	5.396503	3.433417	0.652379738	0.000803849965246	0.00109436416321	-
YDL188C	32.196552	40.185688	-0.319775587	0.000804243192496	0.00109465944727	-
YNL011C	6.900019	5.987369	0.204678149	0.000810375749569	0.00110276472015	-
YOR158W	56.571003	69.197853	-0.290664526	0.000816396504209	0.00111071433227	-
YEL020W-A	244.403351	248.551208	-0.024279049	0.000821475176912	0.00111737903117	-
YKL121W	16.482952	16.411537	0.00626428507334	0.00082643863176	0.00112388412398	-
YHR113W	85.103577	100.842476	-0.24481177	0.000839548421857	0.0011414622433	-
YEL013W	30.527685	30.777559	-0.011760624	0.000848059579311	0.00115278166594	-
YML129C	548.567322	571.468689	-0.059005771	0.000858242719897	0.00116636838991	-
YNL314W	53.255283	66.010818	-0.309777826	0.000866883529324	0.00117785356753	-
YGR012W	34.756474	43.067986	-0.30933413	0.000878093946504	0.00119282433696	-
YLR126C	45.763504	45.048222	0.0227273542928	0.000887681991571	0.00120558516816	-
YJL200C	6.493601	8.585055	-0.402808636	0.000890469421442	0.00120910633603	-
YJL070C	11.956504	15.019357	-0.329027433	0.00089331168697	0.00121270040514	-
YHR082C	56.246368	59.332653	-0.077066353	0.000895019859981	0.00121475367091	-
YOL042W	28.001154	35.270565	-0.332978401	0.000895738340138	0.00121546308537	-

YGL067W	7.87708	10.925828	-0.472009784	0.000897446738493	0.00121751515572	-
YPL120W	18.170046	17.73772	0.0347414938931	0.000913110516706	0.00123849466631	-
YKL106W	10.375432	13.819158	-0.413498308	0.000921728364697	0.0012499103731	-
YLR385C	5.003957	3.154325	0.665738079	0.000927288503805	0.00125717559109	-
YGL110C	21.90954	27.138451	-0.308779183	0.0009308133056	0.00126167882121	-
YGL224C	14.693359	13.26424	0.147622227	0.000939380532969	0.00127301339198	-
YOL131W	2.848718	1.243913	1.195427228	0.000941502000581	0.00127547068622	up
YFL015C	1.891694	0.823781	1.199345981	0.000941502000581	0.00127547068622	up
YER093C-A	51.633652	66.574394	-0.366655749	0.000944387690168	0.00127896133956	-
YLL006W	50.430782	51.38158	-0.026946658	0.000951302609385	0.00128804505977	-
YKR071C	208.146698	220.920547	-0.085926933	0.000952590697442	0.00128950786082	-
YDR255C	85.001549	88.375343	-0.056154777	0.000957447326815	0.00129579965225	-
YDR174W	290.315155	308.033539	-0.085467556	0.000958971007031	0.00129757890402	-
YLL060C	38.790714	37.606205	0.0447406057211	0.000962292252632	0.00130178912712	-
YJR160C	5.264976	7.265203	-0.464576155	0.000966601938459	0.00130733438648	-
YHL015W-A	28.433878	18.688639	0.605449368	0.000967550095641	0.00130833173464	-
YJL061W	12.184225	11.781575	0.0484820723966	0.000985126844199	0.00133180911341	-
YKR053C	15.120235	19.69504	-0.381351785	0.000998708569508	0.00134987650762	-
tC(GCA)P2	4.645223	0.595171	2.964371719	0.00100818574177	0.00136238943947	up
YMR074C	76.055664	95.225182	-0.324287448	0.00100904152756	0.00136324914105	-
YDR301W	6.20072	5.977233	0.0529579626388	0.00101615917247	0.00137256661068	-
YLR256W-A	16.059616	15.284693	0.0713498217885	0.0010222139716	0.00138044471083	-

YBL040C	68.459419	68.539673	-0.00169026	0.00102349698514	0.00138187674723	-
YNL155W	53.428394	53.424599	0.000102477759935	0.00104141184891	0.00140575880801	-
YOL023W	12.144604	15.478697	-0.349968582	0.00105172888553	0.00141937672203	-
YHR011W	11.2317	14.835022	-0.40143076	0.00107787276334	0.00145434342856	-
YBL100W-B	6.130968	7.682447	-0.325451034	0.00108036217051	0.00145738556161	-
YKR104W	1.852964	1.120762	0.725354906	0.00108328246667	0.00146100750665	-
YCR050C	6.20562	10.83948	-0.804648287	0.00109657696706	0.00147861637956	-
YBR135W	35.175831	46.255562	-0.395042347	0.00109872578786	0.00148119211532	-
YHR002W	31.354979	39.190807	-0.321820727	0.00110978994527	0.00149578288759	-
YIL102C-A	38.750507	53.794777	-0.47325092	0.00111496882492	0.00150243682767	-
YLR307W	8.837895	7.578537	0.221783421	0.00112502767495	0.00151566228431	-
YKL069W	113.564682	115.564415	-0.025182992	0.00112569208103	0.00151622834433	-
YEL053C	7.208835	6.701966	0.105181761	0.00113480854361	0.00152817601708	-
YER183C	23.601614	31.152178	-0.400447511	0.00116071753034	0.00156272705017	-
RNA170	15.040453	24.713505	-0.716451616	0.00120713974824	0.00162487501237	-
YNL118C	78.261314	83.236244	-0.088912532	0.00121803832262	0.00163918966114	-
YGL209W	26.519176	33.300014	-0.328486835	0.00122546060292	0.00164882085456	-
YIL108W	28.536694	29.024235	-0.024439835	0.00123763901483	0.00166484572136	-
YOR103C	70.800568	68.897598	0.0393072477788	0.00124344116972	0.00167228829758	-
YHR197W	3.287768	4.632529	-0.494691508	0.00125087829347	0.00168192602249	-
YJR159W	14.648269	19.232693	-0.392830596	0.00125251743094	0.00168376531209	-
YCR006C	32.483532	42.714573	-0.395019852	0.00126468959681	0.00169976034142	-

YAL037W	5.846199	4.611194	0.342358575	0.0012890572323	0.00173213577882	-
YIL004C	36.866791	34.269741	0.105386557	0.00130186635973	0.00174896913248	-
YLR388W	89.246643	82.908577	0.106276545	0.00132791148823	0.00178357302443	-
YGL002W	112.742821	115.748985	-0.037963971	0.00133759566083	0.00179619162069	-
YPR179C	19.322588	23.95389	-0.309971625	0.00135485957387	0.00181898101959	-
YJL141C	311.553802	338.407776	-0.119281397	0.00136940176471	0.00183810727683	-
YLR205C	234.903793	273.51236	-0.219536021	0.00138222396876	0.00185491707697	-
YGL225W	30.046644	37.678711	-0.32654575	0.00139237370373	0.00186813398589	-
YLR352W	7.224111	6.799204	0.0874542040113	0.00140143651238	0.00187988717996	-
YGL019W	104.379272	107.963654	-0.048710465	0.00141264430746	0.00189451195954	-
YCL038C	12.691684	12.093001	0.0697111979927	0.0014516036458	0.00194634026633	-
YOR115C	39.14431	38.513317	0.0234452311121	0.00147093361566	0.00197183240898	-
YBR019C	18.744875	23.171341	-0.305845335	0.00147156510291	0.00197225305676	-
YOR125C	81.179214	98.644333	-0.281125801	0.00147180248625	0.00197214544239	-
snR19	11.423374	16.296246	-0.512550837	0.00149023543966	0.001996414	-
YMR236W	121.360077	147.344955	-0.279903758	0.00149435960027	0.00200150688209	-
YFR021W	20.834745	26.066298	-0.32319426	0.0015004801607	0.00200927110389	-
YER080W	174.488617	201.548233	-0.207992212	0.00150590730822	0.00201610363801	-
YIL157C	273.363281	288.552734	-0.078015532	0.00150818221445	0.00201871392568	-
YPL130W	1.037026	2.275324	-1.13361993	0.00152760818682	0.00204427497818	down
YPL246C	74.067894	89.878754	-0.279131807	0.00153902087176	0.00205910378705	-
YDR179W-A	7.781178	10.524128	-0.435640213	0.00155308059382	0.00207746699557	-

YGL020C	31.875471	30.659128	0.0561299949814	0.00155537698698	0.00208009055045	-
YLR305C	7.608755	7.629753	-0.003975944	0.00155640464756	0.00208101659532	-
YMR279C	4.867833	4.20122	0.212471339	0.00158298160999	0.00211609600233	-
YKL091C	95.522537	98.962914	-0.051046828	0.00160054898877	0.00213911907907	-
YDR523C	0.82918	1.567296	-0.91852045	0.0016414076047	0.00219325407016	-
YLR092W	13.341514	16.548225	-0.310754085	0.00165090822431	0.0022054741245	-
YLR046C	9.039968	7.758372	0.220563714	0.00167120865178	0.00223211344885	-
YDR252W	19.150749	16.772013	0.191344965	0.00170448950429	0.00227607460586	-
YOR192C-B	5.761278	7.208781	-0.323366447	0.00170799816943	0.00228026938405	-
YGR281W	17.453281	20.927752	-0.261919079	0.00170814572779	0.00227997606495	-
YFR038W	15.738877	19.401112	-0.301806739	0.00173853124753	0.00232003482043	-
YCL054W-A	2.487303	0.0001	14.60229465	0.00174571697202	0.00232912335318	up
YCR099C	5.850695	4.119958	0.505978381	0.00174673535018	0.00232998131799	-
YPL269W	11.46387	11.038597	0.0545373367092	0.00175571629986	0.00234145795693	-
YHR204W	5.437575	7.221292	-0.409293586	0.0017772672145	0.00236968961933	-
YER116C	94.100563	112.774689	-0.261168048	0.00178255472705	0.00237622927755	-
YMR030W	49.189648	50.049263	-0.024994094	0.00184794989575	0.00246287525822	-
YFL055W	9.684726	9.099268	0.0899607418291	0.0018558624716	0.00247288993855	-
YLR318W	4.260819	3.865395	0.140514918	0.00194102890253	0.0025858170444	-
YJL037W	13.959657	19.053221	-0.448771416	0.00198403583777	0.00264254333424	-
YOL165C	4.498696	2.880045	0.643415526	0.00199539408137	0.00265710134216	-
YPR027C	41.841072	41.569187	0.00940528263839	0.00201286659084	0.00267979322238	-

YGR016W	19.287298	17.451021	0.144339601	0.00203515529807	0.00270888595421	-
YHR029C	16.665581	21.849361	-0.390719474	0.002036532	0.00271013747886	-
YBL035C	3.875915	3.388855	0.193739023	0.00210829957278	0.00280504152332	-
YGL215W	72.266548	85.815735	-0.24791422	0.00216304277038	0.0028772593783	-
YCR024C	5.765587	7.924916	-0.458928146	0.0021724760202	0.00288918833192	-
YMR112C	35.005337	46.080807	-0.396591087	0.00217305238585	0.00288933587735	-
YLR008C	26.670155	35.165863	-0.398949413	0.00220236813451	0.00292768766147	-
YGR212W	16.230726	15.709685	0.0470732800702	0.00220290935713	0.00292778019462	-
YCL056C	29.1336	26.820995	0.119321225	0.00222523772063	0.00295682272468	-
YNL029C	4.975791	4.326779	0.201632455	0.00226023723604	0.00300268623183	-
YNL104C	275.765717	298.362366	-0.113622459	0.00227489514343	0.00302151241986	-
YLR014C	3.413268	4.645705	-0.444743851	0.00232100388179	0.00308209456645	-
YDL246C	13.649888	17.872646	-0.388864124	0.00237551374417	0.00315380438918	-
YIR021W-A	6.635407	3.846315	0.78670804	0.00237959848497	0.00315855193386	-
YDR542W	0.324019	1.458961	-2.170791	0.00238371578409	0.00316334065575	down
YHR093W	2.325561	4.251551	-0.870410461	0.00238487743512	0.00316420583826	-
YHR101C	22.674231	21.974632	0.0452146170027	0.00238689254118	0.00316620275548	-
YGR081C	26.017635	33.75264	-0.375510521	0.00239686448906	0.00317875128136	-
YAR028W	34.132572	33.205696	0.0397183921416	0.00240748545831	0.00319215500324	-
YOR124C	36.35519	38.306644	-0.075433303	0.00242021293392	0.00320834548234	-
YBR171W	44.149288	55.371349	-0.326749499	0.00242245692557	0.00321063462723	-
YNR020C	12.778829	17.193468	-0.428104933	0.00245784655172	0.00325684341368	-

YBR044C	19.468506	24.13199	-0.309804713	0.00252825857236	0.00334943005063	-
YFL051C	16.130373	14.081163	0.196013305	0.00255770930571	0.00338772335606	-
YFL068W	20.299587	18.24057	0.154299563	0.00259338887412	0.00343424874798	-
YOR279C	12.627996	11.590713	0.123656393	0.00260672528061	0.00345117307358	-
YGR040W	12.078615	11.189718	0.110281358	0.00266696143296	0.0035301698456	-
YFL005W	301.2659	350.858246	-0.219850885	0.00270863304173	0.00358456489513	-
YMR007W	6.057541	9.899861	-0.708676005	0.00272226725691	0.00360184039448	-
YPL110C	6.327568	6.142796	0.042755634	0.00272488762796	0.00360453918482	-
YOR062C	43.114174	53.296227	-0.305871163	0.00274657824255	0.00363245797395	-
YNL039W	45.032875	46.66317	-0.051305733	0.00274818166355	0.00363380442328	-
YNR047W	63.735523	67.573341	-0.084356505	0.00275920174372	0.003647599	-
YLR003C	18.351898	17.332245	0.0824707428912	0.00277194382061	0.00366366345291	-
snR35	13.407453	21.278704	-0.666375089	0.00278972117821	0.00368637485618	-
YKR060W	8.801417	7.622006	0.207565068	0.0027983344837	0.00369696966904	-
YGL202W	41.809452	42.900131	-0.037152919	0.00280097194568	0.00369966677421	-
YCR054C	2.073176	3.143468	-0.600514479	0.00283125013399	0.00373886424842	-
YKL206C	45.323326	45.326519	-0.000101633	0.00287358185026	0.00379395919319	-
YDL200C	98.788345	100.941483	-0.031106439	0.00290581115806	0.00383569544318	-
YEL073C	52.787155	50.166721	0.0734562693924	0.00295036954513	0.00389368497793	-
YML087C	22.033506	28.02902	-0.347222226	0.00307046049432	0.00405131110494	-
YMR214W	14.636137	18.885094	-0.36771314	0.00312058913956	0.00411657827845	-
YNL115C	139.912109	161.765854	-0.209386283	0.0031310285968	0.00412947217525	-

YPR091C	32.699661	39.067684	-0.256700053	0.00313482274343	0.00413359804401	-
YLL063C	6.70441	9.049243	-0.432686733	0.00314972697576	0.00415236888203	-
YBL071W-A	183.560974	223.765549	-0.285728569	0.00316651892228	0.0041736198449	-
tQ(UUG)E1	4.04208	0.510816	2.984222256	0.00323923930999	0.00426856243609	up
YKL086W	25.064806	22.533316	0.153603432	0.00327202240103	0.00431084784924	-
YDL007C-A	0.702946	0.0001	12.77919815	0.00328581472497	0.00432810053312	up
YAL009W	30.450113	38.387123	-0.334174857	0.00328876173156	0.00433106339192	-
YLR453C	9.319937	8.536465	0.126681438	0.0033494146998	0.00441000349021	-
YDR343C	1002.384583	1132.887329	-0.176568255	0.00335673441085	0.00441870382158	-
YGR225W	1.440439	1.047764	0.459194768	0.00339792968125	0.00447198377384	-
YLR461W	2.498882	1.20794	1.048733982	0.00340099815922	0.00447507345749	up
YEL019C	25.61232	24.740131	0.0499848007386	0.00340170070343	0.00447504936785	-
YDL076C	39.099049	48.236923	-0.303004364	0.00340370479252	0.00447673715084	-
YFL062W	6.562689	5.734585	0.194597982	0.00342862173193	0.00450855405884	-
YAL067C	5.138558	4.607996	0.157224098	0.00346835764476	0.00455983995313	-
YPL249C	20.230131	20.678493	-0.031625387	0.00352327695029	0.00463106146673	-
YFL029C	34.338104	34.504299	-0.006965741	0.0035554669539	0.00467238332977	-
YOR353C	15.041274	18.514349	-0.299717054	0.00362711677858	0.0047655324786	-
YDL021W	64.238258	65.983414	-0.038670655	0.00365195078611	0.00479714567926	-
YLR408C	29.002321	26.523115	0.128918139	0.00369449281637	0.00485200156633	-
YJL178C	68.304581	82.440842	-0.2713769	0.00370673156953	0.0048670451742	-
YER127W	8.496382	11.477299	-0.43386263	0.00371153394036	0.00487232030065	-

YPL281C	25.562483	31.561764	-0.304149862	0.00380108693198	0.00498882614667	-
YKR095W-A	44.066948	40.589581	0.118587534	0.00381614969885	0.00500753695423	-
snR10	20.141024	16.601795	0.278797799	0.0038364491843	0.00503311000341	-
YGL082W	95.125626	99.799263	-0.069195119	0.00385291990318	0.00505365027655	-
YBR120C	80.208664	98.244011	-0.29261138	0.00385431013868	0.00505440585994	-
YGL105W	156.134583	181.955933	-0.220798971	0.00386097521273	0.00506207689981	-
YER011W	3.170451	2.262256	0.486925884	0.00386501431663	0.00506630255018	-
YNR033W	21.296242	25.809189	-0.277285938	0.00387684516017	0.00508073775688	-
YJL133W	12.42527	16.425739	-0.402681078	0.00392775929489	0.00514637604531	-
YOL003C	15.058952	19.33531	-0.360616523	0.00396527580062	0.00519443599288	-
YOR161C-C	2.509542	0.554332	2.178601894	0.00396653331492	0.00519498709262	up
YBR233W-A	46.244415	43.160133	0.0995798293691	0.00399619710816	0.00523273394799	-
YPL060W	15.142368	19.312391	-0.350935955	0.00402050726284	0.00526345615515	-
YCR087C-A	6.913667	5.303885	0.382401622	0.00404143607464	0.00528973964818	-
YGR140W	5.603553	5.333666	0.0712143908212	0.00406686028221	0.00532189473692	-
YLR319C	10.797723	13.507626	-0.323047027	0.00415519437458	0.00543634281926	-
YDL077C	8.670631	10.804503	-0.317423817	0.00423421993681	0.00553856665986	-
YMR055C	17.135557	16.272171	0.0745863428622	0.00424509115229	0.0055516169946	-
YLR412W	34.173969	42.529003	-0.315549221	0.0042512506128	0.00555850122246	-
YPR021C	7.227417	6.987168	0.0487723068626	0.00425521890985	0.00556251821275	-
YFR025C	35.796898	35.955627	-0.006382993	0.00431647693971	0.0056414081772	-
snR24	1.225629	5.167451	-2.075930465	0.00431680038668	0.00564064340149	down

YDL220C	11.691744	11.671807	0.00246221287268	0.00434700585647	0.00567891674179	-
YGR066C	11.344041	15.185235	-0.420734583	0.0043655241115	0.00570190904359	-
YCL001W	45.59235	44.893494	0.0222853893804	0.00439281014664	0.00573634105813	-
YGR106C	120.956261	143.052048	-0.242054703	0.00441109572492	0.00575900783603	-
YOL095C	3.412291	4.702083	-0.46255932	0.00442258905057	0.00577279916441	-
YBR229C	14.576653	14.754725	-0.017517537	0.0044313304168	0.00578299332089	-
YHR045W	11.03166	10.626218	0.0540216826922	0.00447491722057	0.00583864777328	-
YBR125C	33.377552	33.69445	-0.013632829	0.00447986080738	0.00584386969788	-
YLR144C	9.474014	11.942858	-0.334100415	0.00448200826704	0.00584544271466	-
YEL020C	99.590294	105.806664	-0.087353445	0.00451665894334	0.00588939691667	-
YCR069W	43.684864	53.26226	-0.285980147	0.00451805125667	0.00588997526279	-
YDL216C	23.383667	28.916979	-0.30641565	0.00454894877383	0.0059290098652	-
YLR193C	372.76712	433.020782	-0.216161654	0.00454914212776	0.00592801728151	-
YIR009W	20.996382	28.978937	-0.464863924	0.00456144225353	0.00594279821824	-
YNL332W	3.626783	5.384534	-0.570131066	0.0046225901948	0.00602120015302	-
YDR224C	553.01825	640.941284	-0.212865108	0.00463381197851	0.00603455103055	-
YER089C	33.890076	41.095188	-0.278106601	0.00468946635011	0.0061057481337	-
YJL115W	51.599651	52.339634	-0.020542528	0.00470785493252	0.00612840499499	-
YLR111W	3.69434	2.187149	0.75626514	0.00471789426831	0.00614018608337	-
YNL256W	5.778157	7.495081	-0.375334665	0.00472505120045	0.00614821166472	-
YOL091W	1.20646	0.853506	0.499306884	0.00480659066902	0.00625299976388	-
YOR350C	46.101673	54.568501	-0.243249307	0.00482588415885	0.00627678375406	-

YDL191W	369.13974	432.478668	-0.228461913	0.00483404549341	0.00628608178112	-
YHR140W	16.006598	14.842253	0.108956609	0.00484759857517	0.00630238574967	-
YNL001W	26.749969	33.056969	-0.30541723	0.00485601383104	0.0063120045777	-
YER038C	11.448226	14.705093	-0.361191851	0.00489900078219	0.00636654738452	-
YGR149W	22.774364	22.665443	0.00691640646775	0.00510298761124	0.00663025263511	-
YDR058C	35.366928	35.510078	-0.005827619	0.00519824795647	0.0067526100259	-
YJR040W	7.000736	8.971762	-0.357884746	0.00521882099796	0.00677791647601	-
YOR093C	4.643011	4.552166	0.0285075307252	0.00536215980543	0.00696262038739	-
YIL087C	279.941956	295.964874	-0.080298237	0.00537134773872	0.00697309217105	-
YDL247W	4.737516	6.382443	-0.429977933	0.00537443565785	0.00697564218356	-
YIL132C	6.382672	5.197159	0.296437314	0.0054129822775	0.00702420442699	-
YNR066C	5.481945	7.53352	-0.458636261	0.00543365548802	0.0070495576321	-
YER112W	106.842819	128.194702	-0.262846693	0.00544041609809	0.00705685397763	-
YLR260W	19.032806	19.247723	-0.016199512	0.00544051050302	0.00705550223579	-
YPR025C	21.849766	27.230091	-0.317583974	0.00544969206876	0.00706593324203	-
YOR192C	3.001566	4.240711	-0.498590777	0.00548103077278	0.00710508227969	-
YOL015W	3.780339	5.225696	-0.467107586	0.00548345083362	0.00710673544366	-
YBR112C	38.654522	45.459435	-0.233942556	0.00549423267641	0.00711922280425	-
YNL090W	19.298199	17.89607	0.10882341	0.00550704858762	0.00713434007344	-
YFL013C	54.979725	64.616379	-0.233000216	0.00563495866045	0.00729852354769	-
YBR271W	16.794769	21.151669	-0.332759553	0.00565699578619	0.00732553813948	-
YDR388W	409.269043	466.721985	-0.189513881	0.00565760528935	0.00732479950705	-

YER044C-A	1.516283	1.045805	0.535925171	0.0056634817882	0.00733087884511	-
tW(CCA)P	3.62076	0.679375	2.414012517	0.00577286293803	0.00747090538238	up
YOL029C	5.980619	8.857826	-0.566657845	0.00580108243986	0.00750586073086	-
YDR486C	122.012405	127.371078	-0.06200989	0.00581927699826	0.00752783321633	-
YJL035C	3.676479	5.685277	-0.628905898	0.00585319231159	0.00757012872299	-
YHR188C	41.777935	49.671951	-0.249690229	0.00597388761337	0.0077246186844	-
YGL059W	121.771736	141.534225	-0.216971646	0.00600837606408	0.00776759654431	-
YLR363W-A	27.86677	38.189903	-0.454645464	0.00601715107564	0.00777732123206	-
YGR169C	6.432191	8.713623	-0.437962448	0.00612274658173	0.00791215878006	-
YDL035C	38.39312	45.134571	-0.23338509	0.00621990971481	0.00803604568357	-
tG(GCC)J1	2.315027	0.0001	14.4987414	0.00622587333735	0.00804207692016	up
YHR026W	95.734344	114.625496	-0.259819498	0.00625462833717	0.00807753957087	-
YMR293C	10.790487	13.874122	-0.362636497	0.00628730814628	0.00811805511067	-
YOR288C	44.192787	44.946819	-0.024408099	0.00633541779128	0.00817847237436	-
YJL114W	8.508183	7.358698	0.209400536	0.00642226400735	0.00828885965855	-
YMR014W	8.804345	11.38835	-0.371271154	0.00642529623288	0.00829104947282	-
YLR031W	4.139143	2.983869	0.472147893	0.00645323035867	0.00832536451925	-
snR32	1.256747	3.535729	-1.492313457	0.00649081474007	0.00837211259221	down
YBR013C	4.805767	7.868371	-0.71129828	0.00649813844943	0.00837981792564	-
YBL011W	20.926809	25.276886	-0.2724664	0.00654591197059	0.00843967217309	-
YBR185C	27.133009	26.676121	0.024500151	0.00665031251166	0.00857249586221	-
YJL047C-A	8.446326	4.941272	0.773441458	0.00666298384821	0.00858704665345	-

snR57	63.669029	54.592991	0.221876022	0.00667736015098	0.00860378825597	-
YDL175C	27.823118	34.381611	-0.305353041	0.00667952032841	0.00860478568142	-
YDR051C	77.199409	91.667564	-0.247821532	0.00669120498678	0.00861804990828	-
YMR107W	8959.475586	9873.841797	-0.140197239	0.00673789728892	0.008676388	-
YER113C	5.652195	5.288868	0.0958522703959	0.0068732705274	0.00884887254959	-
YJR068W	77.713493	92.065247	-0.244491559	0.00707246986783	0.00910344037725	-
YDR359C	15.423868	18.6562	-0.27449055	0.00709507467229	0.00913064335937	-
YHL046C	3.625853	2.262618	0.680327403	0.00725182744615	0.00933043415247	-
YMR282C	22.076941	22.381308	-0.019754067	0.00726753731841	0.00934870942243	-
YNL306W	125.985306	149.203278	-0.244023754	0.00731526110116	0.0094081501794	-
YOR144C	4.019527	3.696849	0.120729624	0.00732045509827	0.00941288012636	-
YOR257W	405.696716	433.621216	-0.096033721	0.00734577125018	0.00944347647983	-
YPL165C	59.768715	62.023293	-0.053419599	0.00737550425847	0.00947973715043	-
YAR002W	23.825302	24.157694	-0.019988246	0.00741238522778	0.00952516818341	-
YGR039W	3.726432	2.234865	0.737607247	0.00747208259473	0.00959989419456	-
YDR235W	18.352081	22.609808	-0.301005077	0.00758937984903	0.00974857647481	-
YMR315W	173.423904	185.095001	-0.093963165	0.0076047790091	0.00976633597197	-
YDR339C	49.547806	61.15527	-0.303655651	0.0076601804138	0.00983544984672	-
YJL143W	202.104523	238.168152	-0.236878899	0.00766482191264	0.00983937436593	-
YDR379W	9.903584	9.920197	-0.002418055	0.00773591136964	0.00992857923976	-
YIL010W	43.591671	53.802837	-0.30362974	0.00811979028765	0.0104191108114	-
YLR127C	18.744455	19.221142	-0.036230175	0.00819837610847	0.0105177761689	-

YNL050C	30.792042	38.196915	-0.310898579	0.00833014207273	0.0106846119809	-
YIR028W	2.884284	4.030381	-0.482703	0.00838369424684	0.010751079	-
YCL069W	1.798832	2.791738	-0.634103099	0.00841313781234	0.0107866087441	-
YBR244W	10.863571	15.223567	-0.486808019	0.00860076310529	0.0110248889857	-
YJL042W	43.266945	50.183899	-0.213959306	0.00860280175132	0.0110252256962	-
YMR139W	157.784897	183.100418	-0.214675966	0.00886250527809	0.0113557136773	-
YDL117W	4.398233	4.139549	0.0874504381622	0.00893092636578	0.0114410216423	-
YOR367W	58.366043	59.098824	-0.01800016	0.00905481136165	0.0115973321504	-
YNR072W	5.202904	6.924073	-0.412303847	0.0092355027295	0.0118263203269	-
YLR146C	7.245132	6.419757	0.174493285	0.00932102357395	0.0119333706635	-
YPR011C	79.715775	83.308296	-0.063594921	0.009408932	0.0120434332966	-
YOL155W-A	1.442636	4.233994	-1.553311894	0.0094532034348	0.0120976060448	down
YHR157W	3.41581	5.485684	-0.68344379	0.00954306750631	0.0122100913189	-
YER077C	10.979262	13.67195	-0.316437943	0.00956072595145	0.0122301641679	-
YIL111W	127.695213	152.615616	-0.257198147	0.0096680839915	0.0123649496125	-
YOR393W	26.10948	31.846268	-0.286550591	0.00975197414338	0.012469672	-
RDN5-2	14.917405	24.204933	-0.698304512	0.00983768001087	0.0125766716449	-
YGL229C	2.16426	1.868718	0.211824951	0.00990177157437	0.0126560012217	-
YDR448W	10.795123	10.343404	0.061668629	0.00991275355125	0.01266743	-
YHR173C	2.889006	1.702527	0.762895522	0.00995656758076	0.0127208008935	-
YMR134W	61.953438	63.441303	-0.034238058	0.0100268042055	0.012807901	-
YGR196C	22.401854	26.81078	-0.259195056	0.01008778	0.0128831385061	-

YFL020C	19.359615	26.181581	-0.435501956	0.0101489776519	0.0129586288076	-
YBR001C	50.444447	59.016068	-0.226412339	0.0101835067517	0.0130000431657	-
YGL260W	1.586148	0.517815	1.615018729	0.0102654161026	0.0131019126572	up
YCR045C	3.308983	2.845208	0.217853755	0.0102761544647	0.0131129222851	-
YLR240W	22.081501	26.372734	-0.256208896	0.0104649180974	0.0133510504621	-
YBR029C	15.650092	15.446166	0.0189223569289	0.0104779969059	0.01336499	-
YML068W	25.050751	30.506983	-0.284285656	0.0105058023616	0.0133977035868	-
YMR091C	36.114742	43.44112	-0.266473435	0.0105317124272	0.0134279874201	-
YBR175W	2.700672	4.105452	-0.604222634	0.010660538	0.0135894496021	-
YDR010C	6.923185	5.210754	0.409943753	0.0107125031254	0.0136528884012	-
YKR100C	55.885052	57.945683	-0.052238737	0.0107573369041	0.0137072141832	-
YIL037C	3.350974	2.989358	0.164744811	0.0112512529728	0.0143336298902	-
YIL011W	8.7963	11.871345	-0.432514685	0.0112697158042	0.0143542051113	-
YDR191W	6.120034	8.285127	-0.436984144	0.0112918349655	0.014379428	-
YNL217W	50.996906	52.565605	-0.043709396	0.0112965947221	0.0143825389735	-
YEL012W	621.755615	674.058167	-0.116525461	0.0113666890383	0.0144688139327	-
YOR351C	1.075484	1.760613	-0.711091764	0.0114765905971	0.0146057143147	-
YDR027C	22.957905	23.842813	-0.05456346	0.0114794957975	0.0146064172804	-
YGR181W	149.102478	179.18158	-0.2651181	0.0114924858624	0.0146199492282	-
YJR036C	8.756746	8.709562	0.00779469689879	0.0115646200987	0.0147086993593	-
YOL138C	8.796193	8.935507	-0.022670331	0.0118141588016	0.0150230024253	-
YPL051W	20.345245	19.320534	0.0745566842763	0.0120623722271	0.0153354918668	-

YNL329C	13.962272	14.314978	-0.035991731	0.0121369565024	0.0154271551938	-
YBR288C	12.386944	12.115393	0.0319790983033	0.0121942228281	0.0154967728387	-
YIL134W	4.35411	6.220893	-0.514743836	0.0122286404475	0.0155373311293	-
YHR191C	29.391748	37.989944	-0.370206423	0.012233495	0.0155403186443	-
YJL066C	399.972595	458.956482	-0.198456211	0.012541943	0.0159288835507	-
YLR108C	7.945988	7.528419	0.0778796900626	0.0125580233798	0.0159460440053	-
YOR079C	1.152033	2.036697	-0.822049323	0.0129179576736	0.0163997303145	-
YER173W	15.714428	19.193308	-0.288513624	0.0129492526839	0.0164360990926	-
YJR051W	44.135445	52.374115	-0.246916217	0.0129499345556	0.0164336046037	-
YKR005C	3.619202	4.970301	-0.457661592	0.0129832450791	0.0164725087781	-
YDR132C	21.107101	21.343729	-0.016083812	0.0129910438314	0.0164790355752	-
YMR312W	33.651478	41.32756	-0.296434318	0.0129964420525	0.0164825152732	-
YGL092W	11.286077	13.57402	-0.266303946	0.0129986860456	0.0164819940708	-
YHR092C	3.341301	2.947989	0.180678812	0.0131025156336	0.0166102546566	-
YFR051C	30.666311	36.717068	-0.25979623	0.013112985	0.0166201326993	-
YJL131C	34.846039	42.204563	-0.276404314	0.0131174488704	0.0166223969356	-
YHR198C	119.026466	139.091003	-0.224746705	0.0132974305508	0.0168470303795	-
YDL043C	30.766237	30.744799	0.00100562434158	0.0133552994158	0.016916894	-
YDR281C	3.559156	6.149074	-0.788834	0.0135549460968	0.01716628	-
YLR105C	13.795081	13.382195	0.0438391558525	0.0135845173085	0.0172002209772	-
YAL058W	13.524676	13.369784	0.0166178752793	0.0136602261258	0.0172925537906	-
YJL081C	74.523575	78.893913	-0.08221711	0.0137617468045	0.0174175176682	-

YKL179C	37.161083	38.935268	-0.067285009	0.0138994149825	0.017588171	-
YCR034W	4.245618	3.615951	0.231599443	0.0139229126327	0.0176143145759	-
YML047C	0.421328	0.909026	-1.109377763	0.0142054176754	0.017968059	down
YKL213C	32.238224	33.688965	-0.063503838	0.0142813349812	0.0180604048815	-
YGL258W-A	14.279781	11.69001	0.28869769	0.01434053	0.01813157	-
YHR155W	6.619598	8.166849	-0.303035947	0.014455356	0.0182730302751	-
YBR084C-A	526.681458	569.036255	-0.111589904	0.014512164	0.018341106	-
YLR447C	129.796402	138.191772	-0.090421328	0.0150818337611	0.0190572000792	-
YDR397C	172.057495	180.397186	-0.068286096	0.0151114771547	0.0190907713017	-
YCR092C	1.81094	2.480593	-0.453946299	0.0151774434749	0.0191702073433	-
YKL189W	26.932886	27.347313	-0.022030258	0.0153421077494	0.019374248	-
YDR215C	9.805317	13.861108	-0.499406407	0.0156956461475	0.0198166709953	-
YER008C	16.474459	17.203867	-0.062501793	0.0157241629274	0.0198486383597	-
YLR042C	3.995812	2.973599	0.426278599	0.0157894926891	0.0199270523712	-
YLR113W	64.587852	67.996017	-0.074187399	0.0159957596181	0.0201832674205	-
YKR045C	27.991341	35.358784	-0.337088056	0.0160926919834	0.0203014492649	-
YDR109C	11.610703	11.644125	-0.004146905	0.0162372086478	0.0204796000174	-
YLR372W	10.128404	9.569818	0.0818434642636	0.016645843	0.0209907354887	-
YDR170C	17.141575	20.049843	-0.226091267	0.0167608145125	0.0211314249581	-
YAR027W	270.40451	290.165436	-0.101756464	0.0168862840106	0.0212852895711	-
YJR097W	7.253173	10.329336	-0.510063351	0.0173228231337	0.0218311177454	-
YBR119W	46.603485	56.003227	-0.265072117	0.0174156092289	0.0219435969339	-

YER050C	224.063644	262.659454	-0.229284933	0.017609177	0.0221829890314	-
YGR096W	7.270661	6.598254	0.140002215	0.0176113755231	0.022181258	-
YPL065W	40.911438	41.455658	-0.019064771	0.0178201717269	0.0224396807466	-
YHR108W	43.720539	51.502197	-0.23632279	0.0178732707007	0.0225019802291	-
YMR053C	21.057108	25.070898	-0.251706363	0.0179639541665	0.0226115627465	-
tK(CUU)K	2.92399	0.586556	2.31759762	0.0180471492802	0.0227116770183	up
YOR281C	32.293552	32.616661	-0.014362966	0.0181114564463	0.0227879857355	-
YHR199C	146.097153	169.708405	-0.216129953	0.0181118349662	0.0227838442696	-
YGL157W	9.648454	9.114213	0.0821797067821	0.0182019379194	0.0228925507706	-
YGR277C	12.297781	15.812417	-0.362659887	0.0182972951559	0.02300782	-
YGL261C	1.819618	0.925348	0.975567677	0.0188535893128	0.023702528	-
YBR091C	25.169403	33.077366	-0.394173358	0.0189008291443	0.0237571061608	-
YFR032C	0.104061	0.424877	-2.029615771	0.0189105050153	0.0237644565051	down
YDR021W	5.975899	7.955375	-0.412774173	0.0189506431566	0.0238100774573	-
YFL034C-A	14.27317	19.543829	-0.45340736	0.0190325489259	0.0239081472545	-
YFL064C	1.645374	0.974341	0.755916871	0.0190698475494	0.0239501544785	-
YHR189W	32.958866	40.978836	-0.3142124	0.019285445	0.0242160275952	-
YCL048W	1.004122	0.699807	0.520905566	0.0196753720207	0.0247006490403	-
YGL096W	135.568771	158.05072	-0.221362723	0.0196781780781	0.0246991770136	-
YDR166C	19.849119	20.688137	-0.059728759	0.0197311231912	0.0247606251811	-
YLR013W	0.937499	0.383359	1.290121102	0.0198279350238	0.0248770858181	up
YHR078W	19.200808	23.283289	-0.278127845	0.0198478334806	0.0248970196499	-

YLR159W	21.358814	28.321478	-0.407065017	0.0198695295481	0.0249191998858	-
YKR017C	43.306992	45.449696	-0.069670675	0.0200171180204	0.0250992261504	-
YJR067C	36.731102	46.088299	-0.327398342	0.0201729062528	0.0252894592119	-
YPR133W-A	161.304108	162.191956	-0.007919089	0.0202611666675	0.0253949773211	-
SRG1	392.665283	422.734802	-0.106452839	0.0203314505666	0.0254779259422	-
YIL015W	2.341471	3.270237	-0.481980024	0.0209631649166	0.0262642437542	-
YOR346W	9.808874	11.9342	-0.282942421	0.0209714443327	0.0262693152577	-
YMR225C	121.078133	146.086288	-0.270882435	0.0210128519569	0.0263158735018	-
YLR419W	3.258663	4.114242	-0.336346495	0.0210815157181	0.0263965408588	-
YOR226C	10.379241	9.13505	0.184216418	0.0214583617057	0.026862978	-
YHR068W	10.190217	12.997764	-0.351078684	0.0215589850342	0.0269835038492	-
YIR021W	4.6299	4.099248	0.175621759	0.0216883976991	0.0271400066349	-
YGL259W	12.600749	11.443477	0.138984022	0.0217511567462	0.0272130554374	-
snR82	51.480419	65.028847	-0.337056048	0.0219196700097	0.0274183581342	-
snR71	7.474884	4.007798	0.899241395	0.0220210892369	0.0275396700206	-
YLR302C	2.736118	1.742871	0.650664659	0.0220215474791	0.0275346962236	-
tC(GCA)B	16.246012	10.851327	0.582214136	0.0222809288077	0.0278534043573	-
YHR125W	3.488728	2.248914	0.633472629	0.0223008886184	0.0278727434152	-
YER188C-A	1.999081	1.070183	0.901479412	0.0225076851885	0.0281255454208	-
YGL104C	13.29253	13.211452	0.00882668826133	0.0225531619302	0.0281767014013	-
YAR010C	17.40497	17.491352	-0.00714248	0.0225784063805	0.0282025647506	-
YLR264C-A	6.283381	11.677032	-0.894060658	0.0228452454664	0.028530132	-

YCL001W-B	6.112874	4.39969	0.474448954	0.0230901828191	0.0288302202214	-
YNL234W	7.307756	9.467641	-0.373576538	0.0234987924595	0.0293345070559	-
YOR105W	3.922056	2.643735	0.569032562	0.023731519	0.0296190728888	-
YAR020C	5.461583	3.475625	0.652046734	0.0237498609666	0.0296360074131	-
YML050W	15.549921	19.553946	-0.330552523	0.0238705219999	0.0297805869323	-
YEL059C-A	128.033447	155.460556	-0.280027837	0.0241047691904	0.0300667886546	-
YDR430C	23.249575	27.402294	-0.23709233	0.0241047803188	0.0300607625993	-
tS(UGA)E	13.509006	8.840811	0.6116709	0.0243212635019	0.0303215993212	-
snR190	5.797661	3.831491	0.597565062	0.0243212635019	0.0303215993212	-
YGL242C	16.690825	21.620512	-0.373345422	0.0243661227881	0.0303683778897	-
YER128W	21.00621	26.630526	-0.342265034	0.024505121	0.0305354859657	-
YMR274C	3.682989	3.104049	0.246725756	0.0250843016222	0.031250922	-
YPL203W	177.781769	191.418869	-0.106625662	0.0252019172529	0.0313911521481	-
YNL218W	13.235847	16.215948	-0.292962848	0.0252892519413	0.0314936160585	-
YLR145W	11.249063	14.918576	-0.407304999	0.0254956504431	0.0317442835841	-
YGL175C	2.570889	2.097171	0.293822821	0.0255191290335	0.0317671451855	-
tV(AAC)G1	6.007668	2.66744	1.171349268	0.0255596667413	0.0318112291761	up
YER106W	1.347704	2.208634	-0.712650697	0.025653162	0.0319211926773	-
YER156C	30.449572	31.106323	-0.030785918	0.0256762670297	0.0319435402245	-
YKL203C	8.965121	10.568352	-0.237355464	0.0257851796801	0.0320726097885	-
RUF20	3.60875	2.648707	0.446210941	0.0258976423224	0.0322060423753	-
YER044C	326.637207	378.064514	-0.210943304	0.0259064289935	0.032210517	-

YER180C-A	54.809216	68.893799	-0.329955636	0.0261497605337	0.0325065505393	-
YHR105W	27.711119	27.520376	0.00996478817793	0.0263172857929	0.0327082502908	-
YCL068C	1.29295	2.199944	-0.766800315	0.0263743565621	0.0327726192028	-
YNL335W	10.269478	9.478237	0.11567221	0.0265869381669	0.0330301605243	-
YBR221W-A	4.841033	2.411748	1.005235759	0.0267484109482	0.0332174705274	up
YIR041W	1.310738	0.673265	0.961132965	0.0267484109482	0.0332174705274	-
RUF21	0.717716	0.35651	1.009470539	0.0267484109482	0.0332174705274	up
YMR166C	10.631406	13.505327	-0.345196166	0.0267614663638	0.0332203925588	-
YKL130C	34.683819	35.1315	-0.018502415	0.0270188762569	0.0335332234711	-
YHL019C	30.855814	32.24976	-0.06374607	0.0270337552104	0.0335449834791	-
YLL006W-A	0.832321	0.038373	4.438976573	0.0273854319475	0.0339745726479	up
YGL233W	29.380739	31.035397	-0.079043917	0.0274272373122	0.0340196382086	-
YJR094W-A	240.794937	283.612823	-0.236117705	0.0275319706504	0.0341427234914	-
YGL101W	59.149349	70.914131	-0.26171085	0.027541529	0.0341477550788	-
YHR054C	4.476545	6.109457	-0.448658472	0.0277565725909	0.0344075085153	-
YNL316C	9.3422	12.023896	-0.3640702	0.0279058620809	0.0345856641641	-
YOR268C	3.074023	2.160248	0.508931014	0.0280215162321	0.0347220704129	-
YKL079W	20.148941	24.11824	-0.259420617	0.0281254030462	0.0348438439655	-
YLR361C	16.166073	16.443434	-0.024542351	0.0282273290947	0.0349631402673	-
YPR157W	2.149086	3.076792	-0.5177037	0.0283170964972	0.0350673319478	-
YKL222C	7.472844	9.314172	-0.317770118	0.0286893125152	0.0355211910839	-
YIL012W	2.082861	1.219447	0.772339508	0.0289376035853	0.0358214642189	-

snR56	3.265336	1.096614	1.574175654	0.0289394852003	0.0358166515398	up
snR51	38.359314	33.451862	0.197490404	0.0290850570126	0.035989642	-
YDR403W	20.045931	24.174212	-0.270159441	0.0293270914385	0.0362819018833	-
YFR005C	4.917635	6.501907	-0.402898262	0.0294054019377	0.036371535	-
YBR254C	25.095299	31.563128	-0.330823053	0.0294899424436	0.0364688371893	-
ETS2-2	8.309159	12.843371	-0.628249547	0.0296113748178	0.036611714	-
YNL063W	39.790489	47.693489	-0.261368697	0.0296688534854	0.0366754763913	-
YFL059W	50.567265	60.205734	-0.251697146	0.0299053446266	0.0369604577826	-
YDR538W	38.212627	38.915058	-0.026279065	0.0301449357325	0.0372491562555	-
YHR035W	3.886736	3.635507	0.096402545	0.0301967334265	0.0373057355447	-
YEL017C-A	120.884338	150.490509	-0.316045165	0.0306552878203	0.0378647088715	-
YGL230C	2.625868	1.788173	0.554308076	0.0308021100738	0.0380384920108	-
YLR157C-C	8.41011	13.768998	-0.711227	0.0310551968828	0.0383434093573	-
YDR522C	1.093571	1.686205	-0.624733052	0.0311731655239	0.0384814101357	-
tQ(UUG)D3	3.318308	0.970082	1.774269194	0.031230743	0.0385409904472	up
YAL064W	0.842958	0.262832	1.681319816	0.031230743	0.0385409904472	up
YDR261C	6.468513	6.247361	0.0501872019523	0.0315208387493	0.038887394	-
YNL258C	20.90324	24.847429	-0.249370005	0.0315387282286	0.0389017335273	-
tL(UAA)D	2.276463	0.359513	2.662678166	0.0315830403277	0.038948652	up
YIL098C	78.418335	80.892792	-0.044820146	0.0316469257638	0.0390196852318	-
YOR032W-A	2.222764	1.013099	1.133579618	0.0318083869601	0.0392109742352	up
YOR253W	33.119247	32.97945	0.00610253298491	0.032000614	0.0394401052717	-

YOR008C-A	5.658012	4.014546	0.495058392	0.0321361859228	0.0395993335071	-
YNL191W	21.153095	21.360023	-0.014044435	0.0321850694477	0.0396516989742	-
YFR026C	6.413929	5.4576	0.232941724	0.0323084461771	0.0397958003705	-
YKL134C	7.568662	9.358562	-0.306248587	0.0325716488228	0.0401120404468	-
YCR018C	2.313991	3.584452	-0.63136932	0.0327339861423	0.0403039638975	-
YBR148W	1.263815	1.014933	0.316400802	0.0329822881134	0.0406016348617	-
YOR222W	21.36727	26.285326	-0.298855035	0.0329865327753	0.0405988095696	-
YKL070W	21.82456	27.696211	-0.343736053	0.0333011681403	0.040977929	-
YLR159C-A	13.000101	19.986385	-0.620494719	0.0335645663298	0.0412938620245	-
YOL144W	13.164016	13.182678	-0.002043792	0.0335939440189	0.0413218158251	-
YER181C	4.95872	3.739736	0.407031335	0.0335944097932	0.0413142028518	-
YMR155W	29.967522	31.259871	-0.060912032	0.0336298691547	0.0413496192735	-
YKR092C	23.419657	28.309273	-0.273554754	0.0338312743079	0.0415890199809	-
YIL117C	83.006264	87.821877	-0.081360157	0.0338402862632	0.0415918624277	-
YHR214C-C	2.200247	1.86809	0.236101528	0.0345745906841	0.0424859578319	-
YNL031C	1516.329834	1658.311646	-0.129131553	0.0346517978389	0.0425724047069	-
YJL029C	25.864742	27.21067	-0.073185679	0.0348474489081	0.0428043060588	-
YGL183C	3.766065	5.471058	-0.53876195	0.0350064486766	0.0429911045271	-
YHR213W	0.588584	0.225936	1.381334161	0.0350255876545	0.0430061012972	up
YGL137W	46.059875	49.247364	-0.096536009	0.0356360908012	0.043747054	-
YMR201C	70.759308	82.77005	-0.22618889	0.0358964249585	0.0440579292492	-
YGR173W	23.245663	23.681873	-0.026821614	0.0359811626488	0.0441532037406	-

YKL129C	25.740541	29.958839	-0.21893934	0.0360360710983	0.0442118437507	-
YJR021C	117.478264	125.358475	-0.093665683	0.0360599862019	0.0442324430629	-
YER012W	360.921356	414.0336	-0.19806334	0.0368101785695	0.0451437353931	-
YLR134W	0.793286	1.250467	-0.656553992	0.0368191368314	0.0451458031699	-
YAR050W	4.301774	4.325033	-0.007779408	0.0368850834424	0.0452177326245	-
YMR322C	53.837975	64.288643	-0.255939752	0.036976084	0.0453203414286	-
YJL208C	14.787688	14.622717	0.0161851113446	0.0370540023289	0.0454068784954	-
YHR004C	38.47282	45.513676	-0.242460532	0.0371664646442	0.0455357040677	-
YDR438W	12.791864	15.974538	-0.320547702	0.0371826623251	0.0455465603225	-
YER137C	23.425793	22.692427	0.0458869698578	0.0375518079916	0.0459896673923	-
YDR084C	65.066559	67.464561	-0.052213596	0.0375714158294	0.046004605	-
snR14	11.463804	17.238987	-0.588589152	0.0375876436453	0.0460153996746	-
YOL085C	22.919214	21.613119	0.0846502853626	0.0378674527989	0.0463488065803	-
YPR060C	5.452797	4.851449	0.16858074	0.03799138	0.0464913237901	-
YGR076C	41.666939	41.942558	-0.009511733	0.0381449855892	0.0466700966768	-
YPL170W	203.223679	216.12326	-0.088785839	0.0381897563629	0.0467156665027	-
YNL328C	22.31249	21.490425	0.0541575084111	0.0383331119871	0.0468817886557	-
YER189W	4.817817	3.733149	0.367986504	0.0383963695631	0.046949904	-
YMR127C	13.633725	17.036547	-0.321453169	0.0384718191846	0.0470328974986	-
YCR081W	2.795195	2.732675	0.0326350439215	0.0386781591808	0.0472758441021	-
YMR316C-A	6.554291	9.741748	-0.571740936	0.03908723	0.0477664414637	-
YGR102C	220.718567	255.589996	-0.211623374	0.0392612686904	0.0479696823519	-

YDR036C	62.283184	72.501816	-0.219174433	0.0394171927364	0.0481507147791	-
YPL244C	12.552493	12.342618	0.0243254826314	0.039544714	0.048296987	-
YDR320C	42.474319	49.549259	-0.222272666	0.0397333036875	0.0485177713005	-
YDR011W	43.600677	47.05209	-0.109908271	0.039956749	0.0487810225793	-
YLL025W	9.260742	7.986501	0.213564215	0.0400393539556	0.0488722590162	-
YDR135C	16.85956	17.850086	-0.08236414	0.0401178454671	0.0489584400746	-
YDR531W	39.2234	46.611614	-0.248974871	0.04013196	0.0489660392246	-
YER163C	35.041557	42.491722	-0.278114931	0.0402470759673	0.0490968456681	-
YAL022C	11.210903	11.212694	-0.00023046	0.0404673859125	0.0493559001463	-
tV(UAC)D	4.022353	1.728136	1.218822939	0.0405752238872	0.0494777037697	up
YKR022C	37.485569	44.807888	-0.257417426	0.0408301750126	0.0497788150979	-
YMR207C	6.34428	7.551649	-0.251335265	0.0408740094817	0.0498224721897	-
YBR107C	17.882151	17.595894	0.02328147	0.041019001	0.0499893912938	-
YBR032W	4.85737	3.610281	0.428064254	0.0411948181004	0.0501938038798	-
YNL003C	45.731335	47.469044	-0.05380396	0.041296638	0.0503079920878	-
YDR092W	216.577362	251.68306	-0.216725665	0.0415804519017	0.0506437993734	-
YJL107C	9.02042	11.441827	-0.343050923	0.0416074108604	0.0506617253351	-
YDR228C	5.583016	7.073617	-0.341403416	0.0416074108604	0.0506617253351	-
YLR361C-A	150.612381	157.02803	-0.060181737	0.0416674889694	0.0507199552004	-
YHR072W	21.418325	25.367252	-0.244121583	0.0423962110865	0.0515968787346	-
YPR070W	9.240857	9.204062	0.00575595202612	0.0428359721208	0.0521218571004	-
YIL066C	16.902103	17.624626	-0.060389882	0.0429272372629	0.0522226707678	-

YHR122W	47.599503	49.144997	-0.046098047	0.0430336512788	0.052341871	-
YDR123C	77.619354	82.086861	-0.080734893	0.0430932573455	0.0524041021745	-
YML105C	52.075726	61.846134	-0.248072368	0.0431823823599	0.0525021993126	-
YHR017W	37.10627	44.065483	-0.247986033	0.0432255243078	0.0525443616414	-
YJR129C	14.143779	14.006891	0.0140308699836	0.0433231232205	0.0526526916509	-
YGR158C	26.29936	26.529799	-0.012586054	0.0437246714677	0.0531303113078	-
YHR079C-A	0.365886	0.0001	11.8371785	0.0443069730535	0.0538273363437	-
YMR066W	10.606545	12.772595	-0.26809688	0.0448713021194	0.0545022585712	-
YOR274W	33.8237	40.106026	-0.245784535	0.0450158437925	0.0546671279859	-
YDR141C	11.676951	13.721209	-0.232743988	0.0453733408894	0.0550904948644	-
YCR073W-A	129.835541	150.144699	-0.209668181	0.045467562	0.0551940998503	-
YDR366C	1.260969	0.640632	0.97696504	0.0455357445749	0.0552660610598	-
YOL001W	24.402607	24.742971	-0.019983458	0.045732834	0.0554944160362	-
YOR193W	46.258511	54.574894	-0.238518595	0.0458490142494	0.0556245222709	-
YCL067C	58.171066	60.322506	-0.052394622	0.0460803242808	0.0558942268729	-
YPL108W	24.043064	30.074953	-0.322941721	0.0462645191381	0.0561066877924	-
YCL027W	1.13768	0.89288	0.349556622	0.0463050962999	0.0561449292636	-
YHR212W-A	1.270148	0.389108	1.706754065	0.0463725171979	0.056215697	-
YGL238W	21.10504	22.247993	-0.076087632	0.0464592442598	0.0563098376347	-
YLR308W	0.475015	0.944165	-0.991065932	0.0468149779473	0.0567299205733	-
YLR156C-A	1.400695	3.525903	-1.331849941	0.047099659	0.0570637553659	-
YCR039C	47.509388	48.91983	-0.042206768	0.0471828234085	0.0571533595551	-

YCL001W-A	2.606406	1.892326	0.46190118	0.0473956160812	0.0573999189684	-
YBR245C	11.567924	12.034688	-0.057068761	0.0477515807128	0.0578197411869	-
YDL189W	11.746515	14.48253	-0.302080857	0.0482508845412	0.0584129273073	-
YBR281C	3.535777	4.499058	-0.347595678	0.0485829603366	0.0588034739266	-
YLL041C	1584.889526	1744.116333	-0.13811399	0.0485896289392	0.0588000811803	-
YNR028W	66.721481	70.414268	-0.077716479	0.0486642461835	0.0588789008589	-
YJR069C	9.859945	9.146121	0.108419593	0.0488521230237	0.0590946959726	-
YJL098W	10.686857	12.768476	-0.256748721	0.04888944	0.0591283155416	-
YOR381W-A	0.906029	2.300681	-1.344431828	0.0489559725535	0.0591972492427	-
YDL045C	67.685997	71.458443	-0.078247082	0.0489713548418	0.0592043175965	-
YLR160C	339.907043	370.324829	-0.123651025	0.0489732236985	0.0591950492057	-
snR49	13.246914	10.844467	0.288697162	0.0492448831445	0.059511823	-
YDL106C	36.170349	38.151031	-0.076914527	0.0493020783846	0.0595693465573	-
YNL309W	12.0222	12.00298	0.00230829526366	0.0493344907514	0.0595969096293	-
YOL156W	6.630007	6.50591	0.0272595286588	0.0501234181758	0.060538167	-
YNL022C	22.223574	26.567286	-0.257560006	0.0502142102342	0.0606360274526	-
YOR258W	29.435024	35.952236	-0.288547696	0.0503572474317	0.0607969257208	-
YEL038W	67.018059	79.223358	-0.24137595	0.0506389624033	0.0611251562512	-
YMR224C	9.350702	11.399968	-0.285883191	0.0507844879261	0.0612888998922	-
YLR080W	36.175945	42.718479	-0.239829577	0.050807965	0.0613053157485	-
YNL334C	41.954319	50.45813	-0.266267403	0.0508742661908	0.0613733860304	-
tR(UCU)B	1.772627	0.16871	3.393271599	0.0509494255266	0.0614521145656	-

YLR424W	6.066108	6.001694	0.0154014195668	0.0512536951036	0.0618070977474	-
YCR075W-A	7.405584	5.798293	0.352985272	0.0513207349587	0.0618759220477	-
YOL071W	212.042084	245.789627	-0.213073406	0.0513989512714	0.0619581921346	-
YLR394W	11.99789	14.711876	-0.294200515	0.0516421803313	0.0622393041151	-
YPR182W	235.794113	248.971191	-0.078451115	0.0516458888963	0.0622316922104	-
YGL076C	233.624298	268.363617	-0.199998765	0.052031601	0.0626842958917	-
YPR053C	12.775482	16.742607	-0.390146465	0.0521665817657	0.0628347185878	-
YJL127C	17.094471	20.420038	-0.256455773	0.0524623021659	0.0631786560321	-
YGR027C	498.031281	536.860718	-0.108311487	0.0528389267195	0.0636198714264	-
YCL029C	10.445075	12.959418	-0.311178077	0.0530235811932	0.0638298219987	-
YBR040W	0.356449	0.16476	1.113329642	0.0531507791271	0.0639581385581	-
YOL024W	0.634676	0.247526	1.358440216	0.0531507791271	0.0639581385581	-
YMR030W-A	1.103536	0.484822	1.186606623	0.0531507791271	0.0639581385581	-
YJL162C	4.274318	4.072341	0.0698358739404	0.0534828259444	0.0643327617638	-
YPR094W	19.751284	25.629847	-0.375878423	0.0536490282704	0.0645201796789	-
YOR009W	6.69306	6.520223	0.0377446400913	0.0538885228494	0.0647956517237	-
YML096W	11.874852	12.008124	-0.016101247	0.0539812785514	0.0648946121702	-
YCL055W	7.245455	6.890828	0.0723989437883	0.0544932355644	0.0654973874896	-
YOL118C	22.351757	21.1548	0.0794031951527	0.0547743135161	0.065822481	-
YCR085W	1.260241	0.648151	0.959297789	0.054811855	0.065854847	-
YDR201W	37.95536	38.397423	-0.016705854	0.055434162	0.0665896441092	-
YHR193C	357.789398	410.216644	-0.19727539	0.0557844847179	0.066997501	-

YCR003W	8.540183	7.849289	0.121705006	0.0558234789955	0.0670313651072	-
YBL112C	4.364869	3.306823	0.400492529	0.0560251966631	0.0672605725942	-
YIL150C	13.009958	13.269093	-0.028453455	0.0560709900826	0.0673025341131	-
snR17b	1.302566	0.821639	0.664779907	0.0565858767219	0.0679074275448	-
YML107C	28.519489	29.393124	-0.043530569	0.0568311338082	0.0681885733826	-
YJR058C	68.436691	81.872177	-0.258603252	0.0577056831864	0.0692245181103	-
YOL075C	8.858913	10.54882	-0.251880033	0.05789352	0.0694364324266	-
YPR199C	75.310822	88.050179	-0.225468745	0.057945842	0.0694857620694	-
YNR029C	23.789688	28.430624	-0.257109413	0.0579599582653	0.0694892663018	-
YNL091W	59.777939	68.362679	-0.193595777	0.0582535690091	0.0698277961786	-
YCR079W	26.997726	32.107433	-0.250069431	0.0585563830219	0.0701772250579	-
RDN5-1	22.241184	31.187109	-0.487716232	0.0594817480065	0.0712724747393	-
YAR029W	9.797494	8.177556	0.26074305	0.0615927416107	0.0737876765571	-
YDR125C	24.219675	25.157593	-0.05481439	0.0634102351374	0.0759503646022	-
YLR285C-A	4.728726	3.189703	0.568029451	0.0635360291505	0.0760863558963	-
YMR244C-A	101.463066	120.939819	-0.253334664	0.0638985996865	0.0765057872428	-
YMR023C	9.694017	11.895061	-0.295196153	0.0656867416393	0.0786315642299	-
YGR223C	53.651821	57.036972	-0.088270255	0.0656925090038	0.0786233074794	-
snR31	17.216766	15.56555	0.145457618	0.065728321	0.0786510059711	-
YNL145W	18.237406	15.208405	0.262031686	0.0660265320225	0.0789926210822	-
YMR181C	461.939209	500.734589	-0.116343109	0.0660318855559	0.078983805	-
YML015C	24.657511	25.391245	-0.042303959	0.0662822441323	0.0792679968355	-

YDR089W	8.08729	8.264006	-0.031184956	0.0666069402386	0.0796409639833	-
YGR187C	14.410584	17.577293	-0.286584102	0.06740359	0.0805779872497	-
YJL180C	18.953375	23.034594	-0.2813474	0.0676301921609	0.0808333140036	-
YOR022C	13.718488	16.378527	-0.255684132	0.0676917920282	0.0808913657191	-
YBR262C	227.264023	241.750229	-0.08914794	0.0679261290938	0.0811557754838	-
YMR160W	43.302685	46.421379	-0.100332898	0.068079841	0.0813237733375	-
YOR269W	3.059542	4.051424	-0.405113374	0.0681679928477	0.0814134089262	-
YBL018C	37.268463	45.780396	-0.296774621	0.0681971605019	0.0814325778795	-
YDL028C	21.310938	22.434446	-0.074121464	0.0683222038247	0.0815662002584	-
YMR105W-A	8.248164	6.557191	0.3309951	0.0684033706083	0.0816473994879	-
YBL008W-A	0.788788	1.81574	-1.202848126	0.0684115062682	0.0816414130936	-
YPL247C	105.888176	121.450943	-0.197832192	0.068485632	0.0817141651681	-
YBL021C	62.624207	74.966148	-0.259518844	0.0685796724334	0.0818106468998	-
YHR213W-A	3.52009	2.397267	0.554221715	0.0687132038791	0.0819541920618	-
YHL048C-A	0.479788	1.698346	-1.823661426	0.0690330639245	0.0823198733851	-
YHR200W	304.747223	331.051453	-0.119442388	0.0691266461174	0.0824156364695	-
YDR034C-C	24.398891	29.044094	-0.251429253	0.06950932	0.0828559640031	-
YFL058W	1.95404	2.821671	-0.530089783	0.0695339174758	0.0828693721807	-
YOR072W	4.558767	6.844265	-0.586251945	0.06994602	0.0833445092126	-
YHL049C	5.504576	5.072584	0.117910591	0.0699778821672	0.0833664733245	-
YNL222W	10.914235	10.430674	0.065378629	0.0702927734364	0.0837255444154	-
YMR204C	23.518858	28.054199	-0.254398712	0.0708023161614	0.0843162821274	-

YNL292W	9.019876	8.915177	0.0168441591955	0.071271666	0.0848589381216	-
YJL011C	6.914221	9.360274	-0.436984045	0.0717070571323	0.0853609608202	-
YLR141W	27.554947	28.578869	-0.052637471	0.0720125438223	0.085708181	-
YBR249C	46.880974	54.97578	-0.229793625	0.0727936759241	0.0866212651211	-
YGL246C	21.4702	22.109837	-0.042352759	0.0730034709526	0.0868542636401	-
YEL076C-A	0.760872	0.458578	0.730486628	0.0730131675778	0.0868491558389	-
YKL096C-B	7.752942	5.932964	0.385990843	0.0730535235848	0.086880512	-
YOR239W	31.761944	33.66272	-0.083852538	0.0750246288975	0.0892076031786	-
YHR036W	10.032478	10.082085	-0.00711603	0.0759400742644	0.0902788167433	-
YBL014C	9.423763	11.289927	-0.260660996	0.0761293622108	0.090486517	-
snR55	17.160217	13.947833	0.299026801	0.0764496667954	0.0908498337416	-
YKL098W	62.569462	72.88739	-0.220210541	0.0768061367241	0.0912559802456	-
snR70	62.297611	61.33633	0.0224349926565	0.0771090296219	0.0915983268069	-
YAL001C	22.500391	24.012072	-0.093809827	0.0772141243818	0.0917056216113	-
YOR291W	5.34725	6.419948	-0.263764483	0.0774109308585	0.0919217786476	-
YDR344C	3.445789	5.127793	-0.573503662	0.0774142656452	0.0919081585629	-
YDL178W	36.07893	38.220577	-0.083193005	0.0776439504116	0.0921632206798	-
YOL052C	20.243849	24.269011	-0.261631699	0.078059557	0.0926388324366	-
YNL163C	9.815838	11.658001	-0.248137086	0.0786704473796	0.0933459742609	-
YOR015W	24.24757	23.740292	0.0305024931909	0.0791095808331	0.0938490880588	-
YJR116W	17.822304	17.979052	-0.012633097	0.0792109946498	0.0939514434058	-
snR69	13.88417	20.647753	-0.572543853	0.0794289108108	0.0941919156281	-

YLR012C	0.12022	0.543246	-2.175928722	0.0794594644015	0.0942101518343	-
YGL258W	2.134868	1.668353	0.355722295	0.0795487878798	0.0942980475765	-
YNL211C	44.755108	55.347191	-0.306457749	0.080591282	0.0955155934756	-
YLR090W	46.736118	49.661106	-0.087578486	0.0808869021174	0.0958476595428	-
YHR128W	13.178373	16.563631	-0.329846702	0.0810695846406	0.0960457979865	-
YNL333W	54.088425	63.43605	-0.229983053	0.0813456686022	0.0963544954555	-
YKL138C	134.541946	142.325958	-0.08114278	0.082056808	0.0971783024633	-
YCR041W	1.550458	0.930839	0.736090883	0.0823693553389	0.0975298413015	-
YJL147C	12.178399	12.265018	-0.010224865	0.0826915175588	0.0978926279568	-
YGR030C	42.17271	50.922089	-0.271981873	0.0827147415951	0.0979014520157	-
YOR293C-A	0.145245	0.728827	-2.327087906	0.0830729664787	0.0983067052801	-
YBL089W	10.365987	12.698598	-0.292811734	0.0834355443543	0.0987169543265	-
tP(UGG)M	2.577702	1.045082	1.302469343	0.0845074620239	0.0999566207953	-
tV(AAC)J	2.6199	0.83215	1.654596235	0.0845074620239	0.0999566207953	-
YKR035W-A	161.559448	173.382751	-0.101895257	0.0851227499318	0.100655625	-
YDL239C	2.951475	2.851606	0.0496614601022	0.0856410476446	0.101249214	-
YLR287C	34.280937	40.50396	-0.240656421	0.085946693	0.101591217	-
YDR391C	69.460785	73.375534	-0.079100382	0.0869258682241	0.102729067	-
YLR367W	112.614311	118.689354	-0.07580036	0.0871770033374	0.10300625	-
snR83	68.221649	70.096756	-0.039118052	0.0874025930504	0.103253149	-
tV(AAC)G2	15.572192	23.580626	-0.598629981	0.087860369	0.103774195	-
YKL063C	70.975166	83.831749	-0.240182411	0.0890636584907	0.105175422	-

YDL027C	96.595451	111.081238	-0.201588007	0.089243724	0.105368018	-
YBR184W	5.148432	5.031569	0.0331247663404	0.089700098	0.10588671	-
YHR117W	26.338535	27.862352	-0.081141946	0.0897365529168	0.105909605	-
YGL216W	11.87196	12.348825	-0.056815639	0.0905130122042	0.106805698	-
YPL096C-A	36.851009	46.484871	-0.33505713	0.0909202996818	0.10726591	-
YHR187W	34.14666	40.454456	-0.244554152	0.0915303728245	0.107965144	-
snR58	1.206797	3.288086	-1.446065018	0.0917482112039	0.108201538	-
YHR213W-B	7.482815	10.48504	-0.486679353	0.0926017773448	0.109187433	-
YBR042C	10.460011	10.5195	-0.008181765	0.0929938329898	0.109628886	-
YDR414C	5.531173	7.089533	-0.358105131	0.0931762195996	0.109823044	-
tM(CAU)P	2.112022	0.517846	2.028029833	0.093966588	0.110712579	-
YCL026C-A	0.235735	0.086964	1.438675769	0.093966588	0.110712579	-
tL(CAA)M	1.807725	0.59233	1.609702163	0.093966588	0.110712579	-
YBR085C-A	2539.301514	2788.421387	-0.135016889	0.0944834438703	0.111279306	-
tC(GCA)P1	1.32487	0.260094	2.348745781	0.0945125821094	0.111281955	-
tD(GUC)B	1.281934	0.011716	6.773698079	0.0945125821094	0.111281955	-
YDR415C	22.023329	22.814978	-0.050948702	0.0946086932838	0.111363437	-
YOL132W	1.705572	1.488245	0.196643612	0.0948790401806	0.111660489	-
YFR032C-B	3.413764	5.305416	-0.636102552	0.0952844437904	0.112116343	-
YLL056C	42.148357	49.662399	-0.23667756	0.0957561110779	0.112649979	-
YLR022C	15.147738	18.669889	-0.301610977	0.0959543804134	0.11286184	-
YGR224W	5.522609	5.485545	0.00971502598115	0.0973945976794	0.114534128	-

YOR316C-A	2.070723	3.632886	-0.810981517	0.0991486946391	0.116574829	-
YIL054W	2.032208	3.352977	-0.722394515	0.0992087636464	0.116623368	-
YDR503C	7.828241	7.566587	0.0490454694222	0.0996052745065	0.117067312	-
YER073W	14.77964	15.281144	-0.048141424	0.100081353	0.117604589	-
YPL157W	21.50584	22.146553	-0.042353685	0.100286943	0.117823872	-
YDR106W	4.345469	4.014704	0.11421827	0.10081421	0.11842093	-
YOR152C	28.396395	29.319637	-0.046159455	0.10244147	0.120309619	-
YNR055C	30.933189	36.071537	-0.221705324	0.1034438	0.1214638	-
YML043C	3.686955	4.721885	-0.356933097	0.103690306	0.121730223	-
YAR033W	18.418613	18.60607	-0.014608935	0.103763241	0.121792815	-
YPL210C	27.997337	29.724087	-0.086342887	0.104158613	0.122233775	-
YDR139C	199.040604	210.567245	-0.081218265	0.104266214	0.122336923	-
YNL167C	20.824295	21.955198	-0.076294891	0.104486212	0.122571883	-
YIL001W	15.19123	15.746367	-0.051780322	0.104686939	0.122784153	-
YLR438C-A	292.557953	313.299896	-0.098821853	0.105044937	0.123180765	-
YAR023C	16.441198	16.278318	0.0143637888388	0.10589657	0.124155978	-
YPR067W	58.959347	69.614601	-0.239669383	0.106804691	0.12519704	-
YGR177C	3.271619	3.133409	0.0622716522203	0.106965324	0.125361663	-
YGR204C-A	18.313162	15.706298	0.221537737	0.107448545	0.125904221	-
YKL219W	7.091349	7.004907	0.0176942030536	0.107909512	0.126420503	-
snR46	7.912604	11.481693	-0.537110924	0.107994023	0.126495641	-
YMR021C	32.74868	38.424553	-0.23059172	0.108861445	0.127487616	-

YKR101W	14.939152	17.679682	-0.242994068	0.110175295	0.129001929	-
YPL096W	42.519451	45.033955	-0.082890215	0.113090772	0.13239063	-
YLR250W	322.923309	351.620117	-0.122826033	0.113186007	0.132477137	-
YJL223C	0.141801	0.513241	-1.855768717	0.11387433	0.133232536	-
YIL176C	0.141801	0.513241	-1.855768717	0.11387433	0.133232536	-
YJR005C-A	0.21768	0.669455	-1.620778221	0.11387433	0.133232536	-
YLR342W-A	1.231807	0.535507	1.201798893	0.114369466	0.133748827	-
snR47	2.032657	0.857183	1.245691645	0.114369466	0.133748827	-
YDR508C	41.571484	44.603523	-0.101563416	0.11479258	0.134205714	-
YML016C	11.506421	11.947567	-0.054277696	0.116787326	0.136512091	-
YPR118W	25.182295	26.394363	-0.067820079	0.118247491	0.138192851	-
YNL025C	19.392805	23.271206	-0.263024487	0.118513813	0.138478025	-
YLR063W	6.7733	6.645449	0.0274922178449	0.118850865	0.138845723	-
YDR065W	8.078864	10.034398	-0.312729717	0.119016398	0.139012944	-
YEL069C	7.540771	7.64586	-0.019966746	0.120278748	0.140460961	-
snR189	3.237013	2.155738	0.58648131	0.120303117	0.140462996	-
YML007C-A	3.822097	2.454653	0.638845299	0.121089787	0.141341618	-
snR87	3.994868	2.365034	0.75628691	0.121089787	0.141341618	-
YNL147W	76.27816	79.623322	-0.061921021	0.121114642	0.141330771	-
YDL059C	6.265056	8.120428	-0.374228364	0.121250602	0.141462834	-
YML011C	26.02021	31.5914	-0.279899267	0.121564751	0.1418027	-
YLR154C-H	3.969016	6.623636	-0.738842014	0.121890204	0.142155624	-
YOR306C	10.061828	12.144634	-0.271426579	0.12216307	0.142447096	-
YLR392C	32.77401	38.186733	-0.220519296	0.122948365	0.143335859	-
YLR387C	66.752426	71.740028	-0.103958038	0.123302625	0.143721873	-

YOR295W	15.187256	18.655336	-0.296727114	0.123743808	0.14420904	-
YCR016W	5.281943	6.801734	-0.364833854	0.123843746	0.144298419	-
YIL064W	64.758667	75.4785	-0.220992463	0.123882392	0.144316362	-
YGL160W	32.622887	37.919445	-0.217053388	0.124708793	0.145251817	-
YJL108C	8.326797	10.272274	-0.302922032	0.125382148	0.146008699	-
YBL072C	396.291229	450.569031	-0.185187119	0.125512045	0.146132554	-
YJR135C	33.78091	40.120384	-0.248127222	0.126285501	0.147005511	-
YMR277W	9.068506	10.837329	-0.257072432	0.12637075	0.147077169	-
YNL146W	10.958961	10.127048	0.113897331	0.126722194	0.147458553	-
YDR247W	68.791588	79.149773	-0.202353053	0.127101622	0.147872352	-
YMR273C	9.677196	11.452319	-0.242978774	0.127702954	0.148544115	-
YGL222C	180.034531	193.884735	-0.106925576	0.128330616	0.149246247	-
YPR046W	3.870809	5.29675	-0.452472295	0.128802719	0.149767237	-
YKL014C	2.425272	2.462973	-0.022254255	0.129684034	0.15076376	-
YJL004C	97.461159	113.071465	-0.214335609	0.130562656	0.151756781	-
YGL111W	25.704145	27.115147	-0.077097967	0.131223439	0.152496276	-
YNL207W	24.289396	25.507402	-0.070589349	0.131777486	0.153111479	-
YPL261C	37.114456	37.546959	-0.016714843	0.132389189	0.153793429	-
YOR161C	26.864325	28.488127	-0.084669183	0.133760941	0.15535789	-
YDL227C	2.51156	2.388093	0.0727247198665	0.134105807	0.155729302	-
YLR382C	17.351215	18.433174	-0.08726782	0.134589078	0.156261268	-
YJL003W	103.628899	109.408134	-0.078293616	0.134801305	0.156478403	-

YKR068C	64.690224	75.811913	-0.228876861	0.134865532	0.156523691	-
YHR210C	5.441952	5.275556	0.0448010815815	0.134874434	0.156504764	-
YLR360W	32.772575	34.750145	-0.084529965	0.135408359	0.157094953	-
YDR302W	27.898321	33.404812	-0.25987764	0.13547596	0.157144013	-
YML030W	131.410828	152.180542	-0.21169975	0.13631861	0.158091898	-
YNL215W	123.019218	140.962296	-0.196425618	0.137460559	0.159386468	-
YOR394C-A	0.163418	0.777773	-2.250782252	0.137661278	0.159589396	-
YJL171C	105.391365	120.650383	-0.195075827	0.138263354	0.16025745	-
YOR320C	20.034819	23.616869	-0.237308236	0.138345235	0.160322422	-
snR75	3.432091	1.81221	0.92133766	0.139243839	0.161318607	-
YBR298C-A	1.330175	0.728036	0.869534366	0.139243839	0.161318607	-
YMR097C	37.970802	44.327911	-0.223324904	0.140058352	0.162216838	-
snR50	2.61212	1.164098	1.166008658	0.143354827	0.166003873	-
YBL108C-A	2.160835	4.122363	-0.931882636	0.143718701	0.166394199	-
YDL005C	116.293335	132.784195	-0.191315021	0.143736095	0.166383307	-
snR73	5.313367	3.698885	0.522535918	0.144573876	0.167321891	-
YEL035C	4.763537	4.266239	0.159068405	0.144710676	0.167448998	-
YCL059C	18.320324	18.915377	-0.046114513	0.145237584	0.16802738	-
tL(CAA)G3	0.878141	2.549959	-1.537949539	0.145595505	0.168410079	-
YGR179C	17.476009	18.187368	-0.057561023	0.145652618	0.168444756	-
YBR257W	56.877571	66.18811	-0.21871222	0.146147161	0.168985207	-
YKR006C	88.943848	102.681519	-0.207209817	0.148679114	0.171880808	-
YIR042C	38.835934	40.762779	-0.069860247	0.149341333	0.172614223	-
YCR061W	12.925556	13.520995	-0.064974982	0.149390485	0.172638892	-

YAR031W	24.441492	25.462496	-0.059041493	0.149473044	0.172702151	-
YDL148C	7.396993	7.653591	-0.049197896	0.150120855	0.17341836	-
YPR184W	79.21067	89.608887	-0.177947039	0.150823038	0.174197102	-
YMR154C	9.467804	11.26154	-0.250302382	0.152060894	0.175594128	-
YNL318C	0.131154	0.25821	-0.977283066	0.152939933	0.176576363	-
YDR157W	2.45978	3.645157	-0.567451666	0.152945423	0.176549867	-
snR68	2.249248	4.096177	-0.864835317	0.154132998	0.177871112	-
snR67	3.768159	6.661984	-0.822092047	0.154132998	0.177871112	-
YER129W	11.647228	12.3634	-0.086088907	0.155700673	0.179630139	-
YLR065C	35.118217	41.836655	-0.252547908	0.156747923	0.180804739	-
YJL071W	9.609467	9.923761	-0.046430578	0.157247971	0.181347837	-
YPR064W	6.271752	8.343106	-0.411716062	0.157432438	0.181526853	-
YOR357C	41.282597	49.060078	-0.249015797	0.158117699	0.182283134	-
YNL249C	8.521965	10.269969	-0.269173795	0.158554795	0.182753095	-
YKL132C	2.38299	3.120677	-0.389086144	0.159874074	0.184239512	-
tX(XXX)L	5.297958	3.498991	0.598497453	0.163773652	0.188698373	-
YBR085W	0.899019	0.694194	0.37301271	0.164321718	0.189294716	-
YHR175W-A	14.05049	18.992235	-0.434789247	0.164466934	0.189426851	-
YPR176C	32.152554	37.692688	-0.229351342	0.164511955	0.189443557	-
YBR258C	14.136748	17.729483	-0.326700185	0.164739147	0.189669997	-
YGL029W	7.728204	10.193703	-0.399473143	0.164762576	0.189661797	-
YJL043W	6.98381	8.776838	-0.329686969	0.165603119	0.190594024	-
YAL068C	0.619516	1.196771	-0.949933674	0.166509187	0.191601304	-

YCL047C	6.187084	7.851632	-0.343732935	0.166530416	0.191590219	-
YPL189W	1.548658	2.037886	-0.396054769	0.166779915	0.191841711	-
YER146W	24.988091	30.895699	-0.306165325	0.166819318	0.191851487	-
YCR014C	2.825312	3.563165	-0.334749099	0.169541378	0.194945893	-
YDL245C	7.342937	8.866792	-0.272055011	0.169961469	0.195392741	-
YGR233C	14.687272	17.018856	-0.212567607	0.170286891	0.195730609	-
YJR152W	18.991514	22.250273	-0.228468116	0.170634517	0.196093869	-
YNL146C-A	1.361106	0.718964	0.920787986	0.171021347	0.196483857	-
YBL005W-A	0.184234	0.10578	0.800472452	0.171021347	0.196483857	-
YLR368W	15.671524	18.407497	-0.232147983	0.172649406	0.198299263	-
YBR293W	15.119941	17.904915	-0.24390316	0.173233144	0.198932919	-
YOL033W	6.19031	6.286355	-0.022212087	0.173244502	0.198909167	-
YER159C-A	0.386049	0.283201	0.446957616	0.174020526	0.199763207	-
YDR461W	80.026642	80.675247	-0.011645716	0.174099501	0.199816917	-
YOR029W	1.887852	1.388844	0.442861108	0.176878932	0.202969392	-
YGL054C	257.96167	279.362152	-0.114979863	0.178880785	0.205228593	-
YIL103W	19.373945	22.817022	-0.235992757	0.180599243	0.207161881	-
YLL058W	12.535235	13.128813	-0.066747443	0.180891052	0.207458277	-
YHR100C	42.47023	44.605022	-0.070754228	0.182511633	0.209278208	-
YCR096C	39.418247	40.618019	-0.04325626	0.182999767	0.209799179	-
YHL018W	8.007616	7.490626	0.0962865009716	0.184139986	0.211067399	-
YLR157W-D	3.755543	5.644147	-0.587734046	0.184203167	0.211100842	-

YDR211W	17.583929	20.479992	-0.219957686	0.185445554	0.212485419	-
YBR182C-A	2.614259	4.070667	-0.638863128	0.185758739	0.212804992	-
YGL235W	1.007059	0.725277	0.473544204	0.186619426	0.213751549	-
YPL034W	20.805956	21.231024	-0.029177377	0.187196473	0.214372939	-
YNR004W	27.819929	33.525063	-0.269121305	0.187680113	0.214887153	-
YNL128W	1.923176	2.531812	-0.396679484	0.189256791	0.216632463	-
YOR376W	6.783925	8.998318	-0.407535132	0.189256791	0.216632463	-
YDR487C	85.890678	92.028694	-0.099582196	0.189437444	0.216779291	-
YKL003C	135.851578	157.083405	-0.209499452	0.190311894	0.217739815	-
YER174C	80.557091	86.49469	-0.10259998	0.190766141	0.218219311	-
YMR067C	46.147102	49.528419	-0.102016519	0.190801661	0.218219733	-
YFL049W	28.534555	32.981701	-0.208955742	0.190951111	0.218350433	-
YFR020W	13.356313	16.271454	-0.284821367	0.19113034	0.218515129	-
YKL097C	0.681239	1.203546	-0.821058348	0.191636056	0.219052962	-
YNL261W	3.724867	4.650887	-0.320316974	0.192916958	0.220476524	-
YPR047W	12.581147	13.110315	-0.059438893	0.193356449	0.220938125	-
YAL012W	12.275513	12.700225	-0.04907074	0.194421074	0.222113734	-
YIR017C	44.331718	52.133751	-0.233878392	0.194538672	0.222207189	-
YCR022C	1.338524	0.923748	0.535071766	0.194947882	0.222633637	-
YDL240W	6.750782	7.975161	-0.240459012	0.195314998	0.223011865	-
YHL017W	13.340116	15.741404	-0.238793011	0.195466493	0.2231438	-
YHR116W	46.597065	48.857426	-0.068338772	0.195718794	0.223390747	-

YJR076C	36.881325	42.722404	-0.212102343	0.197025981	0.224841414	-
YBR193C	69.393349	74.185654	-0.096342831	0.197774877	0.225654556	-
YDR400W	12.685913	13.077573	-0.04386747	0.199011534	0.227023815	-
YNR062C	3.244704	4.193058	-0.369915913	0.200680346	0.228885466	-
YCL004W	8.538897	8.823656	-0.047326823	0.200792815	0.228971674	-
YJL203W	31.85293	33.661728	-0.079683153	0.204152981	0.232760644	-
YGR217W	3.919575	4.12934	-0.07521398	0.204504684	0.233118818	-
YGL190C	71.163452	81.263214	-0.19146593	0.205000448	0.233641046	-
YLR223C	10.049844	11.71985	-0.221780998	0.205744826	0.23444638	-
YGL194C-A	18.602093	18.095699	0.0398181170161	0.20611699	0.234827358	-
YBR270C	16.662785	19.520599	-0.228367772	0.206890204	0.235665025	-
YER078W-A	4.277946	3.391581	0.334960323	0.207585964	0.236414175	-
YOL133W	253.97081	290.695038	-0.194843755	0.208667787	0.237602646	-
YDR374C	0.228084	0.428278	-0.908982324	0.209410094	0.238404156	-
YNL081C	120.072456	138.644394	-0.207484041	0.2101879	0.23924578	-
YDR310C	23.382074	26.836567	-0.198797227	0.212110063	0.241389417	-
YBL071C	25.58223	25.849195	-0.014977323	0.212187741	0.241433558	-
YJL210W	136.194946	155.442825	-0.190710858	0.212528253	0.24177669	-
YHL038C	4.925589	5.974897	-0.278617591	0.21500278	0.244546951	-
YIR030C	3.461597	3.264479	0.0845850147494	0.215574236	0.245152016	-
YER007W	13.209461	13.872028	-0.070607116	0.215589587	0.245124571	-
YPL017C	35.816433	41.32605	-0.206429811	0.215779274	0.245295319	-

YER177W	949.634155	1047.418579	-0.141394371	0.217020711	0.246661401	-
YJR158W	4.754361	5.787836	-0.283772592	0.217355169	0.246996318	-
YFL003C	3.702356	4.473318	-0.272901696	0.21798236	0.247663707	-
YLR039C	10.381372	12.089969	-0.219813423	0.218848822	0.24860265	-
YDR308C	50.631466	59.576534	-0.234709937	0.219892646	0.24974269	-
YLR274W	10.297587	10.872223	-0.078340638	0.220583552	0.250481561	-
YAR035C-A	3.68953	2.293205	0.686071717	0.221899672	0.251906952	-
snR39B	3.152699	1.901501	0.729448739	0.221899672	0.251906952	-
YJL025W	4.002622	4.922616	-0.298479826	0.222593141	0.252624903	-
YJL129C	9.272977	10.78524	-0.217953799	0.222632566	0.252623464	-
YPL062W	0.33258	0.746073	-1.165615385	0.223908331	0.254024657	-
YBL102W	165.174606	179.341217	-0.11871519	0.223937158	0.254010941	-
tP(AGG)N	6.953522	5.344823	0.379601724	0.224271157	0.254320092	-
YEL075C	1.331878	0.978152	0.445331362	0.224271157	0.254320092	-
YNL024C	1.768082	2.448912	-0.469955747	0.225805075	0.255989391	-
YDR284C	31.256098	33.126286	-0.083838779	0.226499321	0.256729557	-
YMR223W	6.654394	6.813687	-0.034128387	0.226506621	0.256690965	-
YML114C	31.113457	35.929153	-0.207616225	0.226660024	0.256817928	-
YMR060C	17.659395	20.883678	-0.241939901	0.229033007	0.259459289	-
YML001W	103.954742	112.158714	-0.109586143	0.231654277	0.262380907	-
YBR108W	28.765762	32.929516	-0.19502862	0.231717361	0.262404484	-
YDR514C	5.726301	6.945107	-0.278393414	0.233132696	0.263959106	-
YJL052C-A	5.245503	4.224777	0.312205929	0.234133988	0.265044456	-
tC(GCA)G	0.57043	1.778484	-1.640526232	0.234423407	0.265323703	-

YJR100C	55.463066	63.890648	-0.204077401	0.235345294	0.266318553	-
YGR121W-A	1.894618	1.398105	0.438434283	0.23644561	0.267514916	-
YOL031C	5.70308	6.954153	-0.286133538	0.236497154	0.267524478	-
YML125C	42.176983	48.852924	-0.211989014	0.236653948	0.267653072	-
YNL021W	10.020469	11.778353	-0.233187783	0.237464655	0.268521052	-
YHR121W	60.042492	69.74868	-0.216182058	0.237955569	0.269027167	-
YGL044C	19.260921	22.749517	-0.240159225	0.23879152	0.269923116	-
YPL024W	56.164425	65.024887	-0.211335382	0.240000807	0.271240672	-
YJL039C	6.131267	6.524718	-0.089730318	0.240916333	0.272225809	-
YIL165C	24.553846	25.115747	-0.032643165	0.243169377	0.274721654	-
YGR276C	33.473557	38.514278	-0.202371516	0.243398117	0.274930042	-
tS(AGA)J	2.93877	1.672697	0.81303632	0.244415249	0.276003613	-
YBL044W	0.632315	0.405544	0.640784994	0.244415249	0.276003613	-
YDL186W	4.768896	4.656312	0.0344675903592	0.245809118	0.27750191	-
snR45	14.985236	14.261871	0.0713785449384	0.246834099	0.278608379	-
YER074W-A	275.673645	298.026367	-0.112478625	0.248513998	0.280453536	-
YBR289W	22.50161	24.279579	-0.109715175	0.249194926	0.281170866	-
YGR198W	12.818472	14.904359	-0.217510031	0.251367235	0.283570378	-
snR85	5.826328	8.181936	-0.48985533	0.252066994	0.284308121	-
YLR445W	0.477452	0.81796	-0.776674593	0.252101104	0.284294941	-
snR74	4.827745	3.554411	0.441738964	0.253551715	0.285878868	-
snR78	9.245324	7.826588	0.240340378	0.254967447	0.2874229	-
YLR135W	12.7886	13.602432	-0.089006279	0.256457845	0.289050527	-
snR34	7.16822	6.47497	0.146741407	0.256519677	0.289067735	-
YDR079W	127.031532	136.280441	-0.101391871	0.257470274	0.290086291	-

YMR197C	266.862701	291.774994	-0.128758569	0.257619482	0.290201732	-
tN(GUU)N2	1.235351	3.077816	-1.316985973	0.25852248	0.291166102	-
YGR105W	87.299866	92.207169	-0.078899483	0.259158135	0.291829077	-
YOR085W	49.91531	57.432983	-0.202397109	0.25959609	0.292269229	-
YJL105W	2.449089	2.418175	0.018326548	0.260124595	0.292811149	-
YLR273C	17.868542	19.092295	-0.095568612	0.260159222	0.292797036	-
YPL280W	66.775848	71.807899	-0.10481616	0.260712274	0.293366285	-
YER153C	14.715002	15.26615	-0.053048534	0.261034846	0.293676028	-
YLR137W	7.637735	9.216084	-0.271009001	0.2631863	0.296042861	-
tV(AAC)E2	2.160373	1.108014	0.963304313	0.263683981	0.296548941	-
tT(UGU)H	1.337137	0.658437	1.022029975	0.264243004	0.297070006	-
snR38	1.058346	0.538096	0.97587587	0.264243004	0.297070006	-
YBR072C-A	0.635174	0.185373	1.776720731	0.264243004	0.297070006	-
YER085C	0.557528	0.956669	-0.778975588	0.264546085	0.297303058	-
YKL170W	226.218781	258.00235	-0.189665498	0.265034864	0.297798449	-
YOL060C	98.144753	111.063774	-0.17840528	0.265545652	0.298318387	-
YDR102C	3.235829	4.437627	-0.455653039	0.265651316	0.298383095	-
YPL021W	1.233257	0.995508	0.308968661	0.266775716	0.299591831	-
snR64	3.483431	2.482207	0.488885555	0.2670233	0.299815635	-
YOR348C	1043.149048	1167.654541	-0.162668197	0.267158151	0.299912803	-
YCR066W	3.963649	4.844187	-0.289425348	0.267489352	0.300230319	-
YAL007C	26.965599	28.373203	-0.073408942	0.267937895	0.300679402	-
YDL008W	71.811073	83.111649	-0.210844381	0.269617279	0.30250932	-

YBR196C-B	6.118235	8.862894	-0.534662337	0.269903276	0.302775486	-
YOR021C	96.784515	104.607758	-0.112141702	0.271378323	0.304375182	-
YGR283C	9.227347	9.51731	-0.044637953	0.273343842	0.306524309	-
YPR159C-A	2.603557	1.558085	0.740710052	0.274584495	0.30785995	-
YDR009W	27.337223	31.517447	-0.20528398	0.274768372	0.308010482	-
YHL041W	0.697111	1.146103	-0.717276407	0.2762244	0.309586762	-
YOR090C	47.883797	54.667702	-0.191151175	0.278857241	0.312481182	-
YGR091W	11.72666	13.783769	-0.233178267	0.281151887	0.314995653	-
YDR184C	10.133881	10.446097	-0.043777213	0.28147545	0.315301261	-
YPR019W	20.791388	23.851437	-0.198090115	0.281682429	0.31547619	-
YMR269W	9.658078	9.798702	-0.020854538	0.285145847	0.319297514	-
YOL094C	10.078431	12.042797	-0.256899445	0.286418154	0.320664364	-
YGL006W-A	14.440948	13.180651	0.131743826	0.286641553	0.320856611	-
YPL282C	1.500329	1.24175	0.27290415	0.28719357	0.321416565	-
YDL042C	19.470734	22.542498	-0.211340122	0.287217317	0.321385203	-
YHR214W	9.621542	11.761174	-0.289692045	0.287564843	0.321716083	-
YOL161C	0.32848	0.666009	-1.01973614	0.28795679	0.322096532	-
YOR188W	7.602068	8.097927	-0.091160708	0.290986745	0.325427078	-
snR54	0.523786	1.636094	-1.643206235	0.292754792	0.327345416	-
YDR087C	17.635912	18.521204	-0.070661703	0.293748516	0.328397404	-
tE(UUC)K	0.147208	0.759195	-2.366614414	0.29383761	0.328437862	-
YMR177W	2.141282	2.68186	-0.32475912	0.294629062	0.329263226	-
YGR003W	13.723386	15.899945	-0.21238529	0.295077877	0.329705447	-

YBR250 W	1.056113	1.395076	-0.401579513	0.295348265	0.329948178	-
YFL041 W-A	14.20554 8	13.69713 5	0.0525803273 428	0.29590147	0.330506716	-
YHL003 C	23.81228 4	25.43056 1	-0.094857282	0.297099547	0.331785211	-
YNR034 W-A	1148.336 06	1263.371 338	-0.13773384	0.298056115	0.33279359	-
YOR049 C	38.37298 6	41.28133	-0.105398419	0.298158279	0.332847797	-
YOL047 C	9.39683	9.731142	-0.050434974	0.299977102	0.334818024	-
YGR035 C	3.134141	2.775782	0.175175818	0.301903299	0.336907367	-
YOR006 C	48.42900 5	52.17559 4	-0.107503758	0.302463378	0.33747172	-
YGL039 W	9.856312	11.73299 2	-0.251451128	0.303410698	0.338467855	-
YMR267 W	63.40725 7	72.61812 6	-0.195681733	0.303488937	0.338494308	-
YML019 W	24.80222 3	26.40622 5	-0.090408637	0.303780037	0.338758123	-
YPL183 W-A	109.2229 39	126.4396 97	-0.2111736	0.303853196	0.33877885	-
YPR139C	50.68967 8	54.64967 7	-0.10852097	0.304808065	0.339782451	-
snR7-L	0.857812	0.50448	0.765864423	0.305780017	0.34080473	-
snR4	31.27689 9	38.06856 9	-0.283502859	0.305969509	0.340954714	-
YPL099C	80.12687 7	92.15400 7	-0.20176065	0.306574687	0.341567778	-
YGL057 C	9.012385	10.82516 1	-0.264407631	0.307368799	0.342391083	-
YOL096 C	40.64188 4	46.85876 5	-0.20535165	0.307818693	0.342830722	-
YDR115 W	124.5169 45	133.9270 17	-0.105104938	0.307877272	0.342834458	-
YGR239 C	57.57385 3	66.05197 1	-0.198187848	0.309964068	0.345096293	-
YPL097 W	19.42595 1	22.50560 4	-0.212299056	0.311366279	0.346595277	-
YIR001C	87.20427 7	94.55595 4	-0.11676941	0.313352922	0.348744163	-
YER157	5.227833	5.473184	-0.066167303	0.313557518	0.348909316	-

W						
tV(CAC)D	2.139554	3.522108	-0.719129056	0.313605104	0.348899729	-
YOR365C	4.2562	4.402433	-0.048735103	0.31721552	0.352853243	-
YNL169C	14.085031	16.420647	-0.221348234	0.31780568	0.353446374	-
YJR060W	90.828445	103.316765	-0.18585829	0.31803731	0.353640627	-
tS(AGA)M	0.556517	0.154308	1.850612899	0.319253409	0.354802213	-
tM(CAU)M	0.689658	0.082737	3.059276557	0.319253409	0.354802213	-
YLL066W-B	0.291144	0.135908	1.099102507	0.319253409	0.354802213	-
tR(ACG)O	0.693691	0.33742	1.039747678	0.319253409	0.354802213	-
tI(AAU)G	0.67636	0.214307	1.658112367	0.319253409	0.354802213	-
YMR259C	4.618242	4.904499	-0.086761997	0.319708452	0.355117207	-
YJL179W	33.193119	39.352348	-0.245565519	0.320151395	0.355545592	-
YOL115W	8.518926	10.000123	-0.231274282	0.320241812	0.355582395	-
YPL147W	88.343758	99.73008	-0.174900504	0.322588184	0.358123649	-
YCR024C-B	196.060867	212.025375	-0.112935328	0.324183912	0.359830812	-
YMR175W-A	15.048408	14.785043	0.0254724302583	0.325343039	0.36105284	-
YLR389C	12.189414	14.035252	-0.203426197	0.326868394	0.362680784	-
YIL008W	58.728539	68.690796	-0.226055055	0.327983003	0.363852481	-
YGL038C	58.513802	66.625214	-0.1872913	0.330154696	0.36619624	-
YKR082W	6.754607	7.842874	-0.215510591	0.331685168	0.367828067	-
YMR232W	1.106591	1.069077	0.0497563288326	0.33175956	0.367844856	-
YLR146W-A	19.412731	24.016613	-0.307029614	0.332839515	0.368976376	-
YER029	92.99246	100.7023	-0.114912286	0.332943603	0.369025868	-

C	2	93				
tG(GCC)P1	1.654532	1.002755	0.722454035	0.333054757	0.369050233	-
YJR151W-A	2.28605	1.276902	0.840209153	0.333054757	0.369050233	-
YMR264W	113.619621	123.417351	-0.119333239	0.333469531	0.369410929	-
YPL103C	8.210051	8.592215	-0.06563891	0.333715549	0.369617508	-
YGR271C-A	7.077925	7.178102	-0.020275949	0.333719897	0.36955639	-
YIL020C	12.32687	12.855651	-0.060596147	0.334557413	0.370417767	-
YJL085W	5.967576	7.049135	-0.2403012	0.335951809	0.371895297	-
YNR044W	8.711955	10.154276	-0.221018972	0.336265129	0.372175775	-
YDL129W	42.586399	49.014462	-0.202814743	0.336937552	0.372853533	-
tI(AAU)E2	0.942925	2.208297	-1.227719288	0.338230221	0.37421729	-
YGR213C	8.026526	8.300739	-0.04846408	0.340239071	0.3763728	-
YLR287C-A	1329.716919	1461.88501	-0.13671069	0.340856215	0.37698831	-
YPL045W	10.784673	12.485166	-0.21123257	0.343130979	0.379436608	-
YOR094W	17.88681	18.684317	-0.062931713	0.343143108	0.379382443	-
YMR082C	2.954464	2.63556	0.164786884	0.343830334	0.380074557	-
YDL107W	18.81778	21.91807	-0.220024329	0.345257297	0.381583995	-
YCR094W	24.168108	25.92351	-0.101156534	0.345408181	0.381682803	-
YFL040W	5.02103	5.989254	-0.254392972	0.345636436	0.381867057	-
YPR143W	40.458084	43.423641	-0.1020527	0.347602465	0.383970837	-
YBR003W	16.467394	17.595066	-0.095558662	0.347876973	0.384205701	-
YBL079W	11.018579	11.928041	-0.114418942	0.349245325	0.385648341	-
YKL221W	7.716192	9.116622	-0.240610317	0.351812279	0.388413769	-

YDR461C-A	428.03476	485.857422	-0.182805047	0.352628576	0.389245768	-
YNL153C	60.295921	69.894348	-0.213115389	0.353780875	0.390448297	-
YBR246W	4.165954	5.061587	-0.280942886	0.358420458	0.395498436	-
YGL041W-A	158.21582	180.187607	-0.187605926	0.358846969	0.3958987	-
YBR144C	1.454319	1.18204	0.299064897	0.359251741	0.396274841	-
YPL194W	8.130446	9.500505	-0.224669708	0.361383152	0.398555091	-
snR13	4.369933	6.29669	-0.526982482	0.362465013	0.39967723	-
YHR038W	26.020512	30.314709	-0.220368624	0.364780582	0.402159093	-
YGL022W	33.146244	36.071148	-0.12199994	0.364876161	0.402193042	-
YKR055W	46.023598	52.78867	-0.197854544	0.365222795	0.402503659	-
YGR146C-A	8.144588	7.613816	0.0972220155062	0.365647937	0.402900673	-
YNL277W-A	3.226109	2.806476	0.201035461	0.367246954	0.404554891	-
YCR001W	1.910897	1.654964	0.207450182	0.367246954	0.404554891	-
YLR221C	41.641811	44.720844	-0.102914603	0.369605178	0.40704434	-
YER028C	18.914022	21.923319	-0.213010046	0.370299699	0.407736881	-
YPL002C	53.765221	61.717777	-0.199012857	0.37058358	0.4079771	-
YOR175C	28.98774	33.11367	-0.191984054	0.371640293	0.409067897	-
tA(UGC)O	5.202065	7.499501	-0.527710179	0.372432724	0.409867461	-
YNL067W-B	0.768404	1.323356	-0.784264283	0.374781404	0.412379113	-
YLR158C	228.916214	251.895874	-0.138007839	0.378153089	0.416015307	-
snR77	18.096518	17.398209	0.0567733308164	0.378779492	0.416630596	-
YGR037C	470.551819	532.714294	-0.179008385	0.379069465	0.416875684	-
YMR075	21.25959	23.00505	-0.113836735	0.379140072	0.416879484	-

W	4	3				
YLR093 C	442.6270 45	497.8464 66	-0.169609285	0.379334517	0.417019423	-
YNL213 C	31.44243 6	36.48711	-0.214673891	0.380698995	0.418445354	-
YOR255 W	0.462377	0.678065	-0.552353943	0.381941498	0.419736736	-
YNR065 C	3.624857	4.245728	-0.228087867	0.383454227	0.421324574	-
YGL173 C	42.51371	46.71369 6	-0.135917433	0.383966086	0.421812328	-
YHR139 C-A	61.41583 6	71.33184 1	-0.215935508	0.386263329	0.424260925	-
YER038 W-A	0.356235	0.204906	0.797867038	0.387738734	0.425806131	-
YLR422 W	15.53758 9	16.96935 3	-0.127168904	0.387887789	0.425894481	-
YCR043 C	6.81181	6.746639	0.0138692268 734	0.389798657	0.427916899	-
YGR152 C	12.30164 3	12.91135 3	-0.069789177	0.390042566	0.428108955	-
tG(GCC) P2	0.603344	1.135821	-0.912682788	0.390321734	0.428301781	-
tK(CUU) E1	0.344829	1.238747	-1.844928549	0.390321734	0.428301781	-
YDR348 C	30.01586	34.33066 2	-0.193772673	0.390521	0.428406851	-
YDR114 C	12.49808 5	15.23010 3	-0.285218642	0.391436677	0.429335494	-
YIL097W	109.4529 11	120.1928 1	-0.135040271	0.392181045	0.430075945	-
YCR095 W-A	12.49136 4	15.79294 4	-0.338349111	0.393392869	0.43132867	-
YFR028C	12.15226 7	14.06807	-0.211198946	0.394349505	0.432301206	-
YDR093 W	5.417939	5.8241	-0.104290976	0.394964792	0.432899263	-
YOL123 W	34.54658 9	37.53874 2	-0.119837032	0.395005096	0.432867015	-
YKL194 C	2.949666	3.584569	-0.28124806	0.398167934	0.436256006	-
YOR190 W	12.37436 3	14.39695 6	-0.218409549	0.398459284	0.436498188	-
YMR079	64.13946	73.09767	-0.18861314	0.39868586	0.43666934	-

W	5	2				
YDL159W-A	3.209575	4.717486	-0.555635963	0.399204627	0.437160403	-
YHR159W	14.043524	16.243589	-0.209965427	0.40099675	0.439045471	-
YIL164C	20.574736	21.762171	-0.080948568	0.401420939	0.439432408	-
YHR201C	25.786633	29.649282	-0.201373758	0.401646915	0.439602265	-
YEL003W	40.750259	43.243965	-0.08568958	0.402067213	0.439984709	-
YDR045C	13.079388	15.807054	-0.273273477	0.402360473	0.440228026	-
YDR013W	13.990136	14.653007	-0.066786769	0.403005107	0.440855631	-
YFL065C	1.660672	1.464426	0.181431861	0.403853721	0.441706114	-
YBR128C	35.662823	40.852898	-0.196017515	0.404016679	0.441806508	-
YKL144C	15.257083	17.955328	-0.234932848	0.404709873	0.442486596	-
YGL034C	0.309297	0.615837	-0.993555708	0.404881356	0.442596137	-
YBR204C	61.781975	67.36042	-0.124715143	0.405589936	0.443292663	-
YGL015C	1.816686	2.503174	-0.462449496	0.40644765	0.444112825	-
YCL021W-A	1.874367	2.579298	-0.460575005	0.40644765	0.444112825	-
YLR156W	9.226241	11.312074	-0.29404858	0.407217708	0.444836799	-
YIL142W	161.274277	181.541412	-0.170782334	0.409661327	0.447427431	-
YHR171W	16.305691	17.601976	-0.110361814	0.410575548	0.448347054	-
YBR261C	18.922411	22.086033	-0.22303839	0.413042034	0.450961123	-
YDR478W	27.71254	32.148136	-0.214196143	0.413279452	0.451140995	-
YPL039W	22.846767	24.50338	-0.100990742	0.414885827	0.452814911	-
YGL007C-A	6.204722	5.451075	0.186825802	0.417335389	0.455408348	-
YLR303W	31.557705	36.075104	-0.193011263	0.417894523	0.455938348	-

YDR197W	31.7892	36.37991	-0.194605264	0.421996828	0.460333212	-
YNL010W	49.047153	53.071114	-0.113757436	0.423023263	0.461371823	-
YJL193W	3.199486	3.886188	-0.280515541	0.423398427	0.461699883	-
YCL034W	133.888565	146.967712	-0.134466488	0.424397231	0.462707764	-
YGR229C	34.062111	38.833729	-0.1891424	0.425872841	0.464235048	-
YKR023W	8.986974	9.560863	-0.089305419	0.428625888	0.467154058	-
tD(GUC)G2	1.171528	0.712331	0.717771754	0.429322404	0.46754381	-
tG(UCC)N	1.198108	0.67484	0.828140567	0.429322404	0.46754381	-
YMR242W-A	0.868526	0.44052	0.979361518	0.429322404	0.46754381	-
tR(UCU)G1	1.136771	0.463952	1.292894197	0.429322404	0.46754381	-
tP(UGG)N1	1.228483	0.584279	1.072148551	0.429322404	0.46754381	-
YKL224C	0.230309	0.114514	1.008046803	0.429322404	0.46754381	-
tT(AGU)N1	0.965925	0.42293	1.191492275	0.429322404	0.46754381	-
tH(GUG)E1	1.052944	0.527218	0.997957178	0.429322404	0.46754381	-
YKR091W	162.334793	178.027405	-0.133127098	0.430077663	0.467996868	-
YJL118W	9.958605	10.396108	-0.062027957	0.430440768	0.4683099	-
YDR052C	14.822046	17.009867	-0.198627253	0.430473472	0.468263416	-
YOR187W	320.547699	354.034637	-0.143351461	0.432078811	0.46992734	-
YOR394W	1.492597	1.339909	0.155689668	0.43228861	0.470073163	-
YMR325W	0.435735	0.319574	0.447300963	0.432542322	0.470143173	-
YOR192C-A	0.118134	0.094026	0.329292595	0.432542322	0.470143173	-
YOL038C-A	1.654554	1.077283	0.619045085	0.432542322	0.470143173	-

YDR210W-A	0.12983	0.087765	0.564906163	0.432542322	0.470143173	-
YKL090W	7.024861	7.40145	-0.075338254	0.434987489	0.472594037	-
YGR216C	9.911346	10.599775	-0.096880742	0.434995509	0.472520055	-
YEL009C-A	2.302647	2.162629	0.0905070687628	0.435465624	0.472947969	-
YNL282W	11.35102	11.827483	-0.059321144	0.436489087	0.473976605	-
YEL016C	15.587831	16.766827	-0.1051895	0.440160002	0.477879205	-
YNL097C-B	6.24496	5.626407	0.150478414	0.441890237	0.479673823	-
YFL050C	6.405508	7.425985	-0.21326941	0.443473644	0.481308458	-
YHR174W	607.057007	672.063049	-0.146764582	0.443570121	0.481329018	-
YDR169C-A	6.273014	8.179056	-0.382775559	0.44462249	0.482386651	-
YPL070W	27.029234	30.783182	-0.187621741	0.448473572	0.486479807	-
YDL104C	38.687859	42.068306	-0.120852833	0.450578693	0.48867794	-
YFR007W	21.652431	23.30484	-0.106100597	0.450693943	0.488717555	-
YFR012W	1.298764	1.206179	0.106695278	0.451188716	0.489168625	-
tN(GUU)P	2.140481	3.50415	-0.711129503	0.451714223	0.489610108	-
YOL160W	0.505327	0.756235	-0.581617355	0.451714223	0.489610108	-
tG(UCC)G	0.670613	1.441064	-1.103582054	0.452422847	0.490249788	-
YPL082C	7.803561	8.901975	-0.18999283	0.454124404	0.492007731	-
YER185W	12.486672	14.577497	-0.223354011	0.456311633	0.494291156	-
YOR157C	248.065109	279.326477	-0.171233501	0.456876221	0.494816395	-
YNL301C	25.479931	29.598213	-0.216148705	0.457734045	0.495658984	-
YKL154W	14.876796	17.359676	-0.222676173	0.458544871	0.496450394	-
YIL086C	15.524465	16.05201	-0.048210408	0.458798667	0.496638557	-

YML099C	11.554946	13.234885	-0.195835141	0.45916661	0.496950194	-
YML122C	1.685355	2.308802	-0.454091945	0.461113138	0.498969907	-
YOR056C	46.952339	53.301933	-0.182990824	0.46136655	0.499157117	-
YOR192C-C	1.897551	1.62054	0.22766403	0.461593318	0.499315442	-
YDR286C	29.14415	34.114605	-0.227183186	0.462503421	0.500212759	-
snR41	47.738705	49.433777	-0.050337709	0.464264732	0.502030213	-
YHR069C	50.329514	57.226711	-0.185284026	0.468529109	0.506553241	-
YLR270W	323.690826	357.424469	-0.143021926	0.46855112	0.50648883	-
YJR077C	1020.594788	1130.755737	-0.147877136	0.469823301	0.507775601	-
YHR086W-A	1.20674	0.868544	0.474444029	0.470153653	0.508044191	-
YCL010C	25.458637	27.395027	-0.105758843	0.474308093	0.512444247	-
YMR051C	0.247022	0.207323	0.25275936	0.475711285	0.513870829	-
YDL040C	15.969431	17.361597	-0.12058675	0.475882828	0.5139667	-
YLR157W-E	2.844455	2.606097	0.126261475	0.476259055	0.514283564	-
YIR026C	13.428709	14.375933	-0.098334976	0.47851465	0.516629382	-
YGR053C	20.636902	22.174658	-0.103685444	0.479448286	0.517547376	-
YGL050W	12.926167	15.047199	-0.219200422	0.479767317	0.517801722	-
YJR065C	261.633331	288.995819	-0.143502275	0.480030579	0.5179958	-
YGR252W	41.193638	44.94598	-0.125770543	0.482183526	0.520228594	-
YNL165W	16.890551	18.203259	-0.107980373	0.482786067	0.520788167	-
YHR109W	7.423776	8.604492	-0.212936839	0.483316946	0.521270257	-
YHR163W	17.339699	20.123718	-0.214818023	0.483626361	0.521513366	-

YMR252C	43.992584	47.264599	-0.103499672	0.486106994	0.524097294	-
YLR332W	82.223839	92.960587	-0.177062446	0.486197792	0.524104167	-
YOR292C	29.530825	31.954016	-0.1137756	0.487322901	0.525225793	-
YHL009C	37.467411	40.720245	-0.120109951	0.488442887	0.526341511	-
YOL086W-A	186.36879	203.278122	-0.125294671	0.488486782	0.526297456	-
YOR051C	22.874708	26.167461	-0.194020621	0.48918145	0.52695444	-
YDR428C	17.420334	18.654036	-0.098715522	0.493383719	0.531388988	-
YLL057C	9.187238	10.675565	-0.216609318	0.494809261	0.532831899	-
YGL118C	1.920759	2.517167	-0.390124421	0.496076798	0.534104191	-
YFR055W	6.616866	6.960314	-0.073004328	0.496186805	0.534129995	-
YNL140C	0.150903	0.261687	-0.794220769	0.496448359	0.5343189	-
YFR054C	1.196222	1.131434	0.0803327256532	0.496974912	0.534746556	-
YAR064W	2.305519	2.138398	0.108561162	0.496974912	0.534746556	-
YMR247W-A	17.821339	21.611271	-0.278178185	0.498905553	0.5366844	-
snR8	7.841525	9.823355	-0.325081583	0.499163169	0.536868495	-
YPL191C	16.378935	17.628548	-0.106072098	0.501001002	0.538751813	-
tQ(UUG)L	2.831705	2.401773	0.237571174	0.502348012	0.540059998	-
YDR365W-A	0.153692	0.133081	0.207737459	0.502348012	0.540059998	-
YPL184C	24.491961	27.842361	-0.184973264	0.503443691	0.541097374	-
YPL046C	73.687302	84.501717	-0.197564624	0.504369803	0.541998916	-
YOL120C	304.280396	335.168823	-0.13948657	0.504458802	0.542000735	-
YNL035C	12.704784	14.657285	-0.206246047	0.504893674	0.542374101	-
NME1	4.996405	4.994309	0.0006053398	0.50860196	0.546263143	-

			88634			
YLL004W	4.984705	5.800485	-0.218665407	0.50898035	0.546574989	-
YBR180W	1.780024	2.13974	-0.265538812	0.510163958	0.547751271	-
YHR074W	40.296425	44.192081	-0.133136017	0.510899126	0.548445751	-
YJR084W	35.964752	39.243885	-0.125884214	0.511866329	0.549389034	-
YAL063C-A	1.26973	1.113137	0.189890586	0.513752071	0.551317693	-
YDR069C	22.659674	25.665199	-0.179686341	0.516222296	0.553872799	-
YJR138W	8.352238	9.099371	-0.123603999	0.51692061	0.554526205	-
YDR119W	18.966255	20.680527	-0.124838112	0.517504564	0.555056727	-
snR63	5.748607	5.76194	-0.003342232	0.51925022	0.556832849	-
YLR297W	73.8881	80.205978	-0.118367738	0.519341699	0.556834761	-
YKL071W	6.662344	7.000257	-0.071378041	0.52030743	0.557773877	-
YFL025C	4.190896	4.849017	-0.210433591	0.521220407	0.558656126	-
YBR237W	28.189478	30.887075	-0.131846492	0.521441965	0.55879712	-
YPL072W	9.281351	9.961082	-0.10196764	0.521965611	0.559261738	-
YDR066C	23.681023	25.408396	-0.101573897	0.52371949	0.561044106	-
YPL175W	11.890465	13.679776	-0.202239471	0.527996086	0.565527898	-
YPR203W	0.330729	0.496422	-0.585917499	0.529583435	0.567130233	-
YPL166W	82.517296	93.530457	-0.18073969	0.535252745	0.573102629	-
YER087W	14.512252	15.732319	-0.116459932	0.536369967	0.57419982	-
YDR450W	268.580048	295.448517	-0.137554622	0.537917352	0.575757055	-
YJL028W	2.022354	2.63308	-0.380715801	0.541896899	0.579866577	-
YJR128W	1.880358	2.399898	-0.351965728	0.541896899	0.579866577	-
tE(UUC)	0.204455	0.365538	-0.838238047	0.543566389	0.580952078	-

G1						
tE(UUC)E2	0.147208	0.67484	-2.196689413	0.543566389	0.580952078	-
YCL075W	0.049069	0.114769	-1.225849236	0.543566389	0.580952078	-
snR53	0.155306	0.450511	-1.536448765	0.543566389	0.580952078	-
snR52	0.160009	0.484124	-1.597223562	0.543566389	0.580952078	-
YJL136W-A	0.241841	0.335411	-0.471871154	0.543566389	0.580952078	-
tR(UCU)M2	0.294416	0.630319	-1.098226075	0.543566389	0.580952078	-
tH(GUG)K	0.147208	0.400686	-1.444616026	0.543566389	0.580952078	-
tL(UAA)L	0.129683	0.600527	-2.211239741	0.543566389	0.580952078	-
tI(AAU)E1	0.021882	0.535768	-4.613791905	0.543566389	0.580952078	-
YBL071C-B	0.172486	0.33742	-0.968066221	0.543566389	0.580952078	-
tH(GUG)E2	0.276015	0.398343	-0.529264551	0.543566389	0.580952078	-
YCR067C	3.932905	4.53971	-0.207004802	0.544061149	0.580830888	-
YHR130C	0.963422	1.333109	-0.468554972	0.544768795	0.581486362	-
YBR242W	61.083828	69.331528	-0.182721081	0.545885798	0.582578482	-
YPL225W	481.909454	531.933472	-0.142483718	0.550277325	0.587164255	-
YOR227W	28.415663	31.254129	-0.137360421	0.550831339	0.5876544	-
YAL033W	17.228666	18.392288	-0.094289963	0.552079467	0.588884765	-
YDL045W-A	116.848717	132.946915	-0.186208407	0.55226555	0.588982053	-
YBR196C-A	0.770387	0.645597	0.254949484	0.555393439	0.592114455	-
YML045W-A	0.090217	0.062504	0.529450793	0.555393439	0.592114455	-
YOL103W-A	0.090207	0.074213	0.281567462	0.555393439	0.592114455	-
YML021C	12.376493	14.257236	-0.204091748	0.555824168	0.592370203	-
YHR144	1.680583	2.070334	-0.300901736	0.557842328	0.594419014	-

C						
YJL027C	2.02949	1.964641	0.0468515193374	0.5581746	0.594671	-
YNL038W	38.554565	41.91634	-0.12061106	0.5597454	0.596242183	-
YML009C	165.49054	180.42691	-0.124665777	0.562205061	0.598759482	-
YLR157C	34.200409	38.852146	-0.18398071	0.563557867	0.600097297	-
YGL084C	31.952742	36.166172	-0.178701164	0.566791257	0.603436824	-
YCR104W	1.023388	1.38762	-0.439259318	0.568074628	0.604699467	-
YMR071C	106.768852	116.943069	-0.131315532	0.568187261	0.604715672	-
YLR437C	34.893002	40.076996	-0.19983665	0.568199589	0.604625137	-
YGL184C	18.83638	20.486305	-0.121138064	0.568319482	0.604649074	-
YML049C	10.401709	11.799495	-0.181904533	0.568509036	0.604747103	-
YNL119W	19.686008	21.429003	-0.122394143	0.569773988	0.60598885	-
YPR109W	97.333603	109.768562	-0.173455056	0.572623663	0.608915331	-
YLR088W	18.066422	19.69919	-0.124825496	0.573794923	0.610056325	-
YCR059C	14.945091	17.224695	-0.204806756	0.574532153	0.610735549	-
YNL151C	29.386835	31.876352	-0.117316546	0.575410047	0.611564042	-
YIL171W	0.128695	0.02275	2.500017553	0.5762552	0.611520048	-
snR6	0.206354	0.177748	0.215288077	0.5762552	0.611520048	-
tI(AAU)L1	0.399848	0.232546	0.781935547	0.5762552	0.611520048	-
tI(AAU)N2	0.42173	0.262184	0.685740006	0.5762552	0.611520048	-
tV(AAC)O	0.381944	0.134512	1.505626245	0.5762552	0.611520048	-
YMR230W-A	0.222759	0.010553	4.399758631	0.5762552	0.611520048	-
tV(AAC)K1	0.419741	0.16187	1.374663761	0.5762552	0.611520048	-
tR(ACG)J	0.419441	0.046222	3.1818163	0.5762552	0.611520048	-

tK(UUU) G1	0.585128	0.216499	1.434391896	0.5762552	0.611520048	-
tW(CCA) M	0.337117	0.14544	1.212825276	0.5762552	0.611520048	-
tL(UAA) K	0.529247	0.074313	2.83225467	0.5762552	0.611520048	-
tG(GCC) O2	0.553584	0.11881	2.220145976	0.5762552	0.611520048	-
tL(CAA) N	0.353629	0.231834	0.609144428	0.5762552	0.611520048	-
tK(CUU) M	0.588831	0.055466	3.408178034	0.5762552	0.611520048	-
tI(AAU) D	0.39388	0.227986	0.788810928	0.5762552	0.611520048	-
tL(GAG) G	0.294416	0.255122	0.206668764	0.5762552	0.611520048	-
tT(AGU) D	0.312564	0.291198	0.102151177	0.5762552	0.611520048	-
snR191	3.4755	4.348898	-0.323429327	0.576284753	0.610559902	-
YAL018 C	0.949926	1.212365	-0.351937073	0.576284753	0.610559902	-
YBR186 W	3.598036	4.194357	-0.221240039	0.580454706	0.614820477	-
YDR352 W	28.437639	32.373028	-0.186990622	0.580520191	0.614784945	-
YDR468 C	58.559685	63.984543	-0.127815636	0.581025367	0.615214989	-
YJL172 W	241.301239	270.220551	-0.163302076	0.581742325	0.615869091	-
YPR195C	6.422719	6.599113	-0.039087943	0.584273884	0.618443695	-
YJR047C	3.808768	3.902395	-0.035035402	0.586550704	0.62074783	-
YMR176 W	11.37152	12.448808	-0.1305825	0.586948415	0.621062854	-
YOR143 C	5.084955	5.954053	-0.227637057	0.587909144	0.621973409	-
snR42	8.82282	10.484046	-0.248883832	0.589155601	0.623185887	-
YNR040 W	10.109793	11.72676	-0.214051006	0.592705072	0.626833576	-
YJR043C	3.815798	3.997607	-0.067151849	0.593458276	0.627523246	-
YOL097 W-A	0.902241	1.336074	-0.566415163	0.59390424	0.627834401	-
tL(CAA) G1	2.11616	2.936281	-0.472541328	0.59390424	0.627834401	-

YKL099C	23.152475	25.071009	-0.114853634	0.594346938	0.628141946	-
YDR530C	223.444366	250.687027	-0.16597167	0.594769655	0.628481705	-
YGR164W	0.403507	0.314825	0.358043593	0.596590474	0.630083985	-
snR72	1.535167	1.093171	0.489876512	0.596590474	0.630083985	-
tX(XXX)D	1.349895	1.09324	0.304237041	0.596590474	0.630083985	-
tK(CUU)G1	2.085107	1.677392	0.313901539	0.596590474	0.630083985	-
snR128	1.22089	0.941294	0.375215918	0.596590474	0.630083985	-
YPR005C	52.204201	59.066193	-0.17816672	0.598193046	0.631454247	-
YPR108W-A	7.390339	7.508719	-0.022926259	0.599831304	0.63307595	-
YLR082C	42.514698	48.090618	-0.177793776	0.601386338	0.634609279	-
YGR240C-A	6.454438	7.946152	-0.299964908	0.601817938	0.634956791	-
YML100W-A	4.251258	4.202234	0.0167333155529	0.605747285	0.638993908	-
YPL075W	11.062167	12.586467	-0.186239351	0.606716512	0.639907596	-
snR33	34.495159	40.163765	-0.219500605	0.607977955	0.641129122	-
YNL008C	40.480247	45.608654	-0.172089501	0.612289044	0.645565622	-
YBL100W-C	6.490635	6.184623	0.069673974	0.613879555	0.647132667	-
YJR135W-A	72.460228	82.716415	-0.190984314	0.614871819	0.648068634	-
YFL026W	8.277029	8.925352	-0.108796055	0.615934871	0.64907888	-
YML055W	46.175537	50.313534	-0.123817788	0.615951611	0.648986355	-
YHR192W	48.766045	53.409477	-0.131218785	0.618857289	0.651937222	-
YDR318W	22.656832	25.754396	-0.184872556	0.619322988	0.65231712	-
YLL062C	15.341766	16.611263	-0.114697208	0.619732244	0.65263745	-
YPL066W	46.816261	52.787571	-0.173188565	0.621852373	0.65475908	-

YDL202W	26.065588	29.707338	-0.188672929	0.625883032	0.658891277	-
YCL005W	14.176814	16.285646	-0.2000676	0.626742877	0.659684602	-
YJR003C	8.358098	9.042743	-0.113585787	0.627850615	0.660738535	-
YIL102C	7.700986	9.205716	-0.257486762	0.62822994	0.661025672	-
YKL107W	50.417709	55.31345	-0.133699765	0.629477699	0.662226327	-
tN(GUU)C	2.218063	1.810211	0.293142474	0.629842935	0.662442175	-
YDR261W-A	0.126803	0.111488	0.185700444	0.629842935	0.662442175	-
YDR246W-A	16.140856	19.047432	-0.238879414	0.630467472	0.662930567	-
tE(UUC)G2	0.57043	0.841206	-0.560409282	0.630590949	0.662667561	-
YDR278C	0.082399	0.172848	-1.068805176	0.630590949	0.662667561	-
tG(GCC)D2	0.356616	0.969488	-1.442851609	0.630590949	0.662667561	-
tN(GUU)O2	0.519206	0.731836	-0.49521333	0.630590949	0.662667561	-
YFR012W-A	0.423011	0.676779	-0.677989624	0.630590949	0.662667561	-
tL(UAG)L1	0.477238	0.647989	-0.441260401	0.630590949	0.662667561	-
YHR164C	7.035333	7.973048	-0.180512643	0.63154605	0.663278274	-
YKR032W	0.726225	0.971019	-0.41908293	0.631729744	0.663302876	-
YFL012W	0.503536	0.695221	-0.465376735	0.631729744	0.663302876	-
YJR055W	27.022882	29.282585	-0.11586137	0.640382522	0.672217568	-
YKL023C-A	81.298065	92.610634	-0.187956846	0.640501205	0.672228484	-
YDL213C	42.957527	48.709637	-0.18129629	0.64135756	0.673013477	-
YER069W	16.770781	18.943415	-0.175746559	0.649467281	0.681408295	-
YLR199C	63.206333	69.425041	-0.135387007	0.649868558	0.681714094	-
YDR124W	11.251352	12.175837	-0.11392258	0.650752147	0.682525651	-

snR5	3.032191	2.959866	0.0348287696396	0.651789689	0.683498376	-
YNL290W	30.515465	34.561943	-0.17964375	0.656795999	0.688631914	-
YLR037C	18.324421	21.123379	-0.20507302	0.659370956	0.691214944	-
Q0285	0.814672	0.753082	0.113412362	0.659883952	0.691635923	-
YGL201C	7.175053	8.151881	-0.184143503	0.660310119	0.69196577	-
YBL019W	22.363337	24.507515	-0.132088726	0.661551841	0.693150013	-
YFR045W	24.680094	28.026161	-0.183426249	0.662709875	0.694246187	-
YGR161C-C	1.444468	1.494562	-0.049184503	0.663258227	0.694703404	-
YGL108C	56.005764	61.172691	-0.127312428	0.664687388	0.696082879	-
YPL041C	6.170459	7.227219	-0.228062801	0.666449355	0.697810355	-
YJR161C	38.678783	42.490292	-0.135590856	0.66727773	0.69855989	-
YJL204C	4.076657	4.409661	-0.113281173	0.667371833	0.698540607	-
snR18	36.51474	42.754723	-0.227604841	0.668027044	0.699108545	-
YGR038C-A	0.303809	0.287525	0.0794772063458	0.672743872	0.703807542	-
YJL007C	1.273464	1.279946	-0.007324769	0.672743872	0.703807542	-
YHR021W-A	0.912301	0.84133	0.11683811	0.672743872	0.703807542	-
YJL087C	3.765532	4.069753	-0.112087532	0.672905545	0.703739492	-
YLR068W	12.889072	13.794254	-0.097919042	0.673051714	0.703773799	-
YBR279W	117.778198	132.07576	-0.165293206	0.675945782	0.706680938	-
YHR199C-A	133.978302	146.735062	-0.131214268	0.676265148	0.70689578	-
tK(CUU)D1	0.746121	0.692866	0.106833252	0.676693679	0.707046093	-
tR(ACG)E	0.675542	0.630929	0.0985678037058	0.676693679	0.707046093	-
tT(AGU)I1	0.840899	0.367464	1.194329612	0.676693679	0.707046093	-
YIL170W	0.039588	0.035404	0.161150816	0.676693679	0.707046093	-
YLR412C-A	0.318595	0.379801	-0.253520079	0.676826055	0.706768066	-

YLR227W-A	0.045638	0.059724	-0.388075223	0.676826055	0.706768066	-
YGL138C	0.058429	0.097358	-0.736614933	0.676826055	0.706768066	-
YPR096C	7.730116	9.121469	-0.238776123	0.677114999	0.706832002	-
YAL026C	6.723361	7.612291	-0.179148099	0.678741978	0.708411264	-
YKL106C-A	1.679395	1.556349	0.109775987	0.681035551	0.710685611	-
RDN5-5	3.456467	4.487202	-0.376517979	0.683488524	0.713125506	-
YPL219W	53.292034	59.923492	-0.169201801	0.685743269	0.715357791	-
YIR031C	10.704966	12.164541	-0.184401671	0.686133313	0.715644423	-
YGR018C	1.304367	1.691587	-0.375027531	0.687438083	0.716884868	-
YBR194W	22.24222	25.481091	-0.196126259	0.692921116	0.722481405	-
YKR015C	4.747558	5.435483	-0.195222614	0.695663466	0.725218941	-
YPR072W	47.763363	53.677513	-0.16841341	0.697275806	0.726777737	-
YFL034C-B	19.44812	21.228937	-0.126401432	0.70477863	0.734474691	-
tS(AGA)E	0.87427	1.388771	-0.667657927	0.706158728	0.735789423	-
YLR440C	18.009176	19.792839	-0.136246388	0.70725166	0.736804549	-
YIL127C	11.961758	13.701363	-0.195889983	0.708184764	0.737652855	-
snR36	7.959734	8.364118	-0.071493196	0.709223134	0.738610504	-
YEL030W	12.202953	13.371939	-0.13197837	0.712715363	0.742122941	-
YEL072W	4.698169	5.452045	-0.21469886	0.714990926	0.744367544	-
YFL061W	8.143868	8.774283	-0.107567061	0.716159952	0.745459588	-
YOR342C	19.206013	21.013521	-0.129759859	0.720399714	0.749747096	-
YML034W	108.80452	120.605148	-0.148552999	0.72444225	0.75382794	-
YDR381C-A	152.645905	168.290802	-0.140767441	0.726380614	0.755718259	-
YIR005	28.95250	32.89012	-0.183966214	0.729145513	0.758467719	-

W	7	5				
YGR131W	4.012183	4.708452	-0.230865412	0.730271192	0.759511403	-
YEL001C	50.150295	56.52702	-0.172682456	0.730318896	0.759433787	-
YMR119W	21.074104	23.190187	-0.13804316	0.73041232	0.759403731	-
YCR025C	2.675886	3.166492	-0.24286878	0.730890599	0.759773751	-
tQ(UUG)B	1.083613	1.009916	0.101614305	0.733262706	0.761984412	-
YPL257W-A	0.061116	0.053223	0.199500291	0.733262706	0.761984412	-
snR59	0.996483	0.884646	0.171744931	0.733262706	0.761984412	-
YMR276W	417.144684	466.033295	-0.159885169	0.73366193	0.76214412	-
YDR222W	22.125278	24.964872	-0.174203927	0.735707946	0.764141698	-
YBL069W	7.840239	8.918475	-0.185899406	0.736158305	0.764481559	-
YKL053C-A	126.488686	139.116501	-0.137285206	0.740803776	0.769177093	-
YGL033W	1.940176	2.011441	-0.052041891	0.742485771	0.770794593	-
YER019W	11.261907	12.32641	-0.130301541	0.743146418	0.77135144	-
YBR031W	345.489075	386.052368	-0.160156472	0.746639024	0.774847051	-
YHR042W	35.346596	39.049351	-0.143727286	0.748729075	0.776886194	-
tP(AGG)C	96.394501	109.888718	-0.189020523	0.749712431	0.777776533	-
YLR100W	83.563248	92.379341	-0.144701685	0.749712448	0.777646596	-
YDR268W	9.332585	10.615699	-0.185850721	0.750329842	0.778156976	-
YLR404W	4.239515	4.885312	-0.204551473	0.750727356	0.77843919	-
YAL030W	258.234009	289.515717	-0.164962656	0.751679462	0.779296276	-
YPR097W	18.830393	20.788704	-0.142736711	0.75215406	0.779658107	-
YPL188W	43.441544	48.809196	-0.168077607	0.752309106	0.779688636	-

YNL130C-A	0.922302	1.326725	-0.524558232	0.753310132	0.780595777	-
YKL021C	18.021912	20.335478	-0.174246824	0.75518382	0.782406735	-
YIL120W	18.334587	20.177485	-0.138178594	0.756548951	0.783690286	-
YHR120W	3.28217	3.5746	-0.123131847	0.756587041	0.783598991	-
YCR083W	142.448044	159.983994	-0.167491763	0.757225519	0.784129445	-
YGL198W	133.588959	149.748138	-0.164737289	0.759745533	0.786607783	-
YMR046C	0.241484	0.240745	0.00442176837072	0.762649779	0.789483046	-
YGR289C	64.524399	71.423988	-0.146563891	0.766213477	0.793039891	-
YPR008W	99.26329	111.010864	-0.161368694	0.767877394	0.794629582	-
YJR041C	1.677458	1.912668	-0.189309828	0.771270184	0.79800755	-
tE(UUC)E1	1.496612	1.340306	0.159137834	0.771314017	0.797587643	-
tS(AGA)A	1.215362	0.938192	0.373430986	0.771314017	0.797587643	-
YML040W	0.073144	0.065841	0.151753277	0.771314017	0.797587643	-
YJR026W	0.068445	0.059105	0.211664974	0.771314017	0.797587643	-
YLR406C-A	0.61631	0.654594	-0.086944179	0.771314017	0.797587643	-
YDR210C-C	0.067455	0.066244	0.0261355952554	0.771314017	0.797587643	-
YMR141C	17.187578	18.647629	-0.117625946	0.77186536	0.797692717	-
YLR227C	9.471955	10.388864	-0.133303775	0.775573528	0.801391555	-
RDN5-3	1.753453	2.340652	-0.416711695	0.776032439	0.801732298	-
YML048W	368.019409	408.621918	-0.150984736	0.77645132	0.802031579	-
YIL073C	11.065602	12.197323	-0.140482608	0.787250046	0.813050788	-
YPL119C-A	50.469398	57.096794	-0.178000862	0.788780479	0.814495877	-
snR39	3.17241	4.020604	-0.341833012	0.789034349	0.814622525	-
YAL023	25.89494	29.04252	-0.165496419	0.790327749	0.815822193	-

C	1	1				
YPR105C	9.2898	10.22267<br>2	-0.138052895	0.791194679	0.816581308	-
YOR011<br>W-A	1.462833	1.836248	-0.327995842	0.792138348	0.817419359	-
YOL162<br>W	2.730871	3.172256	-0.216148035	0.792779219	0.817944721	-
YMR289<br>W	24.76557<br>9	27.85962<br>7	-0.169839592	0.793736434	0.818796242	-
YOR343<br>W-A	0.084554	0.079556	0.087902265	0.799436492	0.824470761	-
YPL152<br>W-A	1.302566	1.148591	0.181491312	0.799436492	0.824470761	-
snR84	8.803559	10.01369<br>9	-0.185816213	0.799514953	0.82434626	-
YIL139C	9.893838	11.24846<br>9	-0.185126471	0.799700572	0.824400723	-
YOR087<br>W	8.198195	9.012918	-0.136687959	0.800171265	0.824748997	-
YPR017C	24.17308	26.46285<br>2	-0.13056725	0.800280393	0.824724549	-
YPL053C	80.75449<br>4	90.36824	-0.162273277	0.801002528	0.825331733	-
YHR153<br>C	2.676617	2.848851	-0.089969451	0.802643543	0.82688535	-
YPR114<br>W	23.76598	26.76116<br>4	-0.171242976	0.803548334	0.827680115	-
snR62	4.444202	5.305926	-0.255680163	0.80359611	0.827592012	-
YBR201<br>W	47.19906<br>6	53.07040<br>4	-0.16914922	0.804670411	0.828560941	-
YCL073<br>C	0.329784	0.388635	-0.236894428	0.809150682	0.833036059	-
YOR039<br>W	77.59857<br>2	85.83794<br>4	-0.145585417	0.809518778	0.833276832	-
YBR292<br>C	1.915283	2.307062	-0.268499197	0.810012547	0.833646865	-
YLR316<br>C	25.60875<br>9	28.80386<br>2	-0.169624918	0.81449656	0.838122765	-
YNR011<br>C	11.05266<br>7	12.19995	-0.142480702	0.817927207	0.841513441	-
tF(GAA)<br>M	6.421386	6.689238	-0.058957153	0.821835312	0.845394137	-
YJL065C	96.70936<br>6	106.9624<br>1	-0.145376355	0.825489252	0.84901214	-

YLR095 C	10.96906	12.11045 1	-0.142812694	0.826731278	0.850148712	-
YJL192C	30.12309 5	33.87598 8	-0.169393016	0.827972834	0.851284424	-
YJR151C	5.25493	5.916542	-0.171082674	0.829309773	0.852517813	-
YDL232 W	45.80283	50.11747 4	-0.129876962	0.829503846	0.852576139	-
YDR034 C-D	5.576501	6.158674	-0.143259583	0.83058549	0.853546552	-
YGR199 W	17.89458 8	19.78589 6	-0.144949071	0.833811078	0.856719493	-
YHR084 W	2.351478	2.67879	-0.188013653	0.83563887	0.858455421	-
YHR181 W	26.20255 3	29.50065 6	-0.171039651	0.836165316	0.858854117	-
YAR066 W	5.954097	6.775411	-0.186425739	0.836216464	0.858764567	-
YKL183 C-A	2.304182	2.433789	-0.078949422	0.836804348	0.859226165	-
YPL038 W-A	0.830408	0.837724	-0.012654664	0.839478491	0.861829415	-
YEL028 W	11.35698 3	12.43121 7	-0.13038791	0.842205755	0.86448633	-
YGR109 C	0.456563	0.474248	-0.054827742	0.843678087	0.86585445	-
YMR294 W	22.27520 9	25.00786	-0.166942639	0.843917677	0.86595718	-
YBR297 W	40.59899 9	45.45179	-0.162892955	0.847181317	0.869162389	-
YNL320 W	6.231623	6.818446	-0.129835014	0.847687746	0.869538257	-
YOR033 C	5.498688	6.043238	-0.136234331	0.84783865	0.86954937	-
YMR315 W-A	30.13397 4	34.31996 5	-0.187657137	0.850326953	0.871957338	-
YDR243 C	14.55907 2	16.08424 9	-0.143730175	0.850941937	0.872443855	-
YDR407 C	5.084601	5.608249	-0.141415838	0.852683926	0.874085505	-
YGR035 W-A	7.983037	9.152892	-0.19728996	0.855083078	0.876400157	-
YDR541 C	8.926906	9.82674	-0.13855265	0.857196908	0.878421658	-
YFR008	53.60020	60.063	-0.164238125	0.858189187	0.879293361	-

W	1					
YNL059C	17.240313	19.321747	-0.164439576	0.858596637	0.879565664	-
snR76	27.961372	31.719004	-0.181912318	0.858712178	0.879538889	-
YGR287C	2.994472	3.380012	-0.174726726	0.858789065	0.879472537	-
YLR457C	29.672483	33.266426	-0.164941221	0.861595668	0.882201205	-
YHR014W	1.196315	1.284776	-0.102919535	0.863392604	0.883895331	-
YAL055W	99.762131	111.622269	-0.16206069	0.864721545	0.885109868	-
YDL241W	8.706231	9.484489	-0.123521748	0.865917424	0.886187828	-
snR48	1.784731	1.843865	-0.047026388	0.867616956	0.887780791	-
YLR411W	4.499367	4.906298	-0.124912815	0.867714707	0.887734492	-
YOR305W	21.462851	24.111629	-0.167887396	0.867733719	0.887607666	-
YBL057C	51.713356	57.957638	-0.164461867	0.869205256	0.88896643	-
YNL304W	24.354902	27.2932	-0.164329376	0.871286868	0.890948588	-
YDR020C	13.820471	15.531206	-0.168363076	0.878471125	0.898147026	-
YGR271W	5.065528	5.609804	-0.147237711	0.883523449	0.90316377	-
YNL291C	9.603005	10.765678	-0.164881346	0.88484763	0.904368471	-
YEL033W	251.211105	280.534058	-0.159275689	0.886629523	0.906040507	-
YDL020C	211.233627	235.583801	-0.157400821	0.887711655	0.906997029	-
YMR130W	10.244623	11.315255	-0.143402203	0.887956613	0.907098018	-
YMR281W	38.844349	43.462063	-0.162051923	0.889030445	0.908045575	-
YKL102C	0.801945	0.849063	-0.08236831	0.889106369	0.907824398	-
tW(CCA)K	3.690084	3.833922	-0.055167328	0.889106369	0.907824398	-
snR81	1.317546	1.338768	-0.02305264	0.889106369	0.907824398	-
YNL127	26.0872	29.15049	-0.160178376	0.889416558	0.907842485	-

W		7				
YOR149C	17.221884	19.063021	-0.146533792	0.890652805	0.908954892	-
YMR182C	83.4161	93.245644	-0.160710468	0.891464472	0.909633702	-
YDR118W	9.17347	10.281055	-0.164448853	0.896161772	0.914276464	-
YLR155C	94.670471	105.12188	-0.151076577	0.89653678	0.914508763	-
YJR124C	2.400678	2.713136	-0.176519457	0.896860994	0.914689182	-
tS(UGA)I	9.23998	9.82015	-0.087855333	0.89732042	0.915007419	-
YOL080C	3.692884	4.190211	-0.182274948	0.897849725	0.915396797	-
YLL046C	1.134677	1.311213	-0.208620388	0.898529576	0.915939509	-
YKL122C	54.203709	60.6492	-0.162097041	0.899665435	0.91694681	-
YJR130C	40.148159	44.845993	-0.159645252	0.899830682	0.916964688	-
YCR009C	896.104126	998.777466	-0.156496891	0.90003932	0.917026768	-
YMR150C	44.539986	49.838318	-0.162154275	0.901347965	0.918209413	-
RDN5-6	6.412154	6.912922	-0.108486573	0.901607144	0.918322748	-
YMR077C	29.220076	32.695522	-0.162133126	0.902449897	0.919030341	-
YCL037C	0.602931	0.694878	-0.204766797	0.90249009	0.918920531	-
YGL097W	24.313156	26.949921	-0.148543868	0.904500613	0.92081663	-
YHR194W	26.589191	29.510948	-0.150410384	0.906927171	0.923135576	-
YDR182W-A	3.959455	4.254963	-0.103844723	0.909762261	0.925869527	-
YML037C	5.17731	5.716016	-0.14280725	0.919087301	0.935206354	-
YKL055C	5.528469	6.210419	-0.167810596	0.921714857	0.937726292	-
YEL068C	28.552118	31.559896	-0.144494682	0.923047951	0.938928671	-
YFL045C	81.297363	90.756889	-0.158798599	0.923682551	0.939420262	-
YLR200W	77.996216	87.222534	-0.161296771	0.92415888	0.93975075	-
YER002	84.06747	93.39378	-0.151778807	0.925049497	0.940502338	-

W	4	4				
RDN5-4	3.566072	4.245794	-0.251698544	0.92723969	0.94257475	-
YDR525W	1.121902	1.172789	-0.063996817	0.927293927	0.942475557	-
YDR110W	13.673965	15.165938	-0.149403092	0.928190603	0.943232487	-
YOR236W	48.280903	53.923237	-0.159454445	0.930217284	0.945137299	-
YDL124W	1042.894287	1161.870728	-0.155856634	0.933097247	0.947908314	-
YOR235W	1.25522	1.306939	-0.058251562	0.933278363	0.947937186	-
YOL141W	3.013247	3.333798	-0.145847754	0.935332267	0.94986794	-
YPL161C	9.907656	11.064512	-0.159324139	0.935578865	0.949962969	-
YPL187W	171.95462	191.112762	-0.152396243	0.936375325	0.950616193	-
YEL014C	1.02853	1.199715	-0.22210785	0.936965851	0.950982425	-
YPR158C-C	0.235996	0.285428	-0.274364461	0.936965851	0.950982425	-
YMR073C	43.127987	48.170849	-0.159535972	0.939221345	0.953037939	-
YPL174C	9.981442	11.151614	-0.159932371	0.940571879	0.954252365	-
YGL226W	41.148129	46.013813	-0.161240179	0.941171124	0.954704304	-
YIL049W	10.075619	11.298914	-0.16531564	0.941358034	0.954737898	-
RUF23	1.024079	1.129825	-0.141772316	0.943608799	0.956864329	-
YIR027C	3.163211	3.489965	-0.141822775	0.943920989	0.95702458	-
YOL104C	0.404648	0.433166	-0.098252543	0.944146387	0.957018657	-
snR43	2.063022	2.281216	-0.14504385	0.944146387	0.957018657	-
YDL002C	95.026222	106.019272	-0.157928961	0.945786753	0.958446647	-
YIR013C	0.628247	0.759195	-0.273138614	0.946005786	0.958433933	-
snR66	2.644604	3.1741	-0.26329587	0.946005786	0.958433933	-
YDR446W	0.240812	0.257427	-0.096256092	0.948514962	0.960740885	-
YGL149W	4.246895	4.773498	-0.168638415	0.949224793	0.961303021	-
YLR446W	12.354823	13.710464	-0.150203055	0.951082686	0.963027453	-

snR40	2.007792	2.172357	-0.113651393	0.951153613	0.962942209	-
YHR138C	1840.981812	2048.852539	-0.15434078	0.952321287	0.963967153	-
YBL056W	133.345642	148.602005	-0.156282906	0.952606794	0.964098953	-
YJR062C	61.077827	68.08709	-0.156732539	0.95271496	0.964051258	-
YBL049W	194.11763	215.886368	-0.153340997	0.953022966	0.964205764	-
YPR137C-A	0.12428	0.139131	-0.162849758	0.953943986	0.964980327	-
YHL037C	0.343485	0.407506	-0.246574203	0.956915552	0.967828567	-
YGR156W	8.920007	9.953356	-0.158138203	0.957379077	0.968139649	-
YGR171C	14.591629	16.287535	-0.158627325	0.957526417	0.968130944	-
YLR228C	26.059084	28.977598	-0.15315164	0.957915185	0.968366303	-
YMR233W	16.656309	18.589058	-0.158384925	0.959422129	0.969731777	-
snR79	1.479087	1.689108	-0.191554661	0.960108781	0.970109915	-
YIL134C-A	0.629241	0.679802	-0.11150193	0.960108781	0.970109915	-
snR161	0.719231	0.923189	-0.360170829	0.960108781	0.970109915	-
YGL041C-B	21.829811	24.056372	-0.140119443	0.962182374	0.971888737	-
YLR157C-A	0.075279	0.076718	-0.027317647	0.963581937	0.972985794	-
YGR109W-A	0.105076	0.140992	-0.424180119	0.963581937	0.972985794	-
YER137C-A	0.08291	0.081971	0.0164325215075	0.963581937	0.972985794	-
YHR124W	7.179816	7.981841	-0.152774668	0.963653879	0.972741997	-
YHR214C-E	1.818752	2.029018	-0.157832829	0.966601773	0.975559064	-
YAL037C-A	0.91807	0.867133	0.0823508693169	0.967424431	0.976071976	-
YHR022C-A	0.989563	1.055374	-0.092890883	0.967424431	0.976071976	-
YLR365W	0.234737	0.239639	-0.029817456	0.967424431	0.976071976	-
YGR235	204.0861	227.4298	-0.156243375	0.96875655	0.977098401	-

C	36	4				
YMR306W	1.871006	2.076504	-0.150342466	0.970662246	0.978861472	-
tV(AAC)M1	0.797707	0.905106	-0.182227823	0.97178677	0.979359135	-
tR(UCU)M1	0.6379	0.993514	-0.639210019	0.97178677	0.979359135	-
YPL080C	0.174218	0.190379	-0.12798066	0.97178677	0.979359135	-
YDR098C-A	0.049567	0.047926	0.048571409	0.97178677	0.979359135	-
YDR261C-C	0.043126	0.054908	-0.348458453	0.97178677	0.979359135	-
YBL100W-A	0.046323	0.046039	0.00887219165682	0.97178677	0.979359135	-
YIR020C	0.156845	0.214829	-0.453849219	0.97178677	0.979359135	-
YDL195W	42.44194	47.234802	-0.154359608	0.972887312	0.979831998	-
YPL018W	21.988066	24.443178	-0.152711156	0.972894836	0.979680639	-
YDR370C	7.042268	7.831617	-0.153270081	0.973746831	0.980379554	-
YDL017W	11.079122	12.315712	-0.152656481	0.97423642	0.980713425	-
YHR183W	23.597	26.296364	-0.156259877	0.975729577	0.982057266	-
YLR176C	8.507291	9.462298	-0.153490793	0.975813775	0.981982804	-
tK(UUU)L	0.366904	0.481112	-0.390970151	0.976962394	0.982262943	-
tI(UAU)L	0.513432	0.66426	-0.371574826	0.976962394	0.982262943	-
tN(GUU)G	0.499313	0.683959	-0.453965376	0.976962394	0.982262943	-
tA(AGC)K2	0.494053	0.702573	-0.507982318	0.976962394	0.982262943	-
tY(GUA)D	0.41794	0.38489	0.118849654	0.976962394	0.982262943	-
YCL074W	0.026678	0.048411	-0.859684381	0.976962394	0.982262943	-
tV(AAC)G3	0.447591	0.50157	-0.164270033	0.976962394	0.982262943	-
YGL193C	0.109631	0.118509	-0.112340825	0.976962394	0.982262943	-
tQ(UUG)E2	0.574519	0.489727	0.230376867	0.976962394	0.982262943	-

tK(CUU)P	0.530351	0.471001	0.171217366	0.976962394	0.982262943	-
YER175W-A	15.996576	17.863821	-0.159277565	0.979627063	0.984065502	-
YJR103W	72.116379	80.335732	-0.155714857	0.982256766	0.986547485	-
tM(CAU)D	0.235936	0.061937	1.929522156	0.983708821	0.986569363	-
tV(AAC)L	0.198929	0.16871	0.237708119	0.983708821	0.986569363	-
tF(GAA)H1	0.187129	0.273092	-0.545353897	0.983708821	0.986569363	-
YLR154C-G	0.130145	0.150217	-0.206928205	0.983708821	0.986569363	-
tT(UGU)P	0.101537	0.138426	-0.447109404	0.983708821	0.986569363	-
tA(AGC)G	0.237952	0.33742	-0.503874909	0.983708821	0.986569363	-
YBR219C	0.026462	0.009887	1.420317367	0.983708821	0.986569363	-
tL(UAA)B1	0.008762	0.102431	-3.547248378	0.983708821	0.986569363	-
tA(UGC)L	0.294416	0.011555	4.671266912	0.983708821	0.986569363	-
tL(UAA)J	0.208544	0.208879	-0.002315651	0.983708821	0.986569363	-
tQ(UUG)D1	0.194232	0.213231	-0.134636289	0.983708821	0.986569363	-
YER138W-A	0.073183	0.123721	-0.757509937	0.983708821	0.986569363	-
tT(AGU)B	0.05243	0.069333	-0.403149641	0.983708821	0.986569363	-
tT(AGU)C	0.044364	0.16871	-1.927084135	0.983708821	0.986569363	-
tK(CUU)F	0.018149	0.184887	-3.348681824	0.983708821	0.986569363	-
tK(CUU)J	0.044364	0.057315	-0.369523309	0.983708821	0.986569363	-
tA(UGC)E	0.296432	0.244976	0.275060779	0.983708821	0.986569363	-
YHR214C-D	2.188088	2.493349	-0.188414073	0.984556228	0.985985653	-
YDR231C	261.799469	291.583923	-0.155449005	0.985523849	0.986795493	-
YLR102	79.18523	88.22385	-0.155937355	0.985534762	0.986647283	-

C	4	4				
YLR089C	37.373726	41.628952	-0.155562838	0.993301199	0.994262148	-
YHR094C	3.998156	4.438229	-0.150649344	0.994094832	0.994896134	-
YNL062C	11.995946	13.35978	-0.155349318	0.995456546	0.996098362	-
YKL183W	39.894585	44.431519	-0.155390525	0.995561208	0.996042543	-
YNL046W	83.606918	93.070648	-0.15470393	0.997664239	0.997985755	-
YOR111W	43.407333	48.355057	-0.155727991	0.999018213	0.999179163	-
YOL164W-A	8.457608	9.322373	-0.140447543	0.999418255	0.999418255	-
tI(AAU)P2	0.0001	0.145911	-10.51087293	1	1	-
tI(AAU)P1	0.0001	0.0001	0	1	1	-
tE(UUC)Q	0.0001	0.0001	0	1	1	-
tD(GUC)G1	0.0001	0.0001	0	1	1	-
tE(UUC)G3	0.0001	0.0001	0	1	1	-
tG(GCC)M	0.0001	0.0001	0	1	1	-
tD(GUC)M	0.0001	0.0001	0	1	1	-
tD(GUC)O	0.0001	0.328047	-11.67968681	1	1	-
tK(UUU)Q	0.0001	0.0001	0	1	1	-
tK(UUU)P	0.0001	0.0001	0	1	1	-
tK(UUU)O	0.0001	0.0001	0	1	1	-
tK(UUU)K	0.479938	0.0001	12.22863233	1	1	-
tK(UUU)D	0.195952	0.0001	10.93628458	1	1	-
tY(GUA)F2	0.0001	0.0001	0	1	1	-
tY(GUA)	0.0001	0.0001	0	1	1	-

F1						
tD(GUC)I 2	0.0001	0.0001	0	1	1	-
tD(GUC)I 1	0.0001	0.0001	0	1	1	-
tE(UUC) E3	0.0001	0.0001	0	1	1	-
tK(CUU) D2	0.0001	0.0001	0	1	1	-
tT(UGU) G2	0.0001	0.0001	0	1	1	-
tS(AGA) H	0.20286	0.0001	10.98626871	1	1	-
tS(AGA) L	0.0001	0.0001	0	1	1	-
tS(AGA) B	0.0001	0.022632	-7.822220272	1	1	-
tS(AGA) G	0.0001	0.0001	0	1	1	-
YLL030C	0.0001	0.0001	0	1	1	-
tA(AGC) M1	0.0001	0.0001	0	1	1	-
tA(AGC) M2	0.0001	0.0001	0	1	1	-
tP(UGG) O1	0.0001	0.0001	0	1	1	-
tP(UGG) O2	0.0001	0.0001	0	1	1	-
tP(UGG) O3	0.0001	0.0001	0	1	1	-
tM(CAU) C	0.0001	0.0001	0	1	1	-
tM(CAU) E	0.0001	0.0001	0	1	1	-
YNR077 C	0.053688	0.0001	9.068455852	1	1	-
tG(GCC) D1	0.0001	0.33742	-11.72032977	1	1	-
YDR210 W	0.0001	0.40895	-11.99770875	1	1	-
tI(AAU) N1	0.929236	0.0001	13.18182933	1	1	-
tP(UGG) A	0.0001	0.0001	0	1	1	-

tP(UGG) F	0.479402	0.0001	12.22702021	1	1	-
tP(UGG) H	0.0001	0.0001	0	1	1	-
tP(UGG) L	0.0001	0.0001	0	1	1	-
tP(UGG) Q	0.0001	0.0001	0	1	1	-
tY(GUA) J2	0.0001	0.0001	0	1	1	-
tY(GUA) J1	0.0001	0.0001	0	1	1	-
tQ(UUG) D2	0.0001	0.0001	0	1	1	-
tG(GCC) F1	0.0001	0.0001	0	1	1	-
tG(GCC) F2	0.0001	0.0001	0	1	1	-
tI(UAU) D	0.0001	0.0001	0	1	1	-
YCR097 W	0.0001	0.0001	0	1	1	-
tD(GUC) K	0.0001	0.0001	0	1	1	-
tD(GUC) D	0.0001	0.0001	0	1	1	-
tD(GUC) Q	0.0001	0.0001	0	1	1	-
tE(UUC) P	0.0001	0.33742	-11.72032977	1	1	-
tE(UUC) C	0.0001	0.0001	0	1	1	-
tE(UUC) B	0.0001	0.16871	-10.72032977	1	1	-
tE(UUC)J	0.173787	0.0001	10.76310445	1	1	-
tE(UUC)I	0.038847	0.0001	8.601659379	1	1	-
tE(UUC) M	0.0001	0.0001	0	1	1	-
tE(UUC) L	0.529539	0.0001	12.37052123	1	1	-
tI(GAU) Q	0.0001	0.0001	0	1	1	-
YOL166 W-A	0.0001	0.0001	0	1	1	-

tN(GUU)Q	0.0001	0.0001	0	1	1	-
tN(GUU)K	0.08355	0.0001	9.706496018	1	1	-
tN(GUU)L	0.129304	0.0001	10.33655119	1	1	-
tN(GUU)F	0.0001	0.038758	-8.598350316	1	1	-
tS(UGA)P	0.0001	0.0001	0	1	1	-
tA(AGC)K1	0.542451	0.0001	12.40527711	1	1	-
tY(GUA)Q	0.0001	0.0001	0	1	1	-
tY(GUA)O	0.0001	0.0001	0	1	1	-
tV(AAC)H	0.0001	0.0001	0	1	1	-
tG(GCC)J2	0.114034	0.0001	10.15524832	1	1	-
tL(CAA)G2	0.0001	0.0001	0	1	1	-
tF(GAA)H2	0.0001	0.0001	0	1	1	-
tC(GCA)Q	0.0001	0.0001	0	1	1	-
tV(AAC)K2	0.0001	0.0001	0	1	1	-
YDR194W-A	0.0001	0.0001	0	1	1	-
tR(ACG)Q2	0.0001	0.0001	0	1	1	-
tM(CAU)J1	0.0001	0.330024	-11.68835523	1	1	-
tM(CAU)O1	0.0001	0.0001	0	1	1	-
tM(CAU)O2	0.0001	0.283802	-11.47066904	1	1	-
tQ(UUG)C	0.0001	0.0001	0	1	1	-
tQ(UUG)H	0.0001	0.0001	0	1	1	-
tQ(UUG)Q	0.0001	0.0001	0	1	1	-

tV(AAC)M2	0.0001	0.16415	-10.68079903	1	1	-
tV(AAC)M3	0.0001	0.0001	0	1	1	-
YCR108C	0.0001	0.0001	0	1	1	-
tW(CCA)G1	0.290057	0.0001	11.50212072	1	1	-
tW(CCA)G2	0.0001	0.0001	0	1	1	-
tG(UCC)Q	0.0001	0.0001	0	1	1	-
tG(UCC)O	0.15743	0.0001	10.62049477	1	1	-
tT(AGU)O1	0.0001	0.0001	0	1	1	-
tT(UGU)G1	0.0001	0.0001	0	1	1	-
tG(GCC)C	0.0001	0.0001	0	1	1	-
tG(GCC)B	0.04354	0.0001	8.766197597	1	1	-
tG(GCC)E	0.0001	0.0001	0	1	1	-
tG(CCC)D	0.0001	0.0001	0	1	1	-
tG(CCC)O	0.0001	0.0001	0	1	1	-
tR(CCG)L	0.0001	0.0001	0	1	1	-
tS(AGA)D2	0.242354	0.0001	11.24290018	1	1	-
tS(AGA)D3	0.294416	0.0001	11.52364036	1	1	-
tS(AGA)D1	0.0001	0.0001	0	1	1	-
tE(CUC)D	0.0001	0.028587	-8.159215417	1	1	-
tR(UCU)D	0.0001	0.0001	0	1	1	-
tR(UCU)E	0.294416	0.0001	11.52364036	1	1	-
tR(UCU)K	0.075648	0.0001	9.56315813	1	1	-

tE(CUC)I	0.0001	0.0001	0	1	1	-
YER134C	0.0001	0.0001	0	1	1	-
PWR1	0.0001	0.0001	0	1	1	-
tN(GUU)O1	0.288448	0.0001	11.49409554	1	1	-
tA(AGC)P	0.0001	0.293509	-11.51918903	1	1	-
tA(AGC)L	0.0001	0.16871	-10.72032977	1	1	-
tA(AGC)H	0.0001	0.466841	-12.18871556	1	1	-
tA(AGC)J	0.05848	0.0001	9.191799501	1	1	-
tA(AGC)D	0.0001	0.0001	0	1	1	-
tA(AGC)F	0.0001	0.300442	-11.55287079	1	1	-
RUF22	0.002858	0.0001	4.836934011	1	1	-
YIL174W	0.0001	0.00814	-6.346956889	1	1	-
tT(AGU)I2	0.213754	0.0001	11.0617357	1	1	-
tH(GUG)Q	0.0001	0.0001	0	1	1	-
tH(GUG)H	0.0001	0.0001	0	1	1	-
tH(GUG)M	0.0001	0.0001	0	1	1	-
tV(UAC)B	1.15379	0.0001	13.49409304	1	1	-
tV(UAC)Q	0.0001	0.0001	0	1	1	-
tI(AAU)L2	0.05769	0.0001	9.172177453	1	1	-
tS(CGA)C	0.0001	0.188264	-10.87854144	1	1	-
ZOD1	0.0001	0.0001	0	1	1	-
YGR031C-A	0.0001	0.0001	0	1	1	-
tM(CAU)J3	0.294416	0.0001	11.52364036	1	1	-
tR(UCU)G2	0.0001	0.0001	0	1	1	-
tR(UCU)	0.255569	0.0001	11.31949714	1	1	-

G3						
tT(CGU)K	0.0001	0.0001	0	1	1	-
tK(CUU)E2	0.2803	0.0001	11.45275603	1	1	-
tV(AAC)E1	0.087529	0.0001	9.773617278	1	1	-
tS(UGA)Q2	0.0001	0.0001	0	1	1	-
YOR072W-B	0.0001	0.0001	0	1	1	-
tD(GUC)J4	0.0001	0.0001	0	1	1	-
tD(GUC)J2	0.0001	0.0001	0	1	1	-
tD(GUC)J1	0.25148	0.0001	11.29622795	1	1	-
tP(UGG)N2	0.0001	0.0001	0	1	1	-
YIL014C-A	0.0001	0.0001	0	1	1	-
tK(CUU)G3	0.094778	0.0001	9.888408407	1	1	-
tK(CUU)G2	0.0001	0.0001	0	1	1	-
tT(UGU)Q1	0.0001	0.0001	0	1	1	-
tL(UAA)B2	0.0001	0.0001	0	1	1	-
tD(GUC)L1	0.0001	0.0001	0	1	1	-
tD(GUC)L2	0.226945	0.0001	11.14812699	1	1	-
tG(GCC)G2	0.0001	0.0001	0	1	1	-
tG(GCC)G1	0.228068	0.0001	11.15524832	1	1	-
tQ(CUG)M	0.0001	0.0001	0	1	1	-
YER014C-A	0.0001	0.0001	0	1	1	-
YER190C-B	0.0001	0.0001	0	1	1	-
tI(AAU)I	1.15578	0.0001	13.49657919	1	1	-

1						
tI(AAU)I2	0.0001	0.0001	0	1	1	-
tR(ACG)K	0.209721	0.0001	11.03425561	1	1	-
tR(ACG)L	0.0001	0.0001	0	1	1	-
tR(ACG)D	0.266184	0.0001	11.37820814	1	1	-
tF(GAA)P1	0.0001	0.0001	0	1	1	-
tF(GAA)P2	0.613366	0.0001	12.58253248	1	1	-
tS(GCU)O	0.0001	0.0001	0	1	1	-
tM(CAU)Q2	0.0001	0.0001	0	1	1	-
tM(CAU)Q1	0.0001	0.0001	0	1	1	-
tT(UAG)Q2	0.0001	0.0001	0	1	1	-
tA(UGC)Q	0.0001	0.0001	0	1	1	-
tA(UGC)A	0.0001	0.0001	0	1	1	-
tA(UGC)G	0.0001	0.120177	-10.2309451	1	1	-
tR(UCU)Q1	0.0001	0.0001	0	1	1	-
tD(GUC)J3	0.0001	0.0001	0	1	1	-
tV(CAC)H	0.0001	0.0001	0	1	1	-
YAL067W-A	0.0001	0.196088	-10.93728553	1	1	-
tY(GUA)M2	0.0001	0.0001	0	1	1	-
tY(GUA)M1	0.0001	0.0001	0	1	1	-
tM(CAU)J2	0.0001	0.0001	0	1	1	-
tK(UUU)G2	0.0001	0.0001	0	1	1	-
YMR272	0.0001	0.109349	-10.09472431	1	1	-

W-B						
YIL105W-A	0.0001	0.0001	0	1	1	-
tL(UAA)N	0.0001	0.0001	0	1	1	-
tL(UAA)Q	0.0001	0.0001	0	1	1	-
tS(GCU)Q1	0.0001	0.0001	0	1	1	-
tG(GCC)O1	0.0001	0.087919	-9.780031167	1	1	-
tR(UCU)J2	0.0001	0.0001	0	1	1	-
tR(UCU)J1	0.0001	0.0001	0	1	1	-
tL(CAA)L	0.0001	0.980897	-13.25988594	1	1	-
tL(CAA)K	0.220967	0.0001	11.10961521	1	1	-
tL(CAA)D	0.0001	0.0001	0	1	1	-
tL(CAA)A	0.263399	0.0001	11.36303415	1	1	-
tT(AGU)N2	0.27425	0.0001	11.42127591	1	1	-
tS(GCU)F	0.175114	0.0001	10.77407871	1	1	-
tK(CUU)I	0.0001	0.217303	-11.08549238	1	1	-
YOR068C	0.0001	0.0001	0	1	1	-
YJL077W-A	0.0001	0.0001	0	1	1	-
tI(AAU)B	0.0001	0.0001	0	1	1	-
YOL013W-A	0.38872	0.0001	11.92451562	1	1	-
YFR057W	0.100076	0.0001	9.966880316	1	1	-
tW(UCA)Q	0.0001	0.0001	0	1	1	-
YHR055C	0.0001	0.0001	0	1	1	-
YOL019W-A	0.0001	0.134527	-10.39368004	1	1	-
tH(GUG)	0.0001	0.215574	-11.07396747	1	1	-

G2						
YIL175W	0.062957	0.0001	9.298222986	1	1	-
YCL058 W-A	0.0001	0.0001	0	1	1	-
tW(CCA) J	0.0001	0.0001	0	1	1	-
tT(AGU) O2	0.471871	0.0001	12.20417679	1	1	-
tT(AGU)J	0.0001	0.0001	0	1	1	-
tT(AGU) H	0.0001	0.0001	0	1	1	-
tL(UAG) L2	0.0001	0.0001	0	1	1	-
tF(GAA) B	0.0001	0.0001	0	1	1	-
tF(GAA) D	0.0001	0.0001	0	1	1	-
tF(GAA) F	0.126467	0.0001	10.30454527	1	1	-
tF(GAA) G	0.0001	0.0001	0	1	1	-
tF(GAA) N	0.0001	0.491901	-12.26415227	1	1	-
tF(GAA) Q	0.047106	0.0001	8.879767021	1	1	-
tK(CUU) C	0.0001	0.0001	0	1	1	-
tR(CCU)J	0.057247	0.0001	9.161056281	1	1	-
snR80	0.027548	0.0001	8.105803772	1	1	-
RME3	0.0001	0.0001	0	1	1	-
